# Supplementary material for: Multifaceted Hi-C benchmarking: what makes a difference in chromosome-scale genome scaffolding?
Source: Gigascience. 2020 Jan 10;9(1):giz158. doi: 10.1093/gigascience/giz158 (PMC6952475; doi:10.1093/gigascience/giz158)
Supplement: giz158_GIGA-D-19-00211_Revision_1 [file giz158_giga-d-19-00211_revision_1.pdf]

GigaScience

Multifaceted Hi-C benchmarking: what makes a difference in chromosome-scale genome scaffolding?

--Manuscript Draft--

**Manuscript Number:**	GIGA-D-19-00211R1
**Full Title:**	Multifaceted Hi-C benchmarking: what makes a difference in chromosome-scale genome scaffolding?
**Article Type:**	Research
**Funding Information:**	RIKEN — Dr. Ichiro Hiratani, Dr. Shigehiro Kuraku<br>Ministry of Education, Culture, Sports, Science and Technology (18H05530) — Dr. Ichiro Hiratani
**Abstract:**	Background: Hi-C is derived from chromosome conformation capture (3C) and targets chromatin contacts on a genomic scale. This method has also been used frequently in scaffolding nucleotide sequences obtained by de novo genome sequencing and assembly, in which the number of resultant sequences rarely converges to the chromosome number. Despite its prevalent use, the sample preparation methods for Hi-C have not been intensively discussed, especially from the standpoint of genome scaffolding.<br><br>Results: To gain insight into the best practice of Hi-C scaffolding, we performed a multifaceted methodological comparison using vertebrate samples and optimized various factors during sample preparation, sequencing, and computation. As a result, we identified several key factors that helped improve Hi-C scaffolding, including the choice and preparation of tissues, library preparation conditions, the choice of restriction enzyme(s), and the choice of scaffolding program and its usage.<br><br>Conclusions: This study provides the first comparison of multiple sample preparation kits/protocols and computational programs for Hi-C scaffolding by an academic third party. We introduce a customized protocol designated 'inexpensive and controllable Hi-C (iconHi-C) protocol', which incorporates the optimal conditions identified in this study, and demonstrated this technique on chromosome-scale genome sequences of the Chinese softshell turtle Pelodiscus sinensis.
**Corresponding Author:**	Shigehiro Kuraku<br><br>JAPAN
**Corresponding Author Secondary Information:**	
**Corresponding Author's Institution:**	
**Corresponding Author's Secondary Institution:**	
**First Author:**	Mitsutaka Kadota
**First Author Secondary Information:**	
**Order of Authors:**	Mitsutaka Kadota<br>Osamu Nishimura<br>Hisashi Miura<br>Kaori Tanaka<br>Ichiro Hiratani<br>Shigehiro Kuraku

Powered by Editorial Manager® and ProduXion Manager® from Aries Systems Corporation

**Order of Authors Secondary Information:**	
**Response to Reviewers:**	We have uploaded the PDF-formatted letter including the point-by-point descriptions of our revision of the manuscript as well as the manuscript with track changes.
**Additional Information:**	
**Question**	**Response**
Are you submitting this manuscript to a special series or article collection?	No
**Experimental design and statistics**<br><br>Full details of the experimental design and statistical methods used should be given in the Methods section, as detailed in our Minimum Standards Reporting Checklist. Information essential to interpreting the data presented should be made available in the figure legends.<br><br>Have you included all the information requested in your manuscript?	Yes
**Resources**<br><br>A description of all resources used, including antibodies, cell lines, animals and software tools, with enough information to allow them to be uniquely identified, should be included in the Methods section. Authors are strongly encouraged to cite Research Resource Identifiers (RRIDs) for antibodies, model organisms and tools, where possible.<br><br>Have you included the information requested as detailed in our Minimum Standards Reporting Checklist?	Yes
**Availability of data and materials**<br><br>All datasets and code on which the conclusions of the paper rely must be either included in your submission or deposited in publicly available repositories (where available and ethically appropriate), referencing such data using a unique identifier in the references and in	Yes

Powered by Editorial Manager® and ProduXion Manager® from Aries Systems Corporation

the “Availability of Data and Materials” section of your manuscript.<br><br>Have you have met the above requirement as detailed in our Minimum Standards Reporting Checklist?	

Powered by Editorial Manager® and ProduXion Manager® from Aries Systems Corporation

Manuscript

Click here to view linked References

Click here to access/download;Manuscript;MS-HiC_benchmarking_v3.3-NoEndNote2nd.pdf

Multifaceted Hi-C benchmarking: what makes a difference in chromosome-scale genome scaffolding?

Mitsutaka Kadota[1*], Osamu Nishimura[1*], Hisashi Miura[2], Kaori Tanaka[1,3], Ichiro Hiratani[2], and Shigehiro Kuraku[1]

[1] Laboratory for Phyloinformatics, RIKEN Center for Biosystems Dynamics Research (BDR), Kobe, 650-0047, Japan, [2] Laboratory for Developmental Epigenetics, RIKEN BDR, Kobe, 650-0047, Japan, [3] Present address: Division of Transcriptomics, Medical Institute of Bioregulation, Kyushu University, Fukuoka, 812-0054, Japan

*These authors contributed equally to this study.

Correspondence address. Shigehiro Kuraku, Laboratory for Phyloinformatics, RIKEN BDR, Japan. Tel: +81 78 306 3048; Fax: +81 78 306 3048; E-mail: shigehiro.kuraku@riken.jp

Abstract

Background: Hi-C is derived from chromosome conformation capture (3C) and targets chromatin contacts on a genomic scale. This method has also been used frequently in scaffolding nucleotide sequences obtained by *de novo* genome sequencing and assembly, in which the number of resultant sequences rarely converges to the chromosome number. Despite its prevalent use, the sample preparation methods for Hi-C have not been intensively discussed, especially from the standpoint of genome scaffolding.

Results: To gain insight into the best practice of Hi-C scaffolding, we performed a multifaceted methodological comparison using vertebrate samples and optimized various factors during sample preparation, sequencing, and computation. As a result, we identified several key factors that helped improve Hi-C scaffolding, including the choice and preparation of tissues, library preparation conditions, the choice of restriction enzyme(s), and the choice of scaffolding program and its usage.

Conclusions: This study provides the first comparison of multiple sample preparation kits/protocols and computational programs for Hi-C scaffolding by an academic third party. We introduce a customized protocol designated ‘inexpensive and controllable Hi-C (iconHi-C) protocol’, which incorporates the optimal conditions identified in this study, and demonstrated this technique on chromosome-scale genome sequences of the Chinese softshell turtle *Pelodiscus sinensis*.

Keywords: Hi-C, genome scaffolding, chromosomes, proximity-guided assembly, softshell turtle

Background

Chromatin, a complex of nucleic acids (DNA and RNA) and proteins, exhibits a complex three-dimensional organization in the nucleus, which enables the intricate regulation of the expression of genome information via spatio-temporal control (reviewed in [1]). To characterize chromatin conformation on a genomic scale, the Hi-C method was introduced as a derivative of chromosome conformation capture (3C) (Fig. 1A; [2]). This method detects chromatin contacts on a genomic scale via the digestion of cross-linked DNA molecules with restriction enzymes, followed by proximity ligation of the digested DNA molecules. Massively parallel sequencing of the library containing ligated DNA molecules enables the comprehensive quantification of contacts both within and between chromosomes, which is presented in a heatmap that is conventionally called the 'contact map' [3].

Analyses of chromatin conformation using Hi-C have revealed more frequent contacts between more closely linked genomic regions, which has recently prompted the use of this method in scaffolding *de novo* genome sequences [4-6]. In *de novo* genome sequencing, the number of assembled sequences is usually far larger than the number of chromosomes in the karyotype of the species of interest, regardless of the sequencing platform chosen [7]. The application of Hi-C scaffolding enabled a remarkable enhancement of sequence continuity to reach a chromosome scale, and the integration of fragmentary sequences into longer sequences, which are similar in number to that of chromosomes in the karyotype.

In early 2018, commercial Hi-C library preparation kits were introduced (Fig. 1B), and *de novo* genome assembly was revolutionized by the release of versatile computational programs for Hi-C scaffolding (Table 1), namely LACHESIS [4], HiRise

[8], SALSA [9, 10], and 3d-dna [11] (reviewed in [12]). These movements assisted the rise of mass sequencing projects targeting a number of species, such as the Earth BioGenome Project (EBP) [13], the Genome 10K (G10K)/Vertebrate Genome Project (VGP) [14], and the DNA Zoo Project [15]. Optimization of Hi-C sample preparation, however, has been limited [16], which leaves room for the improvement of efficiency and the reduction of required sample quantity. Thus, the specific factors that are key for Hi-C scaffolding remain unexplored, mainly because of the costly and resource-demanding nature of this technology.

In addition to performing protocol optimization using human culture cells, we focused on the softshell turtle *Pelodiscus sinensis* (Fig. 2). This species has been adopted as a study system for evolutionary developmental biology (Evo-Devo), including the study of the formation of the dorsal shell (carapace) (reviewed in [17]). Access to genome sequences of optimal quality by relevant research communities is desirable in this field. In Japan, live materials (adults and embryos) of this species are available through local farms mainly between May and August, which implies its high utility for sustainable research. A previous cytogenetic report revealed that the karyotype of this species consists of 33 chromosome pairs including Z and W chromosomes (2n = 66) that show a wide variety of sizes (conventionally categorized as macrochromosomes and microchromosomes) [18]. Despite the moderate global GC-content in its whole genome at around 44%, the intragenomic heterogeneity of GC-content between and within the chromosomes has been suggested [19]. A wealth of cytogenetic efforts on this species led to the accumulation of fluorescence *in situ* hybridization (FISH)-based mapping data for 162 protein-coding genes covering almost all chromosomes [18-22], which serve as structural landmarks for validating genome

assembly sequences.

A draft sequence assembly of the softshell turtle genome was built using short reads and was released in 2013 [23]. This sequence assembly achieved the N50 scaffold length of >3.3 Mb but remains fragmented into approximately 20,000 sequences (see Supplementary Table S1). The longest sequence in this assembly is only slightly larger than 16 Mb, which is much shorter than the largest chromosome size estimated from the karyotype report [18]. The total size of the assembly is approximately 2.2 Gb, which is a moderate size for a vertebrate species. Because of the affordable genome size, sufficiently complex structure, and availability of validation methods, we reasoned that the genome of this species is a suitable target for our methodological comparison, and its improved genome assembly is expected to assist a wide range of genome-based studies of this species.

Results

Stepwise QC prior to large-scale sequencing

The assessment of the quality of prepared libraries before engaging in costly sequencing would be ideal. According to the literature [16, 24], we routinely control the quality of Hi-C DNAs and Hi-C libraries by observing DNA size shifts via digestion targeting the restriction sites in properly prepared samples (Fig. 3). More concretely, a successfully ligated Hi-C DNA sample should exhibit a slight increase in the length of its restricted DNA fragments after ligation (QC1), which serves as an indicator of qualified samples (e.g., Sample 1 in Fig. 3B). In contrast, an unsuccessfully prepared Hi-C DNA does not

exhibit this length recovery (e.g., Sample 2 in Fig. 3B). In a subsequent step, DNA molecules in a successfully prepared HindIII-digested Hi-C library should contain the NheI restriction site at a high probability. Thus, the length distribution observed after NheI digestion of the prepared library serves as an indicator of qualified or disqualified products (QC2; Fig. 3C). This series of QCs is incorporated into our protocol by default (Supplementary Protocol S1) and can also be performed in combination with sample preparation using commercial kits if it employs a single restriction enzyme.

Some of the libraries prepared by us passed the QC steps performed before sequencing but yielded an unfavourably large proportion of invalid read pairs. To identify such libraries, we routinely performed small-scale sequencing for quick and inexpensive QC (designated ‘QC3’) using the HiC-Pro program [25] (see Fig. 4 for the read pair categories assigned by HiC-Pro). Our test using variable input data sizes (500 K to 200 M read pairs) resulted in highly similar breakdowns into different categories of read pair properties (Supplementary Table S2) and guaranteed QC3 with an extremely small data size of 1 M or fewer reads. These post-sequencing QC steps, which do not incur a large cost, are expected to help avoid the large-scale sequencing of unsuccessful libraries that have somehow passed through the QC1 and QC2 steps. Importantly, libraries that have passed QC3 can be further sequenced with greater depth, as necessary.

Optimization of sample preparation conditions

We identified overt differences between the sample preparation protocols of published studies and those of commercial kits, especially regarding the duration of fixation and enzymatic reaction as well as the library preparation method used. (Fig. 1B). Therefore,

we first sought to optimize the conditions of several of these steps using human culture cells.

To evaluate the effect of the degree of cell fixation, we prepared Hi-C libraries from GM12878 cells fixed for 10 and 30 minutes. Our comparison did not detect any marked differences in the quality of the Hi-C DNA (QC1; Fig. 5A) and Hi-C library (QC2; Fig. 5B). However, libraries that were prepared with a longer fixation time exhibited a larger proportion of dangling end read pairs and religation read pairs, as well as a smaller proportion of valid interaction reads (Fig. 5C). The increase in the duration of cell fixation also reduced the proportion of long-range (>1 Mb) interactions among the overall captured interactions (Fig. 5D).

The reduced preparation time of commercial Hi-C kits (up to two days according to their advertisement) is attributable mainly to shortened restriction and ligation times (Fig. 1B). To monitor the effect of shortening these enzymatic reactions, we first analysed the progression of restriction and ligation in a time-course experiment using GM12878 cells. We observed the persistent progression of restriction up to 16 hours and of ligation up to 6 hours (Fig. 6). To scrutinize further the possible adverse effects of the prolonged reaction, Hi-C libraries of GM12878 cells were prepared with variable durations of restriction digestion (1 hour and 16 hours) and ligation (15 minutes, 1 hour, and 6 hours). We found that the proportions of dangling end and religation read pairs were reduced in cases with an extended duration of restriction digestion (Supplementary Table S4). The yield of the library, which can be estimated from the number of PCR cycles, increased with the extended duration of ligation without any effect on the proportion of valid interaction read pairs (Supplementary Table S4). The proportion of valid interaction read pairs containing the proper DpnII

junction sequence ‘GATCGATC’ also remained unchanged, suggesting that the prolonged reaction times did not induce any adverse effects, such as star activity of the restriction enzyme.

Multifaceted comparison using softshell turtle samples

Based on the detailed optimization of the sample preparation conditions described above, we built an original protocol, designated the ‘iconHi-C protocol’, that included a 10 minute-long cell fixation, 16 hour-long restriction, 6 hour-long ligation, and successive QC steps (Methods; also see Supplementary Protocol S1; Fig. 1B).

We performed Hi-C sample preparation and scaffolding using tissues from a female Chinese softshell turtle which has both Z and W chromosomes [18]. We prepared Hi-C libraries using various tissues (liver or blood cells), restriction enzymes (HindIII or DpnII), and protocols (our iconHi-C protocol, the Arima kit in conjunction with the KAPA Hyper Prep Kit, or the Phase kit), as outlined in Fig. 7A (see Supplementary Table S5; Supplementary Fig. S1). As in some of the existing protocols (e.g. [26]), we performed T4 DNA polymerase treatment in our iconHi-C protocol (Library a–d), expecting reduced proportions of ‘dangling end’ read pairs that contain no ligated junction, and thus do not contribute to Hi-C scaffolding. We also incorporated this T4 DNA polymerase treatment into the workflow of the Arima kit (Library e vs. Library f without this additional treatment). Furthermore, we tested a lesser degree of PCR amplification (11 cycles) together with the use of the Phase kit which recommends as many as 15 cycles by default (Library h vs. Library g; Fig. 7A).

All samples prepared using the iconHi-C protocol passed both controls, QC1 and QC2 (Fig. 7B). The prepared Hi-C libraries were sequenced to obtain one million

127 nt-long read pairs and were subjected to QC3 using the HiC-Pro program (Fig. 8). As a result of this QC3, the largest proportion of ‘valid interaction’ pairs was observed for Arima libraries (Library e and f). Regarding the iconHi-C libraries (Library a–d), fewer ‘unmapped’ and ‘religation’ pairs were detected for the DpnII libraries compared with HindIII libraries. It should be noted that the QC3 of the softshell turtle libraries generally produced lower proportions of the ‘valid interaction’ category and larger proportions of ‘unmapped pairs’ and ‘pairs with singleton’ than with the human libraries. This cross-species difference may be attributable to the use of incomplete genome sequences as a reference for Hi-C read mapping (Supplementary Table S1). This invokes a caution when comparing QC results across species.

Scaffolding using variable input and computational conditions

In this study, only well-maintained open-source programs, i.e., 3d-dna and SALSA2, were used in conjunction with variable combinations of input libraries, input read amounts, input sequence cut-off lengths, and number of iterative misjoin correction rounds (Fig. 9A). As a result of scaffolding, we observed a wide spectrum of basic metrics, including the N50 scaffold length (0.6–303 Mb), the largest scaffold length (8.7–703 Mb), and the number of chromosome-sized (>10 Mb) sequences (0–65) (Fig. 9; Supplementary Table S6).

First, using the default parameters, 3d-dna consistently produced more continuous assemblies than did SALSA2 (see Assembly 1 vs. 5, 3 vs. 6, 9 vs. 10, and 11 vs. 12 in Fig. 9). Second, the increase in the number of iterative corrections (‘-r’ option of 3d-dna) resulted in relatively large N50 lengths, but with more missing orthologues (see Assembly 3 and 13–14). Third, a smaller input sequence cut-off length (‘-i’ option

of 3d-dna) resulted in a smaller number of scaffolds but again, with more missing orthologues (see Assembly 3 and 15–17). Fourth, the use of the liver libraries consistently resulted in a higher continuity than the use of the blood cell libraries (see Assembly 1 vs. 2 and 3 vs. 4 in Fig. 9).

Assembly 8, which resulted from input Hi-C reads derived from both liver and blood, exhibited an outstandingly large N50 scaffold length (303 Mb) but a larger number of undetected reference orthologues (141 orthologues) than most of the other assemblies. The largest scaffold (scaffold 5) in this assembly is approximately 703 Mb long, causing a large N50 length, and accounts for approximately one-third of the whole genome in length, as a result of possible chimeric assembly that bridged 14 putative chromosomes (see Supplementary Fig. S4).

The choice of restriction enzymes has not been discussed in depth in the context of genome scaffolding. Here, we prepared Hi-C libraries separately with HindIII and DpnII. We did not mix multiple enzymes in the same reaction (other than using the Arima kit which originally employs two enzymes); rather, we performed a single scaffolding run with both HindIII-based and DpnII-based reads (see Assembly 7 in Fig. 9). As expected, our comparison of multiple metrics yielded a more successful result with DpnII than with HindIII (see Assembly 1 vs. 3 as well as 2 vs. 4; Fig. 9). However, the mixed input of HindIII-based and DpnII-based reads did not necessarily yield a better scaffolding result (see Assembly 3 vs. 7).

To gain additional insight regarding the evaluation of the scaffolding results, we assessed the contact maps constructed upon the Hi-C scaffolds (Supplementary Fig. S5). The comparison of Assembly 3, 9 and 11, which represent the three different preparation methods, revealed anomalous patterns, particularly for Assembly 11, with

intensive contact signals separated from the diagonal line that indicate the presence of errors in the scaffolds [15]. We also performed genome-wide alignments between the Hi-C scaffolds obtained. The comparison of Assembly 3, 9, and 11 revealed a high similarity between Assembly 3 and 9, while Assembly 11 exhibited a significantly larger number of inconsistencies against either of the other two assemblies (Supplementary Fig. S6). These observations are consistent with the evaluation based on sequence length and gene space completeness, which alone does not, however, provide a reliable metric for the assessment of the quality of scaffolding.

Validation of scaffolding results using transcriptome and FISH data

In addition to the above-mentioned evaluation of the scaffolding results, we assessed the sequence continuity using independently obtained data. First, we mapped assembled transcript sequences onto our Hi-C scaffold sequences (see Methods). This did not show any substantial differences between the assemblies (Supplementary Table S7), probably because the sequence continuity after Hi-C scaffolding exceeded that of RNA-seq library inserts, even when the length of intervening introns in the genome was considered. The present analysis with RNA-seq data did not provide an effective source of continuity validation.

Second, we referred to the fluorescence *in situ* hybridization (FISH) mapping data of 162 protein-coding genes from published cytogenetic studies [18-22], which allowed us to check the locations of those genes with our resultant Hi-C assemblies. In this analysis, we evaluated Assembly 3, 7, and 9 (see Fig. 9A) that showed better scaffolding results in terms of sequence length distribution and gene space completeness (Fig. 9D). As a result, we confirmed the positioning of almost all genes and their

continuity over the centromeres, which encompassed not only large but also small chromosomes (conventionally called ‘macrochromosomes’ and ‘microchromosomes’; Fig. 10). Two genes that were not confirmed by Assembly 7 (*UCHL1* and *COX15*; Fig. 10) were found in separate scaffold sequences that were shorter than 1 Mb, which indicates insufficient scaffolding. Conversely, the gene array including *RBM5*, *TKT*, *WNT7A*, and *WNT5A*, previously shown by FISH, was consistently unconfirmed by all three assemblies (Fig. 10), which did not provide any clues for among-assembly evaluation or perhaps indicates an erroneous interpretation of FISH data in a previous study.

Discussion

Starting material: not genomic DNA extraction but *in situ* cell fixation

In genome sequencing, best practices for high molecular weight DNA extraction have often been discussed (e.g. [27]). This factor is fundamental to building longer contigs, regardless of the use of short-read or long-read sequencing platforms. Moreover, the proximity ligation method using Chicago libraries provided by Dovetail Genomics which is based on *in vitro* chromatin reconstruction [8], uses genomic DNA as starting material. In contrast, proximity-guided assembly enabled by Hi-C employs cellular nuclei with preserved chromatin conformation, which brings a new technical challenge regarding appropriate sampling and sample preservation in genomics.

In the preparation of the starting material, it is important to optimize the degree of cell fixation depending on sample choice, to obtain an optimal result in Hi-C

scaffolding (Fig. 5). Another practical indication of tissue choice was obtained by examining Assembly 8 (Fig. 9A). This assembly was produced by 3d-dna scaffolding using both liver and blood libraries (Library b and d), which led to an unacceptable result possibly caused by over-assembly (Fig. 9B–D; also see Results). It is likely that increased cellular heterogeneity, which possibly introduces excessive conflicting chromatin contacts, did not allow the scaffolding program to group and order the input genome sequences properly. In brief, we recommend the use of samples with modest cell-type heterogeneity that are amenable to thorough fixation.

Considerations regarding sample preparation

In this study, we did not test all commercial Hi-C kits available in the market. This was partly because the Dovetail Hi-C kit specifies the non-open source program HiRise as the only supported downstream computation solution and does not allow a direct comparison with other kits, namely those from Phase Genomics and Arima Genomics.

According to our calculations, the preparation of a Hi-C library using the iconHi-C protocol would be at least three times cheaper than the use of a commercial kit. Practically, the cost difference would be even larger, either when the purchased kit is not fully consumed or when the post-sequencing computation steps cannot be undertaken in-house, which implies additional outsourcing costs.

The genomic regions that are targeted by Hi-C are determined by the choice of restriction enzymes. Theoretically, 4-base cutters (e.g. DpnII), which potentially have more frequent restriction sites on the genome, are expected to provide a higher resolution than 6-base cutters (e.g., HindIII) [16]. Obviously, the use of restriction enzymes that were not employed in this study might be promising in the adaptation of

the protocol to organisms with variable GC-content or methylation profiles. However, this might not be so straightforward when considering the interspecies variation in GC-content and the intra-genomic heterogeneity. The use of multiple enzymes in a single reaction is a promising approach; however, from a computational viewpoint, not all scaffolding programs are compatible with multiple enzymes (see Table 1 for a comparison of the specification of scaffolding programs). Another technical downside of this approach is the incompatibility of DNA ends restricted by multiple enzymes, with restriction-based QCs, such as the QC2 step of our iconHi-C protocol (Fig. 3). Therefore, in this study, DpnII and HindIII were used separately in the iconHi-C protocol, which resulted in a higher scaffolding performance with the DpnII library (Figs. 8 and 9), as expected. In addition, we input the separately prepared DpnII and HindIII libraries together in scaffolding (Assembly 7), but this approach did not lead to higher scaffolding performance (Figs. 9B–D and 10). The Arima kit employs two different enzymes that can produce a much greater number of restriction site combinations, because one of these two enzymes recognizes the nucleotide stretch 'GANTC'. Scaffolding with the libraries prepared using this kit resulted in one of the most acceptable assemblies (Assembly 9). However, this result did not explicitly exceed the performance of scaffolding with the iconHi-C libraries, including the one that used a single enzyme (DpnII; Library d).

Overamplification by PCR is a concern regarding the use of commercial kits (with the exception of the Arima kit used with the Arima-QC2) because their manuals specify the use of a certain number of PCR cycles *a priori* (15 cycles for the Phase kit and 11 cycles for the Dovetail Hi-C kit) (Supplementary Table S8). In our iconHi-C protocol, an optimal number of PCR cycles is estimated by means of a preliminary real-

time PCR using a small aliquot (Step 11.25 to 11.29 in Supplementary Protocol S1), as done traditionally for other library types (e.g., [28]). This procedure allowed us to reduce the number of PCR cycles, down to as few as five cycles (Supplementary Table S5). The Dovetail Hi-C kit recommends the use of larger amounts of kit components than that specified for a single sample, depending on the genome size, as well as the degree of genomic heterozygosity and repetitiveness, of the species of interest. In contrast, with our iconHi-C protocol, we always prepared a single library, regardless of those species-specific factors, which seemed to suffice in all the cases tested.

Commercial Hi-C kits, which usually advertise easiness and quickness of use, have largely shortened the protocol down to two days, compared with the published non-commercial protocols (e.g., [16, 26]). Such time-saving protocols are achieved mainly by shortening the duration of restriction enzyme digestion and ligation (Fig. 1B). Our assessment, however, revealed unsaturated reaction within the shortened time frames employed in the commercial kits (Fig. 6), which was accompanied by an unfavorable composition of read pairs (Supplementary Table S4). Our attempt to insert a step of T4 DNA polymerase treatment in the sample preparation of the Arima kit protocol resulted in reduced ‘dangling end’ reads (Library e vs. f in Fig. 8). Regarding the Phase kit, transposase-based library preparation contributes largely to its shortened protocol, but this does not allow flexible control of library insert lengths. Recent protocols (versions 1.5 and 2.0) of the Phase kit instruct users to employ a largely reduced DNA amount in the tagmentation reaction, which should mitigate the difficulty in controlling insert length but require excessive PCR amplification. The Arima and Phase kits assume that the quality control of Hi-C DNA is based on the yield, and not the size, of DNA (see Fig. 1B). Nevertheless, quality control based on DNA size

(equivalent to QC1 in iconHi-C) is feasible by taking aliquots at each step of sample preparation. In particular, if preparing a small number of samples for Hi-C, as practised typically for genome scaffolding, one should opt to consider these points, even when using commercial kits, to improve the quality of the prepared libraries and scaffolding products.

Considerations regarding sequencing

The quantity of Hi-C read pairs to be input for scaffolding is critical because it accounts for the majority of the cost of Hi-C scaffolding. Our protocol introduces a thorough safety system to prevent sequencing unsuccessful libraries, first by performing pre-sequencing QCs for size shift analyses (Fig. 3) and second via small-scale (down to 500 K read pairs) sequencing (see Results; also see Supplementary Tables S2 and S9).

Our comparison showed a dramatic decrease in assembly quality in cases in which <100 M read pairs were used (see the comparison of Assembly 18–22 described above; Fig. 9; also see [29]). Nevertheless, we obtained optimal results with a smaller number of reads (ca. 160 M per 2.2 Gb of genome) than that recommended by the manufacturers of commercial kits (e.g., 100 M per 1 Gb of genome for the Dovetail Hi-C kit and 200 M per Gb of genome for the Arima kit). As generally and repeatedly discussed [29][29], the proportion of informative reads and their diversity, rather than just the overall number of obtained reads, is critical.

In terms of read length, we did not perform any comparisons in this study. Longer reads may enhance the fidelity of the characterization of the read pair properties and allow precise QC. Nevertheless, the existing Illumina sequencing platform has enabled the less expensive acquisition of 150 nt-long paired-end reads, which did not

prompt us to vary the read length.

Considerations regarding computation

In this study, 3d-dna produced a more reliable scaffolding output than did SALSA2, whether sample preparation employed a single or multiple enzyme(s) (Fig. 9B–D). On the other hand, 3d-dna required a greater amount of time for the completion of scaffolding than did SALSA2. Apart from the choice of program, several points should be considered if successful scaffolding for a smaller investment is to be achieved. In general, Hi-C scaffolding results should not be taken for granted, and it is necessary to improve them by referring to contact maps using an interactive tool, such as Juicebox [15]. In this study, however, we compared raw scaffolding output to evaluate sample preparation and reproducible computational steps.

We used various parameters of the scaffolding programs (Fig. 9A). First, the Hi-C scaffolding programs that are available currently have different default length cut-off values for input sequences (e.g., 15000 bp for the '-i' parameter in 3d-dna and 1000 bp for the '-c' parameter in SALSA2). Only sequences that are longer than the cut-off length value contribute to sequence scaffolding towards chromosome sizes, while sequences shorter than the cut-off length are implicitly excluded from the scaffolding process and remain unchanged. Typically, when using the Illumina sequencing platform, genomic regions with unusually high frequencies of repetitive elements and GC-content are not assembled into sequences with a sufficient length (see [30]). Such genomic regions tend to be excluded from chromosome-scale Hi-C scaffolds because their length is smaller than the threshold. Alternatively, these regions may be excluded because few Hi-C read pairs are mapped to them, even if they exceed the cut-off length.

The deliberate setting of a cut-off length is recommended if particular sequences with relatively small lengths are the target of scaffolding. It should be noted that lowering the length threshold can result in frequent misjoins in the scaffolding output (Fig. 9B–D) or in overly long computational times. Regarding the number of iterative misjoin correction rounds (the '-r' parameter in 3d-dna and 'i' parameter in SALSA2), our attempts of using increased values did not necessarily yield favourable results (Fig. 9B–D). This did not provide a consistent optimal range of values but rather suggests the importance of performing multiple scaffolding runs with varying parameters.

Considerations regarding the assessment of chromosome-scale genome sequences

Our assessment using cytogenetic data confirmed the continuity of gene linkage over the obtained chromosome-scale sequences (Fig. 10). This validation was required by the almost saturated scores of typical gene space completeness assessment tools such as BUSCO (Supplementary Table S6) and by transcript contig mapping (Supplementary Table S7), neither of which provided an effective metric for evaluation.

For further evaluation of our scaffolding results, we referred to the sequence length distributions of the genome assemblies of other turtle species that are regarded as being chromosome-scale data. This analysis yielded values of the basic metrics that were comparable to those of our Hi-C scaffolds of the softshell turtle, i.e. an N50 length of 127.5 Mb and a maximum sequence length of 344.5 Mb for the genome assembly of the green sea turtle (*Chelonia mydas*) released by the DNA Zoo Project [15] and an N50 length of 131.6 Mb and a maximum length of 370.3 Mb for the genome assembly of the Goode's thornscrub tortoise (*Gopherus evgoodei*) released by the Vertebrate Genome Project (VGP) [14]. Scaffolding results should be evaluated by referring to the

estimated N50 length and the maximum length based on the actual value and to the length distribution of chromosomes in the intrinsic karyotype of the species in question, or of its close relative. Turtles tend to have an N50 length of approximately 130 Mb and a maximum length of 350 Mb, while many teleost fish genomes exhibit an N50 length as low as 20–30 Mb and a maximum length of <100 Mb [31]. If these values are excessive, the scaffolded sequences harbour overassembly, which erroneously boosts length-based metrics. Thus, higher values, which are conventionally regarded as signs of successful sequence assembly, do not necessarily indicate higher precision.

The total length of assembly sequences is expected to increase after Hi-C scaffolding, because scaffolding programs simply insert a stretch of the unassigned base 'N' with a uniform length between input sequences in most cases (500 bp as a default in both 3d-dna and SALSA2). However, this has a minor impact on the total length of assembled sequences. In fact, the insertion of 'N' stretches with an arbitrary length has been an implicit, rampant practice even before Hi-C scaffolding prevailed—for example, the most and second most frequent lengths of the 'N' stretch in the publicly available zebrafish genome assembly Zv10 are 100 and 10 bp, respectively.

Conclusions

In this study, we introduced the iconHi-C protocol which implements successive QC steps. We also assessed potential key factors for improving Hi-C scaffolding. Overall, our study showed that small variations in sample preparation or computation for scaffolding can have a large impact on scaffolding output, and that any scaffolding output should ideally be validated using independent information, such as cytogenetic data, long reads, or genetic linkage maps. The present study aimed to evaluate the

output of reproducible computational steps, which in practice should be followed by the modification of the raw scaffolding output by referring to independent information or by analysing chromatin contact maps. The study employed limited combinations of species, sample prep methods, scaffolding programs, and its parameters, and we will continue to test different conditions for kits/programs that did not necessarily perform well here using our specific materials.

Methods

Initial genome assembly sequences

The softshell turtle (*Pelodiscus sinensis*) assembly published previously [23] was downloaded from NCBI GenBank (GCA_000230535.1), whose gene space completeness and length statistics were assessed by gVolante [32] (see Supplementary Table S1 for the assessment results). Although it could be suggested to remove haplotigs before Hi-C scaffolding [33], we omitted this step because of the low frequency of the reference orthologues with multiple copies (0.72%; Supplementary Table S1), indicating a minimal degree of haplotig contamination.

Animals and cells

We sampled tissues (liver and blood cells) from a female purchased from a local farmer in Japan, because the previous whole genome sequencing used the whole blood of a female [23]. All experiments were conducted in accordance with the Guideline of the Institutional Animal Care and Use Committee of RIKEN Kobe Branch (Approval ID: A2017-12).

The human lymphoblastoid cell line GM12878 was purchased from the Coriell Cell Repositories and cultured in RPMI-1640 medium (Thermo Fisher Scientific) supplemented with 15% FBS, 2 mM L-glutamine, and a 1× antibiotic-antimycotic solution (Thermo Fisher Scientific), at 37 °C, 5% CO_2, as described previously [34].

Hi-C sample preparation using the original protocol

We have made modifications to the protocols that are available in the literature [3, 26, 35] (Fig. 1B). The full version of our 'inexpensive and controllable Hi-C (iconHi-C)' protocol is described in Supplementary Protocol S1 and available at Protocols.io (https://www.protocols.io/private/950FFCBDE7C46D1598CA7DDFE7441C9F).

Hi-C sample preparation using commercial kits

The Proximo Hi-C kit (Phase Genomics) which employs the restriction enzyme Sau3A1 and transposase-based library preparation [36] (Fig. 1B) was used to prepare a library from 50 mg of the softshell turtle liver according to the official ver. 1.0 animal protocol provided by the manufacturer (Library g in Fig. 7A) and a library from 10 mg of the liver that was amplified with a reduced number of PCR cycles based on a preliminary real-time qPCR using an aliquot (Library h; see [28] for the details of the pre-determination of the optimal number of PCR cycles). The Arima-HiC kit (Arima Genomics), which employs a restriction enzyme cocktail (Fig. 1B), was used in conjunction with the KAPA Hyper Prep Kit (KAPA Biosystems), protocol ver. A160108 v00, to prepare a library using the softshell turtle liver, according to its official animal vertebrate tissue protocol (ver. A160107 v00) (Library f) and a library with an additional step of T4 DNA polymerase treatment for reducing 'dangling end' reads

(Library e). This additional treatment is detailed in Step 8.2 (for DpnII-digested samples) of Supplementary Protocol S1.

DNA sequencing

Small-scale sequencing for library QC (QC3) was performed in-house to obtain 127 nt-long paired-end reads on an Illumina HiSeq 1500 in the Rapid Run Mode. For evaluating the effects of variable duration of the restriction digestion and ligation reactions, sequencing was performed on an Illumina MiSeq using the MiSeq Reagent Kit v3 to obtain 300 nt-long paired-end reads. Large-scale sequencing for Hi-C scaffolding was performed to obtain 151 nt-long paired-end reads on an Illumina HiSeq X. The obtained reads underwent quality control using FastQC ver. 0.11.5 (https://www.bioinformatics.babraham.ac.uk/projects/fastqc/), and low-quality regions and adapter sequences in the reads were removed using Trim Galore ver. 0.4.5 (https://www.bioinformatics.babraham.ac.uk/projects/trim_galore/) with the parameters '-e 0.1 -q 30'.

Post-sequencing quality control (QC3) of Hi-C libraries

For post-sequencing library QC, one million trimmed read pairs for each Hi-C library were sampled using the 'subseq' function of the program seqtk ver. 1.2-r94 (https://github.com/lh3/seqtk). The resultant sets of read pairs were processed using HiC-Pro ver. 2.11.1 [25] with bowtie2 ver. 2.3.4.1 [37] to evaluate the insert structure and mapping status onto the softshell turtle genome assembly PelSin_1.0 (GCF_000230535.1) or the human genome assembly hg19. This resulted in categorization as valid interaction pairs and invalid pairs, with the latter being divided

further into ‘dangling end’, ‘religation’, ‘self circle’, and ‘single-end’ pairs (Fig. 4). To process the read pairs derived from the libraries prepared using either HindIII or DpnII (Sau3AI) with the iconHi-C protocol (Library a–d) and the Phase kit (Library g and h), the restriction fragment file required by HiC-Pro was prepared according to the script ‘digest_genome.py’ of HiC-Pro. To process the reads derived from the Arima kit (Library e and f), all restriction sites (‘GATC’ and ‘GANTC’) were inserted into the script. In addition, the nucleotide sequences of all possible ligated sites generated by restriction enzymes were included in a configuration file of HiC-Pro. The details of this procedure and the sample code used are included in Supplementary Protocol S2.

Computation for Hi-C scaffolding

To control our comparison with intended input data sizes, a certain number of trimmed read pairs were sampled for each library with seqtk, as described above. Scaffolding was processed with the following methods employing two program pipelines, 3d-dna and SALSA2.

Scaffolding via 3d-dna was performed using Hi-C read mapping onto the genome with Juicer ver. 20180805 [38] using the default parameters with BWA ver.0.7.17-r1188 [39]. The restriction fragment file required by Juicer was prepared by the script ‘generate_site_positions.py’ script of Juicer. By converting the restriction fragment file of HiC-Pro to the Juicer format, an original script that was compatible with multiple restriction enzymes was prepared (Supplementary Protocol S2). Scaffolding via 3d-dna ver. 20180929 was performed using variable parameters (see Fig. 9A).

Scaffolding via SALSA2 using Hi-C reads was preceded by Hi-C read pair

processing with the Arima mapping pipeline ver. 20181207 (https://github.com/ArimaGenomics/mapping_pipeline) together with BWA, SAMtools ver. 1.8-21-gf6f50ac [40], and Picard ver. 2.18.12 (https://github.com/broadinstitute/picard). The mapping result in the binary alignment map (bam) format was converted into a BED file by bamToBed of Bedtools ver. 2.26.0 [41], the output of which was used as the input of scaffolding using SALSA2 ver. 20181212 with the default parameters.

Completeness assessment of Hi-C scaffolds

gVolante ver. 1.2.1 [32] was used to perform an assessment of the sequence length distribution and gene space completeness based on the coverage of one-to-one reference orthologues with BUSCO v2/v3 employing the one-to-one orthologue set 'Tetrapoda' supplied with BUSCO [42]. No cut-off length was used in this assessment.

Continuity assessment using RNA-seq read mapping

Paired-end reads obtained by RNA-seq of softshell turtle embryos at multiple stages were downloaded from NCBI SRA (DRX001576) and were assembled using Trinity ver. 2.7.0 [43] with default parameters. The assembled transcript sequences were mapped to the Hi-C scaffold sequences with pblat [44], and the output was assessed with isoblat ver. 0.31 [45].

Comparison with chromosome FISH results

Cytogenetic validation of Hi-C scaffolding results was performed by comparing the gene locations on the scaffold sequences with those provided by previous chromosome

FISH for 162 protein-coding genes [18-22]. The nucleotide exonic sequences for those 162 genes were retrieved from GenBank and aligned with Hi-C scaffold sequences using BLAT ver. 36x2 [46], followed by the analysis of their positions and orientation along the Hi-C scaffold sequences.

Availability of supporting data

All sequence data generated in this study have been submitted to the DDBJ Sequence Read Archive (DRA) under accession IDs DRA008313 and DRA008947. The datasets supporting the results of this article are available in FigShare (https://figshare.com/s/6ea495a65fc231a74458).

Additional files

Supplementary Figure S1. DNA size distribution of the softshell turtle Hi-C libraries.

Supplementary Figure S2. Pre-sequencing quality control of softshell turtle blood Hi-C libraries (Library a and b).

Supplementary Figure S3. Pre-sequencing quality control (QC2) of the Hi-C libraries generated using the Phase kit (Library g and h).

Supplementary Figure S4. Structural analysis of the possibly chimeric scaffold in Assembly 8.

Supplementary Figure S5. Hi-C contact maps for selected softshell turtle Hi-C

scaffolds.

Supplementary Figure S6. Pairwise alignment of Hi-C scaffolds.

Supplementary Table S1. Statistics of the Chinese softshell turtle draft genome assembly before Hi-C.

Supplementary Table S2. HiC-Pro results for the human GM12878 HindIII Hi-C library with reduced reads.

Supplementary Table S3. Quality control of the human GM12878 Hi-C libraries.

Supplementary Table S4. Effect of the duration of restriction enzyme digestion and ligation.

Supplementary Table S5. Quality control of Hi-C libraries.

Supplementary Table S6. Scaffolding results with variable input data and computational parameters.

Supplementary Table S7. Mapping results of assembled transcript sequences onto Hi-C scaffolds.

Supplementary Table S8. Effect of variable degrees of PCR amplification.

Supplementary Table S9. HiC-Pro results for the softshell turtle liver libraries (Library d, e, and h) with reduced reads.

Supplementary Protocol S1. iconHi-C protocol.

Supplementary Protocol S2. Computational protocol to support the use of multiple enzymes.

Abbreviations

PCR: polymerase chain reaction; FISH, fluorescence *in situ* hybridization; BUSCO, benchmarking universal single-copy orthologs; NCBI, National Center for Biotechnology Information; NGS, next generation DNA sequencing.

Funding

This work was supported by intramural grants within RIKEN to S.K. and I.H. and by a Grant-in-Aid for Scientific Research on Innovative Areas from the Ministry of Education, Culture, Sports, Science, and Technology (MEXT) to I.H. (18H05530).

Competing interests

The authors declare that they have no competing interests.

Acknowledgements

The authors acknowledge Naoki Irie, Juan Pascual Anaya and Tatsuya Hirasawa in Laboratory for Evolutionary Morphology, RIKEN BDR for suggestions for sampling, Rawin Poonperm for comments and discussion on the iconHi-C protocol, Olga Dudchenko, Erez Lieberman-Aiden, Arang Rhie, Sergey Koren, and Jay Ghurye for their technical suggestions for sample preparation and computation, Yoshinobu Uno for guidance in the cytogenetic data interpretation, and Anthony Schmitt of Arima Genomics and Stephen Eacker of Phase Genomics for providing information about the Hi-C kits. The authors also thank the other members of the Laboratory for Phyloinformatics and Laboratory for Developmental Epigenetics in RIKEN BDR for technical support and discussion.

Author contributions

S.K., I.H., H.M., and M.K. conceived the study. M.K. and K.T. performed laboratory works, and O.N. performed bioinformatic analysis. M.K., O.N., and H.M. analyzed the data. S.K., M.K., and O.N. drafted the manuscript. All authors contributed to the finalization of the manuscript.

References

1. Rowley MJ and Corces VG. Organizational principles of 3D genome architecture. Nat Rev Genet. 2018;19 12:789-800. doi:10.1038/s41576-018-0060-8.

2. Lieberman-Aiden E, van Berkum NL, Williams L, Imakaev M, Ragoczy T, Telling A, et al. Comprehensive mapping of long-range interactions reveals folding

principles of the human genome. Science. 2009;326 5950:289-93. doi:10.1126/science.1181369.

3. Rao SS, Huntley MH, Durand NC, Stamenova EK, Bochkov ID, Robinson JT, et al. A 3D map of the human genome at kilobase resolution reveals principles of chromatin looping. Cell. 2014;159 7:1665-80. doi:10.1016/j.cell.2014.11.021.

4. Burton JN, Adey A, Patwardhan RP, Qiu R, Kitzman JO and Shendure J. Chromosome-scale scaffolding of de novo genome assemblies based on chromatin interactions. Nat Biotechnol. 2013;31 12:1119-25. doi:10.1038/nbt.2727.

5. Marie-Nelly H, Marbouty M, Cournac A, Flot JF, Liti G, Parodi DP, et al. High-quality genome (re)assembly using chromosomal contact data. Nat Commun. 2014;5 1:5695. doi:10.1038/ncomms6695.

6. Kaplan N and Dekker J. High-throughput genome scaffolding from in vivo DNA interaction frequency. Nat Biotechnol. 2013;31 12:1143-7. doi:10.1038/nbt.2768.

7. Sedlazeck FJ, Lee H, Darby CA and Schatz MC. Piercing the dark matter: bioinformatics of long-range sequencing and mapping. Nat Rev Genet. 2018;19 6:329-46. doi:10.1038/s41576-018-0003-4.

8. Putnam NH, O'Connell BL, Stites JC, Rice BJ, Blanchette M, Calef R, et al. Chromosome-scale shotgun assembly using an in vitro method for long-range

linkage. Genome Res. 2016;26 3:342-50. doi:10.1101/gr.193474.115.

9. Ghurye J, Pop M, Koren S, Bickhart D and Chin CS. Scaffolding of long read assemblies using long range contact information. BMC Genomics. 2017;18 1:527. doi:10.1186/s12864-017-3879-z.

10. Ghurye J, Rhie A, Walenz BP, Schmitt A, Selvaraj S, Pop M, et al. Integrating Hi-C links with assembly graphs for chromosome-scale assembly. PLoS Comput Biol. 2019;15 8:e1007273. doi:10.1371/journal.pcbi.1007273.

11. Dudchenko O, Batra SS, Omer AD, Nyquist SK, Hoeger M, Durand NC, et al. De novo assembly of the Aedes aegypti genome using Hi-C yields chromosome-length scaffolds. Science. 2017;356 6333:92-5. doi:10.1126/science.aal3327.

12. Ghurye J and Pop M. Modern technologies and algorithms for scaffolding assembled genomes. PLoS Comput Biol. 2019;15 6:e1006994. doi:10.1371/journal.pcbi.1006994.

13. Lewin HA, Robinson GE, Kress WJ, Baker WJ, Coddington J, Crandall KA, et al. Earth BioGenome Project: Sequencing life for the future of life. Proc Natl Acad Sci USA. 2018;115 17:4325-33. doi:10.1073/pnas.1720115115.

14. Koepfli KP, Paten B, Genome KCoS and O'Brien SJ. The Genome 10K Project: a way forward. Annu Rev Anim Biosci. 2015;3:57-111. doi:10.1146/annurev-animal-

090414-014900.

15. Dudchenko O, Shamim MS, Batra SS, Durand NC, Musial NT, Mostofa R, et al. The Juicebox Assembly Tools module facilitates de novo assembly of mammalian genomes with chromosome-length scaffolds for under $1000. bioRxiv. 2018:254797. doi:10.1101/254797.

16. Belaghzal H, Dekker J and Gibcus JH. Hi-C 2.0: An optimized Hi-C procedure for high-resolution genome-wide mapping of chromosome conformation. Methods. 2017;123:56-65. doi:10.1016/j.ymeth.2017.04.004.

17. Kuratani S, Kuraku S and Nagashima H. Evolutionary developmental perspective for the origin of turtles: the folding theory for the shell based on the developmental nature of the carapacial ridge. Evol Dev. 2011;13 1:1-14. doi:10.1111/j.1525-142X.2010.00451.x.

18. Matsuda Y, Nishida-Umehara C, Tarui H, Kuroiwa A, Yamada K, Isobe T, et al. Highly conserved linkage homology between birds and turtles: bird and turtle chromosomes are precise counterparts of each other. Chromosome Res. 2005;13 6:601-15. doi:10.1007/s10577-005-0986-5.

19. Kuraku S, Ishijima J, Nishida-Umehara C, Agata K, Kuratani S and Matsuda Y. cDNA-based gene mapping and GC3 profiling in the soft-shelled turtle suggest a

chromosomal size-dependent GC bias shared by sauropsids. Chromosome Res. 2006;14 2:187-202. doi:10.1007/s10577-006-1035-8.

20. Uno Y, Nishida C, Tarui H, Ishishita S, Takagi C, Nishimura O, et al. Inference of the protokaryotypes of amniotes and tetrapods and the evolutionary processes of microchromosomes from comparative gene mapping. PLoS One. 2012;7 12:e53027. doi:10.1371/journal.pone.0053027.

21. Kawai A, Nishida-Umehara C, Ishijima J, Tsuda Y, Ota H and Matsuda Y. Different origins of bird and reptile sex chromosomes inferred from comparative mapping of chicken Z-linked genes. Cytogenet Genome Res. 2007;117 1-4:92-102. doi:10.1159/000103169.

22. Kawagoshi T, Uno Y, Matsubara K, Matsuda Y and Nishida C. The ZW micro-sex chromosomes of the Chinese soft-shelled turtle (Pelodiscus sinensis, Trionychidae, Testudines) have the same origin as chicken chromosome 15. Cytogenet Genome Res. 2009;125 2:125-31. doi:10.1159/000227837.

23. Wang Z, Pascual-Anaya J, Zadissa A, Li W, Niimura Y, Huang Z, et al. The draft genomes of soft-shell turtle and green sea turtle yield insights into the development and evolution of the turtle-specific body plan. Nat Genet. 2013;45 6:701-6. doi:10.1038/ng.2615.

24. Belton JM, McCord RP, Gibcus JH, Naumova N, Zhan Y and Dekker J. Hi-C: a comprehensive technique to capture the conformation of genomes. Methods. 2012;58 3:268-76. doi:10.1016/j.ymeth.2012.05.001.

25. Servant N, Varoquaux N, Lajoie BR, Viara E, Chen CJ, Vert JP, et al. HiC-Pro: an optimized and flexible pipeline for Hi-C data processing. Genome Biol. 2015;16:259. doi:10.1186/s13059-015-0831-x.

26. Sofueva S, Yaffe E, Chan WC, Georgopoulou D, Vietri Rudan M, Mira-Bontenbal H, et al. Cohesin-mediated interactions organize chromosomal domain architecture. Embo j. 2013;32 24:3119-29. doi:10.1038/emboj.2013.237.

27. Mayjonade B, Gouzy J, Donnadieu C, Pouilly N, Marande W, Callot C, et al. Extraction of high-molecular-weight genomic DNA for long-read sequencing of single molecules. Biotechniques. 2016;61 4:203-5. doi:10.2144/000114460.

28. Tanegashima C, Nishimura O, Motone F, Tatsumi K, Kadota M and Kuraku S. Embryonic transcriptome sequencing of the ocellate spot skate Okamejei kenojei. Sci Data. 2018;5:180200. doi:10.1038/sdata.2018.200.

29. DeMaere MZ and Darling AE. bin3C: exploiting Hi-C sequencing data to accurately resolve metagenome-assembled genomes. Genome Biol. 2019;20 1:46. doi:10.1186/s13059-019-1643-1.

30. Botero-Castro F, Figuet E, Tilak MK, Nabholz B and Galtier N. Avian Genomes Revisited: Hidden Genes Uncovered and the Rates versus Traits Paradox in Birds. Mol Biol Evol. 2017;34 12:3123-31. doi:10.1093/molbev/msx236.
31. Hotaling S and Kelley JL. The rising tide of high-quality genomic resources. Mol Ecol Resour. 2019;19 3:567-9. doi:10.1111/1755-0998.12964.
32. Nishimura O, Hara Y and Kuraku S. gVolante for standardizing completeness assessment of genome and transcriptome assemblies. Bioinformatics. 2017;33 22:3635-7. doi:10.1093/bioinformatics/btx445.
33. Roach MJ, Schmidt SA and Borneman AR. Purge Haplotigs: allelic contig reassignment for third-gen diploid genome assemblies. BMC Bioinformatics. 2018;19 1:460. doi:10.1186/s12859-018-2485-7.
34. Kadota M, Hara Y, Tanaka K, Takagi W, Tanegashima C, Nishimura O, et al. CTCF binding landscape in jawless fish with reference to Hox cluster evolution. Sci Rep. 2017;7 1:4957. doi:10.1038/s41598-017-04506-x.
35. Ikeda T, Hikichi T, Miura H, Shibata H, Mitsunaga K, Yamada Y, et al. Srf destabilizes cellular identity by suppressing cell-type-specific gene expression programs. Nat Commun. 2018;9 1:1387. doi:10.1038/s41467-018-03748-1.
36. Adey A, Morrison HG, Asan, Xun X, Kitzman JO, Turner EH, et al. Rapid, low-

input, low-bias construction of shotgun fragment libraries by high-density in vitro transposition. Genome Biol. 2010;11 12:R119. doi:10.1186/gb-2010-11-12-r119.

37. Langmead B and Salzberg SL. Fast gapped-read alignment with Bowtie 2. Nat Methods. 2012;9 4:357-9. doi:10.1038/nmeth.1923.

38. Durand NC, Shamim MS, Machol I, Rao SS, Huntley MH, Lander ES, et al. Juicer Provides a One-Click System for Analyzing Loop-Resolution Hi-C Experiments. Cell Syst. 2016;3 1:95-8. doi:10.1016/j.cels.2016.07.002.

39. Li H and Durbin R. Fast and accurate short read alignment with Burrows-Wheeler transform. Bioinformatics. 2009;25 14:1754-60. doi:10.1093/bioinformatics/btp324.

40. Li H. A statistical framework for SNP calling, mutation discovery, association mapping and population genetical parameter estimation from sequencing data. Bioinformatics. 2011;27 21:2987-93. doi:10.1093/bioinformatics/btr509.

41. Quinlan AR and Hall IM. BEDTools: a flexible suite of utilities for comparing genomic features. Bioinformatics. 2010;26 6:841-2. doi:10.1093/bioinformatics/btq033.

42. Simao FA, Waterhouse RM, Ioannidis P, Kriventseva EV and Zdobnov EM. BUSCO: assessing genome assembly and annotation completeness with single-

copy orthologs. Bioinformatics. 2015;31 19:3210-2. doi:10.1093/bioinformatics/btv351.

43. Grabherr MG, Haas BJ, Yassour M, Levin JZ, Thompson DA, Amit I, et al. Full-length transcriptome assembly from RNA-Seq data without a reference genome. Nat Biotechnol. 2011;29 7:644-52. doi:10.1038/nbt.1883.

44. Wang M and Kong L. pblat: a multithread blat algorithm speeding up aligning sequences to genomes. BMC Bioinformatics. 2019;20 1:28. doi:10.1186/s12859-019-2597-8.

45. Ryan JF. Baa.pl: A tool to evaluate de novo genome assemblies with RNA transcripts. arXiv e-prints. 2013;arXiv:1309.2087.

46. Kent WJ. BLAT--the BLAST-like alignment tool. Genome Res. 2002;12 4:656-64. doi:10.1101/gr.229202.

47. Imakaev M, Fudenberg G, McCord RP, Naumova N, Goloborodko A, Lajoie BR, et al. Iterative correction of Hi-C data reveals hallmarks of chromosome organization. Nat Methods. 2012;9 10:999-1003. doi:10.1038/nmeth.2148.

Table 1: Overview of the specification of major scaffolding programs.

Program	Support and availability	Input data requirement	Other information	Literature
LACHESIS	Developer's support discontinued; intricate installation	Generic bam format	No function to correct scaffold misjoins	[4]
HiRise	Open source version at GitHub not updated since 2015	Generic bam format	Employed in Dovetail Chicago/Hi-C service. Default input sequence length cut-off=1000 bp	[8]
3d-dna	Actively maintained and supported by the developer	Not compatible with multiple enzymes; Accept only Juicer mapper format	Default parameters: -t 15000 (input sequence length cut-off), -r 2 (no. of iterations for misjoin correction)	[11, 38]
SALSA2	Actively maintained and supported by the developer	Compatible with multiple enzymes; generic bam (bed) file, assembly graph, unitig, 10x link files	Default parameters: -c 1000 (input sequence length cut-off), -i 3 (no. of iterations for misjoin correction)	[9, 10]

Figures

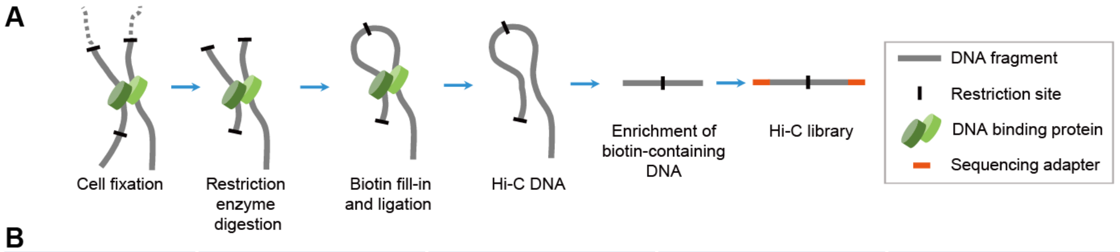

B

Different specifications	iconHi-C (Our protocol)	Arima-HiC Kit (ver. A160107 v00, with the KAPA Hyper Prep Kit)	Phase Proximo Hi-C Kit (Animal ver. 1.0)	Dovetail Hi-C Kit (ver. 1.4, with Dovetail Library Module and Primer Set)
Cell fixation	10 min (cells) or 15 min (tissue) in 1 % formaldehyde at 25°C; up to 1×10^7 cells or up to 1 cm^3 tissue	10 min (cells) or 20 min (tissue) in 2 % formaldehyde at RT; 0.5-1 ×10^7 cells or 100-500 mg tissue	15 min in crosslinking solution (included in the kit) at RT; 1×10^7 cells or 100 mg tissue	20 min in 1.5 % formaldehyde at RT; 0.5×10^6 cells and 20-40 mg tissue
Sample amount for restriction digestion and ligation	1-2×10^6 cells or tissue estimated to contain 2-10 μg DNA	Cells or tissue estimated to contain 750 ng - 5 μg DNA	1×10^7 cells or 100 mg tissue	0.5×10^6 cells or 20-40 mg tissue
Restriction enzyme digestion	HindIII (cuts at "AAGCTT") or DpnII (cuts at "GATC"), 16 hrs at 37°C	Cocktail of A1 and A2 enzymes (cuts at "GATC" and "GANTC"), 30-60 min at 37°C	Sau3AI (cuts at "GATC"), 1 hr at 37°C	DpnII (cuts at "GATC"), 1 hr at 37°C
Ligation	6 hrs at 16°C	15 min at RT	4 hrs at RT	1-16 hrs at 16°C
Reverse crosslinking	16 hrs at 65°C	1.5-16 hrs at 68°C	1-16 hrs at 60°C	45 min at 68°C
Hi-C DNA extraction	Phenol/chloroform extraction	DNA purification beads (e.g. AMPure XP)	Spin column (included in the kit)	SPRIselect beads
Hi-C DNA QC	Check for the size shift before and after ligation (QC1)	Check for the yield of biotin-labeled DNA (Arima-QC1)	Check for the DNA yield before proximity ligation	Check for the DNA yield
DNA amount for library preparation	250 ng - 2 μg	125 ng - 2 μg	N/A	200 ng
Removal of biotin from un-ligated DNA ends	By T4 DNA polymerase	N/A	N/A	N/A
DNA fragmentation	Sonication (Covaris)	Sonication (Covaris or Diagenode)	Transposase	Sonication (Covaris or Diagenode)
Library preparation	Adapter ligation-based (KAPA LTP Library Prep Kit)	Adapter ligation-based	Transposase-based (included in the kit)	Adapter ligation-based
PCR cycles	Pre-determination by qPCR (KAPA Real-time Library Amplification Kit)	Pre-determination by qPCR (KAPA Library Quantification Kit; Arima-QC2)	15 cycles	11 cycles
Size selection	After DNA fragmentation	After DNA fragmentation	After PCR	After PCR
Hi-C library QC	Check for yield and size distribution; check for size shift by NheI or ClaI digestion (QC2)	Check for yield and size distribution	Check for yield and size distribution	Check for yield and size distribution

Figure 1: Hi-C library preparation. (A) Basic procedure. (B) Comparison of Hi-C library preparation methods. Only the major differences between the methods are included here. The versions of the Arima and Phase kits used in this study are presented. The KAPA Hyper Prep Kit (KAPA Biosystems) is assumed to be conjunctly used with Arima Hi-C Kit, among the several specified kits. See Supplementary Protocol S1 for the full version of the iconHi-C protocol which was derived from the protocols published previously [3, 26, 35].

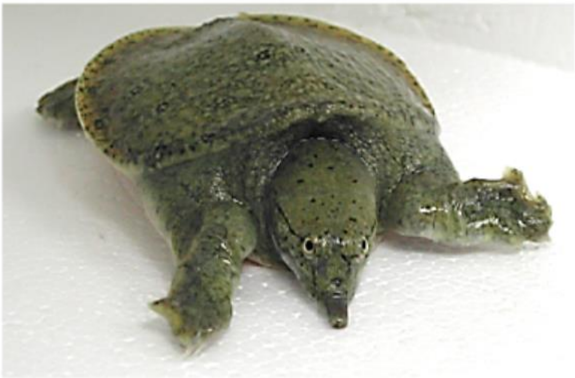

Figure 2: A juvenile softshell turtle *Pelodiscus sinensis*.

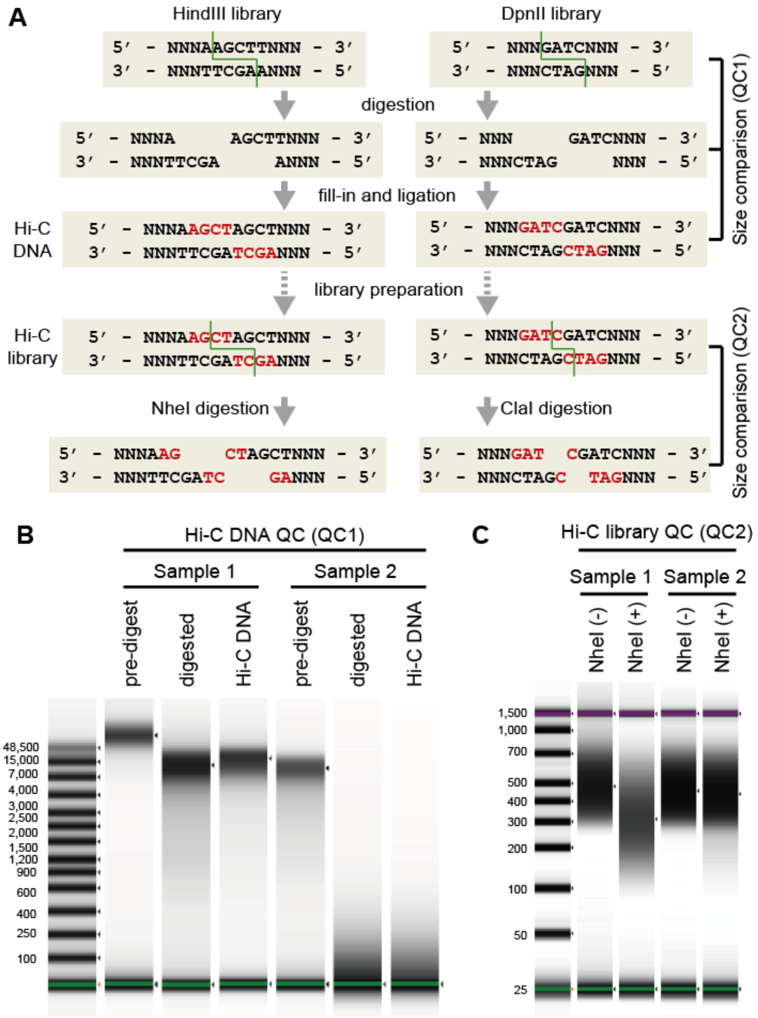

Figure 3: Structure of the Hi-C DNA and principle of the quality controls. (A) Schematic representation of the library preparation workflow based on HindIII or DpnII digestion. The patterns of restriction are indicated by the green lines. The nucleotides that are filled in are indicated by the letters in red. (B) Size shift analysis of HindIII-digested Hi-C DNA (QC1). Representative images of qualified (Sample 1) and disqualified (Sample 2) samples are shown. (C) Size shift analysis of the HindIII-digested Hi-C library (QC2). Representative images of the qualified (Sample 1) and disqualified (Sample 2) samples are shown. Size distributions were measured with

Agilent 4200 TapeStation.

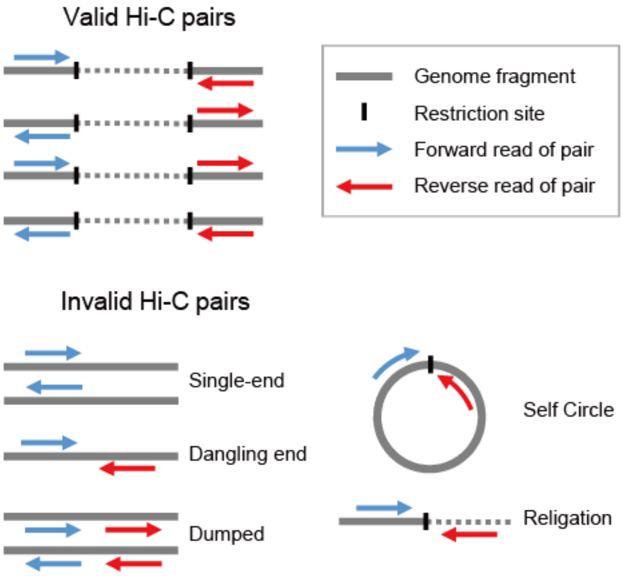

Figure 4: Post-sequencing quality control of Hi-C reads. Read pairs were categorized into valid and invalid pairs by HiC-Pro, based on their status in the mapping to the reference genome (see Methods). This figure was adapted from the article that described HiC-Pro originally [25].

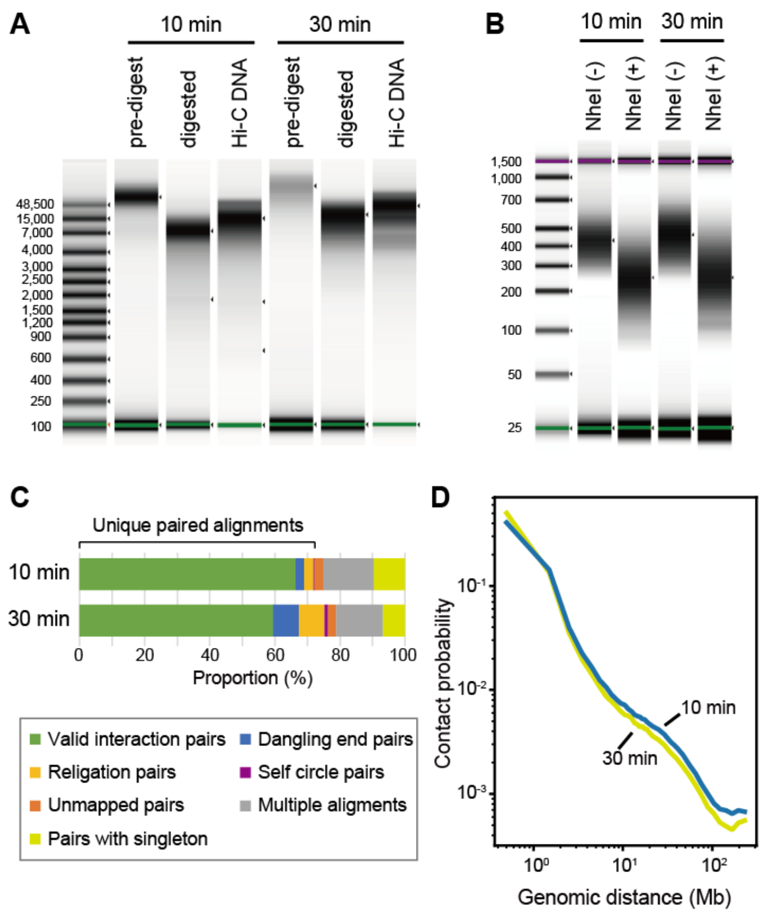

Figure 5: Effect of cell fixation duration. (A) QC1 of the HindIII-digested Hi-C DNA of human GM12878 cells fixed for 10 or 30 minutes in 1% formaldehyde. (B) QC2 of the HindIII-digested library of human GM12878 cells. (C) Quality control of the sequence reads by HiC-Pro using 1 M read pairs. See Fig. 4 for the details of the read pair categorization. See Supplementary Table S3 for the actual proportion of the reads in each category. (D) Contact probability measured by the ratio of observed and expected frequencies of Hi-C read pairs mapped along the same chromosome [47].

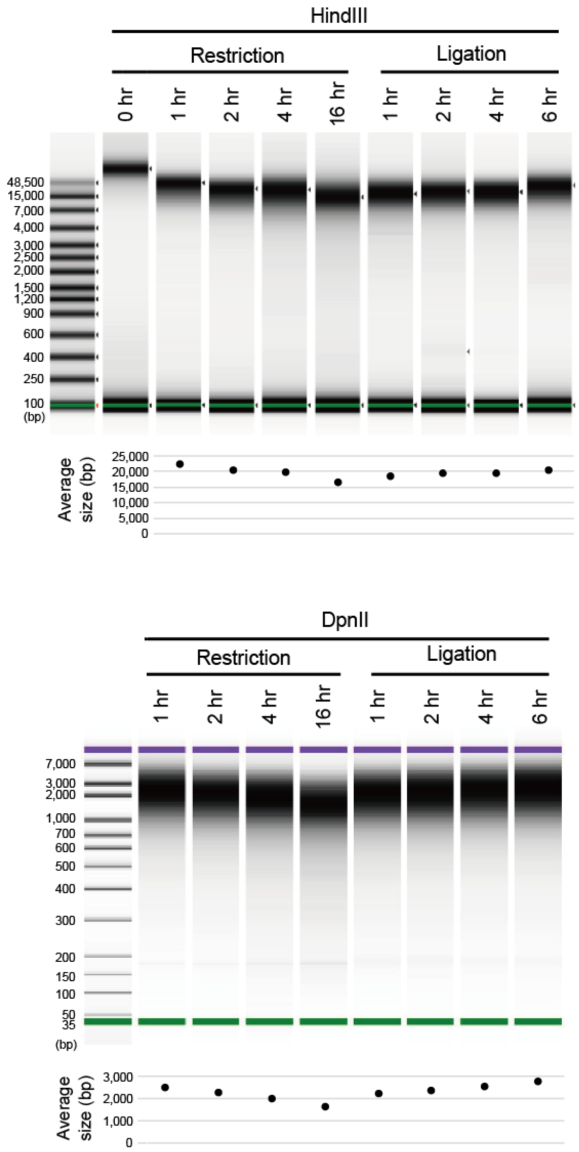

Figure 6: Testing varying durations of restriction and ligation. The length distributions of the DNA molecules prepared from human GM12878 cells after restriction and ligation of variable duration are shown. The size distributions of the HindIII-digested samples (top) and DpnII-digested samples (bottom) were measured with an Agilent 4200 TapeStation and an Agilent Bioanalyzer, respectively.

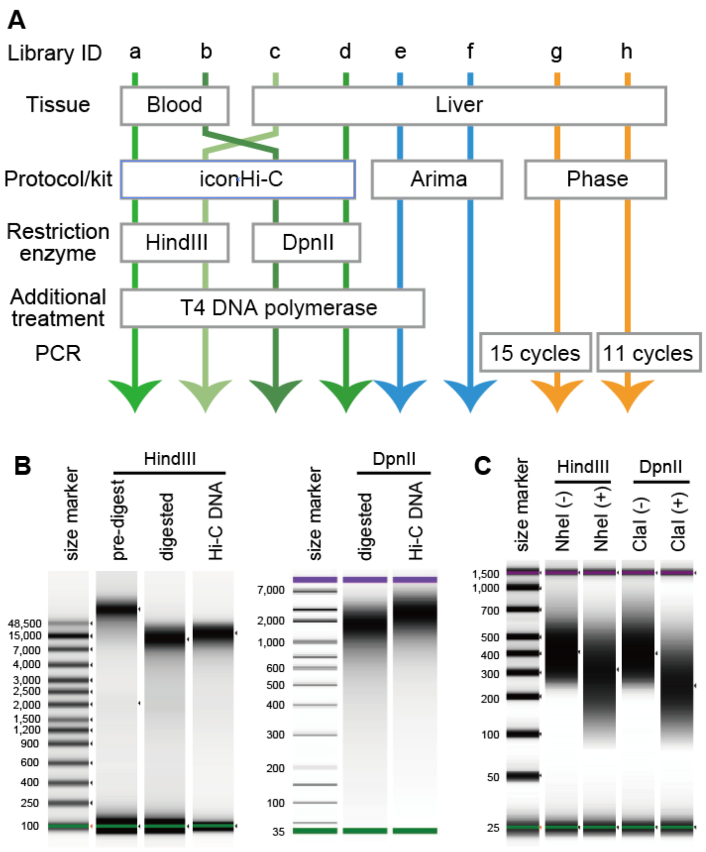

Figure 7: Softshell turtle Hi-C libraries prepared for our methodological comparison. (A) Lineup of the prepared libraries. This chart includes only the conditions in preparation methods that varied between these libraries, and the remainder preparation workflows are described in Supplementary Protocol S1 for the non-commercial ('iconHi-C') protocol and in the manuals of the commercial kits. (B) Quality control of Hi-C DNA (QC1) for Library c and d. The Hi-C DNA for the Chinese softshell turtle liver sample was prepared with either HindIII or DpnII digestion. (C) Quality control of Hi-C libraries (QC2). The HindIII library prepared from the softshell turtle liver was digested by NheI, and the DpnII library was digested by ClaI (see Fig. 3 for the technical principle). See Supplementary Fig. S2 for the QC1 and QC2 results of the samples prepared from the blood of this species. See Supplementary Fig. S3 for the

QC2 result of the Phase libraries.

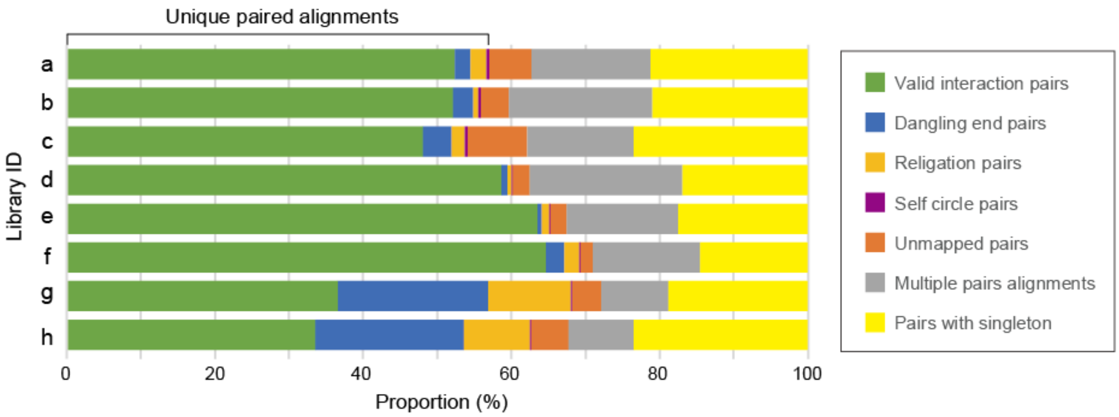

Figure 8: Results of the post-sequencing quality control with HiC-Pro. One million read pairs were used for computation with HiC-Pro. See Fig. 7A for the preparation conditions of Library a-h, Fig. 4 for the categorization, and Supplementary Table S5 for the actual proportion of the reads in each category. The post-sequencing quality control using variable read amounts (500 K to 200 M pairs) for one of these softshell turtle libraries (Supplementary Table S9) and human GM12878 libraries (Supplementary Table S2) shows the validity of this quality control with as few as 500 K read pairs.

A

Assembly ID	Library ID	Scaffolding program	Input sequence length cutoff (nt)	Number of iterative misjoin correction rounds	Number of read pairs input
1	c	3d-dna	15000	2	200 M
2	a				
3	d				
4	b				
5	c	SALSA2	1000	3	
6	d				
7	c + d	3d-dna	15000	2	
8	b + d				
9	e				
10		SALSA2	1000	3	
11	h	3d-dna	15000	2	
12		SALSA2	1000	3	
13	d	3d-dna	15000	4	
14				6	
15			10000	2	
16			5000		
17			3000		
18			15000		280 M
19					160 M
20					80 M
21					20 M
22					10 M

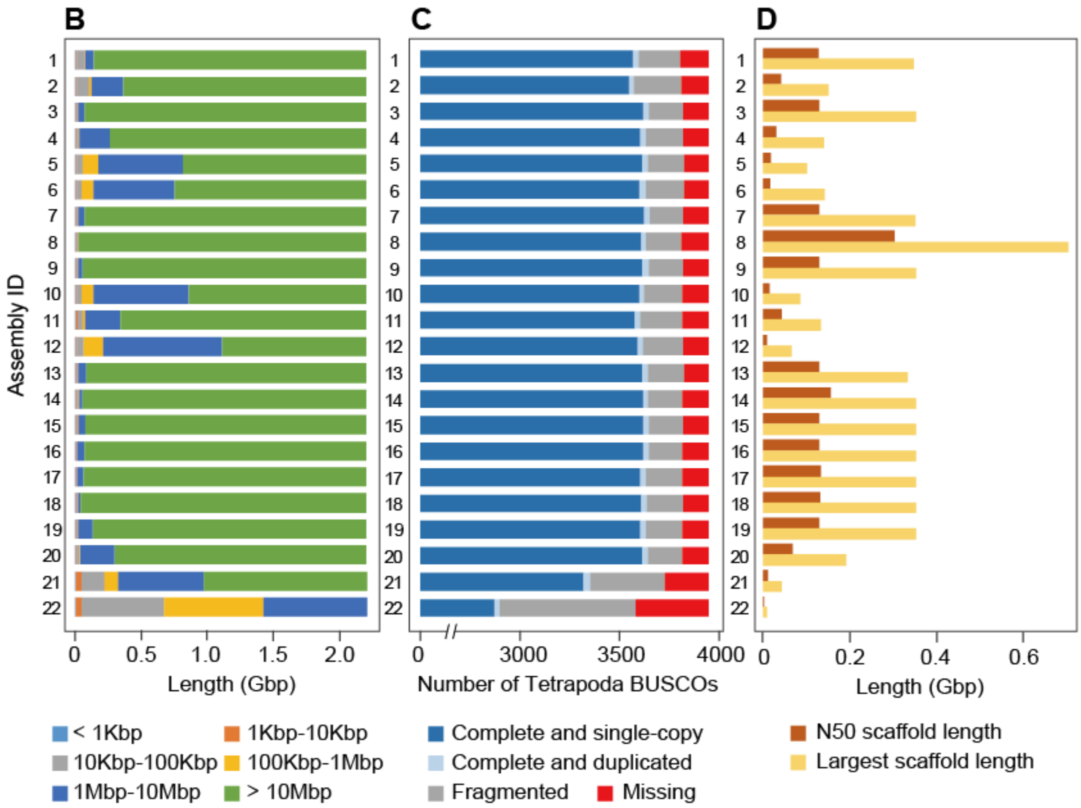

Figure 9: Comparison of Hi-C scaffolding products. (A) Scaffolding conditions used to produce Assembly 1 to 22. The default parameters are shown in red. (B) Scaffold length

distributions. (C) Gene space completeness. (D) Largest and N50 scaffold lengths. See the panel A for Library IDs and Supplementary Table S6 for raw values of the metrics shown in B–D.

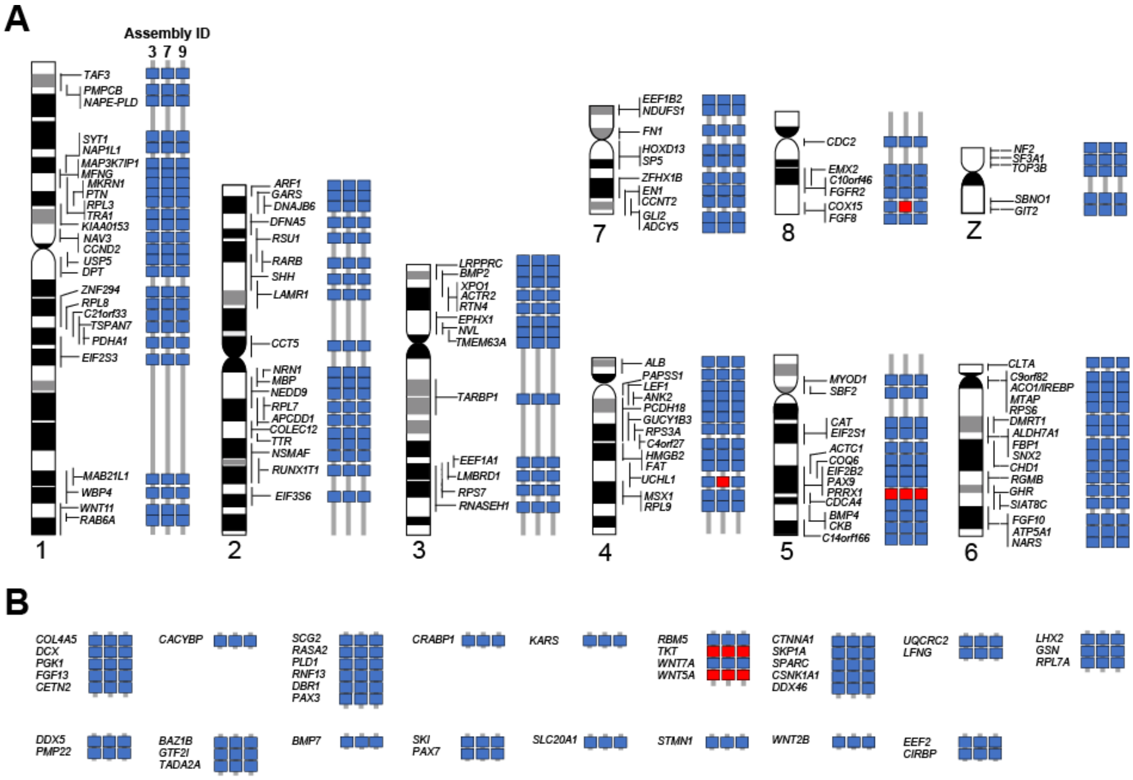

Figure 10: Cytogenetic validation of Hi-C scaffolding results. For the scaffolded sequences of Assembly 3, 7, and 9, we evaluated the consistency of the positions of the selected genes that were previously localized on eight macrochromosomes and Z chromosome (A) and microchromosomes (B) by chromosome FISH [18-22] (see Results). Concordant and discordant gene locations on individual assemblies are indicated with blue and red boxes, respectively. The arrays of genes without idiograms in B were identified on chromosomes that are cytogenetically indistinguishable from each other.

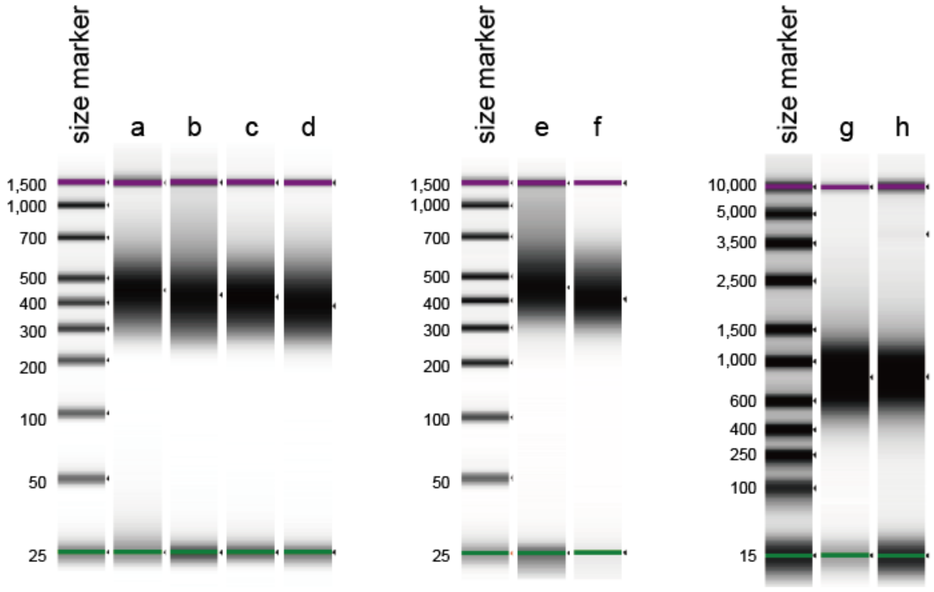

Supplementary Figure S1: DNA size distribution of the softshell turtle Hi-C libraries. The size distribution of the libraries was analysed by an Agilent 4200 TapeStation using the High Sensitivity D1000 kit for Library a-f and the High Sensitivity D5000 kit for Library g and h.

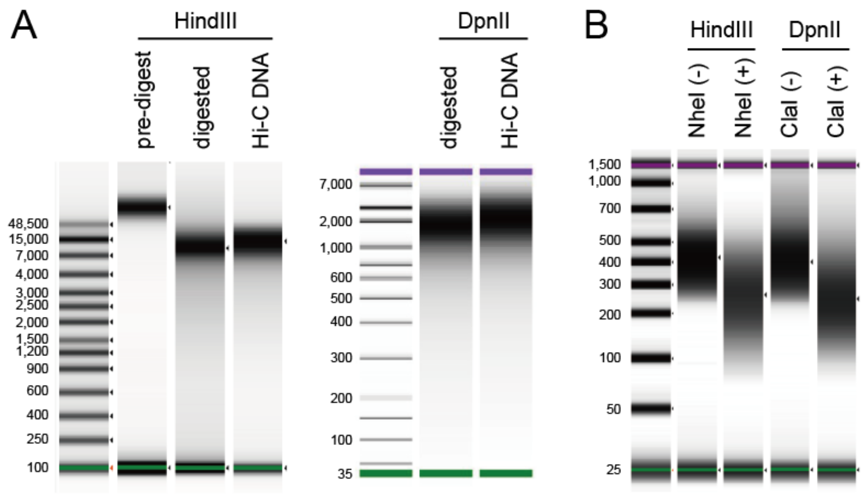

Supplementary Figure S2: Pre-sequencing quality control of softshell turtle blood Hi-C libraries (Library a and b). (A) Quality control of Hi-C DNAs (QC1). Hi-C DNA was prepared from the Chinese softshell turtle blood by HindIII or DpnII digestion (see Fig. 7A for the details). (B) Quality control of Hi-C libraries (QC2). The softshell turtle blood library prepared using HindIII was digested by NheI, and the library prepared using DpnII was digested by ClaI (see Fig. 3 for the technical principle).

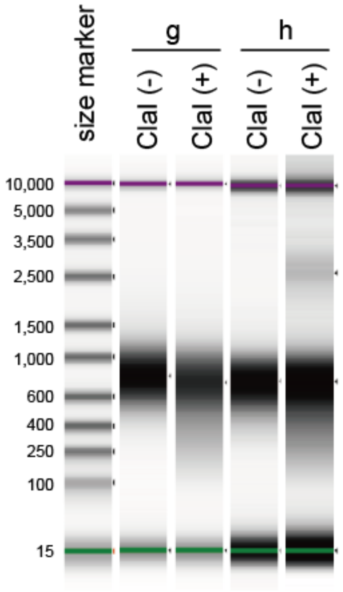

Supplementary Figure S3: Pre-sequencing quality control (QC2) of the Hi-C libraries prepared using the Phase kit (Library g and h). The softshell turtle liver libraries prepared using Sau3A1 were digested by ClaI.

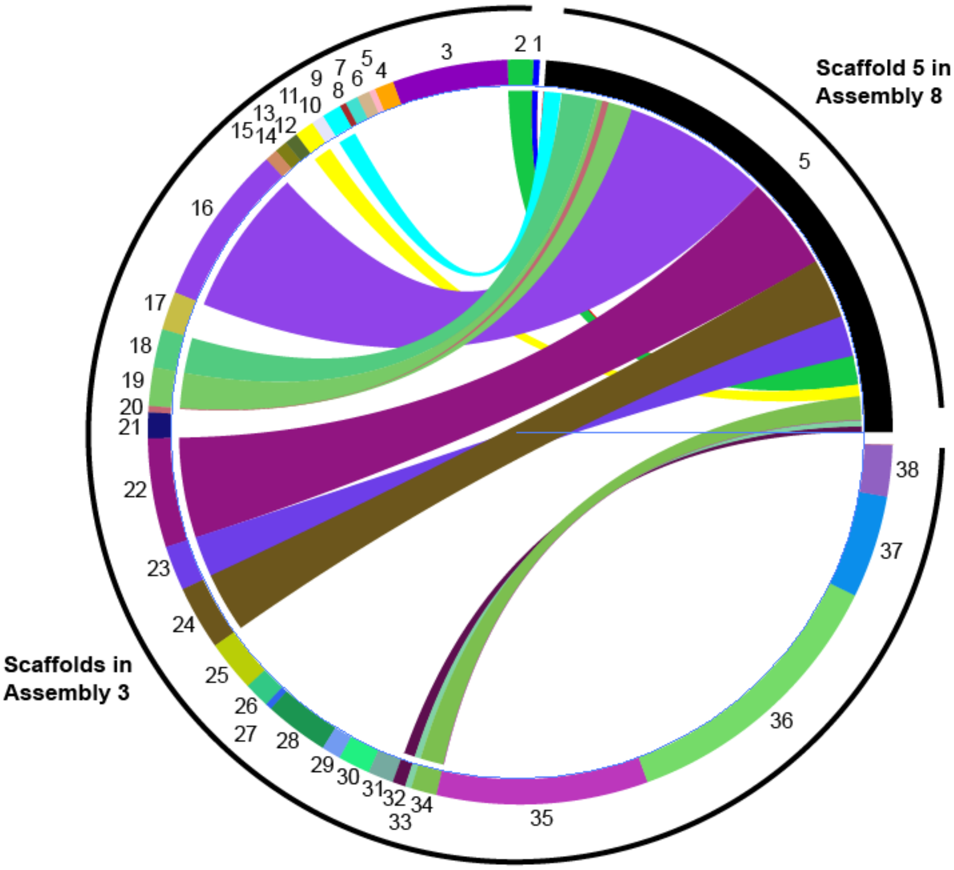

Supplementary Figure S4: Structural analysis of the possibly chimeric scaffold in Assembly 8. This figure shows the nucleotide sequence-level correspondence of the whole sequence of scaffold 5 of Assembly 8 to 14 scaffolds of Assembly 3. Note that the scaffold 5 of Assembly 8 accounts for approximately one-third of the estimated genome size, and that some of the scaffolds of Assembly 3 in the figure have multiple high-similarity regions in scaffold 5 of Assembly 8.

Assembly 3
(iconHi-C)

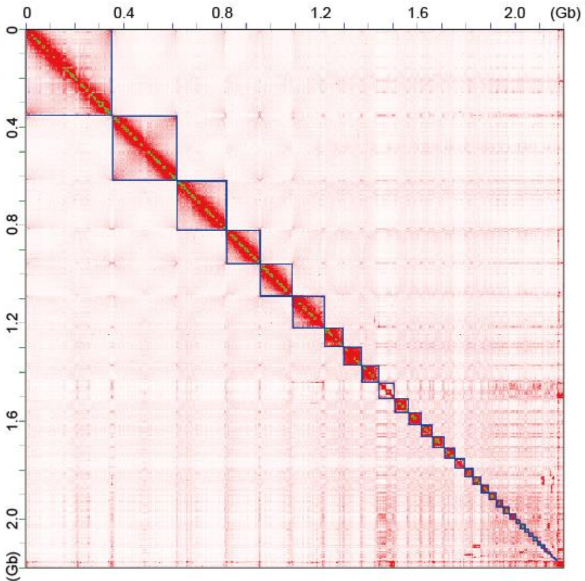

Assembly 9
(Arima)

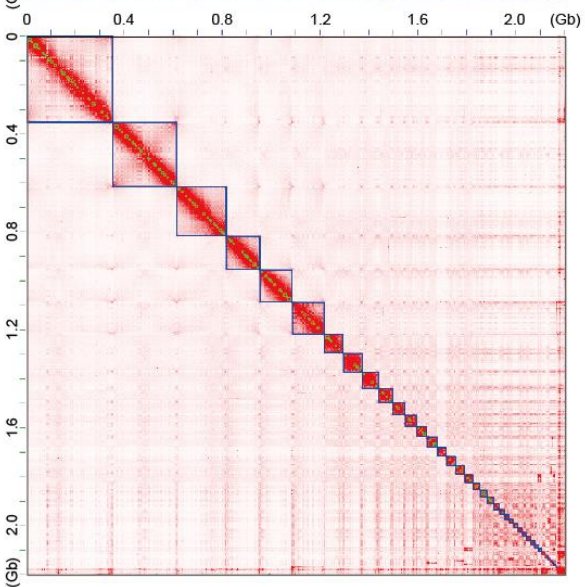

Assembly 11
(Phase)

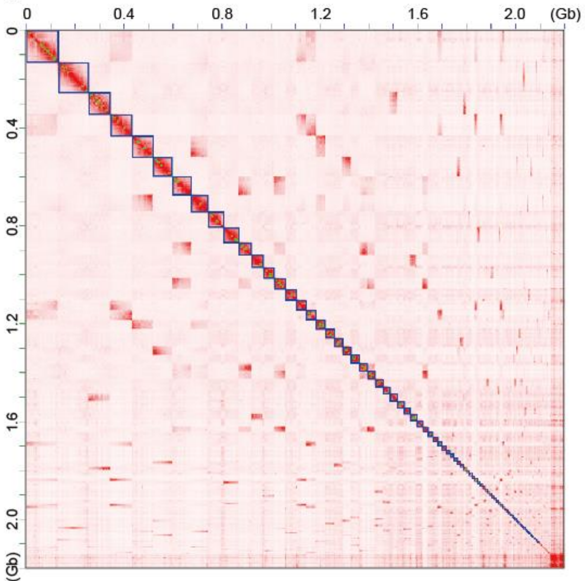

Supplementary Figure S5: Contact maps for selected softshell turtle Hi-C scaffolds. The blue squares are chromosomal units defined by 3d-dna, and the order of the scaffolds is sorted by their length. Assembly 11 exhibits the largest number of intensified blocks diverted from the diagonal line.

937

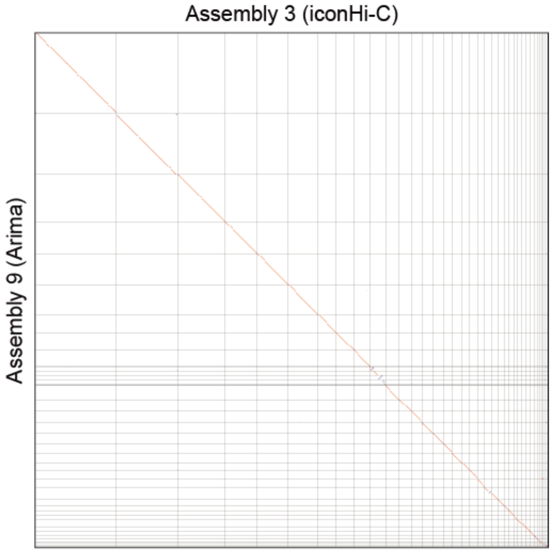
Assembly 3 (iconHi-C)
Assembly 9 (Arima)

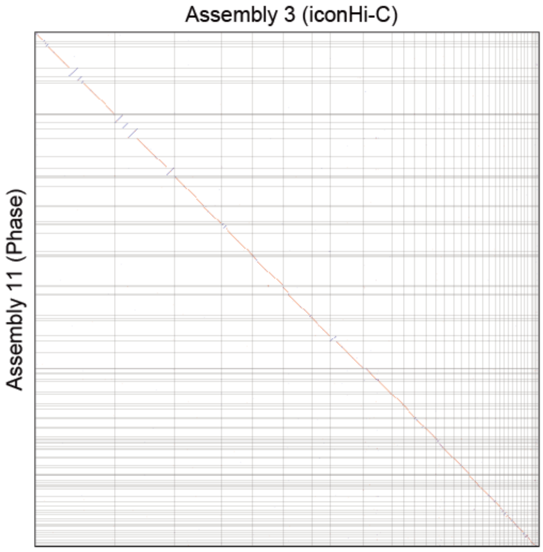
Assembly 3 (iconHi-C)
Assembly 11 (Phase)

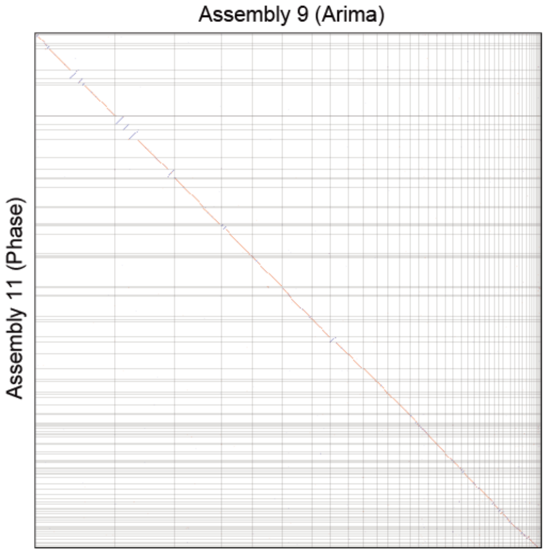
Assembly 9 (Arima)
Assembly 11 (Phase)

Supplementary Figure S6: Pairwise alignment of Hi-C scaffolds. Genome-wide alignments between the Hi-C scaffolds obtained were performed by LAST, and the dot plots were constructed using the last-dotplot script. Only scaffolds that were 1Mb or longer were included, and the order of the scaffolds along the X-axis was sorted by their length.

Response letter including the manuscript with track changes
Click here to access/download;Manuscript;Revision-report-2.pdf
Click here to view linked References

Overall report of our revision

This letter reports our revision and describes our point-by-point responses (in the indented lines) to the reviewers' comments and is followed by a manuscript text highlighting the individual changes from the initially submitted version.

Following the suggestions from two reviewers, we have had our manuscript proofread by a professional editor, which we believe has largely improved it.

Apart from our point-by-point responses to individual reviewers' comments, we have modified several parts of the manuscript as follows.

First, we have polished the accompanying protocol (**Supplementary Protocol S1**), which is also registered in Protocols.io (https://www.protocols.io/private/950FFCBDE7C46D1598CA7DDFE7441C9F). We have modified the relevant part in the **Methods** to include information about the protocol in Protocols.io.

We also realized that the literature cited for an existing Hi-C library preparation protocol (Literature # 31 in the originally submitted manuscript) was not appropriate. We have replaced this with a correct one (now cited as #35).

Our revision includes a renumbering of resultant Hi-C-based assemblies. In the originally submitted manuscript, Assembly 3 and Assembly 13 are identical to each other (but differently labelled because the latter was referred to in the comparison between different parameter settings). Also, we have tested more parameter settings with SALSA2 and thus have more assemblies, which is detailed in our response to one of the reviewers' comments. The new numbering (Assembly 1-28) is found in **Supplementary Table S6**.

Response to Reviewer #1:

General assessment

The authors have demonstrated an optimized protocol and accompanying quality control rationale for the reliable generation of good quality Hi-C sequencing libraries. To highlight the benefits of their method, a comparative analysis is employed against current commercial Hi-C library kits from the companies Phase Genomics, Arima Genomics and Dovetail Genomics.

The Hi-C protocol has proven to be a difficult sticking point for many labs, with inconsistent data quality and significant bench time being two factors which hold back a field with so much potential. Commercial kits, which have aimed to ameliorate both, have thus been quickly adopted.

> Thank you very much for your comments and your precious time to review our manuscript.

I have separately assessed the Hi-C signal content of the generated libraries and find good agreement with those described by the authors. Notably, their libraries for all protocols contain the highest percentage of Hi-C pairs that I have yet observed. The iconHi-C protocol is an important advancement in library production and I applaud the authors for making their key findings public.

Comments overall

Quality of writing: The writing quality of the manuscript is acceptable, albeit the authors may wish to involve a third-party to assist in revising the text for grammatical errors and unusual word choice.

> As suggested, we have had the revised manuscript proofread by a native English speaker, which we believe led to improvement of the manuscript.

Though difficult to furnish, a more complete ground truth (genome) would have aided this study in conclusively interpreting the scaffolding results. However, I do not propose this be carried out.

> As stated below as a response to some other reviewers' comments, we totally recognize the limitation in using the softshell turtle, while our findings provide valuable insights.

As a parametric sweep, it would be helpful if the authors provided a simple table of the parameter ranges tested, even if supplementary.

> The parameter ranges we tested in the present study are included in **Figure 9A**, which is based on the information included in the **Supplementary Table S6**.

The collection primary data-sets generated by the authors will be extremely useful for future work on Hi-C genome scaffolding and consequently so too will their 3d-dna and SALSA2 scaffolding results. In the interests of FAIR, I would strongly encourage the authors to submit all their downstream results to a public archive, such as Zenodo or Figshare.

As suggested, we have submitted the downstream results of scaffolding to the GigaDB repository.

Supplementary_Protocol_S2: this reviewer greatly appreciated the addition of patching notes for HiC-Pro, so as to support Arima's protocol design. Ideally, however, I encourage the authors to fork the HiC-Pro repository on github, make these changes and then submit a pull request back to the maintainers.

We basically agree with this suggestion, but we understand that the program to fork in this situation should not be HiC-Pro but Juicer. In fact, the developer of Juicer seems to have modified the script aidenlab/juicer/misc/generate_site_popsitions.py at GitHub last month, after we received the reviewers' comments. To avoid any redundancy and confusion, we refrain from further forking it by ourselves, and keep the way of releasing our script as it was (**Supplementary Protocol S2**).

Comments by section

In setting the stage, it would be helpful to readers if the authors made clear the motivation for why protocol optimisation should be pursued. What, if anything, is wrong with the status quo?

We first realized that the protocol by Sofueva et al. (*EMBO J*, 24:3119-29, 2013) which we thought was widely used 1) required a relatively large number of cells (namely, 10^7 cells), 2) lacked steps for systematic quality controls before sequencing, and 3) actually resulted in a suboptimal diversity of obtained Hi-C read pairs. Therefore, our original motivation was to improve these points. As suggested, we have inserted the sentence below in the Background, so that the readers can recognize these pre-existing challenges.

'Optimization of Hi-C sample preparation, however, has been limited~~ly attempted~~ [16], which leaves room for the improvement of efficiency and the reduction of required sample quantity. '

Line 134: In the sentence containing "overt differences", the description of how the authors arrived at their chosen set of parameters is extremely brief. Considering the success of their study, expanding on their observations here would be interesting.

As suggested, we have modified this sentence as below:

We identified overt differences between the sample preparation protocols of ~~already~~ published studies and those of commercial kits, especially regarding the duration of fixation and enzymatic reaction as well as the library preparation method used (Fig. 1B). Therefore, we first sought to optimize the conditions of several of these ~~preparation~~ steps using human culture cells.

In addition, we have inserted a sentence below in the figure legend to indicate the versions of the commercial kits employed in this study, although this information was already included in the **Methods**:

‘The versions of the Arima and Phase kits used in this study are presented. ’

Considering the wide range in quality of published Hi-C data-sets, the quality of Hi-C libraries in this study (regardless of protocol), which made it through to the stage of rapid-run and HiC-Pro, is extremely high. It would have been interesting to see HiC-Pro results for libraries which failed QC1 and QC2, so as to better calibrate expectations for the reader.

Unfortunately we could not afford sequencing of unsuccessfully prepared libraries. Thus, we have no such data that allow post-sequencing QC with HiC-Pro.

Did the authors take the restriction digest and ligation reactions to further timepoints? It would seem from figure 6 that neither are slowign down at their final timepoints. How have the authors convinced themselves that these edges of their parameter sweep represent optimal values?

We have not elongated these reactions further, because we thought that further elongating them decreases the overall utility of the protocol, because it becomes longer than ‘overnight’.

Although the authors have explored length cut-offs for 3d-dna down to the default of SALSA2 (1000bp), it does not seem that they've attempted the converse; namely the performance of both tools at 15000bp. There exists a large difference in statistical confidence when counting Hi-C associations (between 1k and 15k), as well as the tendancy for smaller contigs to possess confounding features such as repeats. In this way, the potential for error when scaffolding grows as the contig size decreases. Parallel to this, the criteria governing the choice of default limits are not universal between developers. Holding in mind an understanding of the error processes in their tool, one developer might select a conservative value to minimise error while others might simply chose a limit based on their experience with computational scaling.

> We agree with this suggestion, and have performed Hi-C scaffolding with the program SALSA2 with the input sequence length cutoff set at 3000, 5000, and 15000. The results have been included in the **Supplementary Table S6**. In brief, increasing the input sequence length cutoff (the '-c' option) resulted in smaller lengths of maximum scaffolds (approx. 105 Mbp compared to approx. 352 Mbp for Assembly 3, 7, and 9 that exhibited the best scores), and did not improve gene space completeness scored by BUSCO. We also tentatively increased the rounds of iterative correction (the '-i' option) to 4 or higher, which resulted in a slight increase of the N50 scaffold length while some scaffolds harbored chimeric sequences (e.g., the largest, 427 Mbp-long scaffold of Assembly 24).

Line 25: Is it true that there is a lack of published articles on library protocol development? There are definitely articles which aim to extend or modify the Hi-C protocol, but perhaps a shortage of articles which only aim to optimise the existing protocol. Work that has been done, kept behind closed doors as intellectual property.

> There are some existing efforts on Hi-C sample preparation optimization, as found in the literature cited as [16]. We meant that the existing effort is limited in terms of its application to genome scaffolding. We mention this in Introduction as below:
>
> '*Optimization of Hi-C sample preparation, however, has been limited~~ly attempted~~ [16], which leaves room for the improvement of efficiency and the reduction of required sample quantity. Thus, ~~it remains unexplored which factor in particular makes a difference in the results of Hi-C scaffolding,~~ the specific factors that are key for Hi-C scaffolding remain unexplored, mainly because of ~~its~~ the costly and resource-demanding nature of this technology.*'

Comment line 208: I do not fault the authors for restricting their focus, but the potential depth of discussion on enzyme choice is much greater than what the authors have limited themselves; DpnII, HindIII and multi-enzyme digest. For instance, there are 18 commercially supplied 4-cutters with 4nt overhangs, whose 6 distinct sites effectively cover the spectrum of GC richness. The enzymes in this larger pool will possess differences which could positively or negatively affect the Hi-C protocol. Differences such as methylation sensitivity and fidelity in non-optimal conditions. In any study such as this, some words on limitations would be informative to readers.

We basically agree with this suggestion. We already discussed the restriction enzyme choice in Discussion. To draw readers' attention to possible improvement with other enzymes, we have inserted a sentence below in the middle of the paragraph **Considerations regarding sample preparation** in the **Discussion**.

'Obviously, the use of restriction enzymes that were not employed in this study might be promising in the adaptation of the protocol to organisms with variable GC-content or methylation profiles.'

Minor comments

Line 54: The sentence might read better as "... both within and between chromosomes, ..."

We have modified the text as suggested.

Line 57: Rather than dangling ", more recently" at the end, a more active voice would perhaps be "... which has recently prompted this method to be employed ..."

We have modified this part into the form included below, as suggested by a professional proofreader.

'...which has recently prompted the use of this method in scaffolding de novo genome sequences'

Line 64: "In early 2018" could begin a new paragraph.

We have modified the text as suggested.

Line 71: "has been limited."

We have modified the text as suggested.

Line 78: Perhaps the authors meant "desirable" rather than "anticipated".

We have modified the text as suggested.

Lines 84-86: The sentence beginning with "Despite its moderate global GC-content ..." seems to be missing a final prepositional phrase. What about GC heterogeneity and chromosomal sizes was suggested by the study?

As the last part of this sentence had little to do with the main theme of the present study, we have deleted it as below.

'*Despite ~~its~~ the moderate global GC-content in its whole genome at around 44%, ~~an earlier study suggested~~ the intragenomic heterogeneity of GC-content between and within the chromosomes has been suggested [19]~~, along with their sizes~~.*'

I hope this modification solves the problem pointed out here.

Line 87: species'

We did not get the intention of this suggestion. The subject of this sentence is 'A wealth of cytogenetic efforts on this species', we believe that this part makes sense without making any change.

Line 122: Does "unusable" mean "not valid" in the eyes of HiC-Pro? I recommend that the authors avoid introducing a new term and simply replace unusable with invalid in the body of the text.

We agree with this suggestion and have replaced 'unusable' with 'invalid'.

Paragraph at 121: it may improve manuscript consistency to label the pilot-sequencing based QC step as QC3. This type of pilot-run based QC analysis is likely to become

standard procedure and see further software support. The manuscript would benefit from introducing a convenient term of reference for all three stages of QC.

> We have introduced the naming QC3 as suggested. It is introduced as included below:
>
> *'To identify such libraries, we routinely performed small-scale sequencing ~~with the purpose of~~ for quick and inexpensive QC (designated 'QC3') using the HiC-Pro program [25] (see Fig. 4 for the read pair categories assigned by HiC-Pro). Our test ~~with~~ using variable input data sizes (500 K–200 M read pairs) resulted in highly similar breakdowns into different categories of read pair properties (Supplementary Table S2) and guaranteed ~~the~~ QC3 with an extremely small data size of 1 M or fewer reads. These post-sequencing QC steps ~~that~~ ,which do not incur a large cost, are expected to help avoid large-scale sequencing of unsuccessful libraries that have somehow passed through the QC1 and QC2 steps. Importantly, libraries that have passed ~~this~~ QC3 can be further sequenced ~~in more~~ with greater depth as necessary.'*

Line 142: insert "also" and change tense: "Increased duration of cell fixation also reduced the proportion..."

> We have modified this part into the form included below, as suggested by a professional proofreader.
>
> *'The increase in the duration of cell fixation also reduced the proportion…'*

Line 170: More conventional QC language would be "passing controls" rather than being qualified by them. e.g. "All samples prepared using the iconHi-C protocol passed both controls." Stating that iconHi-C is compatible with these tests could mentioned separately.

> We have modified the text as suggested.

Line 172: Here, you could employ the name QC3 if you named the post-sequencing test as suggested above.

> We have modified the text as suggested.

Line 201: "Of those" seems unnecessary. Instead, "Assembly 8, which employed input Hi-c reads derived from both ..."

We have modified this part into the form included below, as suggested by a professional proofreader.

'Assembly 8, which resulted from input Hi-C reads derived from both…'

Line 240-241: It may be clearer to say "... or perhaps indicates an erroneous ..."

We have modified the text as suggested.

Lines 246, 251, 255: Unnecessary pluralisation "starting material"

We have modified the text as suggested.

Line 255-256: It may be better to replace "seems" with "is" and remove the comma before "to". "In preparing the starting materials, it is important to optimize the degree of cell fixation depending on your sample choice to obtain an optimal result in Hi-C scaffolding."

We have modified this part into the form included below, as suggested by a professional proofreader.

'In the preparation of the starting material, it is important to optimize the degree of cell fixation depending on sample choice, to obtain an optimal result in Hi-C scaffolding'

Line 261: It may be better to replace enhanced with increased.

We have modified the text as suggested.

Line 280: It may be better to replace "species-by-species" with "interspecies"

We have modified the text as suggested.

Line 296: insert comma "... libraries, including the one employing..."

We have modified the text as suggested.

Line 303-304: It may be clearer to say: "This procedure allowed us to minimize the PCR cycles, down to as few as five."

We have modified this part into the form included below, as suggested by a professional proofreader.

'This procedure allowed us to reduce the number of PCR cycles, down to as few as five cycles'

Line 317-319: I am not sure what is meant by "... operability of library insert lengths".

First, our expression was not clear enough. We agree with this, and have modified this part ('<u>*does not allow a flexible control of library insert lengths*</u>' included below). Because we recognized a modification in an updated protocol of the Phase Genomics Proximo Hi-C kit, we have included this information in the following sentence, for the convenience to potential users. In short, the amount of DNA used in this step is now much reduced, but the concern about bias introduced by excessive amplification remains.

'~~As for~~ <u>Regarding</u> the Phase ~~Genomics Proximo Hi-C~~ kit, transposase-based library preparation contributes largely to ~~shortening~~ its <u>shortened</u> protocol, but this <u>does not allow flexible control of library insert lengths</u>. <u>Recent protocols (versions 1.5 and 2.0) of the Phase kit instruct users to employ a largely reduced DNA amount in the tagmentation reaction, which should mitigate the difficulty in controlling insert length but require excessive PCR amplification.</u>'

Line 320: It may improve continuity to begin with "This is especially so if Hi-C ..."

According to this suggestion and professional proofreading, we have replaced this

sentence with the one below.

‘*~~Especially if Hi-C sample preparation is performed for a limited number of samples,~~ In particular, if preparing a small number of samples for Hi-C, as practiced typically for genome scaffolding, one ~~would~~ should opt to consider these points, even ~~in~~ when using commercial kits, ~~in order~~ to ~~further~~ improve the quality of the prepared libraries and scaffolding products.*’

Line 331: Support for the observation that assembly analysis outcome improves with increasing number of Hi-C pairs can be found in the article describing the metagenomic Hi-C binner bin3C.

Thank you very much for introducing literature consistent with our observation. As an additional reference, we have cited this literature in the relevant sentence as below:

‘Our comparison show~~s~~ed a dramatic decrease in assembly quality ~~when less than~~ in cases which <100 M read pairs were used (see the comparison ~~among~~ of Assembly 18–22 described above; ~~in~~ Fig. 9; also see [29]’

Reference:
‘[29] DeMaere MZ and Darling AE. bin3C: exploiting Hi-C sequencing data to accurately resolve metagenome-assembled genomes. Genome Biol. 2019;20 1:46. doi:10.1186/s13059-019-1643-1.’

Line 348: remove comma after consider "... points to consider in order to ..."

We have modified this part into the form included below, as suggested by a professional proofreader.

‘Apart from the choice of program, several points should be considered if successful scaffolding for a smaller investment is to be achieved.’

Line 351: remove comma after maps "... to contact maps using an interactive ..."

We have modified the text as suggested.

Line 365-367: The sentence about cut-off length beginning with "One needs..." is unclear. This may simply be word choice.

We have modified this sentence to enhance the clarity.

'*The deliberate setting of a cut-off length is recommended if particular sequences with relatively small lengths are the target of scaffolding.* ~~*One needs to deliberately set the length cutoff in accordance with the overall continuity of the input assembly and possible interest into particular, fragmentary sequences expected to be elongated.*~~'

Line 500-503: Recommend splitting this sentence in two and revising. "The restriction fragment..."

As suggested, we have split this sentence into two, which are included below:

'*The restriction fragment file required by Juicer was prepared by the script 'generate_site_positions.py' script of Juicer. By converting the restriction fragment file of HiC-Pro to the Juicer format, an original script that was compatible with multiple restriction enzymes was prepared (Supplementary Protocol S2).*'

Comment: Employing downstream tools such as Juicebox and taking these assemblies as different starting points, it would be interesting to see how many hand-optimisation steps were required before achieving diminishing returns and how close to optimal was each final solution. This may require a more complete ground truth than to what the authors have access.

As we already expressed as responses to other comments, we understand that our present study cannot encompass a full benchmarking by referring to the 'answer' of chromosome-scale genome sequences. As pointed out here, usually, a raw output of Hi-C scaffolding is manually optimized, so the amount of effort for these manual steps can also make a huge difference in the final output. In our present study, we do not intend to evaluate those manual steps for finalization and focus on sample preparation and the product of reproducible computational steps, namely raw Hi-C scaffolds (before final manual optimization). We understand that those computational steps make a fundamental difference in the outputs that cannot easily be recovered later by manual modification, and that quality assessment of the steps until that point can provide valuable methodological insights.

Figure 9: Condensing panels B, C and D into a single frame or adding grid lines would make it much easier to make comparative observations between the various assemblies. As well, carrying over the groupings from panel A onto the other panels. I accept that these layout operations may be difficult to achieve.

> We have moved the original panel **B** to the rightmost slot in **Figure 9**, so that a large blank space in the original panel **B** does not interfere. Accordingly, the original panels **C** and **D** have been relabelled to be **B** and **C**, respectively. We hope the visibility of the figure has somewhat increased.

Response to Reviewer #2:

> Thank you very much for your precious time dedicated to reviewing our manuscript.

Summary: In this manuscript, Kadota et al. present the results of a comparison of several kit-based methods for Hi-C library prep against a composite method they have developed called, iconHi-C. They test parameters related to library construction, RE digestion and even scaffolding software with the goal of identifying the best parameters for Hi-C scaffolding. Unfortunately, I do not think that their tests are always appropriate, and I worry that their use of extended duration ligation and restriction digestion adds more bias into Hi-C library preparation. My comments follow in the order in which I encountered an issue in the manuscript:

There appear to be many grammar and terminology errors in the submitted manuscript. As currently written, it would require professional English language editing to improve the text.

> As suggested, we have had the revised manuscript proofread by a native English speaker, which we believe led to improvement of the manuscript.

As an example of the problem, I have identified the following grammar/terminology errors in the abstract alone:

Line 20: This sentence contains a redundant predicate: "a derivative of chromosome conformation capture" was, "originally developed as a means for characterizing

chromosome conformation." I think that the authors should instead reformat the predicate of the sentence to refer to the fact that Hi-C is a "whole-genome" method -- in contrast to 3C -- and abbreviate the sentence from there.

Thank you very much for pointing that out. We have modified the text as below:

‘Hi-C is derived~~, a derivative of~~ from chromosome conformation capture (3C) ~~targeting~~ and targets chromatin contacts on a genomic scale~~the whole genome, was originally developed as a means for characterizing chromatin conformation~~.’

Line 23: Hi-C data is used for "scaffolding." It does not "elongate" nucleotide sequences.

We have replaced the word ‘elongation/elongated’ with ‘scaffolding/scaffolded’.

Line 25: Replace "the prevailing and irreplaceable use" with "Despite its prevalent use"

We have modified the text as suggested.

Line 38: Replace "and release the resultant" with "and demonstrate this technique on a" And there are many more scattered throughout the rest of the manuscript.

We have modified the text as suggested.

Line 38: The authors did not "assemble" the Chinese softshell turtle but used existing contigs from the previously released assembly in scaffolding. The difference is slight but important: I expected to see new de novo contigs for this species in this manuscript because of this statement.

To avoid any misunderstanding, we have replaced the word ‘assembly’ with ‘sequences’.

Fig 1: There are some misleading statistics in the figure. Firstly, Phase Genomics has several different kits for Hi-C preparation, and some of these kits (specifically the "Microbe kit") contain additional RE enzymes such as MluCI. I understand that the authors list the "animal versions" where applicable, but isn't this cherry-picking?

Furthermore, RE enzyme digestion is likely dependent on RE motif prevalence in the target organism. Finally, what do the authors define as the "Hi-C reaction" row specification? Is this the required, post-fixation DNA concentration?

> For the Phase Genomics kit, we included in **Figure 1B** that we used the ‘Animal’ kit. For the readers’ convenience, we also included this in the **Methods** and also modified this part according to the edited manuscript by professional proofreading as below.
>
> *‘The Proximo Hi-C kit (Phase Genomics) which employs the restriction enzyme Sau3A1 and transposase-based library preparation [36] (Fig. 1B) was used to prepare ~~for preparing~~ a library from ~~the~~ 50 mg of the softshell turtle liver ~~following its~~ according to the official ver. 1.0 animal protocol provided by the manufacturer (Library g in Fig. 7A) and ......’*
>
> Regarding the species-specific factor of the restriction enzyme recognition sites in a genome, we included the sentences below in **Discussion**, which has been a bit more elaborated following one of the comments from **Reviewer #1**:
>
> *‘The ~~G~~genomic regions that are targeted by Hi-C are determined by the choice of restriction enzymes. Theoretically, 4-base cutters (e.g., DpnII), which potentially ~~with~~ have more frequent restriction sites on the genome, are expected to provide a higher resolution than 6-base cutters (e.g., HindIII) [16]. Obviously, the use of restriction enzymes that were not employed in this study might be promising in the adaptation of the protocol to organisms with variable GC-content or methylation profiles. However, ~~it~~ this might not be so straightforward when considering the interspecies variation ~~of~~ in GC-content~~, as well as its~~ and the intra-genomic heterogeneity~~, are taken into consideration~~.’*
>
> Regarding the word ‘Hi-C reaction’, we have replaced it with ‘restriction digestion and ligation’.

Line 67: While Bickhart et al. 2017 was one of the first demonstrated uses of LACHESIS, this was not the publication that described the method. Burton et al. 2013 should be cited here.

> Thank you for pointing this out. We have replaced the citation as suggested. Also, we have cited two more publications reporting scaffolding programs introduced in an earlier period: dnaTri and GRAAL.

In the **Background**:

'Analyses of chromatin conformation using Hi-C have revealed more frequent contacts between more closely linked genomic regions, which has recently prompted the use of this method in scaffolding de novo genome sequences [4-6].'

In the **References**:

4. Burton JN, Adey A, Patwardhan RP, Qiu R, Kitzman JO and Shendure J. Chromosome-scale scaffolding of de novo genome assemblies based on chromatin interactions. Nat Biotechnol. 2013;31 12:1119-25. doi:10.1038/nbt.2727.

5. Marie-Nelly H, Marbouty M, Cournac A, Flot JF, Liti G, Parodi DP, et al. High-quality genome (re)assembly using chromosomal contact data. Nat Commun. 2014;5 1:5695. doi:10.1038/ncomms6695.

6. Kaplan N and Dekker J. High-throughput genome scaffolding from in vivo DNA interaction frequency. Nat Biotechnol. 2013;31 12:1143-7. doi:10.1038/nbt.2768.

Line 111: I do not understand the sentence as written. What does, "exhibit a slight length recovery of restricted DNA fragments," mean? Did the authors mean that post-fixation, post-digested DNA should have a higher observed molecular weight on a gel?

We have replaced the word 'recovery' with 'increase' and further modified this part according to a suggestion from a professional proofreader, which now reads '... a slight increase in the length ...'.

Line 114: The difference in shift is quite small -- did the authors calculate an average or variance in shift that can be used to assess the quality of the preparation in a quantitative manner? The authors mention that they used an Agilent Tapestation, so these metrics should be available to them.

The 'quite small' difference this reviewer referred to is not that small - the scale in basepairs on the left should serve as a guide. The peak lengths of the 'digested' and 'Hi-C DNA' samples of sample 1 in **Figure 3B** are 12,744 bp and 18,077 bp, respectively.

Line 117: Again, what is the size of the "shift" of gel electrophoresis products here? Can this be identified and used as a quantitative indicator of library quality rather than a qualitative indicator?

The peaks of the DNA size distribution before and after the NheI digestion for Sample 1 in **Figure 3C** were 483bp and 313bp (and the average lengths, 512bp and 349bp), respectively.

We cannot regard this as a quantitative indicator. We trust this indicator but it allows only a judgement based on relative shifts of length distributions within a sample, and no consistent criterion has been drawn from comparisons between different samples or preparation conditions. In fact, this apparently belongs to a list of future tasks. Thank you very much for your constructive suggestion.

Line 139: Here is where a quantitative metric would help. The fragment distributions in the 10- and 30-minute fixation samples appear to be different. The 30-minute fraction appears to be universally higher. Isn't this significant enough to even be a qualitative indicator of differences in the prep?

Our response included above may apply to this point, as well. We certainly see such a tendency in **Figure 5A** and **5B**, but we have neither accumulated experience to find a reliable criterion for evaluating the effect of variable fixation durations nor think that smaller DNA lengths in between-sample comparisons always indicate success of library preparation.

Line 148: I am concerned with this interpretation of the data here. First, prolonged RE digests can exhibit star activity. Second, prolonged ligation can increase the proportion of chimeric fragments. Both enzymatic activities have measured rates of activity (typically stated in "units") that can be customized based on measured inputs to the reaction. Did the authors estimate the molarity of DNA for the ligation reactions or estimate the amount of time for DNA digestion based on the units of RE enzyme added? Finally, the authors claim that the last timepoint is the best in all cases -- was data collected for a 24-hour timepoint or an 8-hour timepoint for the digest and ligation, respectively?

We were of course aware of a possible adverse effect of prolonged restriction. For this reason, for DpnII digestion, we avoided using NEBuffer 3.1 that is said to cause star activity and instead used NEBuffer DpnII. Also, for HindIII digestion, we used HindIII-HF (high-fidelity). In the revision, we have taken your comment seriously and have performed library preparation and small-scale sequencing to be confident of the absence of the adverse effects. In brief, the proportion of the fragments derived from proper restriction and ligation remained unchanged even

with elongation of reaction duration, which rules out the possible effect of star activity. The details of this new data have been included at the end of the section titled ‘Optimization of sample preparation conditions’ in **Results** as below, and the actual data are presented in **Supplementary Table S4**.

‘To scrutinize further the possible adverse effects of the prolonged reaction, Hi-C libraries of GM12878 cells were prepared with variable durations of restriction digestion (1 hour and 16 hours) and ligation (15 minutes, 1 hour, and 6 hours). We found that the proportions of dangling end and religation read pairs were reduced in cases with an extended duration of restriction digestion (Supplementary Table S4). The yield of the library, which can be estimated from the number of PCR cycles, increased with the extended duration of ligation without any effect on the proportion of valid interaction read pairs (Supplementary Table S4).’

We understand the importance of estimating the optimal enzyme units to digest particular amount of DNA molecules. However, the restriction reaction in Hi-C sample preparation targets DNA in the cell nuclei, and thus it is not realistic to identify the optimal enzyme unit per DNA amount that applies to various samples. For these reasons, in our iconHi-C and other protocols (Sofueva et al., 2013; Hi-C2.0, etc), the amount of restriction enzymes are thought to exceed the optimal amount for individual samples.

In the section ‘Availability of supporting data’, we have inserted an additional DDBJ DRA accession ID for the new sequencing data with varying restriction and ligation reaction durations.

Regarding the duration of restriction enzyme digestion and ligation, we do not claim that the longest in our series (16 hr for restriction and 6 hr for ligation) is the best. As included in the response to one of the comments to **Reviewer #1**, we have not tested further elongated reaction times. It is because further elongating them decreases the overall utility of the protocol, as it becomes longer than an ‘overnight’.

Line 152: So the optimization was based on gel shift data? What was the goal of this optimization? I think that the authors may have simply optimized the shift of sample on the gel here. A sufficient test of optimization would involve the use of several different timepoints for each enzymatic prep in separate Hi-C libraries, and then using the data derived from these libraries in scaffolding.

Cost for large-scale sequencing of a series of Hi-C libraries with variable enzymatic

reaction durations would not be trivial. We fully understand its importance but were unfortunately limited by the budget. Instead, we have performed QC3 (evaluation by HiC-Pro after small-sequencing) of Hi-C libraries prepared with different timepoints. The details have been included above in our response to your comment (regarding Line 148).

Figure 7: Why was the blood sample not used with other kits? Why include it in the comparisons?

It would have been ideal if our comparison was more thorough, but honestly, we were limited with the budget for purchasing the kits. Our comparison between the liver and blood with the iconHi-C protocol showed a better performance with the liver. Thus, we adopted the liver for a comparison between the iconHi-C protocol and the commercial kits.

Line 171: Aren't you only showing the QC1 and QC2 results for iconHi-C in this figure? Also, the authors do not label their alignment-based quality control (via HiC-Pro) as a separate form of QC (e.g. QC3). This becomes confusing later in the paragraph, where the blank "QC" term is used indiscriminantly.

Performing the quality controls equivalent to QC1 and QC2 are not always feasible with commercial kits. For example, QC2 is not feasible with Arima Genomics Kit, because it employs two restriction enzymes. Also, because we simply followed the manufacturers' protocols, we did not perform QC1 for both Arima Genomics and Phase Genomics kits, as the protocols did not instruct so. With the Phase Genomics kit, we performed QC2 for the libraries described in the present manuscript, which has been included in **Supplementary Fig. S3**. This figure has been cited in the legend of **Fig. 7**.

'(C) Quality control of Hi-C libraries (QC2). The ~~prepared softshell turtle liver~~ HindIII library prepared from the softshell turtle liver was digested by NheI, and the DpnII library was digested by ClaI (see Fig. 3 for the technical principle). See Supplementary Fig. S2 for the QC1 and QC2 results for the samples prepared from the blood of this species. See Supplementary Fig. S3 for the QC2 result of the Phase libraries.'

Regarding the labelling of the post-sequencing quality control with HiC-Pro, we have designated it 'QC3' consistently in the revised manuscript.

Line 218: What about short-read WGS alignment comparisons using FRC_align or comparisons with a third technology such as an optical map? I find that the use of the positions of 162 marker genes may be too small to identify fine-scale errors in scaffolding smaller contigs which is a known problem in Hi-C scaffolding (Bickhart et al. 2017). Additionally, assembly-to-assembly alignments and comparisons of WGS read-mapping profiles across these regions could be used to assess quality.

As suggested, we used FRC_align to evaluate the Hi-C scaffolds we obtained. We have tentatively compared Assembly 3, 9 and 11, using publicly available raw reads derived from a paired-end (insert size = 170bp) and a mate-pair (mate distance = 10Kbp) libraries (NCBI SRA IDs: SRA424857) from the original pre-HiC genome assembly published earlier (Wang et al., *Nat. Genet*. 2013). However, this has resulted in highly similar plots to each other, which we understand did not provide a suitable metric in evaluating long-range continuity (see **Supporting Figure A** below).

Supporting Figure A

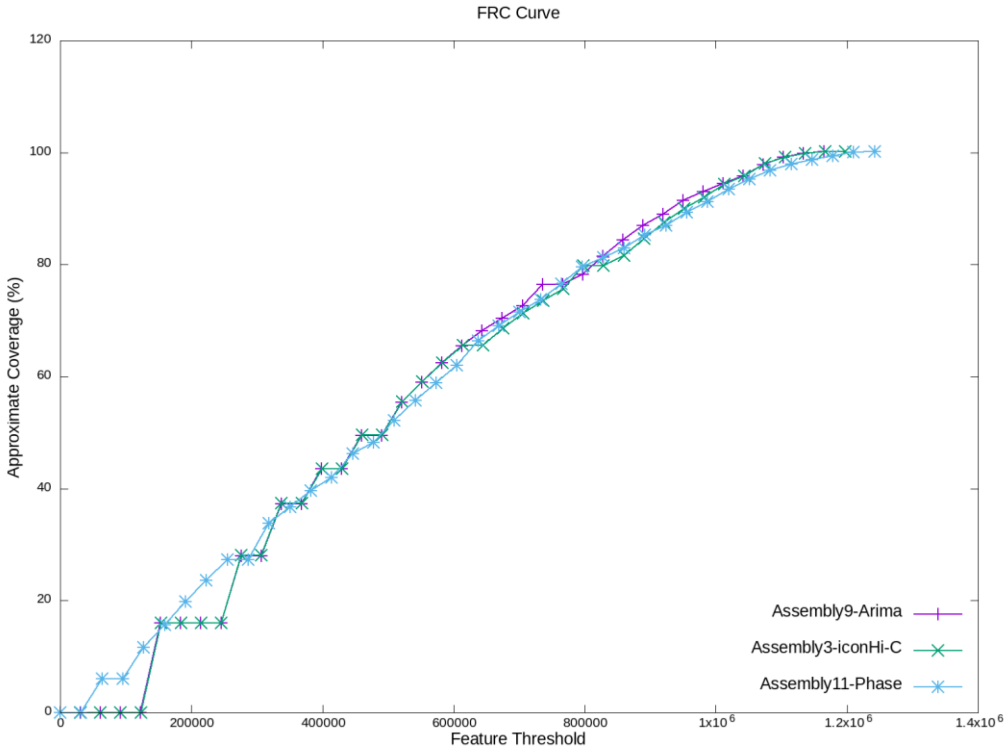

Further following this reviewer's suggestion, we performed assembly-to-assembly alignments between these selected Hi-C scaffolding results using LAST, which

exhibits few visible discrepancies between Assembly 3 and 9, while the comparison between Assembly 3 and 11 (also, the comparison between Assembly 9 and 11) revealed some obvious differences, more likely resulted from fragmentations in Assembly 11. We have included these dot matrix figures in **Supplementary Figure S6**, and cited this figure in Results as below.

‘We also performed genome-wide alignments between the Hi-C scaffolds obtained. The comparison of Assembly 3, 9, and 11 revealed a high similarity between Assembly 3 and 9, while Assembly 11 exhibited a significantly larger number of inconsistencies against either of the other two assemblies (Supplementary Fig. S6). These observations are consistent with the evaluation based on sequence length and gene space completeness, which alone does not, however, provide a reliable metric for the assessment of the quality of scaffolding.’

Line 260: Not "overassembly" but "chimeric scaffolding." This is a major issue with Hi-C that was not adequately measured by the authors in their quality control assessments. In fact, it is difficult to tell the overall "correctness" of scaffolding in each assembly apart from the BUSCO scores and scaffold N50 lengths provided by the authors -- each of which were not very informative by their own admission. More substantial scaffold quality assessment is needed.

We have replaced the word ‘overassembly’ with ‘chimeric scaffolding’. We totally agree that BUSCO or scaffold N50 lengths cannot provide a reliable metric for correctness of Hi-C scaffolds that are highly continuous and mention the saturation of scores in the beginning of the last section in the **Discussion**. To further evaluate the scaffolding results, we have compared the obtained Hi-C scaffolds with the existing report of gene mapping by FISH (**Figure 10**). Moreover, to allow visual assessment of overall consistency, we have included 3D contact maps for selected Hi-C scaffolding results (Assembly 3, 9, and 11) in **Supplementary Fig. S5** and mention this figure in the **Results** as below.

‘To gain additional insight regarding the evaluation of the scaffolding results, we assessed the contact maps constructed upon the Hi-C scaffolds (Supplementary Fig. S5). The comparison of Assembly 3, 9 and 11, which represent the three different preparation methods, revealed anomalous patterns, particularly for Assembly 11, with intensive contact signals separated from the diagonal line that indicate the presence of errors in the scaffolds [15]. ’

We also performed genome-wide alignment between the obtained Hi-C scaffolds. Again in the comparison between Assembly 3, 9 and 11, we observed high similarity between Assembly 3 and 9, while Assembly 11 exhibited significantly

larger number of inconsistencies against either assembly (**Supplementary Fig. S6**). These observations are consistent with the evaluation based on sequence length and gene space completeness, which does not, however, alone provide a reliable metric for quality assessment of scaffolding.

Line 296: The authors refer to the Arima Hi-C assembly by number, but do not refer to the "library d" assembly by number. This is confusing to the reader.

Thank you very much for pointing this out. To be consistent, we have replaced this ‘Library d’ with ‘Assembly 3’.

Line 297: This could be a concern, but it is not addressed in the results by the authors. What noticeable effects on scaffold quality were determined by PCR over-amplification?

For the Phase Genomics Proximo Hi-C kit, we have compared the HiC-Pro results between Library g (15 cycles) and h (11 cycles), which showed a remarkable difference especially in the proportion of valid interactions after deduplication. These data are presented in **Supplementary Table S8** that has been newly prepared, and we have cited this table in the relevant part in **Discussion** as below

‘~~One~~ Overamplification by PCR is a concern ~~about~~ regarding the use of commercial kits (with the exception of the Arima ~~Hi-C~~ kit used with the Arima-QC2) ~~is overamplification by PCR, as~~ because their manuals specify the use of a certain number~~s~~ of PCR cycles a priori (15 cycles for the Phase ~~Genomics Proximo Hi-C~~ kit and 11 cycles for the Dovetail Hi-C kit) (Supplementary Table S8).’

Line 315: I disagree with this interpretation. Figure 8 shows that the Arima kit had ~10% higher unique paired alignments than any of the iconHi-C preps. Was this discrepancy due to over-digestion and over-ligation in the iconHi-C protocol?

As included above in our response to your comment regarding Line 148 (of the originally submitted manuscript), we investigated the possibility of ‘over-digestion’ and ‘over-ligation’ and confirmed that our data are free from such adverse effect of over-digestion and over-ligation.

Line 333: While downsampling reads is a useful and novel comparison, did the authors

consider that the same results could apply to the libraries obtained from the other kits?

> We have confirmed that the same results apply to Arima and Phase kits. In fact, library quality assessment with small-scale sequencing (now designated 'QC3' in our manuscript) have been revealed to be effective for these kits. We have included the HiC-Pro results for Library e (Arima) and h (Phase) in **Supplementary Table S9**.

Line 397: While agree with this conclusion, this study did not adequately measure erroneous scaffolds.

> To a similar comment from **Reviewer #3**, we respond as below:
>
> As no reliable genome assembly exists for the softshell turtle, we need to admit that our evaluation for correctness is limited. To provide another self-contained metric for correctness, we present a comparison of contact matrices for three selected Hi-C scaffolding results in **Supplementary Fig. S5** of the revised manuscript, as suggested.

Line 399: I would recommend removing this entire paragraph as it does not add value to the manuscript. So long as gap regions are set to a fixed size (in the case of unknown gaps) the size of the gap sequence is irrelevant to downstream applications.

> In fact, the size of the gaps influences the evaluation of a total size of genome scaffolds, as well as the sensitivity in gene prediction in which the sizes of introns and intergenic sequences often need to be optimized. We understand that inserting gaps of unknown sizes evokes a new challenge in high-quality, chromosome-scale genome sequencing, although I agree that this is not a major issue. For this reason, we would like to keep this topic as it is.

Response to Reviewer #3:

I thoroughly enjoyed reading the manuscript benchmarking HiC data for assembly through different aspects. To my knowledge, this is the first study that comprehensively studies this topic. This is a novel study and I think the topic of the manuscript will receive tremendous interest. However, I have some queries/concerns that I would like authors to address.

Thank you very much for your positive review and constructive suggestions.

- I see in Supplementary Table S2 the percentage of long and short range read pairs. However less than 20 kbp and greater than 20 kbp is not very informative. Can you stratify more? Like percentage of read pairs between 10k -100k, 100k -1Mbp, 1Mbp-10Mbp, and 10 Mbp and above. This would highlight in what range the utility of iconHi-C protocol.

To highlight any possible range bias with iconHi-C protocol, we presented **Fig. 5D** in the originally submitted manuscript, which shows no marked range-dependent bias in *cis* interactions. Stratifying the HiC-Pro results more can be applied to **Supplementary Table S2** and **S9**, but we understand that it can help the interpretation of **Supplementary Table S4** the most. Thus, we have modified **Supplementary Table S4**, and in addition, inserted below the modified version of **Supplementary Table S2**, as well.

Supplementary Table S2: HiC-Pro results of the human GM12878 HindIII Hi-C library with reduced reads

A. Read alignment category

	Proportion of reads						
Number of input read pairs	500 K	1 M	5 M	10 M	50 M	100 M	200 M
Unique paired alignments	71.0%	71.0%	70.9%	71.0%	71.0%	71.0%	71.0%
Unmapped pairs	3.2%	3.2%	3.2%	3.2%	3.2%	3.2%	3.2%
Low quality pairs	0.0%	0.0%	0.0%	0.0%	0.0%	0.0%	0.0%
Multiple pairs alignments	15.3%	15.3%	15.3%	15.3%	15.3%	15.3%	15.3%
Pairs with singleton	10.5%	10.5%	10.5%	10.5%	10.5%	10.5%	10.5%
Low quality singleton	0.0%	0.0%	0.0%	0.0%	0.0%	0.0%	0.0%
Unique singleton alignments	0.0%	0.0%	0.0%	0.0%	0.0%	0.0%	0.0%
Multiple singleton alignments	0.0%	0.0%	0.0%	0.0%	0.0%	0.0%	0.0%
Reported pairs	71.0%	71.0%	70.9%	71.0%	71.0%	71.0%	71.0%

B. Read pair category

	Proportion of read pairs						
Number of input read pairs	500 K	1 M	5 M	10 M	50 M	100 M	200 M
Valid interaction pairs	65.1%	65.1%	65.1%	65.1%	65.1%	65.1%	65.1%
Valid interaction pairs (forward-forward)	16.2%	16.2%	16.2%	16.2%	16.2%	16.2%	16.2%
Valid interaction pairs (reverse-reverse)	16.1%	16.2%	16.2%	16.2%	16.2%	16.2%	16.2%
Valid interaction pairs (reverse-forward)	15.8%	15.8%	15.7%	15.7%	15.7%	15.7%	15.7%
Valid interaction pairs (forward-reverse)	17.0%	17.0%	16.9%	16.9%	16.9%	16.9%	16.9%
Dangling end pairs	2.8%	2.9%	2.9%	2.9%	2.9%	2.9%	2.9%
Religation pairs	2.6%	2.5%	2.6%	2.6%	2.6%	2.6%	2.6%
Self circle pairs	0.5%	0.4%	0.4%	0.4%	0.4%	0.4%	0.4%
Single-end pairs	0.0%	0.0%	0.0%	0.0%	0.0%	0.0%	0.0%
Filtered pairs	0.0%	0.0%	0.0%	0.0%	0.0%	0.0%	0.0%
Dumped pairs	0.0%	0.0%	0.0%	0.0%	0.0%	0.0%	0.0%

C. Duplicates and contact ranges

	Proportion of read pairs						
Number of input read pairs	500 K	1 M	5 M	10 M	50 M	100 M	200 M
Valid interaction	65.1%	65.1%	65.1%	65.1%	65.1%	65.1%	65.1%
Valid interaction (remove duplicates)	65.1%	65.0%	64.8%	64.5%	62.3%	59.8%	55.2%
Trans interaction	12.0%	12.0%	12.0%	11.9%	11.5%	11.1%	10.2%
Cis interaction (total)	53.1%	53.1%	52.8%	52.6%	50.8%	48.7%	45.0%
(<10Kb)	4.1%	4.1%	4.1%	4.1%	3.9%	3.8%	3.5%
(10K-100Kb)	11.8%	11.8%	11.7%	11.7%	11.3%	10.8%	10.0%
(100K-1Mb)	16.7%	16.6%	16.5%	16.5%	15.9%	15.2%	14.1%
(1Mb-10Mb)	10.2%	10.2%	10.2%	10.1%	9.8%	9.4%	8.7%
(>10Mb)	10.4%	10.4%	10.3%	10.3%	9.9%	9.5%	8.8%

- I understand from Figure 9 the bulk assembly contiguity statistics. However, it doesn't tell much about how correct is the assembly. I would like to see a contact matrix for a couple of assemblies that authors think are the best.

Thank you very much for your insightful comment. As no reliable genome assembly exists for the softshell turtle, we need to admit that our evaluation for correctness is limited. To give self-contained metric for correctness, we have presented a comparison of contact matrices for three selected Hi-C scaffolding results in **Supplementary Fig. S5** of the revised manuscript, as suggested.

Also, a heatmap for iconHi-C assembly constructed using other Hi-C datasets is also interesting to see. Such a comparison would highlight the valuable contact information that's probably missed in iconHi-C or other Hi-C datasets.

Thank you very much for your suggestion. We have constructed a contact map in which the Hi-C reads produced with the Arima kit and those with Phase kit (Library e and h, respectively) have been mapped onto the Hi-C scaffolds produced with the iconHi-C protocol (Assembly 3). In this contact map (**Supporting Figure B**), we still have observed a high integrity of chromosomal blocks and high similarities between the Hi-C read sets derived from different methods. Because these data do not add a lot to our findings, we keep them within this letter.

Supporting Figure B

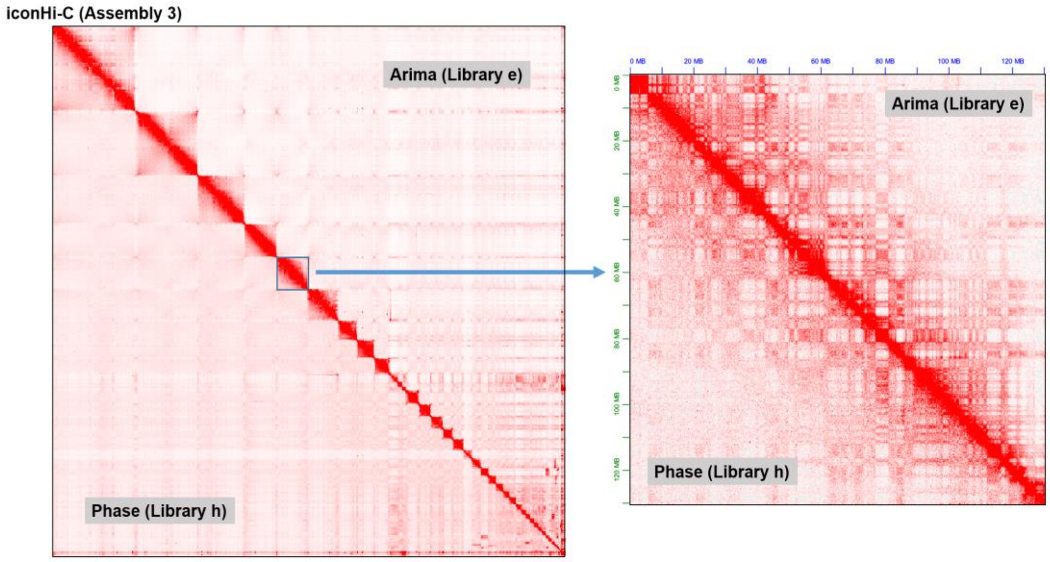

- I saw the library QC results for GM12878, however I was not able to see any scaffolding results for it with different Hi-C datasets. Since we have a known reference genome, we can get a solid evidence that which parameter setting and what type of Hi-C library provides the best assembly in terms of both contiguity and accuracy.

We totally understand your curiosity. We first set out with this project to improve the softshell turtle genome sequences, and could not invest a lot for human Hi-C libraries. Although our evaluation of the correctness of Hi-C scaffolds is limited, we have wanted to provide a model of best practice in the absence of reference genome sequences. Our results are supported by FISH-based gene mapping (**Fig. 10**) and contact maps that has been included as **Supplementary Fig. S5**.

- This may be out of the scope of this manuscript. Did authors find out minimum amount Hi-C read pairs required for good scaffolding? Such a discussion or recommendation would guide the amount of sequencing needed for the scaffolding project and would reduce the cost.

This topic was covered in **Discussion** (already included in the originally submitted manuscript), which has been slightly modified according to the edited manuscript by professional proofreading, as included below.

'Our comparison show~~s~~ed a dramatic decrease in assembly quality ~~when less than~~ in cases in which <100 M read pairs were used (see the comparison of ~~among~~ Assembly 18-22 ~~19–23~~ described above; ~~in~~ Fig. 9; also see [29]). ~~Still~~ Nevertheless, we obtained optimal results with a smaller number of reads (ca. 160 M per 2.2 Gb of genome) than that recommended by the manufacturers of commercial kits (e.g., 100 M per 1 Gb of genome for the Dovetail Hi-C kit and 200 M per Gb of genome for the Arima ~~Hi-C~~ kit). As generally and repeatedly discussed, the proportion of informative reads and their diversity, rather than just the overall number of ~~all~~ obtained reads, ~~are~~ is critical.'

- The scope of the manuscript is mainly understanding the effect of different parameters on scaffolding. But, do authors have any intuition about usage of iconHi-C in other 3D genomic application such as detecting TADs, chromatin loops, etc? Some discussion would be helpful.

We are conducting a separate 3D genome-focused analysis using the Hi-C data

produced by the iconHi-C protocol, which will be published independently from our present study. In fact, we are realizing that good Hi-C data in genome scaffolding tend to perform well with 3D genome studies.

- Figure 8 and Figure 9 is kind of hard to understand. I would appreciate if the data is displayed in a tabular format.

We understand that it is preferable to expose the whole data. For this purpose, we present raw statistics in tables - **Supplementary Table S5** for **Figure 8** and **Supplementary Table S6** for **Figure 9**. In addition, we have modified **Figure 9** (relocated B, C and D) for better visibility, responding to the very last comment from **Reviewer #1**. In each of the figures, we guide readers to these supplementary tables, in their legends.

In the legend of Figure 8:
'See Fig. 7A for the preparation conditions of Library a-h, Fig. 4 for the categorization, and Supplementary Table S5 for the actual proportion of the reads in each category.'

In the legend of Figure 9 B-D:
'See the panel A for Library IDs and Supplementary Table S6 for raw values of the metrics shown in B–D.'

Multifaceted Hi-C benchmarking: what makes a difference in chromosome-scale genome scaffolding?

Mitsutaka Kadota[1*], Osamu Nishimura[1*], Hisashi Miura[2], Kaori Tanaka[1,3], Ichiro Hiratani[2], and Shigehiro Kuraku[1]

[1] Laboratory for Phyloinformatics, RIKEN Center for Biosystems Dynamics Research (BDR), Kobe, 650-0047, Japan, [2] Laboratory for Developmental Epigenetics, RIKEN BDR, Kobe, 650-0047, Japan, [3] Present address: Division of Transcriptomics, Medical Institute of Bioregulation, Kyushu University, Fukuoka, 812-0054, Japan

*These authors contributed equally to this study.

Correspondence address. Shigehiro Kuraku, Laboratory for Phyloinformatics, RIKEN BDR, Japan. Tel: +81 78 306 3048; Fax: +81 78 306 3048; E-mail: shigehiro.kuraku@riken.jp

Abstract

Background: Hi-C~~, a derivative of~~ is derived from chromosome conformation capture (3C) ~~targeting the whole genome, was originally developed as a means for characterizing~~and targets chromatin ~~conformation. More recently, this~~contacts on a genomic scale. This method has also been used frequently ~~employed~~ in ~~elongating~~scaffolding nucleotide sequences obtained by *de novo* genome sequencing and assembly, in which the number of resultant sequences rarely ~~converge into~~converges to the chromosome number. Despite ~~the prevailing and irreplaceable~~its prevalent use, the sample preparation methods for Hi-C have not been intensively discussed, especially from the standpoint of genome scaffolding.

Results: To gain ~~insights~~insight into the best practice of Hi-C scaffolding, we performed a multifaceted methodological comparison using vertebrate samples and optimized various factors during sample preparation, sequencing, and computation. As a result, we ~~have~~ identified ~~some~~several key factors that ~~help~~helped improve Hi-C scaffolding, including the choice and preparation of tissues, library preparation conditions, ~~and~~the choice of restriction enzyme(s), ~~as well as~~and the choice of scaffolding program and its usage.

Conclusions: This study provides the first comparison of multiple sample preparation kits/protocols and computational programs for Hi-C scaffolding~~,~~ by an academic third party. We introduce a customized protocol designated ~~the~~ 'inexpensive and controllable Hi-C (iconHi-C) protocol', ~~in~~ which incorporates the optimal conditions ~~revealed by~~ identified in this study ~~have been incorporated~~, and ~~release the resultant~~demonstrated this technique on chromosome-scale genome ~~assembly~~sequences of the Chinese softshell turtle *Pelodiscus sinensis*.

Keywords: Hi-C, genome scaffolding, chromosomes, proximity-guided assembly, softshell turtle

Background

Chromatin, a complex of nucleic acids (DNA and RNA) and proteins, exhibits a complex three-dimensional organization in the nucleus, which enables the intricate regulation of the expression of genome information ~~expression through spatiotemporal controls~~via spatio-temporal control (reviewed in [1]). ~~In order to~~To characterize chromatin conformation on a genomic scale, the Hi-C method was introduced as a derivative of chromosome conformation capture (3C) (Fig. 1A; [2]). This method detects chromatin contacts on a genomic scale ~~through~~via the digestion of ~~crosslinked~~cross-linked DNA molecules with restriction enzymes, followed by proximity ligation of the digested DNA molecules. Massively parallel sequencing of the library ~~harboring~~containing ligated DNA molecules enables the comprehensive quantification of contacts ~~between different genomic regions inside~~both within and between chromosomes, which is presented in a heatmap that is conventionally called the 'contact map' [3].

Analyses of chromatin conformation ~~with~~using Hi-C have revealed more frequent contacts between more closely linked genomic regions, which has recently prompted the use of this method ~~to be employed~~ in ~~elongating~~scaffolding *de novo* genome sequences~~, more recently~~ [4-6]. In *de novo* genome sequencing, the number of assembled sequences is usually far larger than the number of chromosomes in the karyotype of the species of interest, ~~irrespective~~regardless of the sequencing platform chosen [~~5~~7]. The application of Hi-C scaffolding enabled a remarkable enhancement of sequence continuity to reach a chromosome scale, and the integration of fragmentary sequences into longer sequences, which are similar in number to that of chromosomes in the karyotype.

In early 2018, commercial Hi-C library preparation kits were introduced ~~to the market~~ (Fig. 1B), and *de novo* genome assembly was revolutionized by the release of versatile computational programs for Hi-C scaffolding (Table 1), namely LACHESIS [~~6~~4], HiRise [~~7~~8], SALSA [~~8,~~ 9, 10], and 3d-dna [~~10].~~11] (reviewed in [12]). These movements assisted the rise of mass sequencing projects targeting a number of species, such as the Earth BioGenome Project (EBP) [~~11],~~13], the Genome 10K (G10K)/Vertebrate Genome Project (VGP) [~~12, 13~~14], and the DNA Zoo Project [~~14~~15]. Optimization of Hi-C sample preparation, however, has been ~~limitedly attempted [15]. Thus, it remains unexplored~~limited [16], which ~~factor in particular makes a difference in the results~~leaves room for the improvement of efficiency and the reduction of required sample quantity. Thus, the specific factors that are key for Hi-C scaffolding remain unexplored, mainly because of ~~its~~the costly and resource-demanding nature of this technology.

~~Together with~~ In addition to performing protocol optimization using human culture cells, we focused on the softshell turtle *Pelodiscus sinensis* (Fig. 2). This species has been adopted as a study system for evolutionary developmental biology (Evo-Devo), including the study ~~on~~of the formation of the dorsal shell (carapace) (reviewed in [~~16]). It is anticipated that relevant research communities have access~~17]). Access to genome sequences of optimal quality by relevant research communities is desirable in this field. In Japan, live materials (adults and embryos) of this species are available through local farms mainly between May and August, which ~~allows~~implies its high utility for sustainable research. ~~Based on a~~A previous cytogenetic report~~,~~ revealed that the karyotype of this species consists of 33 chromosome pairs including Z and W chromosomes (2n = 66) that show a wide variety of sizes (conventionally categorized

~~into~~as macrochromosomes and microchromosomes) [~~17~~18]. Despite ~~its~~the moderate global GC-content in its whole genome at around 44%, ~~an earlier study suggested~~ the intragenomic heterogeneity of GC-content between and within the chromosomes~~, along with their sizes [18].~~ has been suggested [19]. A wealth of cytogenetic efforts on this species ~~accumulated~~led to the accumulation of fluorescence *in situ* hybridization (FISH)-based mapping data for 162 protein-coding genes covering almost all chromosomes [~~17-19~~18-22], which ~~serves~~serve as structural landmarks for validating genome assembly sequences.

A draft sequence assembly of the softshell turtle genome was built ~~with~~using short reads and was released ~~already~~ in 2013 [~~20~~23]. This sequence assembly achieved the N50 scaffold length of >3.3 Mb but remains fragmented into approximately 20,000 sequences (see Supplementary Table S1). The longest sequence in this assembly is only slightly larger than 16 Mb, which is much shorter than the largest chromosome size estimated from the karyotype report [~~17~~18]. The total size of the assembly is approximately 2.2 Gb, which is a moderate size for a vertebrate species. Because of ~~its~~the affordable genome size, sufficiently complex structure, and availability of validation methods, we reasoned that the genome of this species is a suitable target for our methodological comparison, and its improved genome assembly is expected to assist a wide range of genome-based studies ~~employing~~of this species.

Results

Stepwise QC ~~before~~prior to large-scale sequencing

~~It would be ideal to judge~~The assessment of the quality of prepared libraries before engaging in costly sequencing~~. Following existing ~~ would be ideal. According to the literature [~~15, 21~~16, 24], we routinely control the quality of Hi-C DNAs and Hi-C libraries by observing DNA size shifts ~~with~~via digestion targeting the restriction sites in properly prepared samples (Fig. 3). More concretely, a successfully ligated Hi-C DNA sample should exhibit a slight increase in the length ~~recovery~~ of its restricted DNA fragments after ligation (QC1), which serves as an indicator of qualified samples (e.g., Sample 1 in Fig. 3B). In contrast, an unsuccessfully prepared Hi-C DNA does not exhibit this length recovery (e.g., Sample 2 in Fig. 3B). In a ~~later~~subsequent step, DNA molecules in a successfully prepared HindIII-digested Hi-C library should contain the NheI restriction site at a high probability. Thus, the length distribution observed after ~~the~~ NheI digestion of the prepared library serves as an indicator of qualified or disqualified products (QC2; Fig. 3C). This series of QCs is incorporated into our protocol by default (Supplementary Protocol S1) and can also be performed ~~along~~in combination with sample preparation using commercial kits ~~provided that~~if it employs a single restriction enzyme.

Some of the libraries ~~we have~~ prepared by us passed the QC steps performed before sequencing but yielded an ~~unpreferably~~unfavourably large proportion of ~~unusable~~invalid read pairs. To identify such libraries, we routinely performed small-scale sequencing ~~with the purpose of~~for quick and inexpensive QC (designated ‘QC3’) using the HiC-Pro program [~~22~~25] (see Fig. 4 for the read pair categories assigned by HiC-Pro). Our test ~~with~~using variable input data sizes (500 K~~–~~ to 200 M read pairs) resulted in highly similar breakdowns into different categories of read pair properties (Supplementary Table S2) and guaranteed ~~the QC~~QC3 with an extremely small data

size of 1 M or fewer reads. These post-sequencing QC steps that, which do not incur a large cost, are expected to help avoid the large-scale sequencing of unsuccessful libraries that have somehow passed through the QC1 and QC2 steps. Importantly, libraries that have passed this QCQC3 can be further sequenced in morewith greater depth, as necessary.

Optimization of sample preparation conditions

We identified overt differences between the sample preparation protocols of already published studies and those of commercial kits, especially regarding the duration of fixation and enzymatic reaction as well as the library preparation method used. (Fig. 1B). Therefore, we first sought to optimize the conditions of several preparationof these steps using human culture cells.

To evaluate the effect of the degree of cell fixation, we prepared Hi-C libraries from GM12878 cells fixed for 10 and 30 minutes. Our comparison did not detect any marked differencedifferences in the quality of the Hi-C DNA (QC1; Fig. 5A) and Hi-C library (QC2; Fig. 5B). However, libraries that were prepared with a longer fixation showedtime exhibited a larger proportionsproportion of dangling end read pairs and re-ligationreligation read pairs, as well as a smaller proportion of valid interaction reads (Fig. 5C). IncreasedThe increase in the duration of cell fixation reducesalso reduced the proportion of long-range (>1 Mb) interactions among the overall captured interactions (Fig. 5D).

The reduced preparation time withof commercial Hi-C kits (up to two days according to their advertisement) is attributable mainly to shortened duration of restriction and ligation times (Fig. 1B). To monitor the effect of shortening these

enzymatic reactions, we ~~analyzed~~first analysed the progression of restriction and ligation in a time~~-~~-course experiment using ~~human~~ GM12878 cells. ~~The results show~~We observed the persistent progression of restriction ~~until~~up to 16 hours and of ligation ~~until~~up to 6 hours (Fig. 6). To scrutinize further the possible adverse effects of the prolonged reaction, Hi-C libraries of GM12878 cells were prepared with variable durations of restriction digestion (1 hour and 16 hours) and ligation (15 minutes, 1 hour, and 6 hours). We found that the proportions of dangling end and religation read pairs were reduced in cases with an extended duration of restriction digestion (Supplementary Table S4). The yield of the library, which can be estimated from the number of PCR cycles, increased with the extended duration of ligation without any effect on the proportion of valid interaction read pairs (Supplementary Table S4). The proportion of valid interaction read pairs containing the proper DpnII junction sequence ‘GATCGATC’ also remained unchanged, suggesting that the prolonged reaction times did not induce any adverse effects, such as star activity of the restriction enzyme.

Multifaceted comparison using softshell turtle samples

~~On the basis of~~Based on the detailed optimization of the sample preparation conditions described above, we built an original protocol, designated the ‘iconHi-C protocol’, ~~with~~that included a 10 ~~min~~minute-long cell fixation, 16 hour-long restriction, 6 hour-long ligation, and successive QC steps (Methods; also see Supplementary Protocol S1; Fig. 1B).

We performed Hi-C sample preparation and scaffolding using tissues from a female Chinese softshell turtle which ~~is known to have~~has both Z and W chromosomes [~~17]. For this purpose, we~~18]. We prepared Hi-C libraries ~~with variable~~using various

tissues (liver or blood cells), restriction enzymes (HindIII or DpnII), and protocols (our iconHi-C protocol, the Arima ~~Genomics~~ kit in conjunction with the KAPA Hyper Prep Kit, or the Phase ~~Genomics~~ kit~~)~~), as outlined in Fig. 7A (see Supplementary Table ~~S3~~S5; Supplementary Fig. S1). As in some of the existing protocols (e.g~~., [23~~. [26]), we performed T4 DNA polymerase treatment in our iconHi-C protocol (Library a–d), expecting reduced proportions of 'dangling end' read pairs that contain no ligated junction, and thus do not contribute to Hi-C scaffolding. We also incorporated this T4 DNA polymerase treatment ~~in~~into the workflow of the Arima kit (Library e vs. Library f without this additional treatment). ~~We also~~Furthermore, we tested a lesser degree of PCR amplification (11 cycles) ~~along~~together with the use of the Phase ~~Genomics~~ kit which ~~compels~~recommends as many as 15 cycles by default (Library h vs. Library g; Fig. 7A).

~~The~~All samples prepared ~~with~~using the iconHi-C protocol~~, which is compatible with the abovementioned~~ passed both controls, QC1 and QC2~~, were all judged as qualified, by these QCs~~ (Fig. 7B). The prepared Hi-C libraries were sequenced to obtain one million ~~127nt~~127 nt-long read pairs and were subjected to ~~post-sequencing QC with~~QC3 using the HiC-Pro program (Fig. 8). As a result of this ~~QC~~QC3, the largest proportion of 'valid interaction' pairs was observed for Arima libraries (Library e and f). ~~As for~~Regarding the iconHi-C libraries (Library a–d), fewer 'unmapped' and 'religation' pairs were detected ~~with~~for the DpnII libraries ~~than~~compared with HindIII libraries. It should be noted that the ~~QC results for~~QC3 of the softshell turtle libraries generally produced lower proportions of the 'valid interaction' category and larger proportions of 'unmapped pairs' and 'pairs with singleton' than ~~those for~~with the human libraries. This cross-species difference ~~is accounted for by possibly~~may be

attributable to the use of incomplete genome sequences used as a reference for Hi-C read mapping (Supplementary Table S1). This evokesinvokes a caution inwhen comparing QC results across species.

Scaffolding withusing variable inputsinput and computational conditions

In this study, only well-maintained, open-source programs, namelyi.e., 3d-dna and SALSA2, were used in conjunction with variable combinations of an input library, anlibraries, input read amount, anamounts, input sequence cutoff lengthcut-off lengths, and a number of iterative misjoin correction rounds (Fig. 9A). As a result of scaffolding, we observed a wide spectrum of basic metrics, including the N50 scaffold length (0.6–303 Mb), the largest scaffold length (8.7–703 Mb), and the number of chromosome-sized (>10 Mb) sequences (0–65) (Fig. 9; Supplementary Table S4S6).

First of all, with, using the default parameters, 3d-dna consistently produced more continuous assemblies than did SALSA2 (see Assembly 1 vs. 5, 3 vs. 6, 9 vs. 10, and 11 vs. 12 in Fig. 9). Second, increasingthe increase in the number of iterative corrections (‘-r’ option withof 3d-dna) resulted in relatively large N50 lengths, but with more missing orthologsorthologues (see Assembly 3 and 13–1514). Third, a smaller input sequence cutoffcut-off length (‘-i’ option withof 3d-dna) resulted in a smaller number of resultant scaffolds but again, with more missing orthologsorthologues (see Assembly 13, 16–183 and 15–17). Fourth, usingthe use of the liver libraries consistently resulted in a higher continuity than usingthe use of the blood cell libraries (see Assembly 1 vs. 2 as well asand 3 vs. 4 in Fig. 9).

Of those, Assembly 8, employingwhich resulted from input Hi-C reads derived from both liver and blood, exhibited an outstandingly large N50 scaffold length (303

Mb) but a larger number of undetected reference ~~ortholog~~orthologues (141 ~~orthologs~~orthologues) than most of the other assemblies. The largest scaffold (scaffold 5) in this assembly is approximately 703 Mb long, causing ~~the~~a large N50 length, and accounts for approximately one-third of the whole genome in length, as a result of possible ~~overassembly bridging~~chimeric assembly that bridged 14 putative chromosomes (see Supplementary Fig. ~~S2~~S4).

The choice of restriction enzymes has not ~~yet~~ been discussed in depth~~,~~ in the context of genome scaffolding. ~~In the present study~~Here, we ~~separately~~ prepared Hi-C libraries separately with HindIII and DpnII. We did not mix multiple enzymes in ~~a~~the same reaction (~~apart from~~other than using the Arima kit which originally ~~employing~~employs two enzymes~~) and instead~~); rather, we performed a single scaffolding run with both HindIII-based and DpnII-based reads (see Assembly 7 in Fig. 9). ~~Our~~As expected, our comparison of multiple metrics ~~expectedly highlights~~yielded a more successful result with DpnII than with HindIII (see Assembly 1 vs. 3 as well as 2 vs. 4; Fig. 9). However, the mixed input of HindIII-based and DpnII-based reads did not necessarily yield a better scaffolding result (see Assembly 3 vs. 7).

To gain additional insight regarding the evaluation of the scaffolding results, we assessed the contact maps constructed upon the Hi-C scaffolds (Supplementary Fig. S5). The comparison of Assembly 3, 9 and 11, which represent the three different preparation methods, revealed anomalous patterns, particularly for Assembly 11, with intensive contact signals separated from the diagonal line that indicate the presence of errors in the scaffolds [15]. We also performed genome-wide alignments between the Hi-C scaffolds obtained. The comparison of Assembly 3, 9, and 11 revealed a high similarity between Assembly 3 and 9, while Assembly 11 exhibited a significantly

larger number of inconsistencies against either of the other two assemblies (Supplementary Fig. S6). These observations are consistent with the evaluation based on sequence length and gene space completeness, which alone does not, however, provide a reliable metric for the assessment of the quality of scaffolding.

Validation of scaffolding results ~~with~~using transcriptome and FISH data

In addition to the above-mentioned evaluation of the scaffolding results ~~based on sequence length and gene space completeness~~, we ~~attempted to evaluate~~assessed the sequence continuity ~~with~~using independently obtained data. First, we mapped assembled transcript sequences onto our Hi-C scaffold sequences (see Methods). This did not ~~reveal~~show any substantial differences between the assemblies (Supplementary Table ~~S5~~S7), probably because the sequence continuity after Hi-C scaffolding ~~already~~ exceeded that of RNA-seq library inserts, even when the ~~lengths~~length of intervening introns in the genome ~~are taken into consideration.~~was considered. The present analysis with RNA-seq data did not provide an effective ~~resort~~source of continuity validation.

Second, we referred to the fluorescence *in situ* hybridization (FISH) mapping data ~~for~~of 162 protein-coding genes from published cytogenetic studies [~~17-19~~18-22], which allowed us to check the locations of those genes with our resultant Hi-C assemblies. In this analysis, we evaluated Assembly 3, 7, and 9 (see Fig. 9A) that showed better scaffolding results in terms of sequence length distribution and gene space completeness (Fig. ~~9B~~9D). As a result, we confirmed the positioning of almost all genes and their continuity over the centromeres, which encompassed not only large but also small chromosomes (conventionally called '~~macro-'~~macrochromosomes' and '~~micro-chromosomes'~~microchromosomes'; Fig. 10). Two genes that were not

confirmed by Assembly 7 (*UCHL1* and *COX15*; Fig. 10) were found in separate scaffold sequences that were shorter than 1 Mb, which indicates insufficient scaffolding. ~~On the other hand~~Conversely, the gene array including *RBM5*, *TKT*, *WNT7A*, and *WNT5A*, previously shown by FISH, was consistently unconfirmed by all ~~the~~ three assemblies (Fig. 10), which did not provide any ~~clue~~clues for among-assembly evaluation or ~~even indicated~~perhaps indicates an erroneous interpretation of FISH data in a previous study.

Discussion

Starting ~~materials~~material: not genomic DNA extraction but *in situ* cell fixation

In genome sequencing, best practices for high molecular weight DNA extraction have often been discussed (e.g.~~, [24~~. [27]). This factor is fundamental to building longer contigs, ~~whether employing~~regardless of the use of short-read or long-read sequencing platforms. ~~Also~~Moreover, the proximity ligation method using Chicago libraries provided by Dovetail Genomics which is based on *in vitro* chromatin reconstruction [~~7~~8], uses genomic DNA as starting ~~materials. Instead~~material. In contrast, proximity~~-~~-guided assembly enabled by Hi-C employs cellular nuclei ~~preserving~~with preserved chromatin conformation, which brings a new technical challenge ~~for~~regarding appropriate sampling and sample preservation in genomics.

In ~~preparing~~ the preparation of the starting ~~materials~~material, it ~~seems~~is important to optimize the degree of cell fixation depending on ~~your~~ sample choice, to obtain an optimal result in Hi-C scaffolding (Fig. 5). Another practical ~~lesson~~

~~about~~indication of tissue choice was obtained by examining Assembly 8 (Fig. 9A). This assembly was produced by 3d-dna scaffolding ~~with~~using both liver and blood libraries (Library b and d), which led to an unacceptable result possibly caused by ~~overassembly~~over-assembly (Fig. 9B–D; also see Results). It is likely that ~~enhanced~~increased cellular heterogeneity, which possibly ~~introducing~~introduces excessive conflicting chromatin contacts, did not allow the scaffolding program to ~~properly~~ group and order the input genome sequences properly. In brief, we recommend the use of samples with modest cell-type heterogeneity that are amenable to thorough fixation.

Considerations ~~in~~regarding sample preparation

In this study, we ~~could~~did not test all commercial Hi-C kits available in the market. This ~~is~~was partly because the Dovetail Hi-C kit specifies ~~a~~the non-open source program HiRise as the only supported downstream computation solution and does not allow a direct comparison with other kits, namely those from Phase Genomics and Arima Genomics.

According to our ~~calculation, it would be at least three times more economical to prepare~~calculations, the preparation of a Hi-C library ~~with~~using the iconHi-C protocol would be at least three times cheaper than ~~with~~the use of a commercial kit. Practically, the cost difference would be even larger, either when ~~one cannot fully consume~~ the purchased kit is not fully consumed or when ~~one cannot undertake~~the post-sequencing computation steps ~~and thus cover~~cannot be undertaken in-house, which implies additional outsourcing ~~cost for this~~costs.

~~Genomic~~The genomic regions that are targeted by Hi-C are determined by the

choice of restriction enzymes. Theoretically, 4-base cutters (e.g.,. DpnII), which potentially withhave more frequent restriction sites on the genome, are expected to provide a higher resolution than 6-base cutters (e.g., HindIII) [15].16]. Obviously, the use of restriction enzymes that were not employed in this study might be promising in the adaptation of the protocol to organisms with variable GC-content or methylation profiles. However, itthis might not be so straightforward when considering the species-by-speciesinterspecies variation ofin GC-content, as well as its and the intra-genomic heterogeneity, are taken into consideration.. The use of multiple enzymes in a single reaction could beis a promising, but approach; however, from a computational viewpoint, not all scaffolding programs are compatible with multiple enzymes from a computational viewpoint (see Table 1 for a comparison of the specification of scaffolding program specifications).programs). Another technical downside of this approach is the incompatibility of DNA ends restricted by multiple enzymes, with restriction-based QCs, such as the QC2 instep of our iconHi-C protocol (Fig. 3). Therefore, in this study, DpnII and HindIII were used separately employed in conjunction withinin the iconHi-C protocol, which resulted in a higher scaffolding performance with the DpnII library (Figs. 8 and 9), as expected. In addition, we input the separately prepared DpnII and HindIII libraries together in scaffolding (Assembly 7), but this attemptapproach did not lead to higher scaffolding performance (Figs. 9B–D and 10). The Arima Hi-C kit employs two different enzymes that can produce a much more combinationsgreater number of restriction sitessite combinations, because one of thethese two enzymes recognizes the nucleotide stretch 'GANTC'. Scaffolding with the libraries prepared using this kit resulted in one of the most acceptable assemblies (Assembly 9). However, this result did not explicitly exceed the performance of

scaffolding with the iconHi-C libraries, including the one ~~employing only~~that used a single enzyme (DpnII~~ (~~; Library d).

~~One ~~Overamplification by PCR is a concern ~~about~~regarding the use of commercial kits (~~except ~~with the exception of the Arima ~~Hi-C ~~kit used with the Arima-QC2) ~~is overamplification by PCR, as~~because their manuals specify the use of a certain ~~numbers~~number of PCR cycles *a priori* (15 cycles for the Phase ~~Genomics Proximo Hi-C ~~kit and 11 cycles for the Dovetail Hi-C kit) (Supplementary Table S8). In our iconHi-C protocol, an optimal number of PCR cycles is estimated by means of a preliminary real-time PCR using a small aliquot (~~Step11~~Step 11.25~~–~~ to 11.29 in Supplementary Protocol S1~~)~~), as done traditionally ~~performed ~~for other library types (e.g., [~~25~~28]). This procedure allowed us to ~~minimize~~reduce the number of PCR cycles, down to as few as five cycles (Supplementary Table ~~S3~~S5). The Dovetail Hi-C kit recommends ~~that one consumes~~the use of larger amounts of kit components than that specified for a single sample, depending on the genome size, as well as the degree of genomic heterozygosity and repetitiveness, of the species of interest. ~~However~~In contrast, with our iconHi-C protocol, we always ~~performed~~prepared a single library~~ preparation, irrespective~~, regardless of those species-specific factors, which ~~we understand suffices~~seemed to suffice in all the cases~~ we have~~ tested.

Commercial Hi-C kits, which usually ~~advertised for ~~advertise easiness and quickness of use, have largely shortened the protocol down to two days, ~~in comparison~~compared with ~~existing~~the published non-commercial protocols (e.g., [~~15, 23~~16, 26]). Such time-saving protocols are achieved mainly by ~~shortened durations~~shortening the duration of restriction enzyme digestion and ligation (Fig. 1B). Our assessment, however, ~~showed~~revealed unsaturated reaction within ~~such~~the

shortened time frames employed in the commercial kits (Fig. ~~6). Also, our~~6), which was accompanied by an unfavorable composition of read pairs (Supplementary Table S4). Our attempt to insert a step ~~for~~of T4 DNA polymerase treatment in the sample preparation ~~with~~of the Arima ~~Hi-C~~ kit protocol resulted in reduced ‘dangling end’ reads (Library e vs. ~~Library~~ f in Fig. 8). ~~As for~~Regarding the Phase ~~Genomics Proximo Hi-C~~ kit, transposase-based library preparation contributes largely to ~~shortening~~ its shortened protocol, but this ~~decreases the operability~~does not allow flexible control of library insert lengths. ~~Especially if Hi-C~~Recent protocols (versions 1.5 and 2.0) of the Phase kit instruct users to employ a largely reduced DNA amount in the tagmentation reaction, which should mitigate the difficulty in controlling insert length but require excessive PCR amplification. The Arima and Phase kits assume that the quality control of Hi-C DNA is based on the yield, and not the size, of DNA (see Fig. 1B). Nevertheless, quality control based on DNA size (equivalent to QC1 in iconHi-C) is feasible by taking aliquots at each step of sample preparation ~~is performed for a limited~~. In particular, if preparing a small number of samples for Hi-C, as ~~practiced~~practised typically for genome scaffolding, one ~~would~~should opt to consider these points, even ~~in~~when using commercial kits, ~~in order~~ to ~~further~~ improve the quality of the prepared libraries and scaffolding products.

Considerations ~~in~~regarding sequencing

The quantity of Hi-C read pairs to be input for scaffolding is critical because it accounts for the majority of the cost of Hi-C scaffolding. Our protocol introduces a thorough safety system to prevent sequencing unsuccessful libraries, ~~firstly with~~first by performing pre-sequencing QCs for size shift ~~analysis~~analyses (Fig. 3) and ~~secondly~~

withsecond via small-scale (down to 500 K read pairs) sequencing (see Results; also see Supplementary TableTables S2, S6 and S9).

Our comparison showsshowed a dramatic decrease in assembly quality when less than in cases in which <100 M read pairs were used (see the comparison amongof Assembly 19–2318–22 described above in; Fig. 9). Still; also see [29]). Nevertheless, we obtained optimal results with a smaller number of reads (ca. 160 M per 2.2 Gb of genome) than that recommended by the manufacturers of commercial kits (e.g., 100 M per 1 Gb of genome for the Dovetail Hi-C kit and 200 M per Gb of genome for the Arima Hi-C kit). As generally and repeatedly discussed, [29][29], the proportion of informative reads and their diversity, rather than just the overall number of all obtained reads, areis critical.

In terms of read length, we did not perform any comparisoncomparisons in this study. Longer reads may enhance the fidelity in characterizingof the characterization of the read pair propertyproperties and allowsallow precise QC. StillNevertheless, the existing Illumina sequencing platform has enabled economicalthe less expensive acquisition of 150 nt-long paired-end reads, which did not prompt us to vary the read length.

Considerations inregarding computation

In this study, 3d-dna produced a more reliable scaffolding output than did SALSA2, whether sample preparation employed a single or multiple enzyme(s) (Fig. 9B–D). On the other hand, 3d-dna needed more required a greater amount of time to completefor the completion of scaffolding than did SALSA2. Apart from the choice of the program, there are quite a fewseveral points to consider, in order to achieveshould be considered

if successful scaffolding for a smaller investment~~.~~ is to be achieved. In general, ~~it is advised not to take~~ Hi-C scaffolding results should not be taken for granted, and it is necessary to improve them by referring to contact maps~~,~~ using an interactive tool, such as Juicebox [~~14~~15]. In this study, however, we compared raw scaffolding ~~outputs~~output to evaluate sample preparation and reproducible computational steps.

~~Our study employed variable~~We used various parameters of the scaffolding programs (Fig. 9A). First, ~~available~~the Hi-C scaffolding programs that are available currently have different default length cut-off values for input sequences (e.g., 15000 bp for the '-i' parameter ~~'-i' within~~in 3d-dna and 1000 bp for the '-c' parameter ~~'-c' within~~in SALSA2). Only sequences that are longer than the cut-off length value contribute to sequence ~~elongation~~scaffolding towards ~~the~~ chromosome sizes, ~~and those~~ while sequences shorter than ~~that~~the cut-off length are implicitly excluded from the scaffolding process and remain unchanged. Typically ~~with~~, when using the Illumina sequencing platform, genomic regions with unusually high frequencies of ~~GC-content and~~ repetitive elements and GC-content are not assembled into sequences with a sufficient ~~lengths~~length (see [~~26~~30]). Such genomic regions tend to be excluded from chromosome-scale Hi-C scaffolds because their length is smaller than the threshold. ~~It is also possible that such~~Alternatively, these regions ~~are~~may be excluded because few Hi-C read pairs are mapped to ~~such regions~~them, even if they exceed the ~~cutoff~~cut-off length. ~~One needs to deliberately set the~~ The deliberate setting of a cut-off length ~~cutoff in accordance with the overall continuity of the input assembly and possible interest into~~is recommended if particular~~, fragmentary~~ sequences ~~expected to be elongated.~~with relatively small lengths are the target of scaffolding. It should be ~~warned~~noted that lowering the length threshold can result in frequent misjoins in the scaffolding output

(Fig. 9B–D) or ~~too much~~in overly long computational ~~time~~times. Regarding the number of iterative misjoin correction rounds (the '-r' parameter ~~'-r' with~~in 3d-dna and 'i' ~~with~~parameter in SALSA2), our attempts ~~with~~of using increased values did not necessarily yield ~~favorable~~favourable results (Fig. 9B–D~~), which~~). This did not provide a consistent optimal range of values but rather suggests the importance of performing multiple scaffolding runs with ~~varied~~varying parameters.

Considerations ~~in assessing~~regarding the assessment of chromosome-scale genome sequences

Our assessment ~~with~~using cytogenetic data confirmed the continuity of gene linkage over the obtained chromosome-scale sequences (Fig. 10). This validation was ~~necessitated~~required by the almost saturated scores of typical gene space completeness assessment tools such as BUSCO (Supplementary Table ~~S4) as well as~~S6) and by transcript contig mapping (Supplementary Table ~~S5), both~~S7), neither of which ~~did not provide~~provided an effective metric for evaluation.

For further evaluation of our scaffolding results, we referred to the sequence length ~~distribution~~distributions of the genome assemblies of other turtle species that are regarded as being chromosome-scale data. This ~~showed comparable~~ analysis yielded values ~~for~~of the basic metrics that were comparable to those of our Hi-C ~~scaffolding results on~~ scaffolds of the softshell turtle, ~~that is, a~~i.e. an N50 length of 127.5 Mb and ~~the~~a maximum sequence length of 344.5 Mb for the genome assembly of the green sea turtle (*Chelonia mydas*) ~~genome assembly~~ released by the DNA Zoo Project [15] and ~~a~~an N50 length of 131.6 Mb and ~~the~~a maximum length of 370.3 Mb for the genome assembly of the Goode's thornscrub tortoise (*Gopherus evgoodei*) ~~genome assembly~~

released by the Vertebrate Genome Project (VGP~~).~~) [14]. Scaffolding results should be evaluated by referring to ~~an estimate~~the estimated N50 length and the maximum length based on the actual ~~number~~value and to the length distribution of chromosomes in the intrinsic karyotype of the species in question, or of its close relative. Turtles tend to have ~~the~~an N50 length of approximately 130 Mb and ~~the~~a maximum length of 350 Mb, while many teleost fish genomes exhibit an N50 length ~~of~~ as low as 20–30 Mb and ~~the~~a maximum length of <100 Mb [~~27~~31]. If these ~~metrics show~~values are excessive ~~values,~~, the scaffolded sequences ~~harbor~~harbour overassembly ~~that~~, which erroneously boosts length-based metrics. ~~Larger~~Thus, higher values ~~that researchers~~, which are conventionally ~~regard~~regarded as signs ~~for~~of successful sequence assembly, do not necessarily indicate higher precision.

The total length of assembly sequences is expected to increase after Hi-C scaffolding, because scaffolding programs simply insert a stretch of the unassigned base 'N' with a uniform length between input sequences in most cases (500 bp as a default ~~within~~in both 3d-dna and SALSA2). However, this has a minor impact on the total ~~assembly sequence~~ length~~.~~ of assembled sequences. In fact, ~~inserting~~ the insertion of 'N' stretches ~~of~~with an arbitrary ~~lengths~~length has been an implicit, rampant practice even before Hi-C scaffolding prevailed—for example, the most and second most frequent lengths of the 'N' stretch in the publicly available zebrafish genome assembly Zv10 are 100 and 10 bp, respectively.

Conclusions

In this study, we introduced the iconHi-C protocol ~~in~~ which implements successive QC steps ~~are implemented, and~~. We also assessed ~~possible keys~~potential key factors for

improving Hi-C scaffolding. Overall, our study ~~shows~~showed that ~~a~~ small ~~variation~~variations in sample preparation or computation for scaffolding can have a large impact on scaffolding output, and that any scaffolding output should ideally be validated ~~by~~using independent information, such as cytogenetic data, long reads, or genetic linkage maps. ~~Our~~The present study aimed to evaluate the output of reproducible computational steps, which in practice should be followed by ~~modifying~~the modification of the raw scaffolding output by referring to independent information or by ~~analyzing~~analysing chromatin contact maps. The study employed ~~only~~ limited combinations of species, sample prep methods, scaffolding programs, and its parameters, and we will continue ~~testing~~to test different conditions for kits/programs that did not necessarily perform well here ~~with~~using our specific materials.

Methods

Initial genome assembly sequences

The softshell turtle (*Pelodiscus sinensis*) assembly published previously [~~20~~23] was downloaded from NCBI GenBank (GCA_000230535.1), whose gene space completeness and length statistics were assessed by gVolante [~~28~~32] (see Supplementary Table S1 for the assessment results). Although it could be suggested to remove haplotigs before Hi-C scaffolding [~~29~~33], we omitted this step because of the low frequency of the reference ~~orthologs~~orthologues with multiple copies (0.72~~ ~~%; Supplementary Table S1), indicating a minimal degree of haplotig contamination.

Animals and cells

We sampled tissues (liver and blood cells) from a female purchased from a local farmer in Japan, because the previous whole genome sequencing used the whole blood of a female [2023]. All the experiments were conducted in accordance with the Guideline of the Institutional Animal Care and Use Committee of RIKEN Kobe Branch (Approval ID: A2017-12).

HumanThe human lymphoblastoid cell line GM12878 was purchased from the Coriell Cell Repositories and cultured in RPMI-1640 mediamedium (Thermo Fisher Scientific) supplemented with 15% FBS, 2 mM L-glutamine, and 1xa 1× antibiotic-antimycotic solution (Thermo Fisher Scientific), at 37 °C, 5-% CO_2, as described previously [3034].

Hi-C sample preparation using the original protocol

We have made modifications to a protocol introducedthe protocols that are available in previousthe literature [23, 313, 26, 35] (Fig. 1B). The full version of the modifiedour 'inexpensive and controllable Hi-C (iconHi-C)' protocol is described in Supplementary Protocol S1. and available at Protocols.io (https://www.protocols.io/private/950FFCBDE7C46D1598CA7DDFE7441C9F).

Hi-C sample preparation using commercial kits

The Proximo Hi-C kit (Phase Genomics) which employs the restriction enzyme Sau3A1 and transposase-based library preparation [3236] (Fig. 1B) was used for preparingto prepare a library from the 50 mg of the softshell turtle liver following its according to the official ver. 1.0 animal protocol provided by the manufacturer (Library g in Fig. 7A) and a library from the 10 mg of the liver that was amplified with a reduced number of

PCR cycles based on a preliminary real-time qPCR using an aliquot (Library h; see [~~25~~28] for the ~~detail~~details of the pre-determination of the optimal number of PCR cycles). The Arima ~~Hi-C~~ HiC kit (Arima Genomics~~)~~), which employs a restriction enzyme cocktail (Fig. 1B~~)~~), was used in conjunction with the KAPA Hyper Prep Kit (KAPA Biosystems), protocol ver. A160108 v00, to prepare a library using the softshell turtle liver, ~~following~~according to its official animal vertebrate tissue protocol (ver. A160107 v00) (Library f) and a library with an additional step of T4 DNA polymerase treatment for reducing ‘dangling end’ reads (Library e). This additional treatment is detailed in Step 8.2 (for DpnII-digested samples) ~~in~~of Supplementary Protocol S1.

DNA sequencing

Small-scale sequencing for library QC (QC3) was performed in-house to obtain 127 nt-long paired-end reads on an Illumina HiSeq 1500 in the Rapid Run Mode. For evaluating the effects of variable duration of the restriction digestion and ligation reactions, sequencing was performed on an Illumina MiSeq using the MiSeq Reagent Kit v3 to obtain 300 nt-long paired-end reads. Large-scale sequencing for Hi-C scaffolding was performed to obtain 151 nt-long paired-end reads on an Illumina HiSeq X. The obtained reads ~~were subjected to~~underwent quality control ~~with~~using FastQC ver. 0.11.5 (https://www.bioinformatics.babraham.ac.uk/projects/fastqc/), and low-quality regions and adapter sequences in the reads were removed using Trim Galore ver. 0.4.5 (https://www.bioinformatics.babraham.ac.uk/projects/trim_galore/) with the parameters ‘-e 0.1 -q 30’.

Post-sequencing quality control (QC3) of Hi-C libraries

For post-sequencing library QC, one million trimmed read pairs for each Hi-C library were sampled using the ‘subseq’ function of the program seqtk ver. 1.2-r94 (https://github.com/lh3/seqtk). The resultant sets of read pairs were processed using HiC-Pro ver. 2.11.1 [~~22~~25] with bowtie2 ver. 2.3.4.1 [~~33~~37] to evaluate the insert structure and mapping status onto the softshell turtle genome assembly PelSin_1.0 (GCF_000230535.1) or the human genome assembly hg19. This resulted in ~~the~~ categorization ~~between~~as valid interaction pairs and invalid pairs, ~~and~~with the latter ~~is~~being divided further into ‘dangling end’, ‘religation’, ‘self circle’, and ‘single-end’ pairs (Fig. 4). To process the read pairs derived from the libraries prepared using either HindIII or DpnII (Sau3AI) with the iconHi-C protocol (Library a–d) and the Phase ~~Genomics Proximo Hi-C~~ kit (Library g and h), the restriction fragment file required by HiC-Pro was prepared according to the script ‘digest_genome.py’ ~~provided with~~of HiC-Pro. To process the reads derived from the Arima ~~Hi-C~~ kit (Library e and f), all restriction sites (‘GATC’ and ‘GANTC’) were inserted into the script. In addition, the nucleotide sequences of all possible ligated sites generated by restriction enzymes were included in a configuration file of HiC-Pro. The details of this procedure and the sample code used are included in Supplementary Protocol S2.

Computation for Hi-C scaffolding

~~In order to~~To control our comparison with intended input data sizes, a certain ~~numbers~~number of trimmed read pairs were sampled for each library with seqtk, as described above. Scaffolding was processed with the following methods employing two program pipelines, 3d-dna and SALSA2.

Scaffolding ~~with the program~~via 3d-dna was ~~preceded by~~ performed using Hi-

C read mapping onto the genome with Juicer ver. 20180805 [~~34~~38] using the default parameters with BWA ver.0.7.17-r1188 [~~35~~39]. The restriction fragment file required by Juicer was prepared by the script ‘generate_site_positions.py’ ~~provided with~~ script of Juicer~~ or our~~. By converting the restriction fragment file of HiC-Pro to the Juicer format, an original script that was compatible with multiple restriction enzymes ~~to convert the restriction fragment file of HiC-Pro to the format required by Juicer~~ was prepared (Supplementary Protocol S2). Scaffolding ~~with~~via 3d-dna ver. 20180929 was performed ~~with~~using variable parameters (see Fig. 9A).

Scaffolding ~~with the program~~via SALSA2 using Hi-C reads was preceded by Hi-C read pair processing with the Arima mapping pipeline ver. 20181207 (https://github.com/ArimaGenomics/mapping_pipeline) together with BWA, SAMtools ver. 1.8-21-gf6f50ac [~~36]~~40], and Picard ver. 2.18.12 (https://github.com/broadinstitute/picard). The mapping result in the binary alignment map (bam) format was converted into a BED file by bamToBed of Bedtools ver. 2.26.0 [~~37], whose~~41], the output of which was used as ~~an~~the input of scaffolding using SALSA2 ver. 20181212 with the default parameters.

Completeness assessment of Hi-C scaffolds

gVolante ver. 1.2.1 [~~28~~32] was used to perform an assessment of the sequence length distribution and gene space completeness based on the coverage of one-to-one reference ~~orthologs~~orthologues with BUSCO v2/v3 employing the one-to-one ~~ortholog~~orthologue set ‘Tetrapoda’ supplied with BUSCO [~~38]. For the assessment, no threshold of~~42]. No cut-off length was ~~set~~used in this assessment.

Continuity assessment ~~with~~using RNA-seq read mapping

Paired-end reads obtained by RNA-seq of softshell turtle embryos at multiple stages were downloaded from NCBI SRA (DRX001576) and were assembled ~~with the program~~using Trinity ver. 2.7.0 [~~39~~43] with ~~the~~ default parameters. The assembled transcript sequences were mapped ~~with pblat [40]~~ to the Hi-C scaffold sequences~~,~~ with pblat [44], and the output was assessed with isoblat ver. 0.31 [~~41~~45].

Comparison with chromosome FISH results

Cytogenetic validation of Hi-C scaffolding results was performed by comparing the gene locations on the scaffold sequences with those ~~in preexisting~~provided by previous chromosome FISH ~~data~~ for 162 protein-coding genes [~~17-19~~18-22]. The nucleotide exonic sequences for those 162 genes were retrieved from GenBank ~~were~~and aligned with Hi-C scaffold sequences using BLAT ver. 36x2 [~~42], and~~46], followed by the analysis of their positions and orientation along the Hi-C scaffold sequences ~~were analyzed~~.

Availability of supporting data

All sequence data generated ~~from~~in this study have been submitted to the DDBJ Sequence Read Archive (DRA) under accession IDs DRA008313 and DRA008947. The datasets supporting the results of this article are available in ~~the~~ FigShare (https://figshare.com/s/6ea495a65fc231a74458).

Additional files

Supplementary Figure S1. ~~Quality control~~DNA size distribution of the softshell turtle

Hi-C libraries.

Supplementary Figure S2. Pre-sequencing quality control of softshell turtle blood Hi-C libraries (Library a and b).

Supplementary Figure S3. Pre-sequencing quality control (QC2) of the Hi-C libraries generated using the Phase kit (Library g and h).

Supplementary Figure S4. Structural analysis of the possibly overassembledchimeric scaffold in Assembly #8.

Supplementary Figure S3. ResultsS5. Hi-C contact maps for selected softshell turtle Hi-C scaffolds.

Supplementary Figure S6. Pairwise alignment of quality controls before sequencingHi-C scaffolds.

Supplementary Table S1. Statistics of the Chinese softshell turtle draft genome assembly before Hi-C.

Supplementary Table S2. HiC-Pro results offor the human GM12878 HindIII Hi-C library with reduced reads.

Supplementary Table S3. Quality control of the human GM12878 Hi-C libraries.

Supplementary Table S4. Effect of the duration of restriction enzyme digestion and ligation.

Supplementary Table S5. Quality control of Hi-C libraries.

Supplementary Table S6. Scaffolding results with variable input data and computational parameters.

Supplementary Table S5S7. Mapping results of assembled transcript sequences onto Hi-C scaffolds.

Supplementary Table S6S8. Effect of variable degrees of PCR amplification.

Supplementary Table S9. HiC-Pro results offor the softshell turtle liver DpnII librarylibraries (Library d, e, and h) with reduced reads.

Supplementary Table S7. Quality control of the human GM12878 Hi-C libraries

Supplementary Protocol S1. Protocol of iconHi-C protocol.

Supplementary Protocol S2. Computational protocol to support the use of multiple enzymes.

Abbreviations

PCR: polymerase chain reaction; FISH, fluorescence *in situ* hybridization; BUSCO, benchmarking universal single-copy orthologs; NCBI, National Center for Biotechnology Information; NGS, next generation DNA sequencing.

Funding

This work was supported by intramural grants within RIKEN to S.K. and I.H. and by a Grant-in-Aid for Scientific Research on Innovative Areas ~~to I.H. (18H05530)~~ from the Ministry of Education, Culture, Sports, Science, and Technology (MEXT) to I.H. (18H05530).

Competing interests

The authors declare that they have no competing interests.

Acknowledgements

The authors acknowledge Naoki Irie, Juan Pascual Anaya and Tatsuya Hirasawa in Laboratory for Evolutionary Morphology, RIKEN BDR for suggestions for sampling, Rawin Poonperm for comments and discussion on the iconHi-C protocol, Olga Dudchenko, Erez Lieberman-Aiden, Arang Rhie, Sergey Koren, and Jay Ghurye for their technical suggestions for sample preparation and computation, Yoshinobu Uno for guidance ~~to~~in the cytogenetic data interpretation, and Anthony Schmitt of Arima Genomics and Stephen Eacker of Phase Genomics for providing information about the

Hi-C kits. ~~They~~The authors also thank the other members of the Laboratory for Phyloinformatics and Laboratory for Developmental Epigenetics in RIKEN BDR for technical support and discussion.

Author contributions

S.K., I.H., H.M., and M.K. conceived the study. M.K. and K.T. performed laboratory works, and O.N. performed bioinformatic analysis. M.K., O.N., and H.M. analyzed the data. S.K., M.K., and O.N. drafted the manuscript. All authors contributed to the finalization of the manuscript.

References

1. Rowley MJ and Corces VG. Organizational principles of 3D genome architecture. ~~Nature Reviews Genetics.~~Nat Rev Genet. 2018;19 12:789-800. doi:10.1038/s41576-018-0060-8.

2. Lieberman-Aiden E, van Berkum NL, Williams L, Imakaev M, Ragoczy T, Telling A, et al. Comprehensive ~~Mapping~~mapping of ~~Long-Range Interactions Reveals Folding Principles~~long-range interactions reveals folding principles of the ~~Human Genome~~human genome. Science. 2009;326 5950:289-93. doi:10.1126/science.1181369.

3. Rao ~~Suhas SP~~SS, Huntley ~~Miriam H~~MH, Durand ~~Neva C~~NC, Stamenova ~~Elena K~~EK, Bochkov ~~Ivan D~~ID, Robinson ~~James T~~JT, et al. A 3D ~~Map~~map of the

Human Genomehuman genome at Kilobase Resolution Reveals Principleskilobase resolution reveals principles of Chromatin Loopingchromatin looping. Cell. 2014;159 7:1665-80. doi:10.1016/j.cell.2014.11.021.

4. Burton JN, Adey A, Patwardhan RP, Qiu R, Kitzman JO and Shendure J. Chromosome-scale scaffolding of de novo genome assemblies based on chromatin interactions. Nature Biotechnology.Nat Biotechnol. 2013;31 12:1119-25. doi:10.1038/nbt.2727.

55. Marie-Nelly H, Marbouty M, Cournac A, Flot JF, Liti G, Parodi DP, et al. High-quality genome (re)assembly using chromosomal contact data. Nat Commun. 2014;5 1:5695. doi:10.1038/ncomms6695.

6. Kaplan N and Dekker J. High-throughput genome scaffolding from in vivo DNA interaction frequency. Nat Biotechnol. 2013;31 12:1143-7. doi:10.1038/nbt.2768.

7. Sedlazeck FJ, Lee H, Darby CA and Schatz MC. Piercing the dark matter: bioinformatics of long-range sequencing and mapping. Nature Reviews Genetics.Nat Rev Genet. 2018;19 6:329-46. doi:10.1038/s41576-018-0003-4.

6. Bickhart DM, Rosen BD, Koren S, Sayre BL, Hastie AR, Chan S, et al. Single-molecule sequencing and chromatin conformation capture enable de novo reference assembly of the domestic goat genome. Nature Genetics. 2017;49:643.

doi:10.1038/ng.3802.

78. Putnam NH, O'Connell BL, Stites JC, Rice BJ, Blanchette M, Calef R, et al. Chromosome-scale shotgun assembly using an in vitro method for long-range linkage. Genome ResearchRes. 2016;-26 3:342-50. doi:10.1101/gr.193474.115.

89. Ghurye J, Pop M, Koren S, Bickhart D and Chin C-SCS. Scaffolding of long read assemblies using long range contact information. BMC Genomics. 2017;18 1:527. doi:10.1186/s12864-017-3879-z.

910. Ghurye J, Rhie A, Walenz BP, Schmitt A, Selvaraj S, Pop M, et al. Integrating Hi-C links with assembly graphs for chromosome-scale assembly. bioRxiv. 2018:261149. doi:10.1101/261149PLoS Comput Biol. 2019;15 8:e1007273. doi:10.1371/journal.pcbi.1007273.

1011. Dudchenko O, Batra SS, Omer AD, Nyquist SK, Hoeger M, Durand NC, et al. De novo assembly of the Aedes aegypti genome using Hi-C yields chromosome-length scaffolds. Science. 2017;356 6333:92-5. doi:10.1126/science.aal3327.

1112. Ghurye J and Pop M. Modern technologies and algorithms for scaffolding assembled genomes. PLoS Comput Biol. 2019;15 6:e1006994. doi:10.1371/journal.pcbi.1006994.

13. Lewin HA, Robinson GE, Kress WJ, Baker WJ, Coddington J, Crandall KA, et al.

Earth BioGenome Project: Sequencing life for the future of life. Proceedings of the National Academy of Sciences of the United States of America.Proc Natl Acad Sci USA. 2018;115 17:4325-33. doi:10.1073/pnas.1720115115.

1214. Koepfli KP, Paten B, Genome KCoS and O'Brien SJ. The Genome 10K Project: a way forward. Annual review of animal biosciences.Annu Rev Anim Biosci. 2015;3:57-111. doi:10.1146/annurev-animal-090414-014900.

13. Editorial. A reference standard for genome biology. Nature Biotechnology. 2018;36:1121. doi:10.1038/nbt.4318.

1415. Dudchenko O, Shamim MS, Batra SS, Durand NC, Musial NT, Mostofa R, et al. The Juicebox Assembly Tools module facilitates de novo assembly of mammalian genomes with chromosome-length scaffolds for under $1000. bioRxiv. 2018:254797. doi:10.1101/254797.

1516. Belaghzal H, Dekker J and Gibcus JH. Hi-C 2.0: An optimized Hi-C procedure for high-resolution genome-wide mapping of chromosome conformation. Methods (San Diego, Calif).. 2017;123:56-65. doi:10.1016/j.ymeth.2017.04.004.

1617. Kuratani S, Kuraku S and Nagashima H. Evolutionary developmental perspective for the origin of turtles: the folding theory for the shell based on the developmental nature of the carapacial ridge. Evolution & Development.Evol Dev.

2011;13 1:1-14. doi:10.1111/j.1525-142X.2010.00451.x.

~~17~~18. Matsuda Y, Nishida-Umehara C, Tarui H, Kuroiwa A, Yamada K, Isobe T, et al. Highly conserved linkage homology between birds and turtles: bird and turtle chromosomes are precise counterparts of each other. ~~Chromosome research : an international journal on the molecular, supramolecular and evolutionary aspects of chromosome biology.~~Chromosome Res. 2005;13 6:601-15. doi:10.1007/s10577-005-0986-5.

~~18~~19. Kuraku S, Ishijima J, Nishida-Umehara C, Agata K, Kuratani S and Matsuda Y. cDNA-based gene mapping and GC3 profiling in the soft-shelled turtle suggest a chromosomal size-dependent GC bias shared by sauropsids. ~~Chromosome research : an international journal on the molecular, supramolecular and evolutionary aspects of chromosome biology.~~Chromosome Res. 2006;14 2:187-202. doi:10.1007/s10577-006-1035-8.

~~19~~20. Uno Y, Nishida C, Tarui H, Ishishita S, Takagi C, Nishimura O, et al. Inference of the protokaryotypes of amniotes and tetrapods and the evolutionary processes of microchromosomes from comparative gene mapping. ~~PloS one~~PLoS One. 2012;7 12:e53027. doi:10.1371/journal.pone.0053027.

~~20~~21. Kawai A, Nishida-Umehara C, Ishijima J, Tsuda Y, Ota H and Matsuda Y.

Different origins of bird and reptile sex chromosomes inferred from comparative mapping of chicken Z-linked genes. Cytogenet Genome Res. 2007;117 1-4:92-102. doi:10.1159/000103169.

22. Kawagoshi T, Uno Y, Matsubara K, Matsuda Y and Nishida C. The ZW micro-sex chromosomes of the Chinese soft-shelled turtle (Pelodiscus sinensis, Trionychidae, Testudines) have the same origin as chicken chromosome 15. Cytogenet Genome Res. 2009;125 2:125-31. doi:10.1159/000227837.

23. Wang Z, Pascual-Anaya J, Zadissa A, Li W, Niimura Y, Huang Z, et al. The draft genomes of soft-shell turtle and green sea turtle yield insights into the development and evolution of the turtle-specific body plan. ~~Nature Genetics.~~Nat Genet. 2013;45 6:701-6. doi:10.1038/ng.2615.

~~21~~24. Belton JM, McCord RP, Gibcus JH, Naumova N, Zhan Y and Dekker J. Hi-C: a comprehensive technique to capture the conformation of genomes. Methods. 2012;58 3:268-76. doi:10.1016/j.ymeth.2012.05.001.

~~22~~25. Servant N, Varoquaux N, Lajoie BR, Viara E, Chen CJ, Vert JP, et al. HiC-Pro: an optimized and flexible pipeline for Hi-C data processing. Genome Biol. 2015;16:259. doi:10.1186/s13059-015-0831-x.

~~23~~26. Sofueva S, Yaffe E, Chan WC, Georgopoulou D, Vietri Rudan M, Mira-

Bontenbal H, et al. Cohesin-mediated interactions organize chromosomal domain architecture. The EMBO journal.Embo j. 2013;32 24:3119-29. doi:10.1038/emboj.2013.237.

2427. Mayjonade B, Gouzy J, Donnadieu C, Pouilly N, Marande W, Callot C, et al. Extraction of high-molecular-weight genomic DNA for long-read sequencing of single molecules. BioTechniquesBiotechniques. 2016;61 4:203-5. doi:10.2144/000114460.

2528. Tanegashima C, Nishimura O, Motone F, Tatsumi K, Kadota M and Kuraku S. Embryonic transcriptome sequencing of the ocellate spot skate Okamejei kenojei. Scientific data.Sci Data. 2018;5:180200. doi:10.1038/sdata.2018.200.

2629. DeMaere MZ and Darling AE. bin3C: exploiting Hi-C sequencing data to accurately resolve metagenome-assembled genomes. Genome Biol. 2019;20 1:46. doi:10.1186/s13059-019-1643-1.

30. Botero-Castro F, Figuet E, Tilak MK, Nabholz B and Galtier N. Avian Genomes Revisited: Hidden Genes Uncovered and the Rates versus Traits Paradox in Birds. Molecular biology and evolution.Mol Biol Evol. 2017;34 12:3123-31. doi:10.1093/molbev/msx236.

2731. Hotaling S and Kelley JL. The rising tide of high-quality genomic resources.

~~Molecular Ecology Resources.~~Mol Ecol Resour. 2019;19 3:567-9. doi:10.1111/1755-0998.12964.

~~28~~32. Nishimura O, Hara Y and Kuraku S. gVolante for standardizing completeness assessment of genome and transcriptome assemblies. Bioinformatics~~ (Oxford, England).~~. 2017;33 22:3635-7. doi:10.1093/bioinformatics/btx445.

~~29~~33. Roach MJ, Schmidt SA and Borneman AR. Purge Haplotigs: allelic contig reassignment for third-gen diploid genome assemblies. BMC Bioinformatics. 2018;19 1:460. doi:10.1186/s12859-018-2485-7.

~~30~~34. Kadota M, Hara Y, Tanaka K, Takagi W, Tanegashima C, Nishimura O, et al. CTCF binding landscape in jawless fish with reference to Hox cluster evolution. ~~Scientific Reports.~~Sci Rep. 2017;7 1:4957. doi:10.1038/s41598-017-04506-x.

~~31. Miura H, Takahashi S, Poonperm R, Tanigawa A, Takebayashi S and Hiratani I. Spatiotemporal developmental dynamics of chromosome organization revealed by single-cell DNA replication profiling. in press.~~

~~32~~35. Ikeda T, Hikichi T, Miura H, Shibata H, Mitsunaga K, Yamada Y, et al. Srf destabilizes cellular identity by suppressing cell-type-specific gene expression programs. Nat Commun. 2018;9 1:1387. doi:10.1038/s41467-018-03748-1.

36. Adey A, Morrison HG, Asan, Xun X, Kitzman JO, Turner EH, et al. Rapid, low-

input, low-bias construction of shotgun fragment libraries by high-density in vitro transposition. Genome ~~Biology~~Biol. 2010;11 12:R119. doi:10.1186/gb-2010-11-12-r119.

~~33~~37. Langmead B and Salzberg SL. Fast gapped-read alignment with Bowtie 2. ~~Nature~~Nat Methods. 2012;9 4:357-9. doi:10.1038/nmeth.1923.

~~34~~38. Durand NC, Shamim MS, Machol I, Rao SS, Huntley MH, Lander ES, et al. Juicer Provides a One-Click System for Analyzing Loop-Resolution Hi-C Experiments. Cell ~~systems~~Syst. 2016;3 1:95-8. doi:10.1016/j.cels.2016.07.002.

~~35~~39. Li H and Durbin R. Fast and accurate short read alignment with Burrows-Wheeler transform. Bioinformatics~~ (Oxford, England).~~. 2009;25 14:1754-60. doi:10.1093/bioinformatics/btp324.

~~36~~40. Li H. A statistical framework for SNP calling, mutation discovery, association mapping and population genetical parameter estimation from sequencing data. Bioinformatics~~ (Oxford, England).~~. 2011;27 21:2987-93. doi:10.1093/bioinformatics/btr509.

~~37~~41. Quinlan AR and Hall IM. BEDTools: a flexible suite of utilities for comparing genomic features. Bioinformatics~~ (Oxford, England).~~. 2010;26 6:841-2. doi:10.1093/bioinformatics/btq033.

~~38~~42. Simao FA, Waterhouse RM, Ioannidis P, Kriventseva EV and Zdobnov EM. BUSCO: assessing genome assembly and annotation completeness with single-copy orthologs. Bioinformatics ~~(Oxford, England).~~. 2015;31 19:3210-2. doi:10.1093/bioinformatics/btv351.

~~39~~43. Grabherr MG, Haas BJ, Yassour M, Levin JZ, Thompson DA, Amit I, et al. Full-length transcriptome assembly from RNA-Seq data without a reference genome. Nat Biotechnol. 2011;29 7:644-52. doi:10.1038/nbt.1883.

~~40~~44. Wang M and Kong L. pblat: a multithread blat algorithm speeding up aligning sequences to genomes. BMC Bioinformatics. 2019;20 1:28. doi:10.1186/s12859-019-2597-8.

~~41~~45. Ryan JF. Baa.pl: A tool to evaluate de novo genome assemblies with RNA transcripts. arXiv e-prints. 2013;arXiv:1309.2087.

~~42~~46. Kent WJ. BLAT--the BLAST-like alignment tool. Genome Res. 2002;12 4:656-64. doi:10.1101/gr.229202.

~~43~~47. Imakaev M, Fudenberg G, McCord RP, Naumova N, Goloborodko A, Lajoie BR, et al. Iterative correction of Hi-C data reveals hallmarks of chromosome organization. ~~Nature~~Nat Methods. 2012;9 10:999-1003. doi:10.1038/nmeth.2148.

Table 1: Overview of the specification of ~~the~~major scaffolding programs~~ released to date~~.

**Program**	**Support and availability**	**Input data requirement**	**Other information**	**Literature**
LACHESIS	Developer's support discontinued; intricate installation	Generic bam format	No function to correct scaffold misjoins	[4]
HiRise	Open source version at GitHub not updated since 2015	Generic bam format	Employed in Dovetail Chicago/Hi-C service. Default input sequence length ~~cutoff~~cut-off=1000 bp	[~~7~~8]
3d-dna	Actively maintained and supported by the developer	Not compatible with multiple enzymes; Accept only Juicer mapper format	Default parameters: -t 15000 (input sequence length ~~cutoff~~cut-off), -r 2 (no. of iterations for misjoin correction)	[~~10, 34~~11, 38]
SALSA2	Actively maintained and supported by the developer	Compatible with multiple enzymes; generic bam (bed) file, assembly graph, unitig, 10x link files	Default parameters: -c 1000 (input sequence length ~~cutoff~~cut-off), -i 3 (no. of iterations for misjoin correction)	[~~8, ~~9, 10]

Figures

A

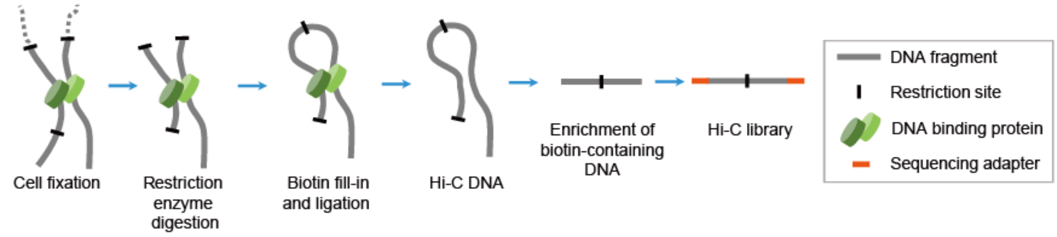

B

Different specifications	iconHi-C (Our protocol)	Arima-HiC Kit (Animal ver. A160132 v00, with the KAPA Hyper Prep Kit)	Proximo Hi-C Kit (Animal ver. 1.5)	Dovetail Hi-C Kit (ver. 1.03)
Cell fixation	10 min (cells) or 15 min (tissue) in 1 % formaldehyde at 25°C; up to 1×10^7 cells or up to 1 cm^3 tissue	10 min (cells) or 20 min (tissue) in 2 % formaldehyde at RT; 0.5-1 ×10^7 cells or 100-500 mg tissue	15 min in crosslinking solution (included in the kit); 200 mg tissue	20 min in 1.5 % formaldehyde at RT; 0.5×10^6 cells and 20-40 mg tissue
Sample amount for Hi-C reaction	1-2×10^6 cells or tissue estimated to contain 2-10 µg DNA	Cells or tissue estimated to contain 750 ng to 5 µg DNA	Up to 1 µg DNA based on the DNA quantification performed after restriction digestion	Cells or tissue estimated to contain 50-500 ng DNA
Restriction enzyme digestion	HindIII (cuts at "AAGCTT") or DpnII (cuts at "GATC"), 16 hrs at 37°C	Cocktail of A1 and A2 enzymes (cuts at "GATC" and "GANTC"), 30-60 min at 37°C	Sau3AI (cuts at "GATC"), 1 hr at 37°C	DpnII (cuts at "GATC"), 1 hr at 37°C
Ligation	6 hrs at 16°C	15 min at RT	4 hrs at RT	1-16 hrs at 16°C
Reverse crosslinking	16 hrs at 65°C	90 min at 68°C	1-16 hrs at 60°C	45 min at 68°C
Hi-C DNA extraction	Phenol/chloroform extraction	DNA purification beads (e.g. AMPure XP)	Spin column (included in the kit)	AMPure XP
Hi-C DNA QC	Check for the size shift before and after ligation (QC1)	Check for the yield of biotin-labeled DNA (Arima-QC1)	Check for the yield of bead-bound DNA	Check for the DNA yield
DNA amount for library preparation	250 ng - 2 µg	125 ng - 2 µg	No more than 50 ng DNA	200 ng
Removal of biotin from un-ligated DNA ends	By T4 DNA polymerase	N/A	N/A	N/A
DNA fragmentation	Sonication (Covaris)	Sonication (Covaris or Diagenode)	Transposase	Sonication (Covaris)
Library preparation	Adapter ligation-based (KAPA LTP Library Prep Kit)	Adapter ligation-based	Transposase-based (included in the kit)	Adapter ligation-based (included in the Hi-C kit)
PCR cycles	Pre-determination by qPCR (KAPA Real-time Library Amplification Kit)	Pre-determination by qPCR (KAPA Library Quantification Kit; Arima-QC2)	15 cycles	11 cycles
Size selection	After DNA fragmentation	After DNA fragmentation	After PCR	After PCR
Hi-C library QC	Check for yield and size distribution; check for size shift by NheI or ClaI digestion (QC2)	Check for yield and size distribution	Check for yield and size distribution	Check for yield and size distribution

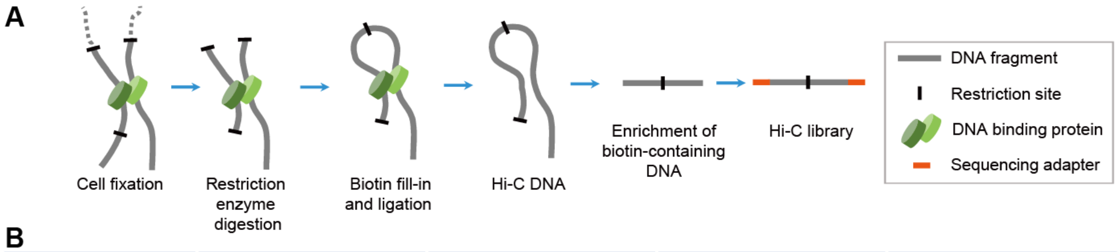

B

Different specifications	iconHi-C (Our protocol)	Arima-HiC Kit (ver. A160107 v00, with the KAPA Hyper Prep Kit)	Phase Proximo Hi-C Kit (Animal ver. 1.0)	Dovetail Hi-C Kit (ver. 1.4, with Dovetail Library Module and Primer Set)
Cell fixation	10 min (cells) or 15 min (tissue) in 1 % formaldehyde at 25°C; up to 1×10^7 cells or up to 1 cm^3 tissue	10 min (cells) or 20 min (tissue) in 2 % formaldehyde at RT; 0.5-1 $\times10^7$ cells or 100-500 mg tissue	15 min in crosslinking solution (included in the kit) at RT; 1×10^7 cells or 100 mg tissue	20 min in 1.5 % formaldehyde at RT; 0.5×10^6 cells and 20-40 mg tissue
Sample amount for restriction digestion and ligation	1-2×10^6 cells or tissue estimated to contain 2-10 µg DNA	Cells or tissue estimated to contain 750 ng - 5 µg DNA	1×10^7 cells or 100 mg tissue	0.5×10^6 cells or 20-40 mg tissue
Restriction enzyme digestion	HindIII (cuts at "AAGCTT") or DpnII (cuts at "GATC"), 16 hrs at 37°C	Cocktail of A1 and A2 enzymes (cuts at "GATC" and "GANTC"), 30-60 min at 37°C	Sau3AI (cuts at "GATC"), 1 hr at 37°C	DpnII (cuts at "GATC"), 1 hr at 37°C
Ligation	6 hrs at 16°C	15 min at RT	4 hrs at RT	1-16 hrs at 16°C
Reverse crosslinking	16 hrs at 65°C	1.5-16 hrs at 68°C	1-16 hrs at 60°C	45 min at 68°C
Hi-C DNA extraction	Phenol/chloroform extraction	DNA purification beads (e.g. AMPure XP)	Spin column (included in the kit)	SPRIselect beads
Hi-C DNA QC	Check for the size shift before and after ligation (QC1)	Check for the yield of biotin-labeled DNA (Arima-QC1)	Check for the DNA yield before proximity ligation	Check for the DNA yield
DNA amount for library preparation	250 ng - 2 µg	125 ng - 2 µg	N/A	200 ng
Removal of biotin from un-ligated DNA ends	By T4 DNA polymerase	N/A	N/A	N/A
DNA fragmentation	Sonication (Covaris)	Sonication (Covaris or Diagenode)	Transposase	Sonication (Covaris or Diagenode)
Library preparation	Adapter ligation-based (KAPA LTP Library Prep Kit)	Adapter ligation-based	Transposase-based (included in the kit)	Adapter ligation-based
PCR cycles	Pre-determination by qPCR (KAPA Real-time Library Amplification Kit)	Pre-determination by qPCR (KAPA Library Quantification Kit; Arima-QC2)	15 cycles	11 cycles
Size selection	After DNA fragmentation	After DNA fragmentation	After PCR	After PCR
Hi-C library QC	Check for yield and size distribution; check for size shift by NheI or ClaI digestion (QC2)	Check for yield and size distribution	Check for yield and size distribution	Check for yield and size distribution

Figure 1: Hi-C library preparation. (A) Basic procedure. (B) Comparison of Hi-C library preparation methods. ~~Included here are only~~Only the major differences between the methods are included here. The versions of the Arima and Phase kits used in this study are presented. The KAPA Hyper Prep Kit (KAPA Biosystems) is assumed to be conjunctly used with Arima Hi-C Kit, among the several specified kits. See Supplementary Protocol S1 for the full version of the iconHi-C protocol which was derived from the ~~protocol~~protocols published previously ~~introduced [23~~[3, 26, 35].

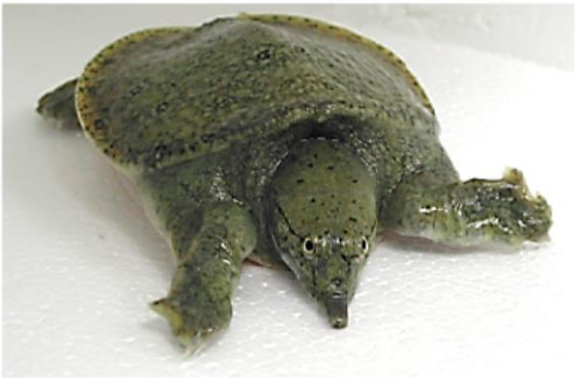

Figure 2: A juvenile softshell turtle *Pelodiscus sinensis*.

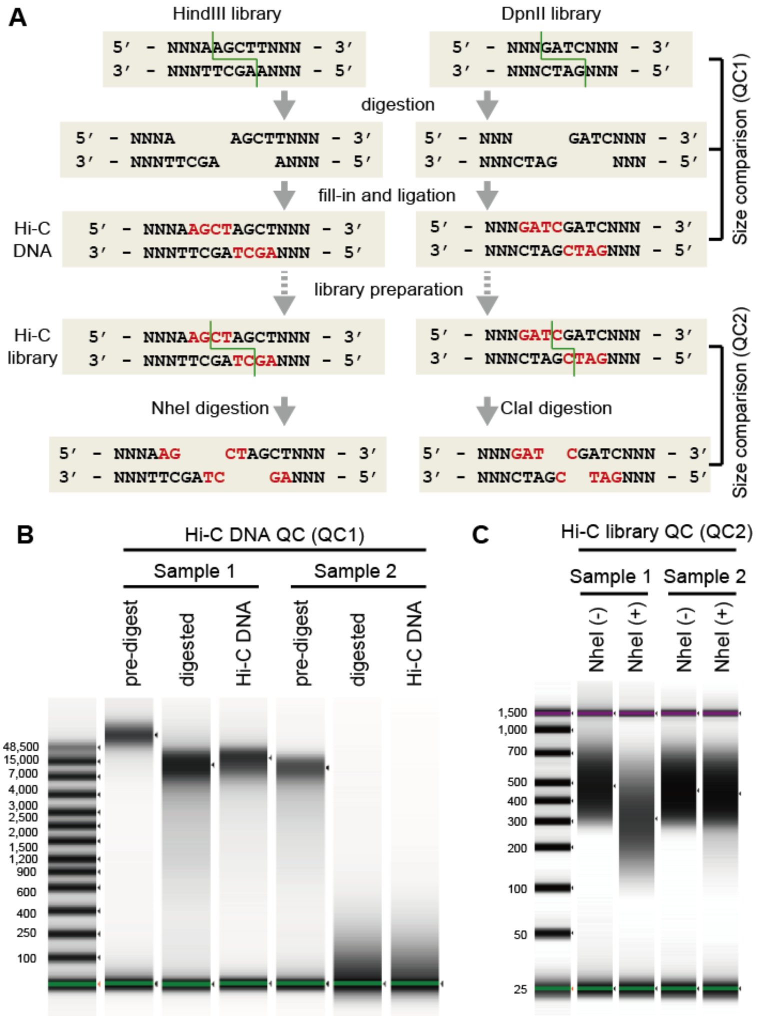

Figure 3: Structure of the Hi-C DNA and principle of the quality controls. (A) Schematic representation of the library preparation workflow based on HindIII or DpnII digestion. ~~Patterns~~The patterns of restriction are indicated by the green lines. ~~Nucleotides~~The nucleotides that ~~were~~are filled in are indicated by the letters in red. (B) Size shift analysis of HindIII-digested Hi-C DNA (QC1). ~~Shown are the representative~~Representative images of qualified (Sample 1) and disqualified ~~samples~~ (Sample 2~~).~~) samples are shown. (C) Size shift analysis of the HindIII-digested Hi-C

library (QC2). Shown are the representativeRepresentative images of the qualified (Sample 1) and disqualified (Sample 2) samples are shown. Size distributions were measured with Agilent 4200 TapeStation.

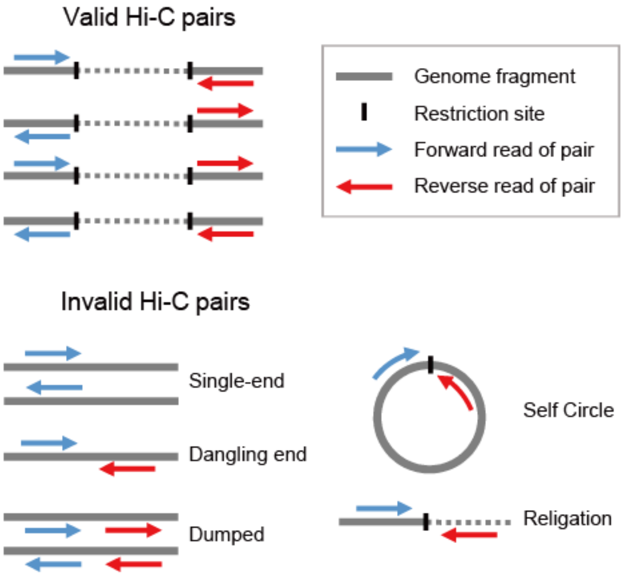

Figure 4: Post-sequencing quality control of Hi-C reads. Read pairs were categorized into valid and invalid pairs by HiC-Pro, based on their status in the mapping to the reference genome (see Methods). This figure was adapted from the ~~literature~~ article that described HiC-Pro originally ~~introducing HiC-Pro [22].~~ [25].

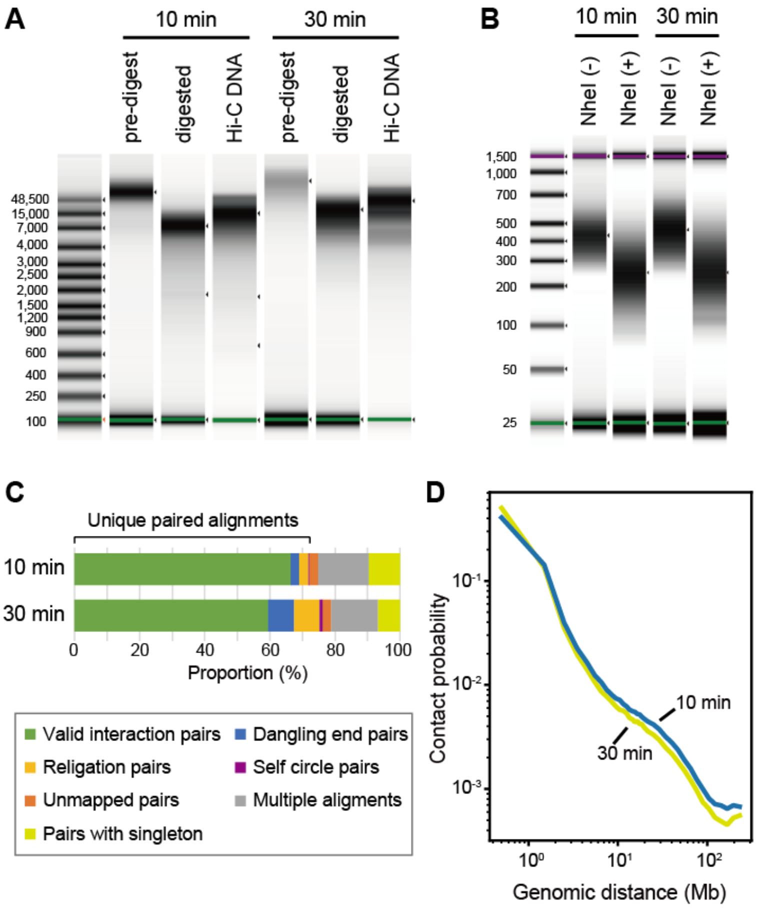

Figure 5: Effect of cell fixation duration. (A) QC1 of the HindIII-digested Hi-C DNA of human GM12878 cells fixed for 10 or 30 minutes in 1% formaldehyde. (B) QC2 of the HindIII-digested library of human GM12878 cells. (C) Quality control of the sequence reads by HiC-Pro using ~~1M~~1 M read pairs. See Fig. 4 for the details of the read pair categorization. See Supplementary Table ~~S7~~S3 for the actual proportion of the reads in each category. (D) Contact probability measured by the ratio of observed and expected frequencies of Hi-C read pairs mapped along the same chromosome [~~43~~47].

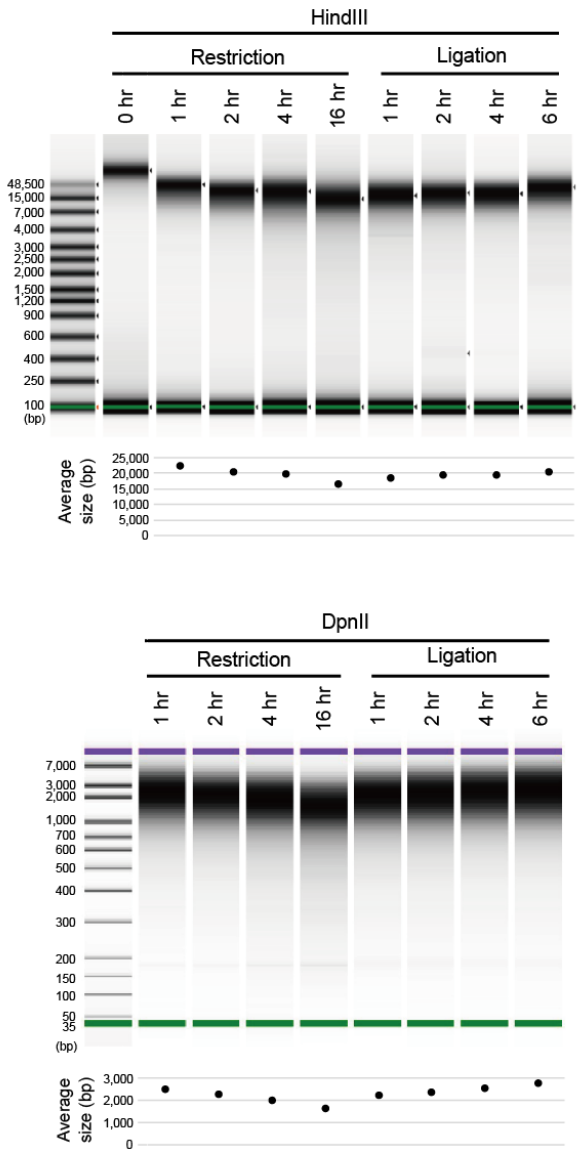

Figure 6: Testing ~~variable~~varying durations of restriction and ligation~~ of Hi-C DNA. Length~~. The length distributions of the DNA molecules prepared from human GM12878 cells after ~~variable durations of~~ restriction and ligation of variable duration are shown. ~~Size distribution for~~The size distributions of the HindIII-digested samples (top) and DpnII-digested samples (bottom) were measured ~~by~~with an Agilent 4200 TapeStation and an Agilent Bioanalyzer, respectively.

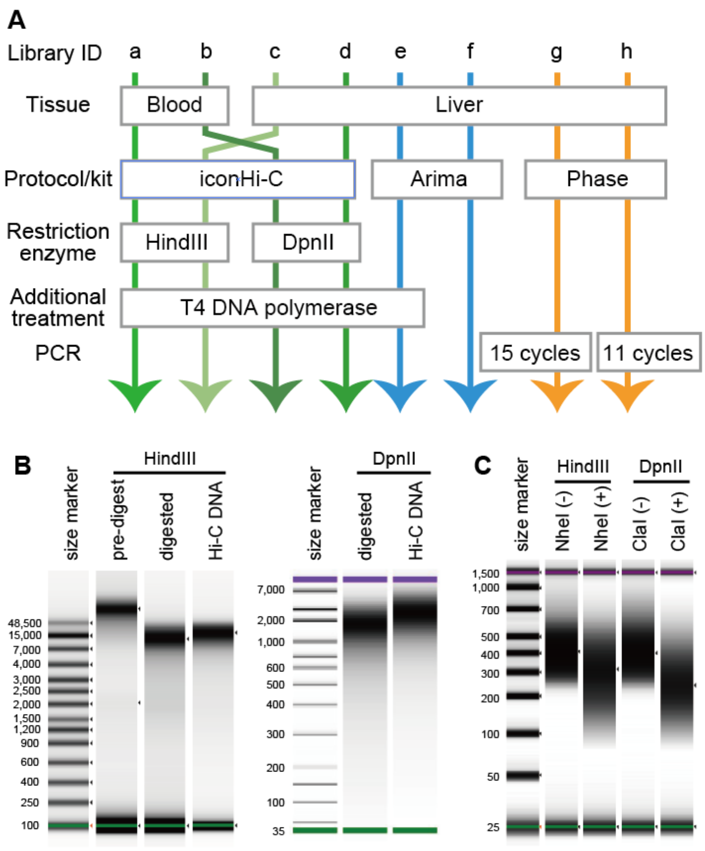

Figure 7: Softshell turtle Hi-C libraries prepared for our methodological comparison. (A) Lineup of the prepared libraries. This chart includes only the conditions ~~that varied~~in preparation methods that varied between these libraries, and the ~~rest of the~~remainder preparation workflows are described in Supplementary Protocol S1 for the non-commercial ('iconHi-C') protocol and in the manuals of the commercial kits. (B) Quality control of Hi-C DNA (QC1) for Library c and d. The ~~prepared~~ Hi-C DNA for the Chinese softshell turtle liver ~~samples were digested~~ sample was prepared with either HindIII or DpnII digestion. (C) Quality control of Hi-C libraries (QC2). The HindIII library prepared from the softshell turtle liver ~~HindIII library~~ was digested by NheI, and the DpnII library was digested by ClaI (see Fig. 3 for the technical principle). See

Supplementary Fig. S3S2 for the QC1 and QC2 results forof the samples prepared from the blood of this species. See Supplementary Fig. S3 for the QC2 result of the Phase libraries.

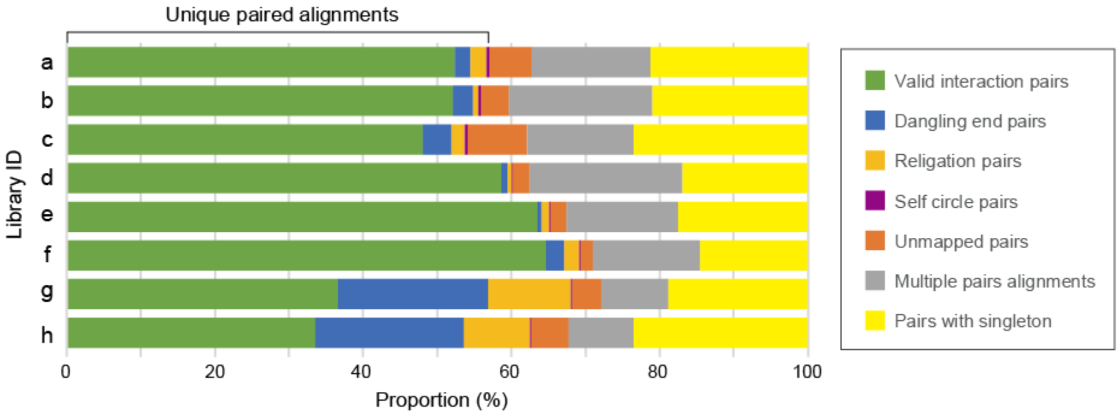

Figure 8: Results of the post-sequencing quality control with HiC-Pro. One million read pairs were used for computation with HiC-Pro. See Fig. 7A for the preparation conditions of Library a-h, Fig. 4 for the categorization, and Supplementary Table S3S5 for the actual proportion of the reads in each category. PostThe post-sequencing quality control using variable read amounts (500 K– to 200 M pairs) for one of these softshell turtle libraries (Supplementary Table S6S9) and human GM12878 libraries (Supplementary Table S2) shows the validity of this quality control with as few as 500 K read pairs.

A

<table>
<tr><th>Assembly ID</th><th>Library ID</th><th>Scaffolding program</th><th>Input sequence length cutoff (nt)</th><th>Number of iterative misjoin correction rounds</th><th>Number of read pairs input</th></tr>
<tr><td>1</td><td>c</td><td rowspan="4">3d-dna</td><td rowspan="4">15000</td><td rowspan="4">2</td><td rowspan="18">200M</td></tr>
<tr><td>2</td><td>a</td></tr>
<tr><td>3</td><td>d</td></tr>
<tr><td>4</td><td>b</td></tr>
<tr><td>5</td><td>c</td><td rowspan="2">SALSA2</td><td rowspan="2">1000</td><td rowspan="2">3</td></tr>
<tr><td>6</td><td>d</td></tr>
<tr><td>7</td><td>c + d</td><td rowspan="3">3d-dna</td><td rowspan="3">15000</td><td rowspan="3">2</td></tr>
<tr><td>8</td><td>b + d</td></tr>
<tr><td>9</td><td rowspan="2">e</td></tr>
<tr><td>10</td><td>SALSA2</td><td>1000</td><td>3</td></tr>
<tr><td>11</td><td rowspan="2">h</td><td>3d-dna</td><td>15000</td><td>2</td></tr>
<tr><td>12</td><td>SALSA2</td><td>1000</td><td>3</td></tr>
<tr><td>13</td><td rowspan="11">d</td><td rowspan="11">3d-dna</td><td rowspan="3">15000</td><td>2</td></tr>
<tr><td>14</td><td>4</td></tr>
<tr><td>15</td><td>6</td></tr>
<tr><td>16</td><td>10000</td><td rowspan="8">2</td></tr>
<tr><td>17</td><td>5000</td></tr>
<tr><td>18</td><td>3000</td></tr>
<tr><td>19</td><td rowspan="5">15000</td><td>280M</td></tr>
<tr><td>20</td><td>160M</td></tr>
<tr><td>21</td><td>80M</td></tr>
<tr><td>22</td><td>20M</td></tr>
<tr><td>23</td><td>10M</td></tr>
</table>

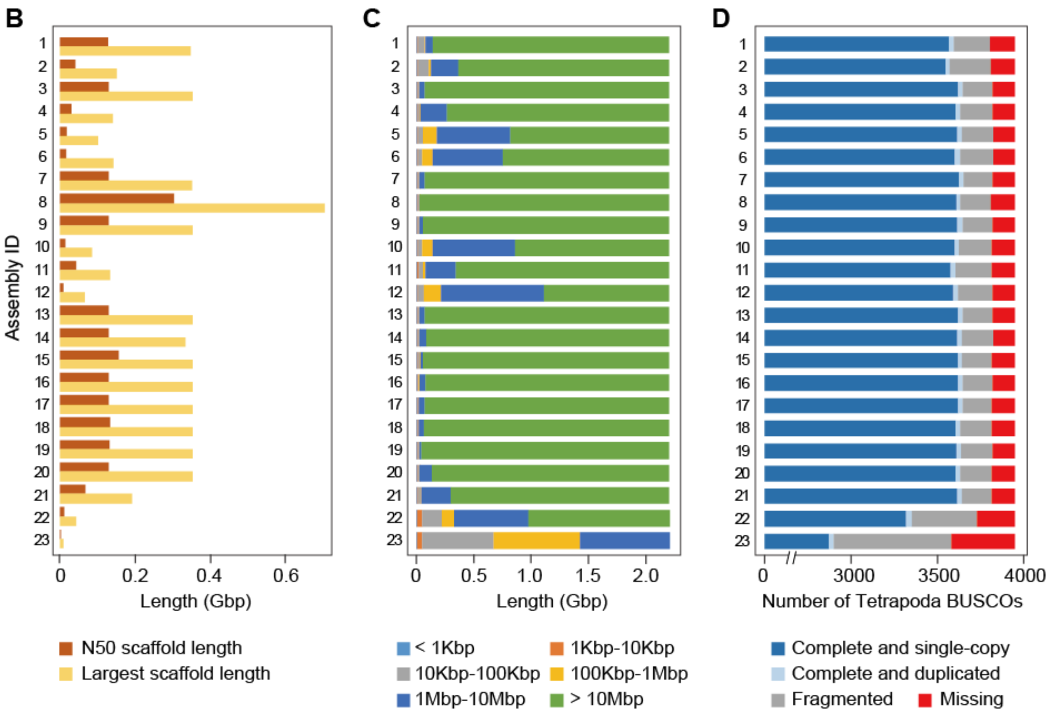

A

Assembly ID	Library ID	Scaffolding program	Input sequence length cutoff (nt)	Number of iterative misjoin correction rounds	Number of read pairs input
1	c	3d-dna	15000	2	200 M
2	a				
3	d				
4	b				
5	c	SALSA2	1000	3	
6	d				
7	c + d	3d-dna	15000	2	
8	b + d				
9	e				
10		SALSA2	1000	3	
11	h	3d-dna	15000	2	
12		SALSA2	1000	3	
13	d	3d-dna	15000	4	
14				6	
15			10000	2	
16			5000		
17			3000		
18			15000		280 M
19					160 M
20					80 M
21					20 M
22					10 M

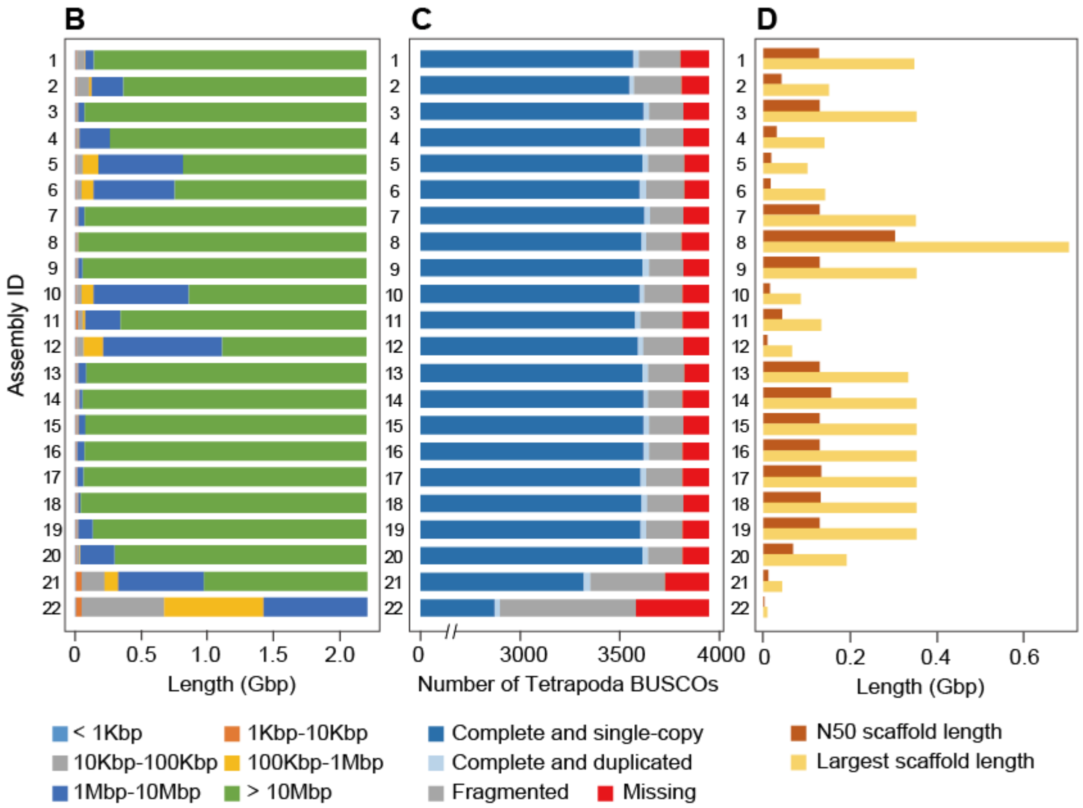

Figure 9: Comparison of Hi-C scaffolding products. (A) Scaffolding conditions used to produce Assembly 1 to ~~23. Default~~22. The default parameters are shown ~~with~~in red

letters. (B) Total and N50 scaffold lengths. (C) Scaffold length distributions. (DC) Gene space completeness. (D) Largest and N50 scaffold lengths. See the panel A for Library IDs and Supplementary Table S4S6 for raw values of the metrics shown in B–D.

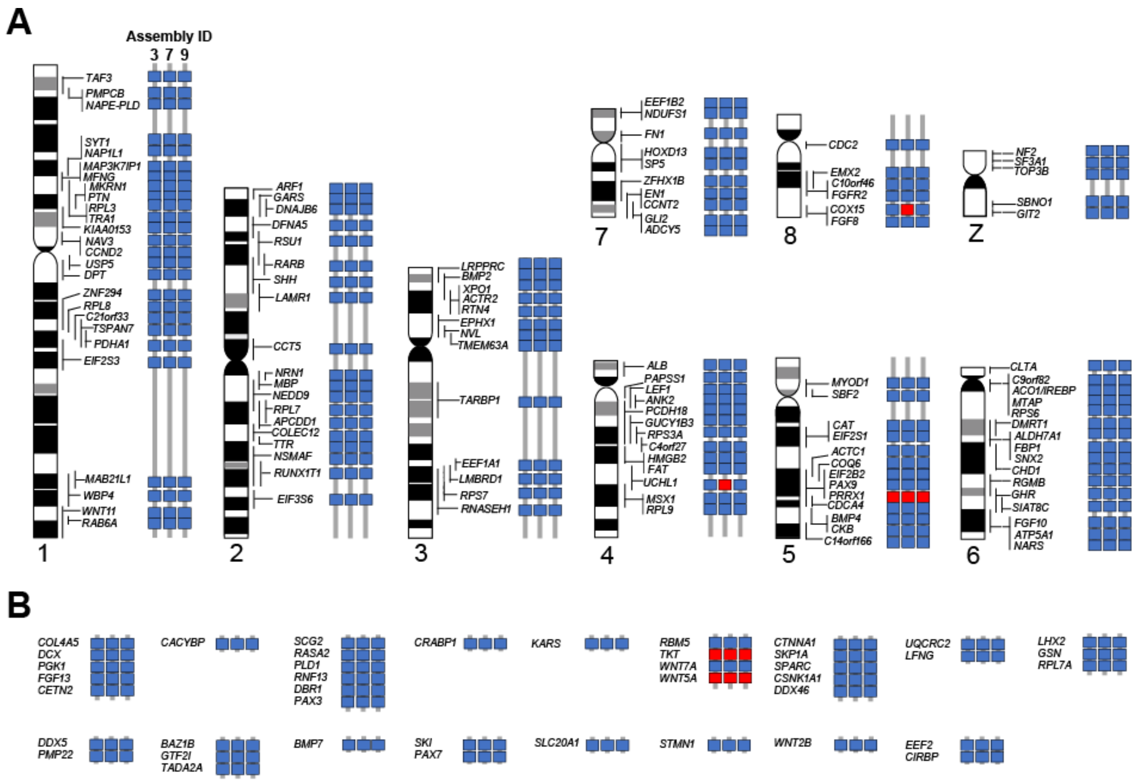

Figure 10: Cytogenetic validation of Hi-C scaffolding results. ~~On~~For the scaffolded sequences of Assembly 3, 7, and 9, we evaluated the consistency of the positions of the selected genes that were previously localized on ~~8~~eight macrochromosomes and Z chromosome (A) and microchromosomes (B) by chromosome FISH [~~17-19~~18-22] (see Results). Concordant and discordant gene locations on individual assemblies are indicated with blue and red boxes, respectively. The arrays of genes without idiograms in B were identified on chromosomes that are cytogenetically indistinguishable from each other.

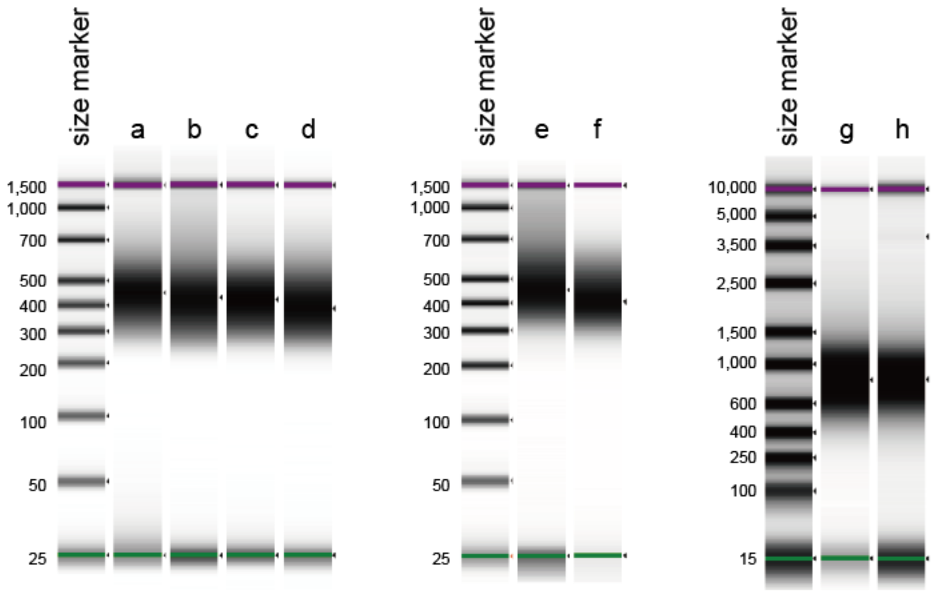

Supplementary Figure S1: DNA size distribution of the softshell turtle Hi-C libraries. ~~Size~~The size distribution of the libraries was ~~analyzed~~analysed by an Agilent 4200 TapeStation using the High Sensitivity D1000 kit for Library a-f and the High Sensitivity D5000 kit for Library g and h.

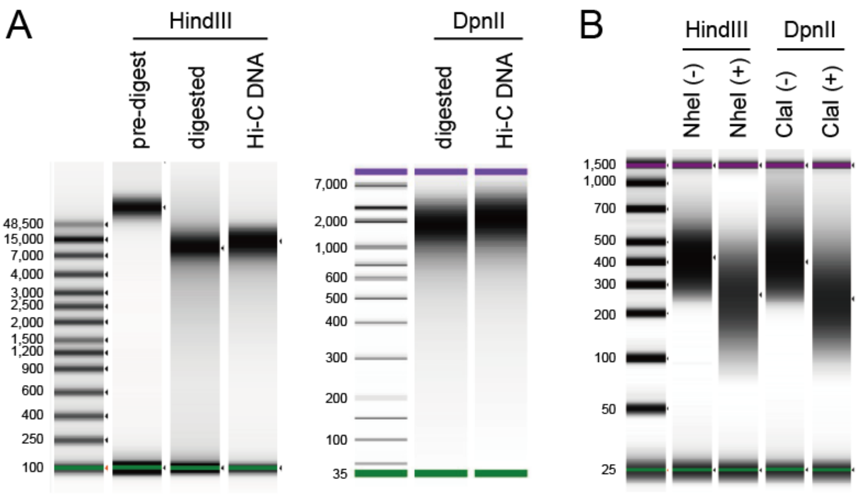

Supplementary Figure S2

~~Supplementary Figure S2: Structural analysis of the possibly overassembled~~: Pre-sequencing quality control of softshell turtle blood Hi-C libraries (Library a and b). (A) Quality control of Hi-C DNAs (QC1). Hi-C DNA was prepared from the Chinese softshell turtle blood by HindIII or DpnII digestion (see Fig. 7A for the details). (B) Quality control of Hi-C libraries (QC2). The softshell turtle blood library prepared using HindIII was digested by NheI, and the library prepared using DpnII was digested by ClaI (see Fig. 3 for the technical principle).

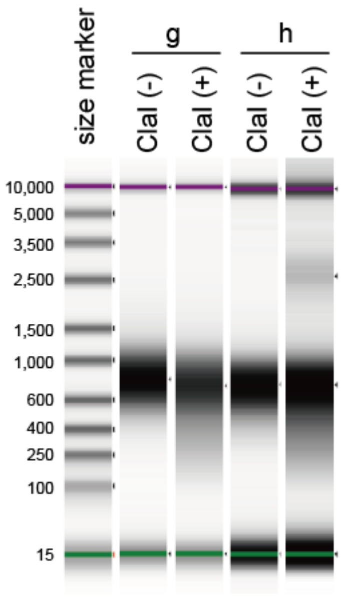

Supplementary Figure S3: Pre-sequencing quality control (QC2) of the Hi-C libraries prepared using the Phase kit (Library g and h). The softshell turtle liver libraries prepared using Sau3A1 were digested by ClaI.

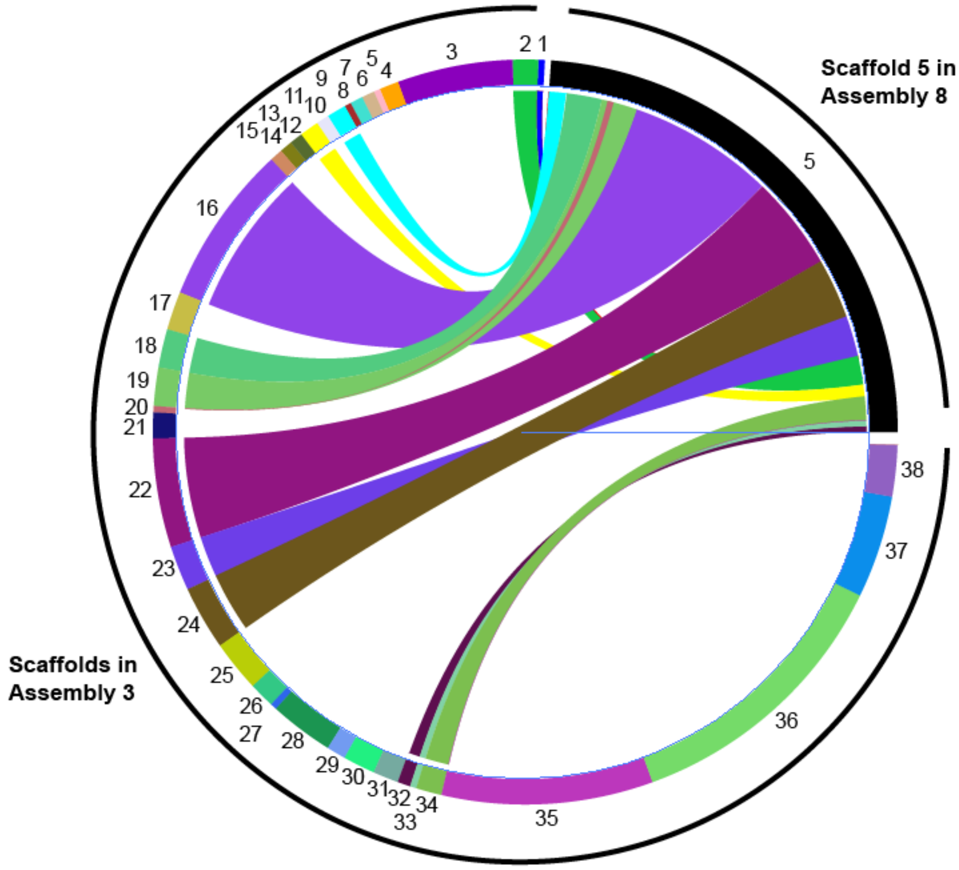

Supplementary Figure S4: Structural analysis of the possibly chimeric scaffold in Assembly 8. This figure shows the nucleotide sequence-level correspondence of the whole sequence of ~~the~~ scaffold 5 of Assembly 8 to 14 scaffolds of Assembly 3. Note that the scaffold 5 of Assembly 8 accounts for approximately one-third of the estimated genome size, and that some of the scaffolds of Assembly 3 in the figure have multiple high-similarity regions in ~~the~~ scaffold 5 of Assembly 8.

Assembly 3
(iconHi-C)

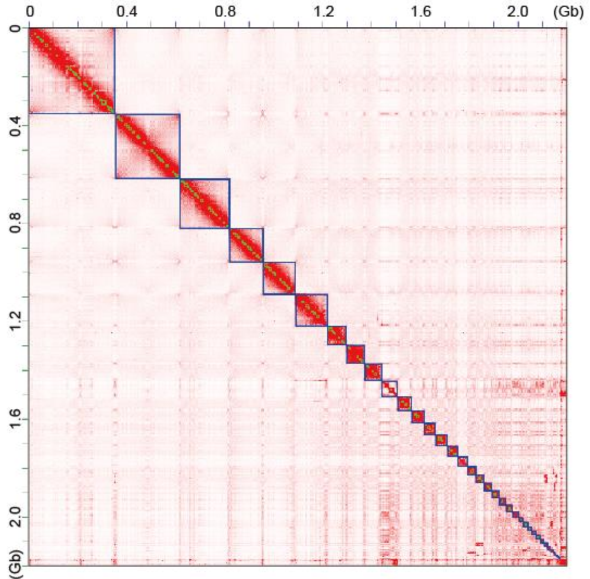

Assembly 9
(Arima)

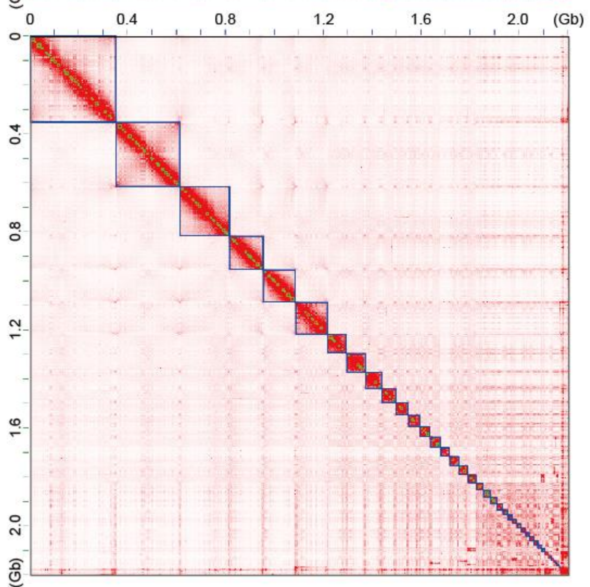

Assembly 11
(Phase)

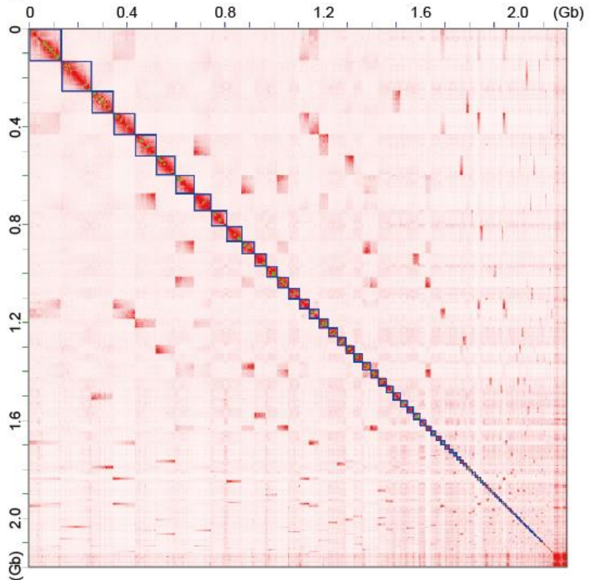

Supplementary Figure S5: Contact maps for selected softshell turtle Hi-C scaffolds. The blue squares are chromosomal units defined by 3d-dna, and the order of the scaffolds is sorted by their length. Assembly 11 exhibits the largest number of intensified blocks diverted from the diagonal line.

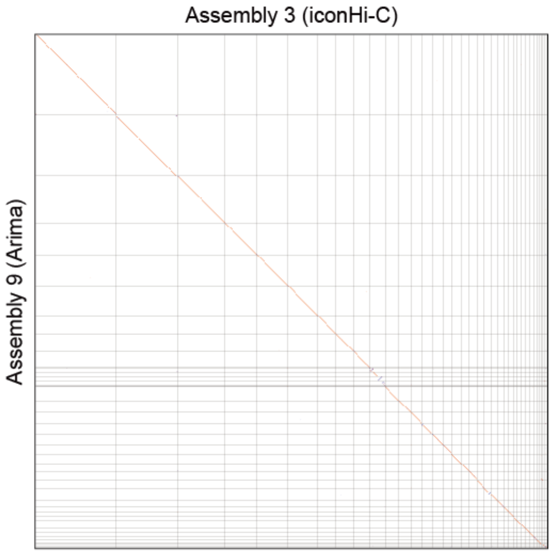
Assembly 3 (iconHi-C)
Assembly 9 (Arima)

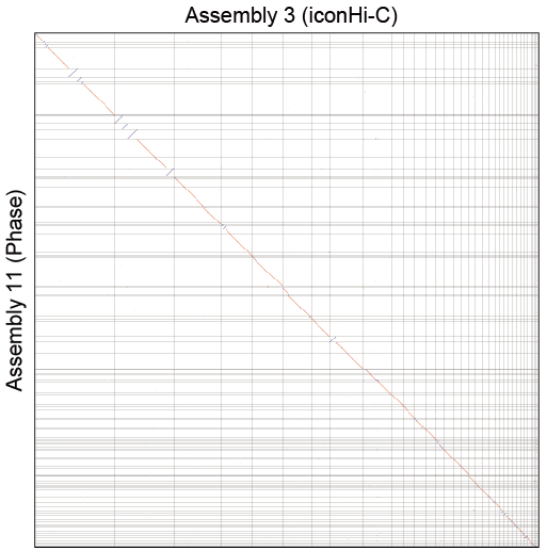
Assembly 3 (iconHi-C)
Assembly 11 (Phase)

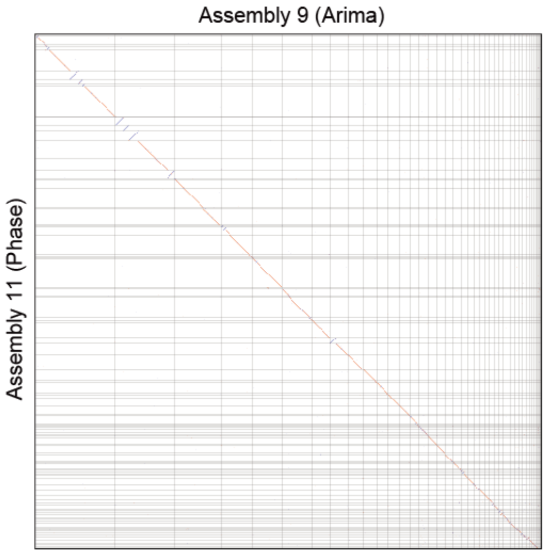
Assembly 9 (Arima)
Assembly 11 (Phase)

Supplementary Figure S6: Pairwise alignment of Hi-C scaffolds. Genome-wide alignments between the Hi-C scaffolds obtained were performed by LAST, and the dot plots were constructed using the last-dotplot script. Only scaffolds that were 1Mb or longer were included, and the order of the scaffolds along the X-axis was sorted by their length.

~~Supplementary Figure **S3:** Pre-sequencing quality control of softshell turtle blood Hi-C libraries (Library a and b). (A) Quality control of Hi-C DNAs (QC1). Hi-C DNA was prepared from the Chinese softshell turtle blood by HindIII or DpnII digestion (see Fig. 7A for the detail). (B) Quality control of Hi-C libraries (QC2). The prepared softshell turtle blood library employing HindIII was digested by NheI, and the one employing DpnII was digested by ClaI (see Fig. 3 for the technical principle).~~

Supplementary Table S1

Click here to access/download
Supplementary Material
Supplementary_TableS1_draft-genome.pdf

Supplementary Table S2

Click here to access/download
Supplementary Material
Supplementary_TableS2_GM12878-reduced-reads.pdf

Supplementary Table S3

Click here to access/download
Supplementary Material
Supplementary_TableS3_QC-GM-fix-time.pdf

Supplementary Table S4

Click here to access/download
Supplementary Material
Supplementary_TableS4_digestion_and_ligation_time.pd
f

Supplementary Table S5

Click here to access/download
Supplementary Material
Supplementary_TableS5_Ps_Lib_QC_1M-Mod.pdf

Supplementary Table S6

Click here to access/download
Supplementary Material
Supplementary_TableS6_all_scaffolding.pdf

Supplementary Table S7

Click here to access/download
Supplementary Material
Supplementary_TableS7_RNAassembly_mapping.pdf

Supplementary Table S8

Click here to access/download
Supplementary Material
Supplementary_TableS8_PCR_cycle.pdf

Supplementary Table S9

Click here to access/download
Supplementary Material
Supplementary_TableS9_Ps-reduced-reads_all-kit.pdf

Click here to access/download
Supplementary Material
Supplementary_Protocol_S1_rev2.pdf

Supplementary Protocol S2

Click here to access/download
Supplementary Material
Supplementary_Protocol_S2_to_support_multiple_enzymes.pdf

Personal Cover
Click here to access/download;Personal Cover;CoverLetter_iconHi-C_rev.pdf

Shigehiro Kuraku, Ph.D.
Team Leader
Laboratory for Phyloinformatics
RIKEN Center for Biosystems Dynamics Research (BDR)

Tel: +81-(0)-78-306-3331
Email: shigehiro.kuraku@riken.jp
URL: https://www.bdr.riken.jp/en/research/labs/kuraku-s/

October 21, 2019

GigaScience
Dear Editor,

Thank you very much for your handling our manuscript entitled, '***Multifaceted Hi-C benchmarking: what makes a difference in chromosome-scale genome scaffolding?***' by **Kadota, Nishimura, *et al.*** to be considered for publication in the journal *GigaScience*. We are very grateful for a number of constructive comments from the reviewers. Following the comments, we have revised the manuscript, which we believe consolidated our findings and led to a significant improvement of the manuscript. We hope that you will find our manuscript ready for publication in *GigaScience*.

Sincerely yours,

Shigehiro Kuraku, Ph.D.
