# Supplementary material for: Multifaceted Hi-C benchmarking: what makes a difference in chromosome-scale genome scaffolding?
Source: Gigascience. 2020 Jan 10;9(1):giz158. doi: 10.1093/gigascience/giz158 (PMC6952475; doi:10.1093/gigascience/giz158)
Supplement: giz158_GIGA-D-19-00211_Revision_2 [file giz158_giga-d-19-00211_revision_2.pdf]

GigaScience

# Multifaceted Hi-C benchmarking: what makes a difference in chromosome-scale genome scaffolding?

--Manuscript Draft--

| | |
|---|---|
| **Manuscript Number:** | GIGA-D-19-00211R2 |
| **Full Title:** | Multifaceted Hi-C benchmarking: what makes a difference in chromosome-scale genome scaffolding? |
| **Article Type:** | Research |
| **Funding Information:** | RIKEN — Dr. Ichiro Hiratani, Dr. Shigehiro Kuraku<br>Ministry of Education, Culture, Sports, Science and Technology (18H05530) — Dr. Ichiro Hiratani |
| **Abstract:** | Background: Hi-C is derived from chromosome conformation capture (3C) and targets chromatin contacts on a genomic scale. This method has also been used frequently in scaffolding nucleotide sequences obtained by de novo genome sequencing and assembly, in which the number of resultant sequences rarely converges to the chromosome number. Despite its prevalent use, the sample preparation methods for Hi-C have not been intensively discussed, especially from the standpoint of genome scaffolding.<br>Results: To gain insight into the best practice of Hi-C scaffolding, we performed a multifaceted methodological comparison using vertebrate samples and optimized various factors during sample preparation, sequencing, and computation. As a result, we identified several key factors that helped improve Hi-C scaffolding, including the choice and preparation of tissues, library preparation conditions, the choice of restriction enzyme(s), and the choice of scaffolding program and its usage.<br>Conclusions: This study provides the first comparison of multiple sample preparation kits/protocols and computational programs for Hi-C scaffolding by an academic third party. We introduce a customized protocol designated 'inexpensive and controllable Hi-C protocol', which incorporates the optimal conditions identified in this study, and demonstrated this technique on chromosome-scale genome sequences of the Chinese softshell turtle Pelodiscus sinensis. |
| **Corresponding Author:** | Shigehiro Kuraku<br><br>JAPAN |
| **Corresponding Author Secondary Information:** | |
| **Corresponding Author's Institution:** | |
| **Corresponding Author's Secondary Institution:** | |
| **First Author:** | Mitsutaka Kadota |
| **First Author Secondary Information:** | |
| **Order of Authors:** | Mitsutaka Kadota<br>Osamu Nishimura<br>Hisashi Miura<br>Kaori Tanaka<br>Ichiro Hiratani<br>Shigehiro Kuraku |
| **Order of Authors Secondary Information:** | |
| **Response to Reviewers:** | We have uploaded a letter containing our response to the editor's and reviewer's |

Powered by Editorial Manager® and ProduXion Manager® from Aries Systems Corporation

| | |
|---|---|
| | request and comments. |
| **Additional Information:** | |
| **Question** | **Response** |
| Are you submitting this manuscript to a special series or article collection? | No |
| **Experimental design and statistics**<br><br>Full details of the experimental design and statistical methods used should be given in the Methods section, as detailed in our Minimum Standards Reporting Checklist. Information essential to interpreting the data presented should be made available in the figure legends.<br><br>Have you included all the information requested in your manuscript? | Yes |
| **Resources**<br><br>A description of all resources used, including antibodies, cell lines, animals and software tools, with enough information to allow them to be uniquely identified, should be included in the Methods section. Authors are strongly encouraged to cite Research Resource Identifiers (RRIDs) for antibodies, model organisms and tools, where possible.<br><br>Have you included the information requested as detailed in our Minimum Standards Reporting Checklist? | Yes |
| **Availability of data and materials**<br><br>All datasets and code on which the conclusions of the paper rely must be either included in your submission or deposited in publicly available repositories (where available and ethically appropriate), referencing such data using a unique identifier in the references and in the "Availability of Data and Materials" section of your manuscript. | Yes |

| | |
|---|---|
| Have you have met the above requirement as detailed in our Minimum Standards Reporting Checklist? | |

Manuscript
Click here to view linked References
Click here to access/download;Manuscript;chcked3_MS-HiC_benchmarking-from authors 2.docx

# Multifaceted Hi-C benchmarking: what makes a difference in chromosome-scale genome scaffolding?

Mitsutaka Kadota[1*], Osamu Nishimura[1*], Hisashi Miura[2], Kaori Tanaka[1,3], Ichiro Hiratani[2], and Shigehiro Kuraku[1]

[1] Laboratory for Phyloinformatics, RIKEN Center for Biosystems Dynamics Research (BDR), Kobe, 650-0047, Japan, [2] Laboratory for Developmental Epigenetics, RIKEN BDR, Kobe, 650-0047, Japan, [3] Present address: Division of Transcriptomics, Medical Institute of Bioregulation, Kyushu University, Fukuoka, 812-0054, Japan

*These authors contributed equally to this study.

Correspondence address. Shigehiro Kuraku, Laboratory for Phyloinformatics, RIKEN BDR, Japan. Tel: +81 78 306 3048; Fax: +81 78 306 3048; E-mail: shigehiro.kuraku@riken.jp

ORCIDs:

Mitsutaka Kadota, 0000-0002-1674-6697;

Osamu Nishimura, 0000-0003-1969-2580;

Hisashi Miura, 0000-0003-1270-776X;

Ichiro Hiratani, 0000-0003-3710-3540;

Shigehiro Kuraku, 0000-0003-1464-8388

**Abstract**

**Background:** Hi-C is derived from chromosome conformation capture (3C) and targets chromatin contacts on a genomic scale. This method has also been used frequently in scaffolding nucleotide sequences obtained by *de novo* genome sequencing and assembly, in which the number of resultant sequences rarely converges to the chromosome number. Despite its prevalent use, the sample preparation methods for Hi-C have not been intensively discussed, especially from the standpoint of genome scaffolding.

**Results:** To gain insight into the best practice of Hi-C scaffolding, we performed a multifaceted methodological comparison using vertebrate samples and optimized various factors during sample preparation, sequencing, and computation. As a result, we identified several key factors that helped improve Hi-C scaffolding, including the choice and preparation of tissues, library preparation conditions, the choice of restriction enzyme(s), and the choice of scaffolding program and its usage.

**Conclusions:** This study provides the first comparison of multiple sample preparation kits/protocols and computational programs for Hi-C scaffolding by an academic third party. We introduce a customized protocol designated 'inexpensive and controllable Hi-C (iconHi-C) protocol', which incorporates the optimal conditions identified in this study, and demonstrated this technique on chromosome-scale genome sequences of the Chinese softshell turtle *Pelodiscus sinensis*.

**Keywords:** Hi-C, genome scaffolding, chromosomes, proximity-guided assembly, softshell turtle

## Background

Chromatin, a complex of nucleic acids (DNA and RNA) and proteins, exhibits a complex three-dimensional organization in the nucleus, which enables the intricate regulation of the expression of genome information via spatio-temporal control (reviewed in [1]). To characterize chromatin conformation on a genomic scale, the Hi-C method was introduced as a derivative of chromosome conformation capture (3C) (Fig. 1A; [2]). This method detects chromatin contacts on a genomic scale via the digestion of cross-linked DNA molecules with restriction enzymes, followed by proximity ligation of the digested DNA molecules. Massively parallel sequencing of the library containing ligated DNA molecules enables the comprehensive quantification of contacts both within and between chromosomes, which is presented in a heatmap that is conventionally called the 'contact map' [3].

Analyses of chromatin conformation using Hi-C have revealed more frequent contacts between more closely linked genomic regions, which has recently prompted the use of this method in scaffolding *de novo* genome sequences [4-6]. In *de novo* genome sequencing, the number of assembled sequences is usually far larger than the number of chromosomes in the karyotype of the species of interest, regardless of the sequencing platform chosen [7]. The application of Hi-C scaffolding enabled a remarkable enhancement of sequence continuity to reach a chromosome scale, and the integration of fragmentary sequences into longer sequences, which are similar in number to that of chromosomes in the karyotype.

In early 2018, commercial Hi-C library preparation kits were introduced (Fig. 1B), and *de novo* genome assembly was revolutionized by the release of versatile computational programs for Hi-C scaffolding (Table 1), namely LACHESIS [4], HiRise

[8], SALSA [9, 10], and 3d-dna [11] (reviewed in [12]). These movements assisted the rise of mass sequencing projects targeting a number of species, such as the Earth BioGenome Project (EBP) [13], the Genome 10K (G10K)/Vertebrate Genome Project (VGP) [14], and the DNA Zoo Project [15]. Optimization of Hi-C sample preparation, however, has been limited [16], which leaves room for the improvement of efficiency and the reduction of required sample quantity. Thus, the specific factors that are key for Hi-C scaffolding remain unexplored, mainly because of the costly and resource-demanding nature of this technology.

In addition to performing protocol optimization using human culture cells, we focused on the softshell turtle *Pelodiscus sinensis* (Fig. 2). This species has been adopted as a study system for evolutionary developmental biology (Evo-Devo), including the study of the formation of the dorsal shell (carapace) (reviewed in [17]). Access to genome sequences of optimal quality by relevant research communities is desirable in this field. In Japan, live materials (adults and embryos) of this species are available through local farms mainly between May and August, which implies its high utility for sustainable research. A previous cytogenetic report revealed that the karyotype of this species consists of 33 chromosome pairs including Z and W chromosomes (2n = 66) that show a wide variety of sizes (conventionally categorized as macrochromosomes and microchromosomes) [18]. Despite the moderate global GC-content in its whole genome at around 44%, the intragenomic heterogeneity of GC-content between and within the chromosomes has been suggested [19]. A wealth of cytogenetic efforts on this species led to the accumulation of fluorescence *in situ* hybridization (FISH)-based mapping data for 162 protein-coding genes covering almost

all chromosomes [18-22], which serve as structural landmarks for validating genome assembly sequences.

A draft sequence assembly of the softshell turtle genome was built using short reads and was released in 2013 [23]. This sequence assembly achieved the N50 scaffold length of >3.3 Mb but remains fragmented into approximately 20,000 sequences (see Supplementary Table S1). The longest sequence in this assembly is only slightly larger than 16 Mb, which is much shorter than the largest chromosome size estimated from the karyotype report [18]. The total size of the assembly is approximately 2.2 Gb, which is a moderate size for a vertebrate species. Because of the affordable genome size, sufficiently complex structure, and availability of validation methods, we reasoned that the genome of this species is a suitable target for our methodological comparison, and its improved genome assembly is expected to assist a wide range of genome-based studies of this species.

## Results

### Stepwise QC prior to large-scale sequencing

The assessment of the quality of prepared libraries before engaging in costly sequencing would be ideal. According to the literature [16, 24], we routinely control the quality of Hi-C DNAs and Hi-C libraries by observing DNA size shifts via digestion targeting the restriction sites in properly prepared samples (Fig. 3). More concretely, a successfully ligated Hi-C DNA sample should exhibit a slight increase in the length of its restricted DNA fragments after ligation (QC1), which serves as an indicator of qualified samples (e.g., Sample 1 in Fig. 3B). In contrast, an unsuccessfully prepared Hi-C DNA does not

exhibit this length recovery (e.g., Sample 2 in Fig. 3B). In a subsequent step, DNA molecules in a successfully prepared HindIII-digested Hi-C library should contain the NheI restriction site at a high probability. Thus, the length distribution observed after NheI digestion of the prepared library serves as an indicator of qualified or disqualified products (QC2; Fig. 3C). This series of QCs is incorporated into our protocol by default (Supplementary Protocol S1) and can also be performed in combination with sample preparation using commercial kits if it employs a single restriction enzyme.

Some of the libraries prepared by us passed the QC steps performed before sequencing but yielded an unfavourably large proportion of invalid read pairs. To identify such libraries, we routinely performed small-scale sequencing for quick and inexpensive QC (designated ‘QC3’) using the HiC-Pro program [25] (see Fig. 4 for the read pair categories assigned by HiC-Pro). Our test using variable input data sizes (500 K to 200 M read pairs) resulted in highly similar breakdowns into different categories of read pair properties (Supplementary Table S2) and guaranteed QC3 with an extremely small data size of 1 M or fewer reads. These post-sequencing QC steps, which do not incur a large cost, are expected to help avoid the large-scale sequencing of unsuccessful libraries that have somehow passed through the QC1 and QC2 steps. Importantly, libraries that have passed QC3 can be further sequenced with greater depth, as necessary.

**Optimization of sample preparation conditions**

We identified overt differences between the sample preparation protocols of published studies and those of commercial kits, especially regarding the duration of fixation and enzymatic reaction as well as the library preparation method used. (Fig. 1B). Therefore,

we first sought to optimize the conditions of several of these steps using human culture cells.

To evaluate the effect of the degree of cell fixation, we prepared Hi-C libraries from GM12878 cells fixed for 10 and 30 minutes. Our comparison did not detect any marked differences in the quality of the Hi-C DNA (QC1; Fig. 5A) and Hi-C library (QC2; Fig. 5B). However, libraries that were prepared with a longer fixation time exhibited a larger proportion of dangling end read pairs and religation read pairs, as well as a smaller proportion of valid interaction reads (Fig. 5C). The increase in the duration of cell fixation also reduced the proportion of long-range (>1 Mb) interactions among the overall captured interactions (Fig. 5D).

The reduced preparation time of commercial Hi-C kits (up to two days according to their advertisement) is attributable mainly to shortened restriction and ligation times (Fig. 1B). To monitor the effect of shortening these enzymatic reactions, we first analysed the progression of restriction and ligation in a time-course experiment using GM12878 cells. We observed the persistent progression of restriction up to 16 hours and of ligation up to 6 hours (Fig. 6). To scrutinize further the possible adverse effects of the prolonged reaction, Hi-C libraries of GM12878 cells were prepared with variable durations of restriction digestion (1 hour and 16 hours) and ligation (15 minutes, 1 hour, and 6 hours). We found that the proportions of dangling end and religation read pairs were reduced in cases with an extended duration of restriction digestion  (Supplementary Table S4). The yield of the library, which can be estimated from the number of PCR cycles, increased with the extended duration of ligation without any effect on the proportion of valid interaction read pairs (Supplementary Table S4). The proportion of valid interaction read pairs containing the proper DpnII

junction sequence ‘GATCGATC’ also remained unchanged, suggesting that the prolonged reaction times did not induce any adverse effects, such as star activity of the restriction enzyme.

**Multifaceted comparison using softshell turtle samples**

Based on the detailed optimization of the sample preparation conditions described above, we built an original protocol, designated the ‘iconHi-C protocol’, that included a 10 minute-long cell fixation, 16 hour-long restriction, 6 hour-long ligation, and successive QC steps (Methods; also see Supplementary Protocol S1; Fig. 1B).

We performed Hi-C sample preparation and scaffolding using tissues from a female Chinese softshell turtle which has both Z and W chromosomes [18]. We prepared Hi-C libraries using various tissues (liver or blood cells), restriction enzymes (HindIII or DpnII), and protocols (our iconHi-C protocol, the Arima kit in conjunction with the KAPA Hyper Prep Kit, or the Phase kit), as outlined in Fig. 7A (see Supplementary Table S5; Supplementary Fig. S1). As in some of the existing protocols (e.g. [26]), we performed T4 DNA polymerase treatment in our iconHi-C protocol (Library a–d), expecting reduced proportions of ‘dangling end’ read pairs that contain no ligated junction, and thus do not contribute to Hi-C scaffolding. We also incorporated this T4 DNA polymerase treatment into the workflow of the Arima kit (Library e vs. Library f without this additional treatment). Furthermore, we tested a lesser degree of PCR amplification (11 cycles) together with the use of the Phase kit which recommends as many as 15 cycles by default (Library h vs. Library g; Fig. 7A).

All samples prepared using the iconHi-C protocol passed both controls, QC1 and QC2 (Fig. 7B). The prepared Hi-C libraries were sequenced to obtain one million

127 nt-long read pairs and were subjected to QC3 using the HiC-Pro program (Fig. 8). As a result of this QC3, the largest proportion of 'valid interaction' pairs was observed for Arima libraries (Library e and f). Regarding the iconHi-C libraries (Library a–d), fewer 'unmapped' and 'religation' pairs were detected for the DpnII libraries compared with HindIII libraries. It should be noted that the QC3 of the softshell turtle libraries generally produced lower proportions of the 'valid interaction' category and larger proportions of 'unmapped pairs' and 'pairs with singleton' than with the human libraries. This cross-species difference may be attributable to the use of incomplete genome sequences as a reference for Hi-C read mapping (Supplementary Table S1). This invokes a caution when comparing QC results across species.

**Scaffolding using variable input and computational conditions**

In this study, only well-maintained open-source programs, i.e., 3d-dna and SALSA2, were used in conjunction with variable combinations of input libraries, input read amounts, input sequence cut-off lengths, and number of iterative misjoin correction rounds (Fig. 9A). As a result of scaffolding, we observed a wide spectrum of basic metrics, including the N50 scaffold length (0.6–303 Mb), the largest scaffold length (8.7–703 Mb), and the number of chromosome-sized (>10 Mb) sequences (0–65) (Fig. 9; Supplementary Table S6).

First, using the default parameters, 3d-dna consistently produced more continuous assemblies than did SALSA2 (see Assembly 1 vs. 5, 3 vs. 6, 9 vs. 10, and 11 vs. 12 in Fig. 9). Second, the increase in the number of iterative corrections ('-r' option of 3d-dna) resulted in relatively large N50 lengths, but with more missing orthologues (see Assembly 3 and 13–14). Third, a smaller input sequence cut-off length ('-i' option

of 3d-dna) resulted in a smaller number of scaffolds but again, with more missing orthologues (see Assembly 3 and 15–17). Fourth, the use of the liver libraries consistently resulted in a higher continuity than the use of the blood cell libraries (see Assembly 1 vs. 2 and 3 vs. 4 in Fig. 9).

Assembly 8, which resulted from input Hi-C reads derived from both liver and blood, exhibited an outstandingly large N50 scaffold length (303 Mb) but a larger number of undetected reference orthologues (141 orthologues) than most of the other assemblies. The largest scaffold (scaffold 5) in this assembly is approximately 703 Mb long, causing a large N50 length, and accounts for approximately one-third of the whole genome in length, as a result of possible chimeric assembly that bridged 14 putative chromosomes (see Supplementary Fig. S4).

The choice of restriction enzymes has not been discussed in depth in the context of genome scaffolding. Here, we prepared Hi-C libraries separately with HindIII and DpnII. We did not mix multiple enzymes in the same reaction (other than using the Arima kit which originally employs two enzymes); rather, we performed a single scaffolding run with both HindIII-based and DpnII-based reads (see Assembly 7 in Fig. 9). As expected, our comparison of multiple metrics yielded a more successful result with DpnII than with HindIII (see Assembly 1 vs. 3 as well as 2 vs. 4; Fig. 9). However, the mixed input of HindIII-based and DpnII-based reads did not necessarily yield a better scaffolding result (see Assembly 3 vs. 7).

To gain additional insight regarding the evaluation of the scaffolding results, we assessed the contact maps constructed upon the Hi-C scaffolds (Supplementary Fig. S5). The comparison of Assembly 3, 9 and 11, which represent the three different preparation methods, revealed anomalous patterns, particularly for Assembly 11, with

intensive contact signals separated from the diagonal line that indicate the presence of errors in the scaffolds [15]. We also performed genome-wide alignments between the Hi-C scaffolds obtained. The comparison of Assembly 3, 9, and 11 revealed a high similarity between Assembly 3 and 9, while Assembly 11 exhibited a significantly larger number of inconsistencies against either of the other two assemblies (Supplementary Fig. S6). These observations are consistent with the evaluation based on sequence length and gene space completeness, which alone does not, however, provide a reliable metric for the assessment of the quality of scaffolding.

**Validation of scaffolding results using transcriptome and FISH data**

In addition to the above-mentioned evaluation of the scaffolding results, we assessed the sequence continuity using independently obtained data. First, we mapped assembled transcript sequences onto our Hi-C scaffold sequences (see Methods). This did not show any substantial differences between the assemblies (Supplementary Table S7), probably because the sequence continuity after Hi-C scaffolding exceeded that of RNA-seq library inserts, even when the length of intervening introns in the genome was considered. The present analysis with RNA-seq data did not provide an effective source of continuity validation.

Second, we referred to the fluorescence *in situ* hybridization (FISH) mapping data of 162 protein-coding genes from published cytogenetic studies [18-22], which allowed us to check the locations of those genes with our resultant Hi-C assemblies. In this analysis, we evaluated Assembly 3, 7, and 9 (see Fig. 9A) that showed better scaffolding results in terms of sequence length distribution and gene space completeness (Fig. 9D). As a result, we confirmed the positioning of almost all genes and their

continuity over the centromeres, which encompassed not only large but also small chromosomes (conventionally called ‘macrochromosomes’ and ‘microchromosomes’; Fig. 10). Two genes that were not confirmed by Assembly 7 (*UCHL1* and *COX15*; Fig. 10) were found in separate scaffold sequences that were shorter than 1 Mb, which indicates insufficient scaffolding. Conversely, the gene array including *RBM5*, *TKT*, *WNT7A*, and *WNT5A*, previously shown by FISH, was consistently unconfirmed by all three assemblies (Fig. 10), which did not provide any clues for among-assembly evaluation or perhaps indicates an erroneous interpretation of FISH data in a previous study.

## Discussion

### Starting material: not genomic DNA extraction but *in situ* cell fixation

In genome sequencing, best practices for high molecular weight DNA extraction have often been discussed (e.g. [27]). This factor is fundamental to building longer contigs, regardless of the use of short-read or long-read sequencing platforms. Moreover, the proximity ligation method using Chicago libraries provided by Dovetail Genomics which is based on *in vitro* chromatin reconstruction [8], uses genomic DNA as starting material. In contrast, proximity-guided assembly enabled by Hi-C employs cellular nuclei with preserved chromatin conformation, which brings a new technical challenge regarding appropriate sampling and sample preservation in genomics.

In the preparation of the starting material, it is important to optimize the degree of cell fixation depending on sample choice, to obtain an optimal result in Hi-C

scaffolding (Fig. 5). Another practical indication of tissue choice was obtained by examining Assembly 8 (Fig. 9A). This assembly was produced by 3d-dna scaffolding using both liver and blood libraries (Library b and d), which led to an unacceptable result possibly caused by over-assembly (Fig. 9B–D; also see Results). It is likely that increased cellular heterogeneity, which possibly introduces excessive conflicting chromatin contacts, did not allow the scaffolding program to group and order the input genome sequences properly. In brief, we recommend the use of samples with modest cell-type heterogeneity that are amenable to thorough fixation.

**Considerations regarding sample preparation**

In this study, we did not test all commercial Hi-C kits available in the market. This was partly because the Dovetail Hi-C kit specifies the non-open source program HiRise as the only supported downstream computation solution and does not allow a direct comparison with other kits, namely those from Phase Genomics and Arima Genomics.

According to our calculations, the preparation of a Hi-C library using the iconHi-C protocol would be at least three times cheaper than the use of a commercial kit. Practically, the cost difference would be even larger, either when the purchased kit is not fully consumed or when the post-sequencing computation steps cannot be undertaken in-house, which implies additional outsourcing costs.

The genomic regions that are targeted by Hi-C are determined by the choice of restriction enzymes. Theoretically, 4-base cutters (e.g. DpnII), which potentially have more frequent restriction sites on the genome, are expected to provide a higher resolution than 6-base cutters (e.g., HindIII) [16]. Obviously, the use of restriction enzymes that were not employed in this study might be promising in the adaptation of

the protocol to organisms with variable GC-content or methylation profiles. However, this might not be so straightforward when considering the interspecies variation in GC-content and the intra-genomic heterogeneity. The use of multiple enzymes in a single reaction is a promising approach; however, from a computational viewpoint, not all scaffolding programs are compatible with multiple enzymes (see Table 1 for a comparison of the specification of scaffolding programs). Another technical downside of this approach is the incompatibility of DNA ends restricted by multiple enzymes, with restriction-based QCs, such as the QC2 step of our iconHi-C protocol (Fig. 3). Therefore, in this study, DpnII and HindIII were used separately in the iconHi-C protocol, which resulted in a higher scaffolding performance with the DpnII library (Figs. 8 and 9), as expected. In addition, we input the separately prepared DpnII and HindIII libraries together in scaffolding (Assembly 7), but this approach did not lead to higher scaffolding performance (Figs. 9B–D and 10). The Arima kit employs two different enzymes that can produce a much greater number of restriction site combinations, because one of these two enzymes recognizes the nucleotide stretch 'GANTC'. The increase of restriction site combinations might have possibly contributed to the larger proportion of valid interaction pairs (Fig. 8). Scaffolding with the libraries prepared using this kit resulted in one of the most acceptable assemblies (Assembly 9). However, this result did not explicitly exceed the performance of scaffolding with the iconHi-C libraries, including the one that used a single enzyme (DpnII; Library d).

Overamplification by PCR is a concern regarding the use of commercial kits (with the exception of the Arima kit used with the Arima-QC2) because their manuals specify the use of a certain number of PCR cycles *a priori* (15 cycles for the Phase kit

and 11 cycles for the Dovetail Hi-C kit) (Supplementary Table S8). In our iconHi-C protocol, an optimal number of PCR cycles is estimated by means of a preliminary real-time PCR using a small aliquot (Step 11.25 to 11.29 in Supplementary Protocol S1), as done traditionally for other library types (e.g., [28]). This procedure allowed us to reduce the number of PCR cycles, down to as few as five cycles (Supplementary Table S5). The Dovetail Hi-C kit recommends the use of larger amounts of kit components than that specified for a single sample, depending on the genome size, as well as the degree of genomic heterozygosity and repetitiveness, of the species of interest. In contrast, with our iconHi-C protocol, we always prepared a single library, regardless of those species-specific factors, which seemed to suffice in all the cases tested.

Commercial Hi-C kits, which usually advertise easiness and quickness of use, have largely shortened the protocol down to two days, compared with the published non-commercial protocols (e.g., [16, 26]). Such time-saving protocols are achieved mainly by shortening the duration of restriction enzyme digestion and ligation (Fig. 1B). Our assessment, however, revealed unsaturated reaction within the shortened time frames employed in the commercial kits (Fig. 6), which was accompanied by an unfavorable composition of read pairs (Supplementary Table S4). Our attempt to insert a step of T4 DNA polymerase treatment in the sample preparation of the Arima kit protocol resulted in reduced ‘dangling end’ reads (Library e vs. f in Fig. 8). Regarding the Phase kit, transposase-based library preparation contributes largely to its shortened protocol, but this does not allow flexible control of library insert lengths. Recent protocols (versions 1.5 and 2.0) of the Phase kit instruct users to employ a largely reduced DNA amount in the tagmentation reaction, which should mitigate the difficulty in controlling insert length but require excessive PCR amplification. The Arima and

Phase kits assume that the quality control of Hi-C DNA is based on the yield, and not the size, of DNA (see Fig. 1B). Nevertheless, quality control based on DNA size (equivalent to QC1 in iconHi-C) is feasible by taking aliquots at each step of sample preparation. In particular, if preparing a small number of samples for Hi-C, as practised typically for genome scaffolding, one should opt to consider these points, even when using commercial kits, to improve the quality of the prepared libraries and scaffolding products.

**Considerations regarding sequencing**

The quantity of Hi-C read pairs to be input for scaffolding is critical because it accounts for the majority of the cost of Hi-C scaffolding. Our protocol introduces a thorough safety system to prevent sequencing unsuccessful libraries, first by performing pre-sequencing QCs for size shift analyses (Fig. 3) and second via small-scale (down to 500 K read pairs) sequencing (see Results; also see Supplementary Tables S2 and S9).

Our comparison showed a dramatic decrease in assembly quality in cases in which <100 M read pairs were used (see the comparison of Assembly 18–22 described above; Fig. 9; also see [29]). Nevertheless, we obtained optimal results with a smaller number of reads (ca. 160 M per 2.2 Gb of genome) than that recommended by the manufacturers of commercial kits (e.g., 100 M per 1 Gb of genome for the Dovetail Hi-C kit and 200 M per Gb of genome for the Arima kit). As generally and repeatedly discussed [29], the proportion of informative reads and their diversity, rather than just the overall number of obtained reads, is critical.

In terms of read length, we did not perform any comparisons in this study. Longer reads may enhance the fidelity of the characterization of the read pair properties

and allow precise QC. Nevertheless, the existing Illumina sequencing platform has enabled the less expensive acquisition of 150 nt-long paired-end reads, which did not prompt us to vary the read length.

**Considerations regarding computation**

In this study, 3d-dna produced a more reliable scaffolding output than did SALSA2, whether sample preparation employed a single or multiple enzyme(s) (Fig. 9B–D). On the other hand, 3d-dna required a greater amount of time for the completion of scaffolding than did SALSA2. Apart from the choice of program, several points should be considered if successful scaffolding for a smaller investment is to be achieved. In general, Hi-C scaffolding results should not be taken for granted, and it is necessary to improve them by referring to contact maps using an interactive tool, such as Juicebox [15]. In this study, however, we compared raw scaffolding output to evaluate sample preparation and reproducible computational steps.

We used various parameters of the scaffolding programs (Fig. 9A). First, the Hi-C scaffolding programs that are available currently have different default length cut-off values for input sequences (e.g., 15000 bp for the '-i' parameter in 3d-dna and 1000 bp for the '-c' parameter in SALSA2). Only sequences that are longer than the cut-off length value contribute to sequence scaffolding towards chromosome sizes, while sequences shorter than the cut-off length are implicitly excluded from the scaffolding process and remain unchanged. Typically, when using the Illumina sequencing platform, genomic regions with unusually high frequencies of repetitive elements and GC-content are not assembled into sequences with a sufficient length (see [30]). Such genomic regions tend to be excluded from chromosome-scale Hi-C scaffolds because

their length is smaller than the threshold. Alternatively, these regions may be excluded because few Hi-C read pairs are mapped to them, even if they exceed the cut-off length. The deliberate setting of a cut-off length is recommended if particular sequences with relatively small lengths are the target of scaffolding. It should be noted that lowering the length threshold can result in frequent misjoins in the scaffolding output (Fig. 9B–D) or in overly long computational times. Regarding the number of iterative misjoin correction rounds (the '-r' parameter in 3d-dna and 'i' parameter in SALSA2), our attempts of using increased values did not necessarily yield favourable results (Fig. 9B–D). This did not provide a consistent optimal range of values but rather suggests the importance of performing multiple scaffolding runs with varying parameters.

**Considerations regarding the assessment of chromosome-scale genome sequences**

Our assessment using cytogenetic data confirmed the continuity of gene linkage over the obtained chromosome-scale sequences (Fig. 10). This validation was required by the almost saturated scores of typical gene space completeness assessment tools such as BUSCO (Supplementary Table S6) and by transcript contig mapping (Supplementary Table S7), neither of which provided an effective metric for evaluation.

For further evaluation of our scaffolding results, we referred to the sequence length distributions of the genome assemblies of other turtle species that are regarded as being chromosome-scale data. This analysis yielded values of the basic metrics that were comparable to those of our Hi-C scaffolds of the softshell turtle, i.e. an N50 length of 127.5 Mb and a maximum sequence length of 344.5 Mb for the genome assembly of the green sea turtle (*Chelonia mydas*) released by the DNA Zoo Project [15] and an N50 length of 131.6 Mb and a maximum length of 370.3 Mb for the genome assembly of the

Goode's thornscrub tortoise (*Gopherus evgoodei*) released by the Vertebrate Genome Project (VGP) [14]. Scaffolding results should be evaluated by referring to the estimated N50 length and the maximum length based on the actual value and to the length distribution of chromosomes in the intrinsic karyotype of the species in question, or of its close relative. Turtles tend to have an N50 length of approximately 130 Mb and a maximum length of 350 Mb, while many teleost fish genomes exhibit an N50 length as low as 20–30 Mb and a maximum length of <100 Mb [31]. If these values are excessive, the scaffolded sequences harbour overassembly, which erroneously boosts length-based metrics. Thus, higher values, which are conventionally regarded as signs of successful sequence assembly, do not necessarily indicate higher precision.

The total length of assembly sequences is expected to increase after Hi-C scaffolding, because scaffolding programs simply insert a stretch of the unassigned base 'N' with a uniform length between input sequences in most cases (500 bp as a default in both 3d-dna and SALSA2). However, this has a minor impact on the total length of assembled sequences.

## Conclusions

In this study, we introduced the iconHi-C protocol which implements successive QC steps. We also assessed potential key factors for improving Hi-C scaffolding. Overall, our study showed that small variations in sample preparation or computation for scaffolding can have a large impact on scaffolding output, and that any scaffolding output should ideally be validated using independent information, such as cytogenetic data, long reads, or genetic linkage maps. The present study aimed to evaluate the output of reproducible computational steps, which in practice should be followed by the

modification of the raw scaffolding output by referring to independent information or by analysing chromatin contact maps. The study employed limited combinations of species, sample prep methods, scaffolding programs, and its parameters, and we will continue to test different conditions for kits/programs that did not necessarily perform well here using our specific materials.

## Methods

### Initial genome assembly sequences

The softshell turtle (*Pelodiscus sinensis*) assembly published previously [23] was downloaded from NCBI GenBank (GCA_000230535.1), whose gene space completeness and length statistics were assessed by gVolante [32] (see Supplementary Table S1 for the assessment results). Although it could be suggested to remove haplotigs before Hi-C scaffolding [33], we omitted this step because of the low frequency of the reference orthologues with multiple copies (0.72%; Supplementary Table S1), indicating a minimal degree of haplotig contamination.

### Animals and cells

We sampled tissues (liver and blood cells) from a female purchased from a local farmer in Japan, because the previous whole genome sequencing used the whole blood of a female [23]. All experiments were conducted in accordance with the Guideline of the Institutional Animal Care and Use Committee of RIKEN Kobe Branch (Approval ID: A2017-12).

The human lymphoblastoid cell line GM12878 (Coriell Cat# GM12878, RRID:CVCL_7526) was purchased from the Coriell Cell Repositories and cultured in

RPMI-1640 medium (Thermo Fisher Scientific) supplemented with 15% FBS, 2 mM L-glutamine, and a 1× antibiotic-antimycotic solution (Thermo Fisher Scientific), at 37 °C, 5% $CO_2$, as described previously [34].

**Hi-C sample preparation using the original protocol**

We have made modifications to the protocols that are available in the literature [3, 26, 35] (Fig. 1B). The full version of our 'inexpensive and controllable Hi-C (iconHi-C)' protocol is described in Supplementary Protocol S1 and available at Protocols.io [36].

**Hi-C sample preparation using commercial kits**

The Proximo Hi-C kit (Phase Genomics) which employs the restriction enzyme Sau3A1 and transposase-based library preparation [37] (Fig. 1B) was used to prepare a library from 50 mg of the softshell turtle liver according to the official ver. 1.0 animal protocol provided by the manufacturer (Library g in Fig. 7A) and a library from 10 mg of the liver that was amplified with a reduced number of PCR cycles based on a preliminary real-time qPCR using an aliquot (Library h; see [28] for the details of the pre-determination of the optimal number of PCR cycles). The Arima-HiC kit (Arima Genomics), which employs a restriction enzyme cocktail (Fig. 1B), was used in conjunction with the KAPA Hyper Prep Kit (KAPA Biosystems), protocol ver. A160108 v00, to prepare a library using the softshell turtle liver, according to its official animal vertebrate tissue protocol (ver. A160107 v00) (Library f) and a library with an additional step of T4 DNA polymerase treatment for reducing 'dangling end' reads (Library e). This additional treatment is detailed in Step 8.2 (for DpnII-digested samples) of Supplementary Protocol S1.

## DNA sequencing

Small-scale sequencing for library QC (QC3) was performed in-house to obtain 127 nt-long paired-end reads on an Illumina HiSeq 1500 in the Rapid Run Mode. For evaluating the effects of variable duration of the restriction digestion and ligation reactions, sequencing was performed on an Illumina MiSeq using the MiSeq Reagent Kit v3 to obtain 300 nt-long paired-end reads. Large-scale sequencing for Hi-C scaffolding was performed to obtain 151 nt-long paired-end reads on an Illumina HiSeq X. The obtained reads underwent quality control using FastQC ver. 0.11.5 (FastQC, RRID:SCR_014583; https://www.bioinformatics.babraham.ac.uk/projects/fastqc/), and low-quality regions and adapter sequences in the reads were removed using Trim Galore ver. 0.4.5 (TrimGalore, RRID:SCR_ 011847; https://www.bioinformatics.babraham.ac.uk/projects/trim_galore/) with the parameters '-e 0.1 -q 30'.

## Post-sequencing quality control (QC3) of Hi-C libraries

For post-sequencing library QC, one million trimmed read pairs for each Hi-C library were sampled using the 'subseq' function of the program seqtk ver. 1.2-r94 (https://github.com/lh3/seqtk). The resultant sets of read pairs were processed using HiC-Pro ver. 2.11.1 [25] with bowtie2 ver. 2.3.4.1 [38] to evaluate the insert structure and mapping status onto the softshell turtle genome assembly PelSin_1.0 (GCF_000230535.1) or the human genome assembly hg19. This resulted in categorization as valid interaction pairs and invalid pairs, with the latter being divided further into 'dangling end', 'religation', 'self circle', and 'single-end' pairs (Fig. 4). To

process the read pairs derived from the libraries prepared using either HindIII or DpnII (Sau3AI) with the iconHi-C protocol (Library a–d) and the Phase kit (Library g and h), the restriction fragment file required by HiC-Pro was prepared according to the script 'digest_genome.py' of HiC-Pro. To process the reads derived from the Arima kit (Library e and f), all restriction sites ('GATC' and 'GANTC') were inserted into the script. In addition, the nucleotide sequences of all possible ligated sites generated by restriction enzymes were included in a configuration file of HiC-Pro. The details of this procedure and the sample code used are included in Supplementary Protocol S2.

**Computation for Hi-C scaffolding**

To control our comparison with intended input data sizes, a certain number of trimmed read pairs were sampled for each library with seqtk, as described above. Scaffolding was processed with the following methods employing two program pipelines, 3d-dna and SALSA2.

Scaffolding via 3d-dna was performed using Hi-C read mapping onto the genome with Juicer ver. 20180805 (Juicer, RRID:SCR_017226) [39] using the default parameters with BWA ver.0.7.17-r1188 (BWA, RRID:SCR_010910) [40]. The restriction fragment file required by Juicer was prepared by the script 'generate_site_positions.py' script of Juicer. By converting the restriction fragment file of HiC-Pro to the Juicer format, an original script that was compatible with multiple restriction enzymes was prepared (Supplementary Protocol S2). Scaffolding via 3d-dna ver. 20180929 was performed using variable parameters (see Fig. 9A).

Scaffolding via SALSA2 using Hi-C reads was preceded by Hi-C read pair processing with the Arima mapping pipeline ver. 20181207 [41] together with BWA,

SAMtools ver. 1.8-21-gf6f50ac (SAMTOOLS, RRID:SCR_002105) [42], and Picard ver. 2.18.12 (Picard, RRID:SCR_006525) [43]. The mapping result in the binary alignment map (bam) format was converted into a BED file by bamToBed of Bedtools ver. 2.26.0 (BEDTools, RRID:SCR_006646) [44], the output of which was used as the input of scaffolding using SALSA2 ver. 20181212 with the default parameters.

**Completeness assessment of Hi-C scaffolds**

gVolante ver. 1.2.1 [32] was used to perform an assessment of the sequence length distribution and gene space completeness based on the coverage of one-to-one reference orthologues with BUSCO v2/v3 employing the one-to-one orthologue set ‘Tetrapoda’ supplied with BUSCO (BUSCO, RRID:SCR_015008) [45]. No cut-off length was used in this assessment.

**Continuity assessment using RNA-seq read mapping**

Paired-end reads obtained by RNA-seq of softshell turtle embryos at multiple stages were downloaded from NCBI SRA (DRX001576) and were assembled using Trinity ver. 2.7.0 (Trinity, RRID:SCR_013048) [46] with default parameters. The assembled transcript sequences were mapped to the Hi-C scaffold sequences with pblat [47], and the output was assessed with isoblat ver. 0.31 [48].

**Comparison with chromosome FISH results**

Cytogenetic validation of Hi-C scaffolding results was performed by comparing the gene locations on the scaffold sequences with those provided by previous chromosome FISH for 162 protein-coding genes [18-22]. The nucleotide exonic sequences for those

162 genes were retrieved from GenBank and aligned with Hi-C scaffold sequences using BLAT ver. 36x2 (BLAT, RRID:SCR_011919) [49], followed by the analysis of their positions and orientation along the Hi-C scaffold sequences.

**Availability of supporting data**

All sequence data generated in this study have been submitted to the DDBJ Sequence Read Archive (DRA) under accession IDs DRA008313 and DRA008947. The datasets supporting the results of this article are available in FigShare [50] and the *GigaScience* GigaDB database [51].

**Additional files**

Supplementary Figure S1. DNA size distribution of the softshell turtle Hi-C libraries.

Supplementary Figure S2. Pre-sequencing quality control of softshell turtle blood Hi-C libraries (Library a and b).

Supplementary Figure S3. Pre-sequencing quality control (QC2) of the Hi-C libraries generated using the Phase kit (Library g and h).

Supplementary Figure S4. Structural analysis of the possibly chimeric scaffold in Assembly 8.

Supplementary Figure S5. Hi-C contact maps for selected softshell turtle Hi-C scaffolds.

Supplementary Figure S6. Pairwise alignment of Hi-C scaffolds.

Supplementary Table S1. Statistics of the Chinese softshell turtle draft genome assembly before Hi-C.

Supplementary Table S2. HiC-Pro results for the human GM12878 HindIII Hi-C library with reduced reads.

Supplementary Table S3. Quality control of the human GM12878 Hi-C libraries.

Supplementary Table S4. Effect of the duration of restriction enzyme digestion and ligation.

Supplementary Table S5. Quality control of Hi-C libraries.

Supplementary Table S6. Scaffolding results with variable input data and computational parameters.

Supplementary Table S7. Mapping results of assembled transcript sequences onto Hi-C scaffolds.

Supplementary Table S8. Effect of variable degrees of PCR amplification.

Supplementary Table S9. HiC-Pro results for the softshell turtle liver libraries (Library d, e, and h) with reduced reads.

Supplementary Protocol S1. iconHi-C protocol.

Supplementary Protocol S2. Computational protocol to support the use of multiple enzymes.

## Abbreviations

3C: chromosome conformation capture; PCR: polymerase chain reaction; FISH, fluorescence *in situ* hybridization; BUSCO, benchmarking universal single-copy orthologs; NCBI, National Center for Biotechnology Information; NGS, next generation sequencing.


## Funding

This work was supported by intramural grants within RIKEN including the All-RIKEN ‘Epigenome Manipulation Project’ to S.K. and I.H. and by a Grant-in-Aid for Scientific Research on Innovative Areas from the Ministry of Education, Culture, Sports, Science, and Technology (MEXT) to I.H. (18H05530).


## Competing interests

The authors declare that they have no competing interests.

## Acknowledgements

The authors acknowledge Naoki Irie, Juan Pascual Anaya and Tatsuya Hirasawa in Laboratory for Evolutionary Morphology, RIKEN BDR for suggestions for sampling, Rawin Poonperm for comments and discussion on the iconHi-C protocol, Olga Dudchenko, Erez Lieberman-Aiden, Arang Rhie, Sergey Koren, and Jay Ghurye for their technical suggestions for sample preparation and computation, Yoshinobu Uno for guidance in the cytogenetic data interpretation, and Anthony Schmitt of Arima Genomics and Stephen Eacker of Phase Genomics for providing information about the Hi-C kits. The authors also thank the other members of the Laboratory for Phyloinformatics and Laboratory for Developmental Epigenetics in RIKEN BDR for technical support and discussion.

## Author contributions

S.K., I.H., H.M., and M.K. conceived the study. M.K. and K.T. performed laboratory works, and O.N. performed bioinformatic analysis. M.K., O.N., and H.M. analyzed the data. S.K., M.K., and O.N. drafted the manuscript. All authors contributed to the finalization of the manuscript.

## References

1. Rowley MJ and Corces VG. Organizational principles of 3D genome architecture. Nat Rev Genet. 2018;19 12:789-800. doi:10.1038/s41576-018-0060-8.
2. Lieberman-Aiden E, van Berkum NL, Williams L, Imakaev M, Ragoczy T, Telling A, et al. Comprehensive mapping of long-range interactions reveals folding

principles of the human genome. Science. 2009;326 5950:289-93. doi:10.1126/science.1181369.

3. Rao SS, Huntley MH, Durand NC, Stamenova EK, Bochkov ID, Robinson JT, et al. A 3D map of the human genome at kilobase resolution reveals principles of chromatin looping. Cell. 2014;159 7:1665-80. doi:10.1016/j.cell.2014.11.021.

4. Burton JN, Adey A, Patwardhan RP, Qiu R, Kitzman JO and Shendure J. Chromosome-scale scaffolding of de novo genome assemblies based on chromatin interactions. Nat Biotechnol. 2013;31 12:1119-25. doi:10.1038/nbt.2727.

5. Marie-Nelly H, Marbouty M, Cournac A, Flot JF, Liti G, Parodi DP, et al. High-quality genome (re)assembly using chromosomal contact data. Nat Commun. 2014;5 1:5695. doi:10.1038/ncomms6695.

6. Kaplan N and Dekker J. High-throughput genome scaffolding from in vivo DNA interaction frequency. Nat Biotechnol. 2013;31 12:1143-7. doi:10.1038/nbt.2768.

7. Sedlazeck FJ, Lee H, Darby CA and Schatz MC. Piercing the dark matter: bioinformatics of long-range sequencing and mapping. Nat Rev Genet. 2018;19 6:329-46. doi:10.1038/s41576-018-0003-4.

8. Putnam NH, O'Connell BL, Stites JC, Rice BJ, Blanchette M, Calef R, et al. Chromosome-scale shotgun assembly using an in vitro method for long-range

linkage. Genome Res. 2016;26 3:342-50. doi:10.1101/gr.193474.115.

9. Ghurye J, Pop M, Koren S, Bickhart D and Chin CS. Scaffolding of long read assemblies using long range contact information. BMC Genomics. 2017;18 1:527. doi:10.1186/s12864-017-3879-z.

10. Ghurye J, Rhie A, Walenz BP, Schmitt A, Selvaraj S, Pop M, et al. Integrating Hi-C links with assembly graphs for chromosome-scale assembly. PLoS Comput Biol. 2019;15 8:e1007273. doi:10.1371/journal.pcbi.1007273.

11. Dudchenko O, Batra SS, Omer AD, Nyquist SK, Hoeger M, Durand NC, et al. De novo assembly of the Aedes aegypti genome using Hi-C yields chromosome-length scaffolds. Science. 2017;356 6333:92-5. doi:10.1126/science.aal3327.

12. Ghurye J and Pop M. Modern technologies and algorithms for scaffolding assembled genomes. PLoS Comput Biol. 2019;15 6:e1006994. doi:10.1371/journal.pcbi.1006994.

13. Lewin HA, Robinson GE, Kress WJ, Baker WJ, Coddington J, Crandall KA, et al. Earth BioGenome Project: Sequencing life for the future of life. Proc Natl Acad Sci USA. 2018;115 17:4325-33. doi:10.1073/pnas.1720115115.

14. Koepfli KP, Paten B, Genome KCoS and O'Brien SJ. The Genome 10K Project: a way forward. Annu Rev Anim Biosci. 2015;3:57-111. doi:10.1146/annurev-animal-

090414-014900.

15. Dudchenko O, Shamim MS, Batra SS, Durand NC, Musial NT, Mostofa R, et al. The Juicebox Assembly Tools module facilitates de novo assembly of mammalian genomes with chromosome-length scaffolds for under $1000. bioRxiv. 2018:254797. doi:10.1101/254797.

16. Belaghzal H, Dekker J and Gibcus JH. Hi-C 2.0: An optimized Hi-C procedure for high-resolution genome-wide mapping of chromosome conformation. Methods. 2017;123:56-65. doi:10.1016/j.ymeth.2017.04.004.

17. Kuratani S, Kuraku S and Nagashima H. Evolutionary developmental perspective for the origin of turtles: the folding theory for the shell based on the developmental nature of the carapacial ridge. Evol Dev. 2011;13 1:1-14. doi:10.1111/j.1525-142X.2010.00451.x.

18. Matsuda Y, Nishida-Umehara C, Tarui H, Kuroiwa A, Yamada K, Isobe T, et al. Highly conserved linkage homology between birds and turtles: bird and turtle chromosomes are precise counterparts of each other. Chromosome Res. 2005;13 6:601-15. doi:10.1007/s10577-005-0986-5.

19. Kuraku S, Ishijima J, Nishida-Umehara C, Agata K, Kuratani S and Matsuda Y. cDNA-based gene mapping and GC3 profiling in the soft-shelled turtle suggest a

chromosomal size-dependent GC bias shared by sauropsids. Chromosome Res. 2006;14 2:187-202. doi:10.1007/s10577-006-1035-8.

20. Uno Y, Nishida C, Tarui H, Ishishita S, Takagi C, Nishimura O, et al. Inference of the protokaryotypes of amniotes and tetrapods and the evolutionary processes of microchromosomes from comparative gene mapping. PLoS One. 2012;7 12:e53027. doi:10.1371/journal.pone.0053027.

21. Kawai A, Nishida-Umehara C, Ishijima J, Tsuda Y, Ota H and Matsuda Y. Different origins of bird and reptile sex chromosomes inferred from comparative mapping of chicken Z-linked genes. Cytogenet Genome Res. 2007;117 1-4:92-102. doi:10.1159/000103169.

22. Kawagoshi T, Uno Y, Matsubara K, Matsuda Y and Nishida C. The ZW micro-sex chromosomes of the Chinese soft-shelled turtle (Pelodiscus sinensis, Trionychidae, Testudines) have the same origin as chicken chromosome 15. Cytogenet Genome Res. 2009;125 2:125-31. doi:10.1159/000227837.

23. Wang Z, Pascual-Anaya J, Zadissa A, Li W, Niimura Y, Huang Z, et al. The draft genomes of soft-shell turtle and green sea turtle yield insights into the development and evolution of the turtle-specific body plan. Nat Genet. 2013;45 6:701-6. doi:10.1038/ng.2615.

24. Belton JM, McCord RP, Gibcus JH, Naumova N, Zhan Y and Dekker J. Hi-C: a comprehensive technique to capture the conformation of genomes. Methods. 2012;58 3:268-76. doi:10.1016/j.ymeth.2012.05.001.

25. Servant N, Varoquaux N, Lajoie BR, Viara E, Chen CJ, Vert JP, et al. HiC-Pro: an optimized and flexible pipeline for Hi-C data processing. Genome Biol. 2015;16:259. doi:10.1186/s13059-015-0831-x.

26. Sofueva S, Yaffe E, Chan WC, Georgopoulou D, Vietri Rudan M, Mira-Bontenbal H, et al. Cohesin-mediated interactions organize chromosomal domain architecture. Embo j. 2013;32 24:3119-29. doi:10.1038/emboj.2013.237.

27. Mayjonade B, Gouzy J, Donnadieu C, Pouilly N, Marande W, Callot C, et al. Extraction of high-molecular-weight genomic DNA for long-read sequencing of single molecules. Biotechniques. 2016;61 4:203-5. doi:10.2144/000114460.

28. Tanegashima C, Nishimura O, Motone F, Tatsumi K, Kadota M and Kuraku S. Embryonic transcriptome sequencing of the ocellate spot skate Okamejei kenojei. Sci Data. 2018;5:180200. doi:10.1038/sdata.2018.200.

29. DeMaere MZ and Darling AE. bin3C: exploiting Hi-C sequencing data to accurately resolve metagenome-assembled genomes. Genome Biol. 2019;20 1:46. doi:10.1186/s13059-019-1643-1.

30. Botero-Castro F, Figuet E, Tilak MK, Nabholz B and Galtier N. Avian Genomes Revisited: Hidden Genes Uncovered and the Rates versus Traits Paradox in Birds. Mol Biol Evol. 2017;34 12:3123-31. doi:10.1093/molbev/msx236.

31. Hotaling S and Kelley JL. The rising tide of high-quality genomic resources. Mol Ecol Resour. 2019;19 3:567-9. doi:10.1111/1755-0998.12964.

32. Nishimura O, Hara Y and Kuraku S. gVolante for standardizing completeness assessment of genome and transcriptome assemblies. Bioinformatics. 2017;33 22:3635-7. doi:10.1093/bioinformatics/btx445.

33. Roach MJ, Schmidt SA and Borneman AR. Purge Haplotigs: allelic contig reassignment for third-gen diploid genome assemblies. BMC Bioinformatics. 2018;19 1:460. doi:10.1186/s12859-018-2485-7.

34. Kadota M, Hara Y, Tanaka K, Takagi W, Tanegashima C, Nishimura O, et al. CTCF binding landscape in jawless fish with reference to Hox cluster evolution. Sci Rep. 2017;7 1:4957. doi:10.1038/s41598-017-04506-x.

35. Ikeda T, Hikichi T, Miura H, Shibata H, Mitsunaga K, Yamada Y, et al. Srf destabilizes cellular identity by suppressing cell-type-specific gene expression programs. Nat Commun. 2018;9 1:1387. doi:10.1038/s41467-018-03748-1.

36. Kadota M, Nishimura O, Miura H, Tanaka K, Hiratani I, Kuraku S. iconHi-C

Protocol (ver. 1.0). protocols.io http://dx.doi.org/10.17504/protocols.io.4mjgu4n

37. Adey A, Morrison HG, Asan, Xun X, Kitzman JO, Turner EH, et al. Rapid, low-input, low-bias construction of shotgun fragment libraries by high-density in vitro transposition. Genome Biol. 2010;11 12:R119. doi:10.1186/gb-2010-11-12-r119.

38. Langmead B and Salzberg SL. Fast gapped-read alignment with Bowtie 2. Nat Methods. 2012;9 4:357-9. doi:10.1038/nmeth.1923.

39. Durand NC, Shamim MS, Machol I, Rao SS, Huntley MH, Lander ES, et al. Juicer Provides a One-Click System for Analyzing Loop-Resolution Hi-C Experiments. Cell Syst. 2016;3 1:95-8. doi:10.1016/j.cels.2016.07.002.

40. Li H and Durbin R. Fast and accurate short read alignment with Burrows-Wheeler transform. Bioinformatics. 2009;25 14:1754-60. doi:10.1093/bioinformatics/btp324.

41. Arima Hi-C mapping pipeline Github. 2019. https://github.com/ArimaGenomics/mapping_pipeline

42. Li H. A statistical framework for SNP calling, mutation discovery, association mapping and population genetical parameter estimation from sequencing data. Bioinformatics. 2011;27 21:2987-93. doi:10.1093/bioinformatics/btr509.

43. “Picard Toolkit.” 2019. Broad Institute, GitHub Repository.

http://broadinstitute.github.io/picard/; Broad Institute

44. Quinlan AR and Hall IM. BEDTools: a flexible suite of utilities for comparing genomic features. Bioinformatics. 2010;26 6:841-2. doi:10.1093/bioinformatics/btq033.

45. Simao FA, Waterhouse RM, Ioannidis P, Kriventseva EV and Zdobnov EM. BUSCO: assessing genome assembly and annotation completeness with single-copy orthologs. Bioinformatics. 2015;31 19:3210-2. doi:10.1093/bioinformatics/btv351.

46. Grabherr MG, Haas BJ, Yassour M, Levin JZ, Thompson DA, Amit I, et al. Full-length transcriptome assembly from RNA-Seq data without a reference genome. Nat Biotechnol. 2011;29 7:644-52. doi:10.1038/nbt.1883.

47. Wang M and Kong L. pblat: a multithread blat algorithm speeding up aligning sequences to genomes. BMC Bioinformatics. 2019;20 1:28. doi:10.1186/s12859-019-2597-8.

48. Ryan JF. Baa.pl: A tool to evaluate de novo genome assemblies with RNA transcripts. arXiv e-prints. 2013;arXiv:1309.2087.

49. Kent WJ. BLAT--the BLAST-like alignment tool. Genome Res. 2002;12 4:656-64. doi:10.1101/gr.229202.

50. Kadota M, Nishimura O, Miura H, Tanaka K, Hiratani I, Kuraku S. Softshell turtle genome assemblies scaffolded with Hi-C data. *Figshare*. 2019, http://dx.doi.org/10.6084/m9.figshare.8024858.v2

51. Kadota M, Nishimura O, Miura H, Tanaka K, Hiratani I, Kuraku S. Supporting data for "Multifaceted Hi-C benchmarking: what makes a difference in chromosome-scale genome scaffolding?" *GigaScience Database*. 2019, http://dx.doi.org/10.5524/100675

52. Imakaev M, Fudenberg G, McCord RP, Naumova N, Goloborodko A, Lajoie BR, et al. Iterative correction of Hi-C data reveals hallmarks of chromosome organization. Nat Methods. 2012;9 10:999-1003. doi:10.1038/nmeth.2148.

**Table 1:** Overview of the specification of major scaffolding programs.

| Program | Support and availability | Input data requirement | Other information | Literature |
|---|---|---|---|---|
| LACHESIS | Developer's support discontinued; intricate installation | Generic bam format | No function to correct scaffold misjoins | [4] |
| HiRise | Open source version at GitHub not updated since 2015 | Generic bam format | Employed in Dovetail Chicago/Hi-C service. Default input sequence length cut-off=1000 bp | [8] |
| 3d-dna | Actively maintained and supported by the developer | Not compatible with multiple enzymes; Accept only Juicer mapper format | Default parameters: -t 15000 (input sequence length cut-off), -r 2 (no. of iterations for misjoin correction) | [11, 39] |
| SALSA2 | Actively maintained and supported by the developer | Compatible with multiple enzymes; generic bam (bed) file, assembly graph, unitig, 10x link files | Default parameters: -c 1000 (input sequence length cut-off), -i 3 (no. of iterations for misjoin correction) | [9, 10] |

**Figure legends**

**Figure 1**: Hi-C library preparation. (A) Basic procedure. (B) Comparison of Hi-C library preparation methods. Only the major differences between the methods are included here. The versions of the Arima and Phase kits used in this study are presented. The KAPA Hyper Prep Kit (KAPA Biosystems) is assumed to be conjunctly used with Arima Hi-C Kit, among the several specified kits. See Supplementary Protocol S1 for the full version of the iconHi-C protocol which was derived from the protocols published previously [3, 26, 35].

**Figure 2**: A juvenile softshell turtle *Pelodiscus sinensis*.

**Figure 3**: Structure of the Hi-C DNA and principle of the quality controls. (A) Schematic representation of the library preparation workflow based on HindIII or DpnII digestion. The patterns of restriction are indicated by the green lines. The nucleotides that are filled in are indicated by the letters in red. (B) Size shift analysis of HindIII-digested Hi-C DNA (QC1). Representative images of qualified (Sample 1) and disqualified (Sample 2) samples are shown. (C) Size shift analysis of the HindIII-digested Hi-C library (QC2). Representative images of the qualified (Sample 1) and disqualified (Sample 2) samples are shown. Size distributions were measured with Agilent 4200 TapeStation.

**Figure 4**: Post-sequencing quality control of Hi-C reads. Read pairs were categorized into valid and invalid pairs by HiC-Pro, based on their status in the mapping to the

reference genome (see Methods). This figure was adapted from the article that described HiC-Pro originally [25].

**Figure 5**: Effect of cell fixation duration. (A) QC1 of the HindIII-digested Hi-C DNA of human GM12878 cells fixed for 10 or 30 minutes in 1% formaldehyde. (B) QC2 of the HindIII-digested library of human GM12878 cells. (C) Quality control of the sequence reads by HiC-Pro using 1 M read pairs. See Fig. 4 for the details of the read pair categorization. See Supplementary Table S3 for the actual proportion of the reads in each category. (D) Contact probability measured by the ratio of observed and expected frequencies of Hi-C read pairs mapped along the same chromosome [52].

**Figure 6**: Testing varying durations of restriction and ligation. The length distributions of the DNA molecules prepared from human GM12878 cells after restriction and ligation of variable duration are shown. The size distributions of the HindIII-digested samples (top) and DpnII-digested samples (bottom) were measured with an Agilent 4200 TapeStation and an Agilent Bioanalyzer, respectively.

**Figure 7**: Softshell turtle Hi-C libraries prepared for our methodological comparison. (A) Lineup of the prepared libraries. This chart includes only the conditions in preparation methods that varied between these libraries, and the remainder preparation workflows are described in Supplementary Protocol S1 for the non-commercial (‘iconHi-C’) protocol and in the manuals of the commercial kits. (B) Quality control of Hi-C DNA (QC1) for Library c and d. The Hi-C DNA for the Chinese softshell turtle liver sample was prepared with either HindIII or DpnII digestion. (C) Quality control of

Hi-C libraries (QC2). The HindIII library prepared from the softshell turtle liver was digested by NheI, and the DpnII library was digested by ClaI (see Fig. 3 for the technical principle). See Supplementary Fig. S2 for the QC1 and QC2 results of the samples prepared from the blood of this species. See Supplementary Fig. S3 for the QC2 result of the Phase libraries.

**Figure 8**: Results of the post-sequencing quality control with HiC-Pro. One million read pairs were used for computation with HiC-Pro. See Fig. 7A for the preparation conditions of Library a-h, Fig. 4 for the categorization, and Supplementary Table S5 for the actual proportion of the reads in each category. The post-sequencing quality control using variable read amounts (500 K to 200 M pairs) for one of these softshell turtle libraries (Supplementary Table S9) and human GM12878 libraries (Supplementary Table S2) shows the validity of this quality control with as few as 500 K read pairs.

**Figure 9**: Comparison of Hi-C scaffolding products. (A) Scaffolding conditions used to produce Assembly 1 to 22. The default parameters are shown in red. (B) Scaffold length distributions. (C) Gene space completeness. (D) Largest and N50 scaffold lengths. See the panel A for Library IDs and Supplementary Table S6 for raw values of the metrics shown in B–D.

**Figure 10**: Cytogenetic validation of Hi-C scaffolding results. For the scaffolded sequences of Assembly 3, 7, and 9, we evaluated the consistency of the positions of the selected genes that were previously localized on eight macrochromosomes and Z

chromosome (A) and microchromosomes (B) by chromosome FISH [18-22] (see Results). Concordant and discordant gene locations on individual assemblies are indicated with blue and red boxes, respectively. The arrays of genes without idiograms in B were identified on chromosomes that are cytogenetically indistinguishable from each other.

Figure 1 Click here to access/download;Figure;Fig1.pdf

A

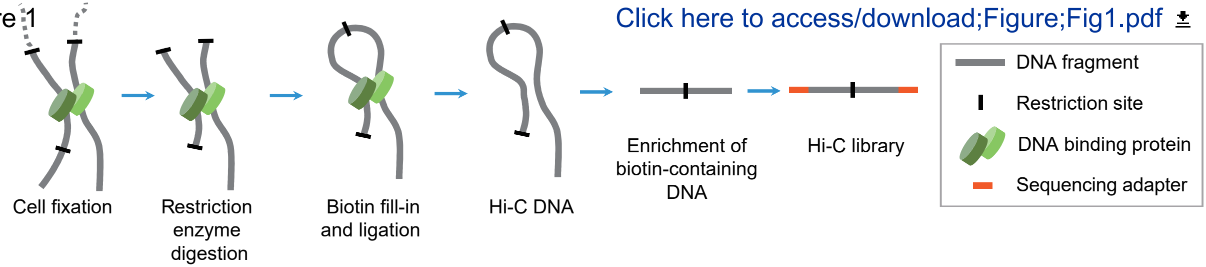


B

| Different specifications | iconHi-C (Our protocol) | Arima-HiC Kit (ver. A160107 v00, with the KAPA Hyper Prep Kit) | Phase Proximo Hi-C Kit (Animal ver. 1.0) | Dovetail Hi-C Kit (ver. 1.4, with Dovetail Library Module and Primer Set) |
|---|---|---|---|---|
| **Cell fixation** | 10 min (cells) or 15 min (tissue) in 1 % formaldehyde at 25°C; up to $1\times10^7$ cells or up to 1 $cm^3$ tissue | 10 min (cells) or 20 min (tissue) in 2 % formaldehyde at RT; 0.5-1 $\times10^7$ cells or 100-500 mg tissue | 15 min in crosslinking solution (included in the kit) at RT; $1\times10^7$ cells or 100 mg tissue | 20 min in 1.5 % formaldehyde at RT; $0.5\times10^6$ cells and 20-40 mg tissue |
| **Sample amount for restriction digestion and ligation** | 1-$2\times10^6$ cells or tissue estimated to contain 2-10 μg DNA | Cells or tissue estimated to contain 750 ng - 5 μg DNA | $1\times10^7$ cells or 100 mg tissue | $0.5\times10^6$ cells or 20-40 mg tissue |
| **Restriction enzyme digestion** | HindIII (cuts at "AAGCTT") or DpnII (cuts at "GATC"), 16 hrs at 37°C | Cocktail of A1 and A2 enzymes (cuts at "GATC" and "GANTC"), 30-60 min at 37°C | Sau3AI (cuts at "GATC"), 1 hr at 37°C | DpnII (cuts at "GATC"), 1 hr at 37°C |
| **Ligation** | 6 hrs at 16°C | 15 min at RT | 4 hrs at RT | 1-16 hrs at 16°C |
| **Reverse crosslinking** | 16 hrs at 65°C | 1.5-16 hrs at 68°C | 1-16 hrs at 60°C | 45 min at 68°C |
| **Hi-C DNA extraction** | Phenol/chloroform extraction | DNA purification beads (e.g. AMPure XP) | Spin column (included in the kit) | SPRIselect beads |
| **Hi-C DNA QC** | Check for the size shift before and after ligation (QC1) | Check for the yield of biotin-labeled DNA (Arima-QC1) | Check for the DNA yield before proximity ligation | Check for the DNA yield |
| **DNA amount for library preparation** | 250 ng - 2 μg | 125 ng - 2 μg | N/A | 200 ng |
| **Removal of biotin from un-ligated DNA ends** | By T4 DNA polymerase | N/A | N/A | N/A |
| **DNA fragmentation** | Sonication (Covaris) | Sonication (Covaris or Diagenode) | Transposase | Sonication (Covaris or Diagenode) |
| **Library preparation** | Adapter ligation-based (KAPA LTP Library Prep Kit) | Adapter ligation-based | Transposase-based (included in the kit) | Adapter ligation-based |
| **PCR cycles** | Pre-determination by qPCR (KAPA Real-time Library Amplification Kit) | Pre-determination by qPCR (KAPA Library Quantification Kit; Arima-QC2) | 15 cycles | 11 cycles |
| **Size selection** | After DNA fragmentation | After DNA fragmentation | After PCR | After PCR |
| **Hi-C library QC** | Check for yield and size distribution; check for size shift by NheI or ClaI digestion (QC2) | Check for yield and size distribution | Check for yield and size distribution | Check for yield and size distribution |

Figure 2

Click here to access/download;Figu

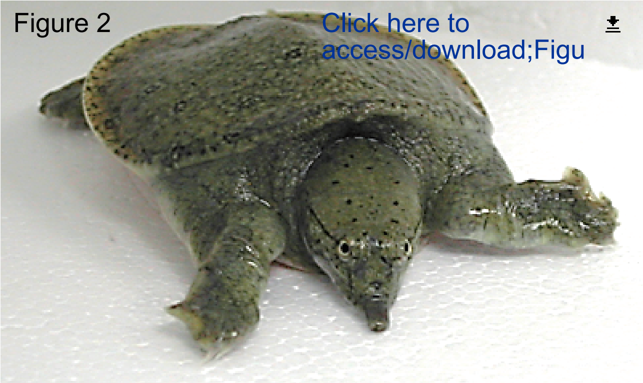

Figure 3

Click here to access/download;Figure

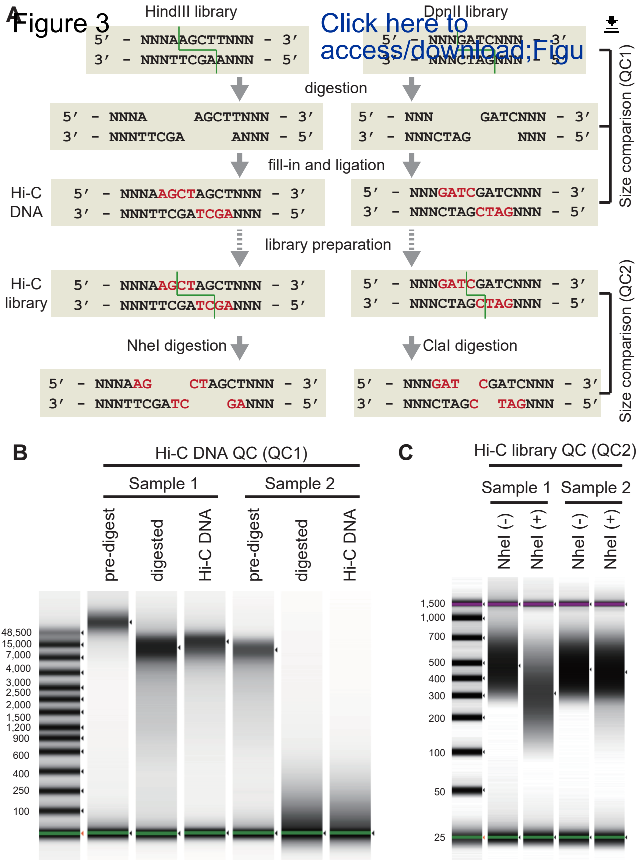

Figure 4

Click here to access/download;Figu

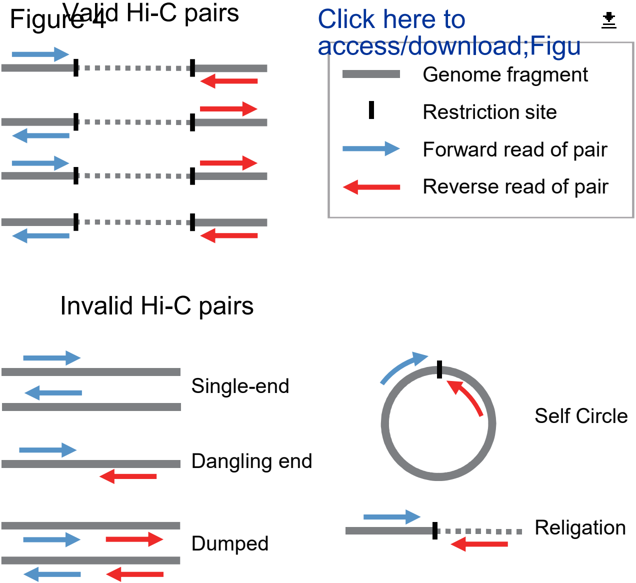

Figure 5

Click here to access/download;Figu

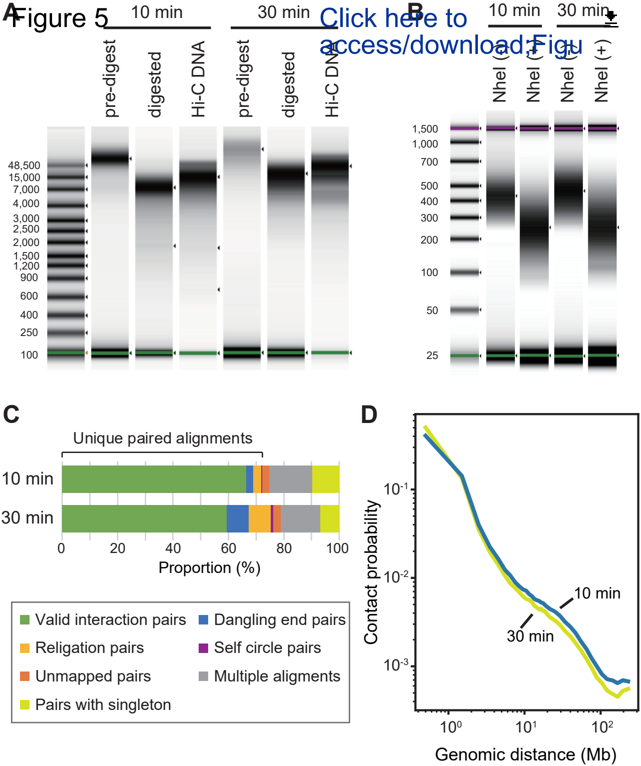

Click here to access/download;Figu

Figure 6

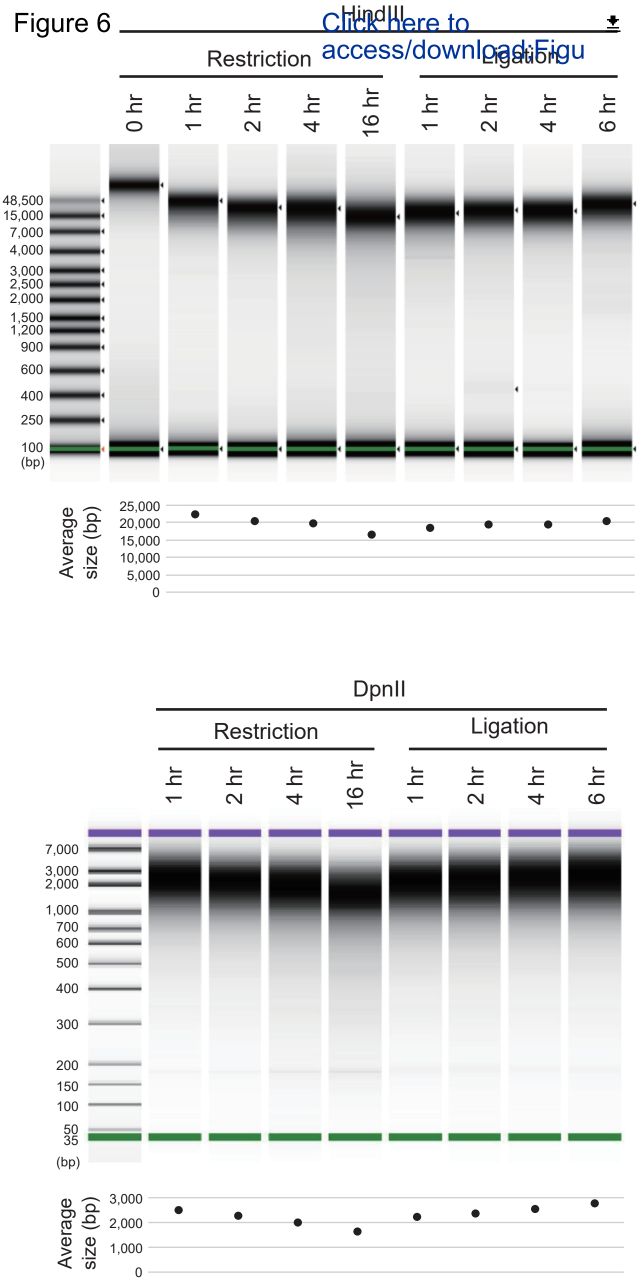

Figure 7

Click here to access/download;Figu

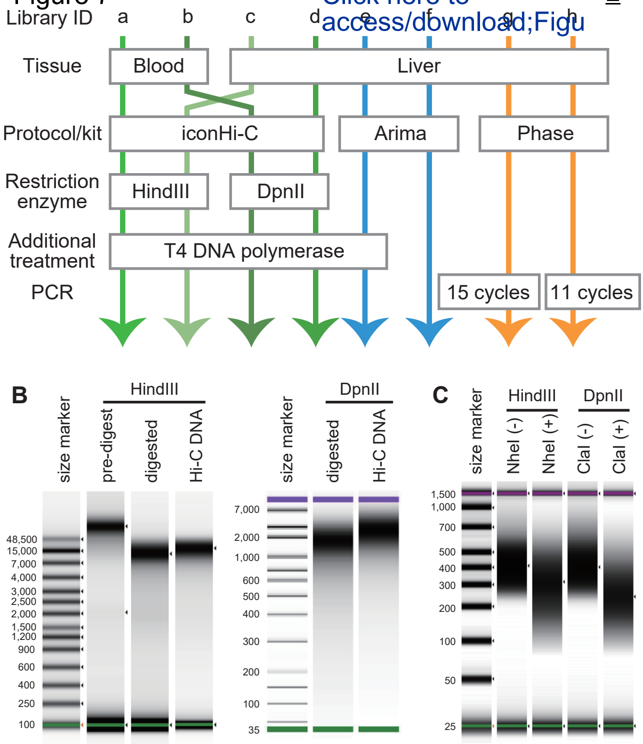

Figure 8

Click here to access/download;Figure;Fig8.pdf

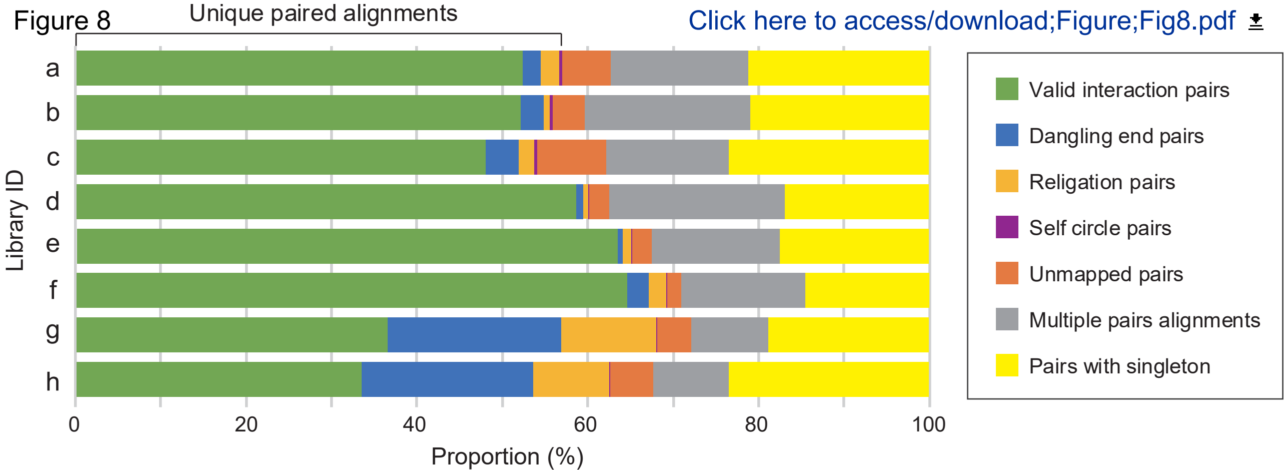

Click here to access/download;Figure;Fig9.pdf

Figure 9

**A**

| Assembly ID | Library ID | Scaffolding program | Input sequence length cutoff (nt) | Number of iterative misjoin correction rounds | Number of read pairs input |
|---|---|---|---|---|---|
| 1 | c | 3d-dna | 15000 | 2 | 200 M |
| 2 | a | | | | |
| 3 | d | | | | |
| 4 | b | | | | |
| 5 | c | SALSA2 | 1000 | 3 | |
| 6 | d | | | | |
| 7 | c + d | 3d-dna | 15000 | 2 | |
| 8 | b + d | | | | |
| 9 | e | | | | |
| 10 | | SALSA2 | 1000 | 3 | |
| 11 | h | 3d-dna | 15000 | 2 | |
| 12 | | SALSA2 | 1000 | 3 | |
| 13 | d | 3d-dna | 15000 | 4 | |
| 14 | | | | 6 | |
| 15 | | | 10000 | 2 | |
| 16 | | | 5000 | | |
| 17 | | | 3000 | | |
| 18 | | | 15000 | | 280 M |
| 19 | | | | | 160 M |
| 20 | | | | | 80 M |
| 21 | | | | | 20 M |
| 22 | | | | | 10 M |

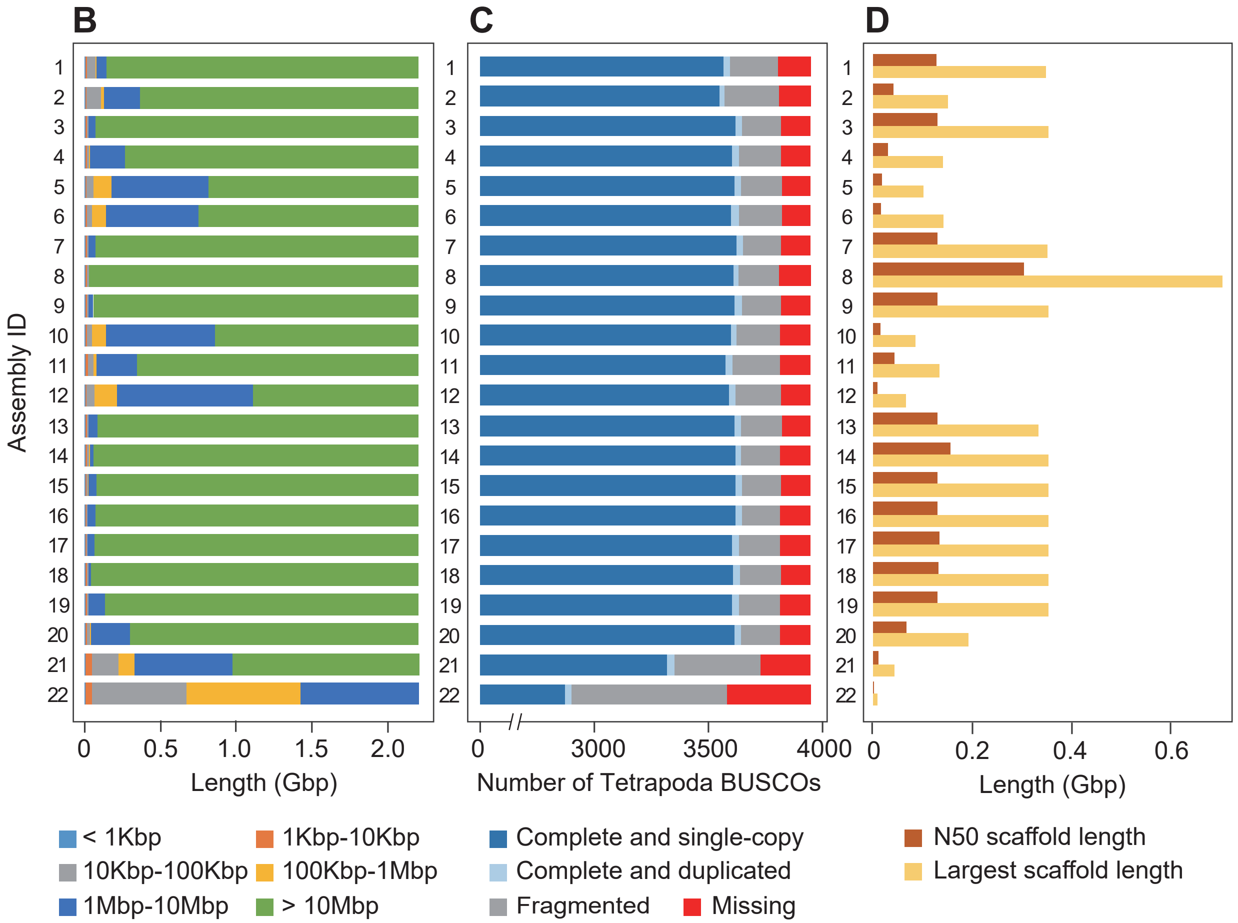

Figure 10

Click here to access/download;Figure;Fig10.pdf

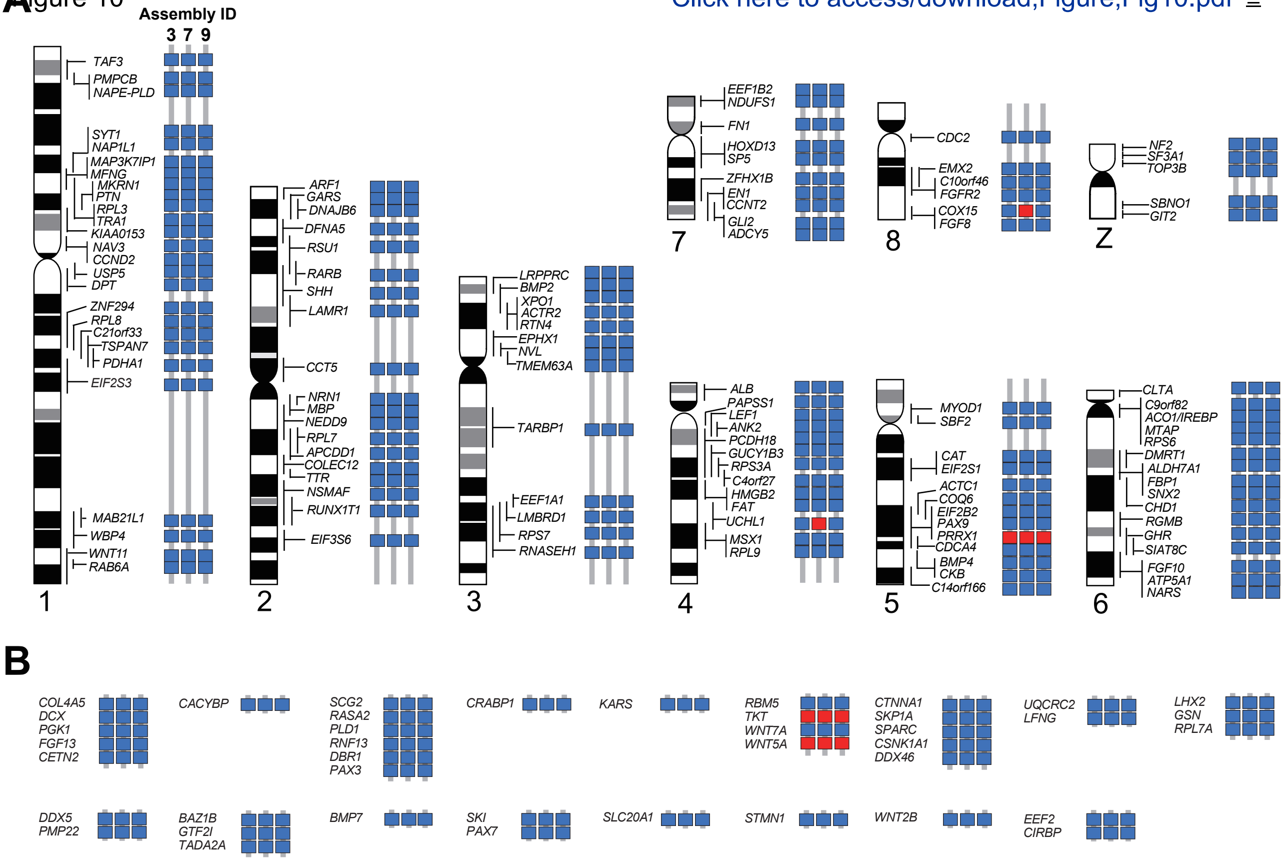

Click here to access/download
**Supplementary Material**
Supplementary_Figs_and_Tables.pdf

Click here to access/download
**Supplementary Material**
Supplementary_Protocol_S1_iconHi-C.pdf

Click here to access/download
**Supplementary Material**
Supplementary_Protocol_S2_to_support_multiple_enzymes.pdf

Cover letter

Click here to access/download;Personal Cover;CoverLetter_iconHi-C_rev.pdf

Shigehiro Kuraku, Ph.D.
Team Leader
Laboratory for Phyloinformatics
RIKEN Center for Biosystems Dynamics Research (BDR)

Tel: +81-(0)-78-306-3331
Email: shigehiro.kuraku@riken.jp
URL: https://www.bdr.riken.jp/en/research/labs/kuraku-s/

November 11, 2019

*GigaScience*

Dear Dr. Hongling Zhou,

Thank you very much for your handling our manuscript entitled, '***Multifaceted Hi-C benchmarking: what makes a difference in chromosome-scale genome scaffolding?***' by **Kadota, Nishimura, *et al.*** to be considered for publication in the journal *GigaScience*. We are pleased to see supportive reaction from you and the reviewers Following the residual comments from Reviewer #2, we have revised the manuscript. We hope that you will find our manuscript ready for publication in *GigaScience*.

Sincerely yours,

工樂樹洋

Shigehiro Kuraku, Ph.D.

**Request from the editor**:
In addition, please register any new software application in the bio.tools and SciCrunch.org databases to receive RRID (Research Resource Identification Initiative ID) and biotoolsID identifiers, and include these in your manuscript. This will facilitate tracking, reproducibility and re-use of your tool.

Regarding this request, our manuscript does not include any software application. We provide a short script to adapt Arima Hi-C data to the Juicer program (Supplementary Protocol S2), but this is not going to be updated in the future and guarantees full reproducibility and re-use as it is.

**Reviewer #2**:
Summary: I was impressed with the authors' tests on PCR overamplification and assembly quality. These have addressed many of my concerns with the previous manuscript, so my remaining concerns are minor.

We appreciate the reviewer's repeated assessment of our manuscript.

Line 323: The authors' tests of PCR overamplification bias have allayed many of my concerns. I still think that the interpretation of the data in this sentence could be couched in more caution. The Arima libraries had 10% more valid interaction pairs than the Icon-HI-C prep. Why was this?

We have not reached any understanding of what exactly contributed to the larger proportion of valid interaction pairs with the Arima kit, but it is possible that the most obvious characteristic of the Arima kit, namely the multiplicity of restriction enzymes, contributed to the larger proportion of valid interaction pairs. To suggest this possibility, we have inserted a sentence below in front of the sentence in question.

'*The increase of restriction site combinations might have possibly contributed to the larger proportion of valid interaction pairs (Fig. 8)*.'

Line 435: I still believe that this paragraph is gratuitous. I would be satisfied if the authors shortened this by two sentences and made the point that Hi-C scaffolding software does not provide consistent gap lengths for gaps of unknown length.

We have deleted the second half of this paragraph to satisfy this reviewer's suggestion as below.

*'The total length of assembly sequences is expected to increase after Hi-C scaffolding, because scaffolding programs simply insert a stretch of the unassigned base 'N' with a uniform length between input sequences in most cases (500 bp as a default in both 3d-dna and SALSA2). However, this has a minor impact on the total length of assembled sequences. ~~In fact, the insertion of 'N' stretches with an arbitrary length has been an implicit, rampant practice even before Hi-C scaffolding prevailed—for~~*

~~*example, the most and second most frequent lengths of the 'N' stretch in the publicly available zebrafish genome assembly Zv10 are 100 and 10 bp, respectively.*~~*'*

Supplementary table S8: Please provide captions that explain the difference between libraries "g" and "h" in the table as this is not immediately clear without referring to the main text.

In the previously submitted manuscript, we included a line showing the number of PCR cycles in this table (shown in red below). Also, we have inserted a guide to Figure 7A in the footnote as shown below in green.

**Supplementary Table S8:** Effect of variable degrees of PCR amplification

| **Library preparation condition** | **Library ID** | |
|---|---|---|
| | **g** | **h** |
| Tissue type | Liver | |
| Restriction enzyme | Sau3AI | |
| Number of PCR cycles | 15 | 11 |

| **Hi-C Pro results** | | |
|---|---|---|
| Number of input read pairs | 200,000,000 | |
| Category | Proportion of valid interaction (%) | |
| Valid interaction after removing duplicates | 55.1 | 70.4 |

See Figure 7A for the detail of the library preparation procedure. Note that 'trans' and 'cis' interactions mean contacts between scaffolds and those within scaffolds,

# Multifaceted Hi-C benchmarking: what makes a difference in chromosome-scale genome scaffolding?

Mitsutaka Kadota[1*], Osamu Nishimura[1*], Hisashi Miura[2], Kaori Tanaka[1,3], Ichiro Hiratani[2], and Shigehiro Kuraku[1]

[1] Laboratory for Phyloinformatics, RIKEN Center for Biosystems Dynamics Research (BDR), Kobe, 650-0047, Japan, [2] Laboratory for Developmental Epigenetics, RIKEN BDR, Kobe, 650-0047, Japan, [3] Present address: Division of Transcriptomics, Medical Institute of Bioregulation, Kyushu University, Fukuoka, 812-0054, Japan

*These authors contributed equally to this study.

Correspondence address. Shigehiro Kuraku, Laboratory for Phyloinformatics, RIKEN BDR, Japan. Tel: +81 78 306 3048; Fax: +81 78 306 3048; E-mail: shigehiro.kuraku@riken.jp

**Abstract**

**Background:** Hi-C is derived from chromosome conformation capture (3C) and targets chromatin contacts on a genomic scale. This method has also been used frequently in scaffolding nucleotide sequences obtained by *de novo* genome sequencing and assembly, in which the number of resultant sequences rarely converges to the chromosome number. Despite its prevalent use, the sample preparation methods for Hi-C have not been intensively discussed, especially from the standpoint of genome scaffolding.

**Results:** To gain insight into the best practice of Hi-C scaffolding, we performed a multifaceted methodological comparison using vertebrate samples and optimized various factors during sample preparation, sequencing, and computation. As a result, we identified several key factors that helped improve Hi-C scaffolding, including the choice and preparation of tissues, library preparation conditions, the choice of restriction enzyme(s), and the choice of scaffolding program and its usage.

**Conclusions:** This study provides the first comparison of multiple sample preparation kits/protocols and computational programs for Hi-C scaffolding by an academic third party. We introduce a customized protocol designated ‘inexpensive and controllable Hi-C (iconHi-C) protocol’, which incorporates the optimal conditions identified in this study, and demonstrated this technique on chromosome-scale genome sequences of the Chinese softshell turtle *Pelodiscus sinensis*.

**Keywords:** Hi-C, genome scaffolding, chromosomes, proximity-guided assembly, softshell turtle

## Background

Chromatin, a complex of nucleic acids (DNA and RNA) and proteins, exhibits a complex three-dimensional organization in the nucleus, which enables the intricate regulation of the expression of genome information via spatio-temporal control (reviewed in [1]). To characterize chromatin conformation on a genomic scale, the Hi-C method was introduced as a derivative of chromosome conformation capture (3C) (Fig. 1A; [2]). This method detects chromatin contacts on a genomic scale via the digestion of cross-linked DNA molecules with restriction enzymes, followed by proximity ligation of the digested DNA molecules. Massively parallel sequencing of the library containing ligated DNA molecules enables the comprehensive quantification of contacts both within and between chromosomes, which is presented in a heatmap that is conventionally called the ‘contact map’ [3].

Analyses of chromatin conformation using Hi-C have revealed more frequent contacts between more closely linked genomic regions, which has recently prompted the use of this method in scaffolding *de novo* genome sequences [4-6]. In *de novo* genome sequencing, the number of assembled sequences is usually far larger than the number of chromosomes in the karyotype of the species of interest, regardless of the sequencing platform chosen [7]. The application of Hi-C scaffolding enabled a remarkable enhancement of sequence continuity to reach a chromosome scale, and the integration of fragmentary sequences into longer sequences, which are similar in number to that of chromosomes in the karyotype.

In early 2018, commercial Hi-C library preparation kits were introduced (Fig. 1B), and *de novo* genome assembly was revolutionized by the release of versatile computational programs for Hi-C scaffolding (Table 1), namely LACHESIS [4], HiRise

[8], SALSA [9, 10], and 3d-dna [11] (reviewed in [12]). These movements assisted the rise of mass sequencing projects targeting a number of species, such as the Earth BioGenome Project (EBP) [13], the Genome 10K (G10K)/Vertebrate Genome Project (VGP) [14], and the DNA Zoo Project [15]. Optimization of Hi-C sample preparation, however, has been limited [16], which leaves room for the improvement of efficiency and the reduction of required sample quantity. Thus, the specific factors that are key for Hi-C scaffolding remain unexplored, mainly because of the costly and resource-demanding nature of this technology.

In addition to performing protocol optimization using human culture cells, we focused on the softshell turtle *Pelodiscus sinensis* (Fig. 2). This species has been adopted as a study system for evolutionary developmental biology (Evo-Devo), including the study of the formation of the dorsal shell (carapace) (reviewed in [17]). Access to genome sequences of optimal quality by relevant research communities is desirable in this field. In Japan, live materials (adults and embryos) of this species are available through local farms mainly between May and August, which implies its high utility for sustainable research. A previous cytogenetic report revealed that the karyotype of this species consists of 33 chromosome pairs including Z and W chromosomes (2n = 66) that show a wide variety of sizes (conventionally categorized as macrochromosomes and microchromosomes) [18]. Despite the moderate global GC-content in its whole genome at around 44%, the intragenomic heterogeneity of GC-content between and within the chromosomes has been suggested [19]. A wealth of cytogenetic efforts on this species led to the accumulation of fluorescence *in situ* hybridization (FISH)-based mapping data for 162 protein-coding genes covering almost all chromosomes [18-22], which serve as structural landmarks for validating genome

assembly sequences.

A draft sequence assembly of the softshell turtle genome was built using short reads and was released in 2013 [23]. This sequence assembly achieved the N50 scaffold length of >3.3 Mb but remains fragmented into approximately 20,000 sequences (see Supplementary Table S1). The longest sequence in this assembly is only slightly larger than 16 Mb, which is much shorter than the largest chromosome size estimated from the karyotype report [18]. The total size of the assembly is approximately 2.2 Gb, which is a moderate size for a vertebrate species. Because of the affordable genome size, sufficiently complex structure, and availability of validation methods, we reasoned that the genome of this species is a suitable target for our methodological comparison, and its improved genome assembly is expected to assist a wide range of genome-based studies of this species.

## Results

### Stepwise QC prior to large-scale sequencing

The assessment of the quality of prepared libraries before engaging in costly sequencing would be ideal. According to the literature [16, 24], we routinely control the quality of Hi-C DNAs and Hi-C libraries by observing DNA size shifts via digestion targeting the restriction sites in properly prepared samples (Fig. 3). More concretely, a successfully ligated Hi-C DNA sample should exhibit a slight increase in the length of its restricted DNA fragments after ligation (QC1), which serves as an indicator of qualified samples (e.g., Sample 1 in Fig. 3B). In contrast, an unsuccessfully prepared Hi-C DNA does not

exhibit this length recovery (e.g., Sample 2 in Fig. 3B). In a subsequent step, DNA molecules in a successfully prepared HindIII-digested Hi-C library should contain the NheI restriction site at a high probability. Thus, the length distribution observed after NheI digestion of the prepared library serves as an indicator of qualified or disqualified products (QC2; Fig. 3C). This series of QCs is incorporated into our protocol by default (Supplementary Protocol S1) and can also be performed in combination with sample preparation using commercial kits if it employs a single restriction enzyme.

Some of the libraries prepared by us passed the QC steps performed before sequencing but yielded an unfavourably large proportion of invalid read pairs. To identify such libraries, we routinely performed small-scale sequencing for quick and inexpensive QC (designated ‘QC3’) using the HiC-Pro program [25] (see Fig. 4 for the read pair categories assigned by HiC-Pro). Our test using variable input data sizes (500 K to 200 M read pairs) resulted in highly similar breakdowns into different categories of read pair properties (Supplementary Table S2) and guaranteed QC3 with an extremely small data size of 1 M or fewer reads. These post-sequencing QC steps, which do not incur a large cost, are expected to help avoid the large-scale sequencing of unsuccessful libraries that have somehow passed through the QC1 and QC2 steps. Importantly, libraries that have passed QC3 can be further sequenced with greater depth, as necessary.

**Optimization of sample preparation conditions**

We identified overt differences between the sample preparation protocols of published studies and those of commercial kits, especially regarding the duration of fixation and enzymatic reaction as well as the library preparation method used. (Fig. 1B). Therefore,

we first sought to optimize the conditions of several of these steps using human culture cells.

To evaluate the effect of the degree of cell fixation, we prepared Hi-C libraries from GM12878 cells fixed for 10 and 30 minutes. Our comparison did not detect any marked differences in the quality of the Hi-C DNA (QC1; Fig. 5A) and Hi-C library (QC2; Fig. 5B). However, libraries that were prepared with a longer fixation time exhibited a larger proportion of dangling end read pairs and religation read pairs, as well as a smaller proportion of valid interaction reads (Fig. 5C). The increase in the duration of cell fixation also reduced the proportion of long-range (>1 Mb) interactions among the overall captured interactions (Fig. 5D).

The reduced preparation time of commercial Hi-C kits (up to two days according to their advertisement) is attributable mainly to shortened restriction and ligation times (Fig. 1B). To monitor the effect of shortening these enzymatic reactions, we first analysed the progression of restriction and ligation in a time-course experiment using GM12878 cells. We observed the persistent progression of restriction up to 16 hours and of ligation up to 6 hours (Fig. 6). To scrutinize further the possible adverse effects of the prolonged reaction, Hi-C libraries of GM12878 cells were prepared with variable durations of restriction digestion (1 hour and 16 hours) and ligation (15 minutes, 1 hour, and 6 hours). We found that the proportions of dangling end and religation read pairs were reduced in cases with an extended duration of restriction digestion  (Supplementary Table S4). The yield of the library, which can be estimated from the number of PCR cycles, increased with the extended duration of ligation without any effect on the proportion of valid interaction read pairs (Supplementary Table S4). The proportion of valid interaction read pairs containing the proper DpnII

junction sequence ‘GATCGATC’ also remained unchanged, suggesting that the prolonged reaction times did not induce any adverse effects, such as star activity of the restriction enzyme.

## Multifaceted comparison using softshell turtle samples

Based on the detailed optimization of the sample preparation conditions described above, we built an original protocol, designated the ‘iconHi-C protocol’, that included a 10 minute-long cell fixation, 16 hour-long restriction, 6 hour-long ligation, and successive QC steps (Methods; also see Supplementary Protocol S1; Fig. 1B).

We performed Hi-C sample preparation and scaffolding using tissues from a female Chinese softshell turtle which has both Z and W chromosomes [18]. We prepared Hi-C libraries using various tissues (liver or blood cells), restriction enzymes (HindIII or DpnII), and protocols (our iconHi-C protocol, the Arima kit in conjunction with the KAPA Hyper Prep Kit, or the Phase kit), as outlined in Fig. 7A (see Supplementary Table S5; Supplementary Fig. S1). As in some of the existing protocols (e.g. [26]), we performed T4 DNA polymerase treatment in our iconHi-C protocol (Library a–d), expecting reduced proportions of ‘dangling end’ read pairs that contain no ligated junction, and thus do not contribute to Hi-C scaffolding. We also incorporated this T4 DNA polymerase treatment into the workflow of the Arima kit (Library e vs. Library f without this additional treatment). Furthermore, we tested a lesser degree of PCR amplification (11 cycles) together with the use of the Phase kit which recommends as many as 15 cycles by default (Library h vs. Library g; Fig. 7A).

All samples prepared using the iconHi-C protocol passed both controls, QC1 and QC2 (Fig. 7B). The prepared Hi-C libraries were sequenced to obtain one million

127 nt-long read pairs and were subjected to QC3 using the HiC-Pro program (Fig. 8). As a result of this QC3, the largest proportion of ‘valid interaction’ pairs was observed for Arima libraries (Library e and f). Regarding the iconHi-C libraries (Library a–d), fewer ‘unmapped’ and ‘religation’ pairs were detected for the DpnII libraries compared with HindIII libraries. It should be noted that the QC3 of the softshell turtle libraries generally produced lower proportions of the ‘valid interaction’ category and larger proportions of ‘unmapped pairs’ and ‘pairs with singleton’ than with the human libraries. This cross-species difference may be attributable to the use of incomplete genome sequences as a reference for Hi-C read mapping (Supplementary Table S1). This invokes a caution when comparing QC results across species.

**Scaffolding using variable input and computational conditions**

In this study, only well-maintained open-source programs, i.e., 3d-dna and SALSA2, were used in conjunction with variable combinations of input libraries, input read amounts, input sequence cut-off lengths, and number of iterative misjoin correction rounds (Fig. 9A). As a result of scaffolding, we observed a wide spectrum of basic metrics, including the N50 scaffold length (0.6–303 Mb), the largest scaffold length (8.7–703 Mb), and the number of chromosome-sized (>10 Mb) sequences (0–65) (Fig. 9; Supplementary Table S6).

First, using the default parameters, 3d-dna consistently produced more continuous assemblies than did SALSA2 (see Assembly 1 vs. 5, 3 vs. 6, 9 vs. 10, and 11 vs. 12 in Fig. 9). Second, the increase in the number of iterative corrections (‘-r’ option of 3d-dna) resulted in relatively large N50 lengths, but with more missing orthologues (see Assembly 3 and 13–14). Third, a smaller input sequence cut-off length (‘-i’ option

of 3d-dna) resulted in a smaller number of scaffolds but again, with more missing orthologues (see Assembly 3 and 15–17). Fourth, the use of the liver libraries consistently resulted in a higher continuity than the use of the blood cell libraries (see Assembly 1 vs. 2 and 3 vs. 4 in Fig. 9).

Assembly 8, which resulted from input Hi-C reads derived from both liver and blood, exhibited an outstandingly large N50 scaffold length (303 Mb) but a larger number of undetected reference orthologues (141 orthologues) than most of the other assemblies. The largest scaffold (scaffold 5) in this assembly is approximately 703 Mb long, causing a large N50 length, and accounts for approximately one-third of the whole genome in length, as a result of possible chimeric assembly that bridged 14 putative chromosomes (see Supplementary Fig. S4).

The choice of restriction enzymes has not been discussed in depth in the context of genome scaffolding. Here, we prepared Hi-C libraries separately with HindIII and DpnII. We did not mix multiple enzymes in the same reaction (other than using the Arima kit which originally employs two enzymes); rather, we performed a single scaffolding run with both HindIII-based and DpnII-based reads (see Assembly 7 in Fig. 9). As expected, our comparison of multiple metrics yielded a more successful result with DpnII than with HindIII (see Assembly 1 vs. 3 as well as 2 vs. 4; Fig. 9). However, the mixed input of HindIII-based and DpnII-based reads did not necessarily yield a better scaffolding result (see Assembly 3 vs. 7).

To gain additional insight regarding the evaluation of the scaffolding results, we assessed the contact maps constructed upon the Hi-C scaffolds (Supplementary Fig. S5). The comparison of Assembly 3, 9 and 11, which represent the three different preparation methods, revealed anomalous patterns, particularly for Assembly 11, with

intensive contact signals separated from the diagonal line that indicate the presence of errors in the scaffolds [15]. We also performed genome-wide alignments between the Hi-C scaffolds obtained. The comparison of Assembly 3, 9, and 11 revealed a high similarity between Assembly 3 and 9, while Assembly 11 exhibited a significantly larger number of inconsistencies against either of the other two assemblies (Supplementary Fig. S6). These observations are consistent with the evaluation based on sequence length and gene space completeness, which alone does not, however, provide a reliable metric for the assessment of the quality of scaffolding.

**Validation of scaffolding results using transcriptome and FISH data**

In addition to the above-mentioned evaluation of the scaffolding results, we assessed the sequence continuity using independently obtained data. First, we mapped assembled transcript sequences onto our Hi-C scaffold sequences (see Methods). This did not show any substantial differences between the assemblies (Supplementary Table S7), probably because the sequence continuity after Hi-C scaffolding exceeded that of RNA-seq library inserts, even when the length of intervening introns in the genome was considered. The present analysis with RNA-seq data did not provide an effective source of continuity validation.

Second, we referred to the fluorescence *in situ* hybridization (FISH) mapping data of 162 protein-coding genes from published cytogenetic studies [18-22], which allowed us to check the locations of those genes with our resultant Hi-C assemblies. In this analysis, we evaluated Assembly 3, 7, and 9 (see Fig. 9A) that showed better scaffolding results in terms of sequence length distribution and gene space completeness (Fig. 9D). As a result, we confirmed the positioning of almost all genes and their

continuity over the centromeres, which encompassed not only large but also small chromosomes (conventionally called ‘macrochromosomes’ and ‘microchromosomes’; Fig. 10). Two genes that were not confirmed by Assembly 7 (*UCHL1* and *COX15*; Fig. 10) were found in separate scaffold sequences that were shorter than 1 Mb, which indicates insufficient scaffolding. Conversely, the gene array including *RBM5*, *TKT*, *WNT7A*, and *WNT5A*, previously shown by FISH, was consistently unconfirmed by all three assemblies (Fig. 10), which did not provide any clues for among-assembly evaluation or perhaps indicates an erroneous interpretation of FISH data in a previous study.

## Discussion

### Starting material: not genomic DNA extraction but *in situ* cell fixation

In genome sequencing, best practices for high molecular weight DNA extraction have often been discussed (e.g. [27]). This factor is fundamental to building longer contigs, regardless of the use of short-read or long-read sequencing platforms. Moreover, the proximity ligation method using Chicago libraries provided by Dovetail Genomics which is based on *in vitro* chromatin reconstruction [8], uses genomic DNA as starting material. In contrast, proximity-guided assembly enabled by Hi-C employs cellular nuclei with preserved chromatin conformation, which brings a new technical challenge regarding appropriate sampling and sample preservation in genomics.

In the preparation of the starting material, it is important to optimize the degree of cell fixation depending on sample choice, to obtain an optimal result in Hi-C

scaffolding (Fig. 5). Another practical indication of tissue choice was obtained by examining Assembly 8 (Fig. 9A). This assembly was produced by 3d-dna scaffolding using both liver and blood libraries (Library b and d), which led to an unacceptable result possibly caused by over-assembly (Fig. 9B–D; also see Results). It is likely that increased cellular heterogeneity, which possibly introduces excessive conflicting chromatin contacts, did not allow the scaffolding program to group and order the input genome sequences properly. In brief, we recommend the use of samples with modest cell-type heterogeneity that are amenable to thorough fixation.

**Considerations regarding sample preparation**

In this study, we did not test all commercial Hi-C kits available in the market. This was partly because the Dovetail Hi-C kit specifies the non-open source program HiRise as the only supported downstream computation solution and does not allow a direct comparison with other kits, namely those from Phase Genomics and Arima Genomics.

According to our calculations, the preparation of a Hi-C library using the iconHi-C protocol would be at least three times cheaper than the use of a commercial kit. Practically, the cost difference would be even larger, either when the purchased kit is not fully consumed or when the post-sequencing computation steps cannot be undertaken in-house, which implies additional outsourcing costs.

The genomic regions that are targeted by Hi-C are determined by the choice of restriction enzymes. Theoretically, 4-base cutters (e.g. DpnII), which potentially have more frequent restriction sites on the genome, are expected to provide a higher resolution than 6-base cutters (e.g., HindIII) [16]. Obviously, the use of restriction enzymes that were not employed in this study might be promising in the adaptation of

the protocol to organisms with variable GC-content or methylation profiles. However, this might not be so straightforward when considering the interspecies variation in GC-content and the intra-genomic heterogeneity. The use of multiple enzymes in a single reaction is a promising approach; however, from a computational viewpoint, not all scaffolding programs are compatible with multiple enzymes (see Table 1 for a comparison of the specification of scaffolding programs). Another technical downside of this approach is the incompatibility of DNA ends restricted by multiple enzymes, with restriction-based QCs, such as the QC2 step of our iconHi-C protocol (Fig. 3). Therefore, in this study, DpnII and HindIII were used separately in the iconHi-C protocol, which resulted in a higher scaffolding performance with the DpnII library (Figs. 8 and 9), as expected. In addition, we input the separately prepared DpnII and HindIII libraries together in scaffolding (Assembly 7), but this approach did not lead to higher scaffolding performance (Figs. 9B–D and 10). The Arima kit employs two different enzymes that can produce a much greater number of restriction site combinations, because one of these two enzymes recognizes the nucleotide stretch ‘GANTC’. The increase of restriction site combinations might have possibly contributed to the larger proportion of valid interaction pairs (Fig. 8). Scaffolding with the libraries prepared using this kit resulted in one of the most acceptable assemblies (Assembly 9). However, this result did not explicitly exceed the performance of scaffolding with the iconHi-C libraries, including the one that used a single enzyme (DpnII; Library d).

Overamplification by PCR is a concern regarding the use of commercial kits (with the exception of the Arima kit used with the Arima-QC2) because their manuals specify the use of a certain number of PCR cycles *a priori* (15 cycles for the Phase kit

and 11 cycles for the Dovetail Hi-C kit) (Supplementary Table S8). In our iconHi-C protocol, an optimal number of PCR cycles is estimated by means of a preliminary real-time PCR using a small aliquot (Step 11.25 to 11.29 in Supplementary Protocol S1), as done traditionally for other library types (e.g., [28]). This procedure allowed us to reduce the number of PCR cycles, down to as few as five cycles (Supplementary Table S5). The Dovetail Hi-C kit recommends the use of larger amounts of kit components than that specified for a single sample, depending on the genome size, as well as the degree of genomic heterozygosity and repetitiveness, of the species of interest. In contrast, with our iconHi-C protocol, we always prepared a single library, regardless of those species-specific factors, which seemed to suffice in all the cases tested.

Commercial Hi-C kits, which usually advertise easiness and quickness of use, have largely shortened the protocol down to two days, compared with the published non-commercial protocols (e.g., [16, 26]). Such time-saving protocols are achieved mainly by shortening the duration of restriction enzyme digestion and ligation (Fig. 1B). Our assessment, however, revealed unsaturated reaction within the shortened time frames employed in the commercial kits (Fig. 6), which was accompanied by an unfavorable composition of read pairs (Supplementary Table S4). Our attempt to insert a step of T4 DNA polymerase treatment in the sample preparation of the Arima kit protocol resulted in reduced 'dangling end' reads (Library e vs. f in Fig. 8). Regarding the Phase kit, transposase-based library preparation contributes largely to its shortened protocol, but this does not allow flexible control of library insert lengths. Recent protocols (versions 1.5 and 2.0) of the Phase kit instruct users to employ a largely reduced DNA amount in the tagmentation reaction, which should mitigate the difficulty in controlling insert length but require excessive PCR amplification. The Arima and

Phase kits assume that the quality control of Hi-C DNA is based on the yield, and not the size, of DNA (see Fig. 1B). Nevertheless, quality control based on DNA size (equivalent to QC1 in iconHi-C) is feasible by taking aliquots at each step of sample preparation. In particular, if preparing a small number of samples for Hi-C, as practised typically for genome scaffolding, one should opt to consider these points, even when using commercial kits, to improve the quality of the prepared libraries and scaffolding products.

**Considerations regarding sequencing**

The quantity of Hi-C read pairs to be input for scaffolding is critical because it accounts for the majority of the cost of Hi-C scaffolding. Our protocol introduces a thorough safety system to prevent sequencing unsuccessful libraries, first by performing pre-sequencing QCs for size shift analyses (Fig. 3) and second via small-scale (down to 500 K read pairs) sequencing (see Results; also see Supplementary Tables S2 and S9).

Our comparison showed a dramatic decrease in assembly quality in cases in which <100 M read pairs were used (see the comparison of Assembly 18–22 described above; Fig. 9; also see [29]). Nevertheless, we obtained optimal results with a smaller number of reads (ca. 160 M per 2.2 Gb of genome) than that recommended by the manufacturers of commercial kits (e.g., 100 M per 1 Gb of genome for the Dovetail Hi-C kit and 200 M per Gb of genome for the Arima kit). As generally and repeatedly discussed [29][29], the proportion of informative reads and their diversity, rather than just the overall number of obtained reads, is critical.

In terms of read length, we did not perform any comparisons in this study. Longer reads may enhance the fidelity of the characterization of the read pair properties

and allow precise QC. Nevertheless, the existing Illumina sequencing platform has enabled the less expensive acquisition of 150 nt-long paired-end reads, which did not prompt us to vary the read length.

**Considerations regarding computation**

In this study, 3d-dna produced a more reliable scaffolding output than did SALSA2, whether sample preparation employed a single or multiple enzyme(s) (Fig. 9B–D). On the other hand, 3d-dna required a greater amount of time for the completion of scaffolding than did SALSA2. Apart from the choice of program, several points should be considered if successful scaffolding for a smaller investment is to be achieved. In general, Hi-C scaffolding results should not be taken for granted, and it is necessary to improve them by referring to contact maps using an interactive tool, such as Juicebox [15]. In this study, however, we compared raw scaffolding output to evaluate sample preparation and reproducible computational steps.

We used various parameters of the scaffolding programs (Fig. 9A). First, the Hi-C scaffolding programs that are available currently have different default length cut-off values for input sequences (e.g., 15000 bp for the '-i' parameter in 3d-dna and 1000 bp for the '-c' parameter in SALSA2). Only sequences that are longer than the cut-off length value contribute to sequence scaffolding towards chromosome sizes, while sequences shorter than the cut-off length are implicitly excluded from the scaffolding process and remain unchanged. Typically, when using the Illumina sequencing platform, genomic regions with unusually high frequencies of repetitive elements and GC-content are not assembled into sequences with a sufficient length (see [30]). Such genomic regions tend to be excluded from chromosome-scale Hi-C scaffolds because

their length is smaller than the threshold. Alternatively, these regions may be excluded because few Hi-C read pairs are mapped to them, even if they exceed the cut-off length. The deliberate setting of a cut-off length is recommended if particular sequences with relatively small lengths are the target of scaffolding. It should be noted that lowering the length threshold can result in frequent misjoins in the scaffolding output (Fig. 9B–D) or in overly long computational times. Regarding the number of iterative misjoin correction rounds (the '-r' parameter in 3d-dna and 'i' parameter in SALSA2), our attempts of using increased values did not necessarily yield favourable results (Fig. 9B–D). This did not provide a consistent optimal range of values but rather suggests the importance of performing multiple scaffolding runs with varying parameters.

**Considerations regarding the assessment of chromosome-scale genome sequences**

Our assessment using cytogenetic data confirmed the continuity of gene linkage over the obtained chromosome-scale sequences (Fig. 10). This validation was required by the almost saturated scores of typical gene space completeness assessment tools such as BUSCO (Supplementary Table S6) and by transcript contig mapping (Supplementary Table S7), neither of which provided an effective metric for evaluation.

For further evaluation of our scaffolding results, we referred to the sequence length distributions of the genome assemblies of other turtle species that are regarded as being chromosome-scale data. This analysis yielded values of the basic metrics that were comparable to those of our Hi-C scaffolds of the softshell turtle, i.e. an N50 length of 127.5 Mb and a maximum sequence length of 344.5 Mb for the genome assembly of the green sea turtle (*Chelonia mydas*) released by the DNA Zoo Project [15] and an N50 length of 131.6 Mb and a maximum length of 370.3 Mb for the genome assembly of the

Goode’s thornscrub tortoise (*Gopherus evgoodei*) released by the Vertebrate Genome Project (VGP) [14]. Scaffolding results should be evaluated by referring to the estimated N50 length and the maximum length based on the actual value and to the length distribution of chromosomes in the intrinsic karyotype of the species in question, or of its close relative. Turtles tend to have an N50 length of approximately 130 Mb and a maximum length of 350 Mb, while many teleost fish genomes exhibit an N50 length as low as 20–30 Mb and a maximum length of <100 Mb [31]. If these values are excessive, the scaffolded sequences harbour overassembly, which erroneously boosts length-based metrics. Thus, higher values, which are conventionally regarded as signs of successful sequence assembly, do not necessarily indicate higher precision.

The total length of assembly sequences is expected to increase after Hi-C scaffolding, because scaffolding programs simply insert a stretch of the unassigned base ‘N’ with a uniform length between input sequences in most cases (500 bp as a default in both 3d-dna and SALSA2). However, this has a minor impact on the total length of assembled sequences. ~~In fact, the insertion of ‘N’ stretches with an arbitrary length has been an implicit, rampant practice even before Hi-C scaffolding prevailed—for example, the most and second most frequent lengths of the ‘N’ stretch in the publicly available zebrafish genome assembly Zv10 are 100 and 10 bp, respectively.~~

## Conclusions

In this study, we introduced the iconHi-C protocol which implements successive QC steps. We also assessed potential key factors for improving Hi-C scaffolding. Overall, our study showed that small variations in sample preparation or computation for scaffolding can have a large impact on scaffolding output, and that any scaffolding

output should ideally be validated using independent information, such as cytogenetic data, long reads, or genetic linkage maps. The present study aimed to evaluate the output of reproducible computational steps, which in practice should be followed by the modification of the raw scaffolding output by referring to independent information or by analysing chromatin contact maps. The study employed limited combinations of species, sample prep methods, scaffolding programs, and its parameters, and we will continue to test different conditions for kits/programs that did not necessarily perform well here using our specific materials.

## Methods

### Initial genome assembly sequences

The softshell turtle (*Pelodiscus sinensis*) assembly published previously [23] was downloaded from NCBI GenBank (GCA_000230535.1), whose gene space completeness and length statistics were assessed by gVolante [32] (see Supplementary Table S1 for the assessment results). Although it could be suggested to remove haplotigs before Hi-C scaffolding [33], we omitted this step because of the low frequency of the reference orthologues with multiple copies (0.72%; Supplementary Table S1), indicating a minimal degree of haplotig contamination.

### Animals and cells

We sampled tissues (liver and blood cells) from a female purchased from a local farmer in Japan, because the previous whole genome sequencing used the whole blood of a female [23]. All experiments were conducted in accordance with the Guideline of the

Institutional Animal Care and Use Committee of RIKEN Kobe Branch (Approval ID: A2017-12).

The human lymphoblastoid cell line GM12878 was purchased from the Coriell Cell Repositories and cultured in RPMI-1640 medium (Thermo Fisher Scientific) supplemented with 15% FBS, 2 mM L-glutamine, and a 1× antibiotic-antimycotic solution (Thermo Fisher Scientific), at 37 °C, 5% $CO_2$, as described previously [34].

**Hi-C sample preparation using the original protocol**

We have made modifications to the protocols that are available in the literature [3, 26, 35] (Fig. 1B). The full version of our ‘inexpensive and controllable Hi-C (iconHi-C)’ protocol is described in Supplementary Protocol S1 and available at Protocols.io (https://www.protocols.io/private/950FFCBDE7C46D1598CA7DDFE7441C9F).

**Hi-C sample preparation using commercial kits**

The Proximo Hi-C kit (Phase Genomics) which employs the restriction enzyme Sau3A1 and transposase-based library preparation [36] (Fig. 1B) was used to prepare a library from 50 mg of the softshell turtle liver according to the official ver. 1.0 animal protocol provided by the manufacturer (Library g in Fig. 7A) and a library from 10 mg of the liver that was amplified with a reduced number of PCR cycles based on a preliminary real-time qPCR using an aliquot (Library h; see [28] for the details of the pre-determination of the optimal number of PCR cycles). The Arima-HiC kit (Arima Genomics), which employs a restriction enzyme cocktail (Fig. 1B), was used in conjunction with the KAPA Hyper Prep Kit (KAPA Biosystems), protocol ver. A160108 v00, to prepare a library using the softshell turtle liver, according to its official

animal vertebrate tissue protocol (ver. A160107 v00) (Library f) and a library with an additional step of T4 DNA polymerase treatment for reducing ‘dangling end’ reads (Library e). This additional treatment is detailed in Step 8.2 (for DpnII-digested samples) of Supplementary Protocol S1.

**DNA sequencing**

Small-scale sequencing for library QC (QC3) was performed in-house to obtain 127 nt-long paired-end reads on an Illumina HiSeq 1500 in the Rapid Run Mode. For evaluating the effects of variable duration of the restriction digestion and ligation reactions, sequencing was performed on an Illumina MiSeq using the MiSeq Reagent Kit v3 to obtain 300 nt-long paired-end reads. Large-scale sequencing for Hi-C scaffolding was performed to obtain 151 nt-long paired-end reads on an Illumina HiSeq X. The obtained reads underwent quality control using FastQC ver. 0.11.5 (https://www.bioinformatics.babraham.ac.uk/projects/fastqc/), and low-quality regions and adapter sequences in the reads were removed using Trim Galore ver. 0.4.5 (https://www.bioinformatics.babraham.ac.uk/projects/trim_galore/) with the parameters ‘-e 0.1 -q 30’.

**Post-sequencing quality control (QC3) of Hi-C libraries**

For post-sequencing library QC, one million trimmed read pairs for each Hi-C library were sampled using the ‘subseq’ function of the program seqtk ver. 1.2-r94 (https://github.com/lh3/seqtk). The resultant sets of read pairs were processed using HiC-Pro ver. 2.11.1 [25] with bowtie2 ver. 2.3.4.1 [37] to evaluate the insert structure and mapping status onto the softshell turtle genome assembly PelSin_1.0

(GCF_000230535.1) or the human genome assembly hg19. This resulted in categorization as valid interaction pairs and invalid pairs, with the latter being divided further into 'dangling end', 'religation', 'self circle', and 'single-end' pairs (Fig. 4). To process the read pairs derived from the libraries prepared using either HindIII or DpnII (Sau3AI) with the iconHi-C protocol (Library a–d) and the Phase kit (Library g and h), the restriction fragment file required by HiC-Pro was prepared according to the script 'digest_genome.py' of HiC-Pro. To process the reads derived from the Arima kit (Library e and f), all restriction sites ('GATC' and 'GANTC') were inserted into the script. In addition, the nucleotide sequences of all possible ligated sites generated by restriction enzymes were included in a configuration file of HiC-Pro. The details of this procedure and the sample code used are included in Supplementary Protocol S2.

**Computation for Hi-C scaffolding**

To control our comparison with intended input data sizes, a certain number of trimmed read pairs were sampled for each library with seqtk, as described above. Scaffolding was processed with the following methods employing two program pipelines, 3d-dna and SALSA2.

Scaffolding via 3d-dna was performed using Hi-C read mapping onto the genome with Juicer ver. 20180805 [38] using the default parameters with BWA ver.0.7.17-r1188 [39]. The restriction fragment file required by Juicer was prepared by the script 'generate_site_positions.py' script of Juicer. By converting the restriction fragment file of HiC-Pro to the Juicer format, an original script that was compatible with multiple restriction enzymes was prepared (Supplementary Protocol S2). Scaffolding via 3d-dna ver. 20180929 was performed using variable parameters (see

Fig. 9A).

Scaffolding via SALSA2 using Hi-C reads was preceded by Hi-C read pair processing with the Arima mapping pipeline ver. 20181207 (https://github.com/ArimaGenomics/mapping_pipeline) together with BWA, SAMtools ver. 1.8-21-gf6f50ac [40], and Picard ver. 2.18.12 (https://github.com/broadinstitute/picard). The mapping result in the binary alignment map (bam) format was converted into a BED file by bamToBed of Bedtools ver. 2.26.0 [41], the output of which was used as the input of scaffolding using SALSA2 ver. 20181212 with the default parameters.

**Completeness assessment of Hi-C scaffolds**

gVolante ver. 1.2.1 [32] was used to perform an assessment of the sequence length distribution and gene space completeness based on the coverage of one-to-one reference orthologues with BUSCO v2/v3 employing the one-to-one orthologue set 'Tetrapoda' supplied with BUSCO [42]. No cut-off length was used in this assessment.

**Continuity assessment using RNA-seq read mapping**

Paired-end reads obtained by RNA-seq of softshell turtle embryos at multiple stages were downloaded from NCBI SRA (DRX001576) and were assembled using Trinity ver. 2.7.0 [43] with default parameters. The assembled transcript sequences were mapped to the Hi-C scaffold sequences with pblat [44], and the output was assessed with isoblat ver. 0.31 [45].

**Comparison with chromosome FISH results**

Cytogenetic validation of Hi-C scaffolding results was performed by comparing the gene locations on the scaffold sequences with those provided by previous chromosome FISH for 162 protein-coding genes [18-22]. The nucleotide exonic sequences for those 162 genes were retrieved from GenBank and aligned with Hi-C scaffold sequences using BLAT ver. 36x2 [46], followed by the analysis of their positions and orientation along the Hi-C scaffold sequences.

## Availability of supporting data

All sequence data generated in this study have been submitted to the DDBJ Sequence Read Archive (DRA) under accession IDs DRA008313 and DRA008947. The datasets supporting the results of this article are available in FigShare (https://figshare.com/s/6ea495a65fc231a74458).

## Additional files

Supplementary Figure S1. DNA size distribution of the softshell turtle Hi-C libraries.

Supplementary Figure S2. Pre-sequencing quality control of softshell turtle blood Hi-C libraries (Library a and b).

Supplementary Figure S3. Pre-sequencing quality control (QC2) of the Hi-C libraries generated using the Phase kit (Library g and h).

Supplementary Figure S4. Structural analysis of the possibly chimeric scaffold in Assembly 8.

Supplementary Figure S5. Hi-C contact maps for selected softshell turtle Hi-C scaffolds.

Supplementary Figure S6. Pairwise alignment of Hi-C scaffolds.

Supplementary Table S1. Statistics of the Chinese softshell turtle draft genome assembly before Hi-C.

Supplementary Table S2. HiC-Pro results for the human GM12878 HindIII Hi-C library with reduced reads.

Supplementary Table S3. Quality control of the human GM12878 Hi-C libraries.

Supplementary Table S4. Effect of the duration of restriction enzyme digestion and ligation.

Supplementary Table S5. Quality control of Hi-C libraries.

Supplementary Table S6. Scaffolding results with variable input data and computational parameters.

Supplementary Table S7. Mapping results of assembled transcript sequences onto Hi-C scaffolds.

Supplementary Table S8. Effect of variable degrees of PCR amplification.

Supplementary Table S9. HiC-Pro results for the softshell turtle liver libraries (Library d, e, and h) with reduced reads.

Supplementary Protocol S1. iconHi-C protocol.

Supplementary Protocol S2. Computational protocol to support the use of multiple enzymes.

**Abbreviations**

PCR: polymerase chain reaction; FISH, fluorescence *in situ* hybridization; BUSCO, benchmarking universal single-copy orthologs; NCBI, National Center for Biotechnology Information; NGS, next generation DNA sequencing.

**Funding**

This work was supported by intramural grants within RIKEN to S.K. and I.H. and by a Grant-in-Aid for Scientific Research on Innovative Areas from the Ministry of Education, Culture, Sports, Science, and Technology (MEXT) to I.H. (18H05530).

**Competing interests**

The authors declare that they have no competing interests.


## Acknowledgements

The authors acknowledge Naoki Irie, Juan Pascual Anaya and Tatsuya Hirasawa in Laboratory for Evolutionary Morphology, RIKEN BDR for suggestions for sampling, Rawin Poonperm for comments and discussion on the iconHi-C protocol, Olga Dudchenko, Erez Lieberman-Aiden, Arang Rhie, Sergey Koren, and Jay Ghurye for their technical suggestions for sample preparation and computation, Yoshinobu Uno for guidance in the cytogenetic data interpretation, and Anthony Schmitt of Arima Genomics and Stephen Eacker of Phase Genomics for providing information about the Hi-C kits. The authors also thank the other members of the Laboratory for Phyloinformatics and Laboratory for Developmental Epigenetics in RIKEN BDR for technical support and discussion.


## Author contributions

S.K., I.H., H.M., and M.K. conceived the study. M.K. and K.T. performed laboratory works, and O.N. performed bioinformatic analysis. M.K., O.N., and H.M. analyzed the data. S.K., M.K., and O.N. drafted the manuscript. All authors contributed to the finalization of the manuscript.

## References


1. Rowley MJ and Corces VG. Organizational principles of 3D genome architecture. Nat Rev Genet. 2018;19 12:789-800. doi:10.1038/s41576-018-0060-8.

2. Lieberman-Aiden E, van Berkum NL, Williams L, Imakaev M, Ragoczy T, Telling A, et al. Comprehensive mapping of long-range interactions reveals folding principles of the human genome. Science. 2009;326 5950:289-93. doi:10.1126/science.1181369.
3. Rao SS, Huntley MH, Durand NC, Stamenova EK, Bochkov ID, Robinson JT, et al. A 3D map of the human genome at kilobase resolution reveals principles of chromatin looping. Cell. 2014;159 7:1665-80. doi:10.1016/j.cell.2014.11.021.
4. Burton JN, Adey A, Patwardhan RP, Qiu R, Kitzman JO and Shendure J. Chromosome-scale scaffolding of de novo genome assemblies based on chromatin interactions. Nat Biotechnol. 2013;31 12:1119-25. doi:10.1038/nbt.2727.
5. Marie-Nelly H, Marbouty M, Cournac A, Flot JF, Liti G, Parodi DP, et al. High-quality genome (re)assembly using chromosomal contact data. Nat Commun. 2014;5 1:5695. doi:10.1038/ncomms6695.
6. Kaplan N and Dekker J. High-throughput genome scaffolding from in vivo DNA interaction frequency. Nat Biotechnol. 2013;31 12:1143-7. doi:10.1038/nbt.2768.
7. Sedlazeck FJ, Lee H, Darby CA and Schatz MC. Piercing the dark matter: bioinformatics of long-range sequencing and mapping. Nat Rev Genet. 2018;19 6:329-46. doi:10.1038/s41576-018-0003-4.

8. Putnam NH, O'Connell BL, Stites JC, Rice BJ, Blanchette M, Calef R, et al. Chromosome-scale shotgun assembly using an in vitro method for long-range linkage. Genome Res. 2016;26 3:342-50. doi:10.1101/gr.193474.115.

9. Ghurye J, Pop M, Koren S, Bickhart D and Chin CS. Scaffolding of long read assemblies using long range contact information. BMC Genomics. 2017;18 1:527. doi:10.1186/s12864-017-3879-z.

10. Ghurye J, Rhie A, Walenz BP, Schmitt A, Selvaraj S, Pop M, et al. Integrating Hi-C links with assembly graphs for chromosome-scale assembly. PLoS Comput Biol. 2019;15 8:e1007273. doi:10.1371/journal.pcbi.1007273.

11. Dudchenko O, Batra SS, Omer AD, Nyquist SK, Hoeger M, Durand NC, et al. De novo assembly of the Aedes aegypti genome using Hi-C yields chromosome-length scaffolds. Science. 2017;356 6333:92-5. doi:10.1126/science.aal3327.

12. Ghurye J and Pop M. Modern technologies and algorithms for scaffolding assembled genomes. PLoS Comput Biol. 2019;15 6:e1006994. doi:10.1371/journal.pcbi.1006994.

13. Lewin HA, Robinson GE, Kress WJ, Baker WJ, Coddington J, Crandall KA, et al. Earth BioGenome Project: Sequencing life for the future of life. Proc Natl Acad Sci USA. 2018;115 17:4325-33. doi:10.1073/pnas.1720115115.

14. Koepfli KP, Paten B, Genome KCoS and O'Brien SJ. The Genome 10K Project: a way forward. Annu Rev Anim Biosci. 2015;3:57-111. doi:10.1146/annurev-animal-090414-014900.

15. Dudchenko O, Shamim MS, Batra SS, Durand NC, Musial NT, Mostofa R, et al. The Juicebox Assembly Tools module facilitates de novo assembly of mammalian genomes with chromosome-length scaffolds for under $1000. bioRxiv. 2018:254797. doi:10.1101/254797.

16. Belaghzal H, Dekker J and Gibcus JH. Hi-C 2.0: An optimized Hi-C procedure for high-resolution genome-wide mapping of chromosome conformation. Methods. 2017;123:56-65. doi:10.1016/j.ymeth.2017.04.004.

17. Kuratani S, Kuraku S and Nagashima H. Evolutionary developmental perspective for the origin of turtles: the folding theory for the shell based on the developmental nature of the carapacial ridge. Evol Dev. 2011;13 1:1-14. doi:10.1111/j.1525-142X.2010.00451.x.

18. Matsuda Y, Nishida-Umehara C, Tarui H, Kuroiwa A, Yamada K, Isobe T, et al. Highly conserved linkage homology between birds and turtles: bird and turtle chromosomes are precise counterparts of each other. Chromosome Res. 2005;13 6:601-15. doi:10.1007/s10577-005-0986-5.

19. Kuraku S, Ishijima J, Nishida-Umehara C, Agata K, Kuratani S and Matsuda Y. cDNA-based gene mapping and GC3 profiling in the soft-shelled turtle suggest a chromosomal size-dependent GC bias shared by sauropsids. Chromosome Res. 2006;14 2:187-202. doi:10.1007/s10577-006-1035-8.

20. Uno Y, Nishida C, Tarui H, Ishishita S, Takagi C, Nishimura O, et al. Inference of the protokaryotypes of amniotes and tetrapods and the evolutionary processes of microchromosomes from comparative gene mapping. PLoS One. 2012;7 12:e53027. doi:10.1371/journal.pone.0053027.

21. Kawai A, Nishida-Umehara C, Ishijima J, Tsuda Y, Ota H and Matsuda Y. Different origins of bird and reptile sex chromosomes inferred from comparative mapping of chicken Z-linked genes. Cytogenet Genome Res. 2007;117 1-4:92-102. doi:10.1159/000103169.

22. Kawagoshi T, Uno Y, Matsubara K, Matsuda Y and Nishida C. The ZW micro-sex chromosomes of the Chinese soft-shelled turtle (Pelodiscus sinensis, Trionychidae, Testudines) have the same origin as chicken chromosome 15. Cytogenet Genome Res. 2009;125 2:125-31. doi:10.1159/000227837.

23. Wang Z, Pascual-Anaya J, Zadissa A, Li W, Niimura Y, Huang Z, et al. The draft genomes of soft-shell turtle and green sea turtle yield insights into the development

and evolution of the turtle-specific body plan. Nat Genet. 2013;45 6:701-6. doi:10.1038/ng.2615.

24. Belton JM, McCord RP, Gibcus JH, Naumova N, Zhan Y and Dekker J. Hi-C: a comprehensive technique to capture the conformation of genomes. Methods. 2012;58 3:268-76. doi:10.1016/j.ymeth.2012.05.001.

25. Servant N, Varoquaux N, Lajoie BR, Viara E, Chen CJ, Vert JP, et al. HiC-Pro: an optimized and flexible pipeline for Hi-C data processing. Genome Biol. 2015;16:259. doi:10.1186/s13059-015-0831-x.

26. Sofueva S, Yaffe E, Chan WC, Georgopoulou D, Vietri Rudan M, Mira-Bontenbal H, et al. Cohesin-mediated interactions organize chromosomal domain architecture. Embo j. 2013;32 24:3119-29. doi:10.1038/emboj.2013.237.

27. Mayjonade B, Gouzy J, Donnadieu C, Pouilly N, Marande W, Callot C, et al. Extraction of high-molecular-weight genomic DNA for long-read sequencing of single molecules. Biotechniques. 2016;61 4:203-5. doi:10.2144/000114460.

28. Tanegashima C, Nishimura O, Motone F, Tatsumi K, Kadota M and Kuraku S. Embryonic transcriptome sequencing of the ocellate spot skate Okamejei kenojei. Sci Data. 2018;5:180200. doi:10.1038/sdata.2018.200.

29. DeMaere MZ and Darling AE. bin3C: exploiting Hi-C sequencing data to

accurately resolve metagenome-assembled genomes. Genome Biol. 2019;20 1:46. doi:10.1186/s13059-019-1643-1.

30. Botero-Castro F, Figuet E, Tilak MK, Nabholz B and Galtier N. Avian Genomes Revisited: Hidden Genes Uncovered and the Rates versus Traits Paradox in Birds. Mol Biol Evol. 2017;34 12:3123-31. doi:10.1093/molbev/msx236.

31. Hotaling S and Kelley JL. The rising tide of high-quality genomic resources. Mol Ecol Resour. 2019;19 3:567-9. doi:10.1111/1755-0998.12964.

32. Nishimura O, Hara Y and Kuraku S. gVolante for standardizing completeness assessment of genome and transcriptome assemblies. Bioinformatics. 2017;33 22:3635-7. doi:10.1093/bioinformatics/btx445.

33. Roach MJ, Schmidt SA and Borneman AR. Purge Haplotigs: allelic contig reassignment for third-gen diploid genome assemblies. BMC Bioinformatics. 2018;19 1:460. doi:10.1186/s12859-018-2485-7.

34. Kadota M, Hara Y, Tanaka K, Takagi W, Tanegashima C, Nishimura O, et al. CTCF binding landscape in jawless fish with reference to Hox cluster evolution. Sci Rep. 2017;7 1:4957. doi:10.1038/s41598-017-04506-x.

35. Ikeda T, Hikichi T, Miura H, Shibata H, Mitsunaga K, Yamada Y, et al. Srf destabilizes cellular identity by suppressing cell-type-specific gene expression

programs. Nat Commun. 2018;9 1:1387. doi:10.1038/s41467-018-03748-1.

36. Adey A, Morrison HG, Asan, Xun X, Kitzman JO, Turner EH, et al. Rapid, low-input, low-bias construction of shotgun fragment libraries by high-density in vitro transposition. Genome Biol. 2010;11 12:R119. doi:10.1186/gb-2010-11-12-r119.
37. Langmead B and Salzberg SL. Fast gapped-read alignment with Bowtie 2. Nat Methods. 2012;9 4:357-9. doi:10.1038/nmeth.1923.
38. Durand NC, Shamim MS, Machol I, Rao SS, Huntley MH, Lander ES, et al. Juicer Provides a One-Click System for Analyzing Loop-Resolution Hi-C Experiments. Cell Syst. 2016;3 1:95-8. doi:10.1016/j.cels.2016.07.002.
39. Li H and Durbin R. Fast and accurate short read alignment with Burrows-Wheeler transform. Bioinformatics. 2009;25 14:1754-60. doi:10.1093/bioinformatics/btp324.
40. Li H. A statistical framework for SNP calling, mutation discovery, association mapping and population genetical parameter estimation from sequencing data. Bioinformatics. 2011;27 21:2987-93. doi:10.1093/bioinformatics/btr509.
41. Quinlan AR and Hall IM. BEDTools: a flexible suite of utilities for comparing genomic features. Bioinformatics. 2010;26 6:841-2. doi:10.1093/bioinformatics/btq033.

42. Simao FA, Waterhouse RM, Ioannidis P, Kriventseva EV and Zdobnov EM. BUSCO: assessing genome assembly and annotation completeness with single-copy orthologs. Bioinformatics. 2015;31 19:3210-2. doi:10.1093/bioinformatics/btv351.

43. Grabherr MG, Haas BJ, Yassour M, Levin JZ, Thompson DA, Amit I, et al. Full-length transcriptome assembly from RNA-Seq data without a reference genome. Nat Biotechnol. 2011;29 7:644-52. doi:10.1038/nbt.1883.

44. Wang M and Kong L. pblat: a multithread blat algorithm speeding up aligning sequences to genomes. BMC Bioinformatics. 2019;20 1:28. doi:10.1186/s12859-019-2597-8.

45. Ryan JF. Baa.pl: A tool to evaluate de novo genome assemblies with RNA transcripts. arXiv e-prints. 2013;arXiv:1309.2087.

46. Kent WJ. BLAT--the BLAST-like alignment tool. Genome Res. 2002;12 4:656-64. doi:10.1101/gr.229202.

47. Imakaev M, Fudenberg G, McCord RP, Naumova N, Goloborodko A, Lajoie BR, et al. Iterative correction of Hi-C data reveals hallmarks of chromosome organization. Nat Methods. 2012;9 10:999-1003. doi:10.1038/nmeth.2148.

**Table 1:** Overview of the specification of major scaffolding programs.

| Program | Support and availability | Input data requirement | Other information | Literature |
|---|---|---|---|---|
| LACHESIS | Developer's support discontinued; intricate installation | Generic bam format | No function to correct scaffold misjoins | [4] |
| HiRise | Open source version at GitHub not updated since 2015 | Generic bam format | Employed in Dovetail Chicago/Hi-C service. Default input sequence length cut-off=1000 bp | [8] |
| 3d-dna | Actively maintained and supported by the developer | Not compatible with multiple enzymes; Accept only Juicer mapper format | Default parameters: -t 15000 (input sequence length cut-off), -r 2 (no. of iterations for misjoin correction) | [11, 38] |
| SALSA2 | Actively maintained and supported by the developer | Compatible with multiple enzymes; generic bam (bed) file, assembly graph, unitig, 10x link files | Default parameters: -c 1000 (input sequence length cut-off), -i 3 (no. of iterations for misjoin correction) | [9, 10] |

# Figures

A

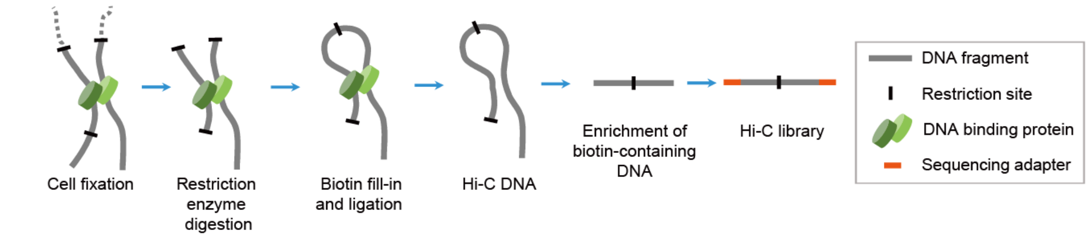


B

| Different specifications | iconHi-C (Our protocol) | Arima-HiC Kit (ver. A160107 v00, with the KAPA Hyper Prep Kit) | Phase Proximo Hi-C Kit (Animal ver. 1.0) | Dovetail Hi-C Kit (ver. 1.4, with Dovetail Library Module and Primer Set) |
|---|---|---|---|---|
| Cell fixation | 10 min (cells) or 15 min (tissue) in 1 % formaldehyde at 25°C; up to $1\times10^7$ cells or up to 1 $cm^3$ tissue | 10 min (cells) or 20 min (tissue) in 2 % formaldehyde at RT; 0.5-1 $\times10^7$ cells or 100-500 mg tissue | 15 min in crosslinking solution (included in the kit) at RT; $1\times10^7$ cells or 100 mg tissue | 20 min in 1.5 % formaldehyde at RT; $0.5\times10^6$ cells and 20-40 mg tissue |
| Sample amount for restriction digestion and ligation | $1\text{-}2\times10^6$ cells or tissue estimated to contain 2-10 µg DNA | Cells or tissue estimated to contain 750 ng - 5 µg DNA | $1\times10^7$ cells or 100 mg tissue | $0.5\times10^6$ cells or 20-40 mg tissue |
| Restriction enzyme digestion | HindIII (cuts at "AAGCTT") or DpnII (cuts at "GATC"), 16 hrs at 37°C | Cocktail of A1 and A2 enzymes (cuts at "GATC" and "GANTC"), 30-60 min at 37°C | Sau3AI (cuts at "GATC"), 1 hr at 37°C | DpnII (cuts at "GATC"), 1 hr at 37°C |
| Ligation | 6 hrs at 16°C | 15 min at RT | 4 hrs at RT | 1-16 hrs at 16°C |
| Reverse crosslinking | 16 hrs at 65°C | 1.5-16 hrs at 68°C | 1-16 hrs at 60°C | 45 min at 68°C |
| Hi-C DNA extraction | Phenol/chloroform extraction | DNA purification beads (e.g. AMPure XP) | Spin column (included in the kit) | SPRIselect beads |
| Hi-C DNA QC | Check for the size shift before and after ligation (QC1) | Check for the yield of biotin-labeled DNA (Arima-QC1) | Check for the DNA yield before proximity ligation | Check for the DNA yield |
| DNA amount for library preparation | 250 ng - 2 µg | 125 ng - 2 µg | N/A | 200 ng |
| Removal of biotin from un-ligated DNA ends | By T4 DNA polymerase | N/A | N/A | N/A |
| DNA fragmentation | Sonication (Covaris) | Sonication (Covaris or Diagenode) | Transposase | Sonication (Covaris or Diagenode) |
| Library preparation | Adapter ligation-based (KAPA LTP Library Prep Kit) | Adapter ligation-based | Transposase-based (included in the kit) | Adapter ligation-based |
| PCR cycles | Pre-determination by qPCR (KAPA Real-time Library Amplification Kit) | Pre-determination by qPCR (KAPA Library Quantification Kit; Arima-QC2) | 15 cycles | 11 cycles |
| Size selection | After DNA fragmentation | After DNA fragmentation | After PCR | After PCR |
| Hi-C library QC | Check for yield and size distribution; check for size shift by NheI or ClaI digestion (QC2) | Check for yield and size distribution | Check for yield and size distribution | Check for yield and size distribution |

**Figure 1**: Hi-C library preparation. (A) Basic procedure. (B) Comparison of Hi-C library preparation methods. Only the major differences between the methods are included here. The versions of the Arima and Phase kits used in this study are presented. The KAPA Hyper Prep Kit (KAPA Biosystems) is assumed to be conjunctly used with Arima Hi-C Kit, among the several specified kits. See Supplementary Protocol S1 for the full version of the iconHi-C protocol which was derived from the protocols published previously [3, 26, 35].

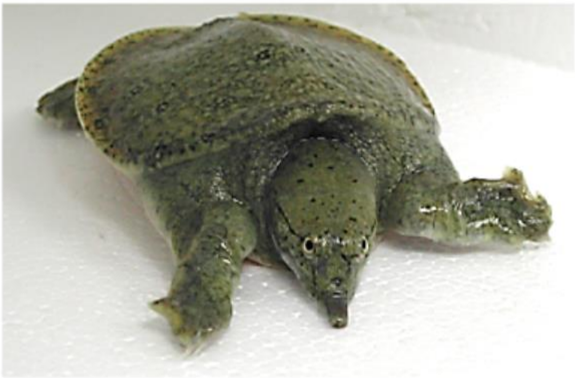

**Figure 2**: A juvenile softshell turtle *Pelodiscus sinensis*.

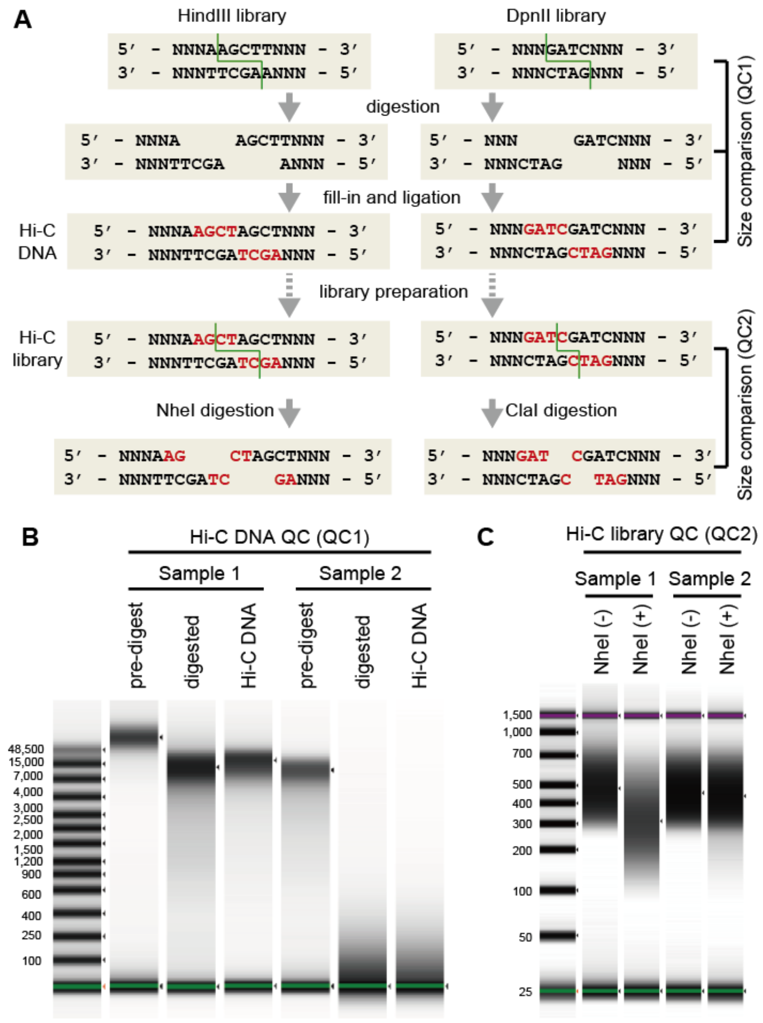


**Figure 3**: Structure of the Hi-C DNA and principle of the quality controls. (A) Schematic representation of the library preparation workflow based on HindIII or DpnII digestion. The patterns of restriction are indicated by the green lines. The nucleotides that are filled in are indicated by the letters in red. (B) Size shift analysis of HindIII-digested Hi-C DNA (QC1). Representative images of qualified (Sample 1) and disqualified (Sample 2) samples are shown. (C) Size shift analysis of the HindIII-digested Hi-C library (QC2). Representative images of the qualified (Sample 1) and disqualified (Sample 2) samples are shown. Size distributions were measured with Agilent 4200 TapeStation.

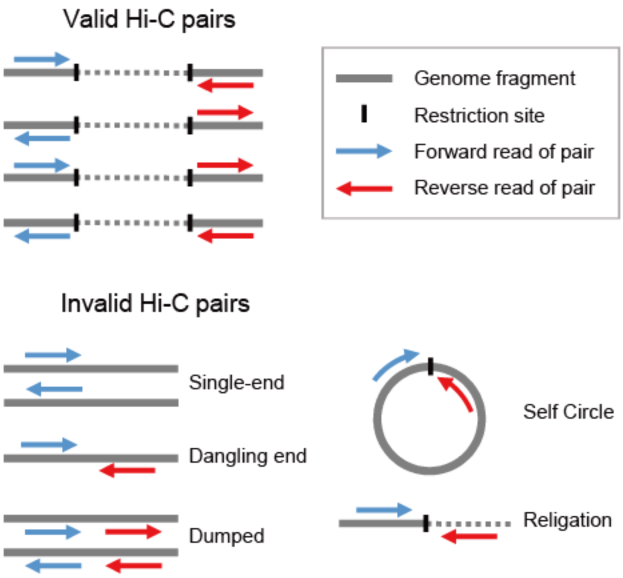


**Figure 4**: Post-sequencing quality control of Hi-C reads. Read pairs were categorized into valid and invalid pairs by HiC-Pro, based on their status in the mapping to the reference genome (see Methods). This figure was adapted from the article that described HiC-Pro originally [25].

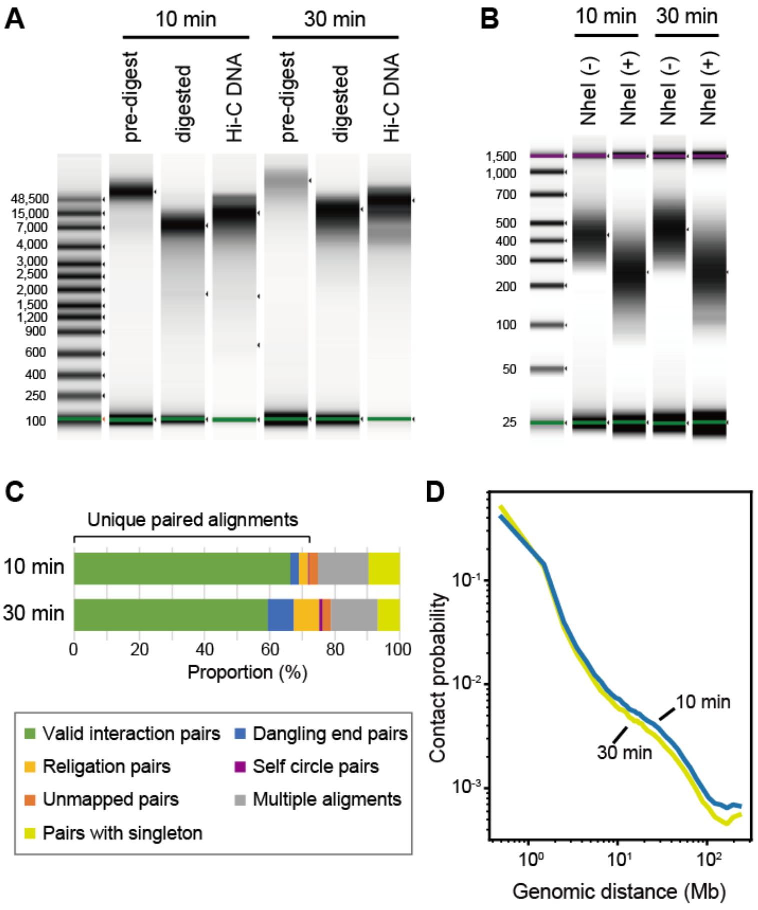


**Figure 5**: Effect of cell fixation duration. (A) QC1 of the HindIII-digested Hi-C DNA of human GM12878 cells fixed for 10 or 30 minutes in 1% formaldehyde. (B) QC2 of the HindIII-digested library of human GM12878 cells. (C) Quality control of the sequence reads by HiC-Pro using 1 M read pairs. See Fig. 4 for the details of the read pair categorization. See Supplementary Table S3 for the actual proportion of the reads in each category. (D) Contact probability measured by the ratio of observed and expected frequencies of Hi-C read pairs mapped along the same chromosome [47].

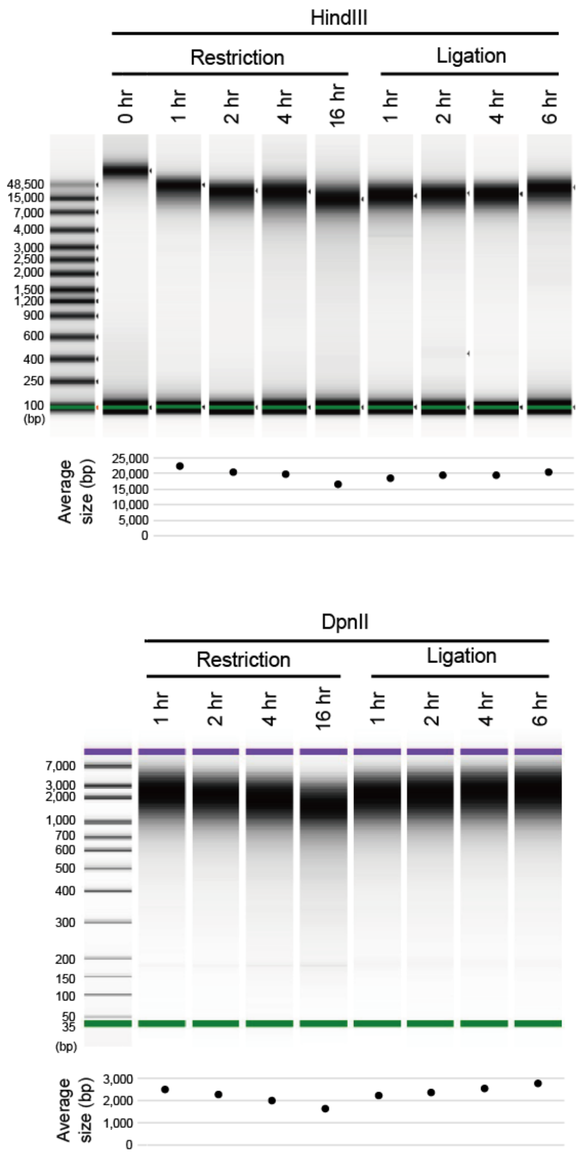


**Figure 6**: Testing varying durations of restriction and ligation. The length distributions of the DNA molecules prepared from human GM12878 cells after restriction and ligation of variable duration are shown. The size distributions of the HindIII-digested samples (top) and DpnII-digested samples (bottom) were measured with an Agilent 4200 TapeStation and an Agilent Bioanalyzer, respectively.

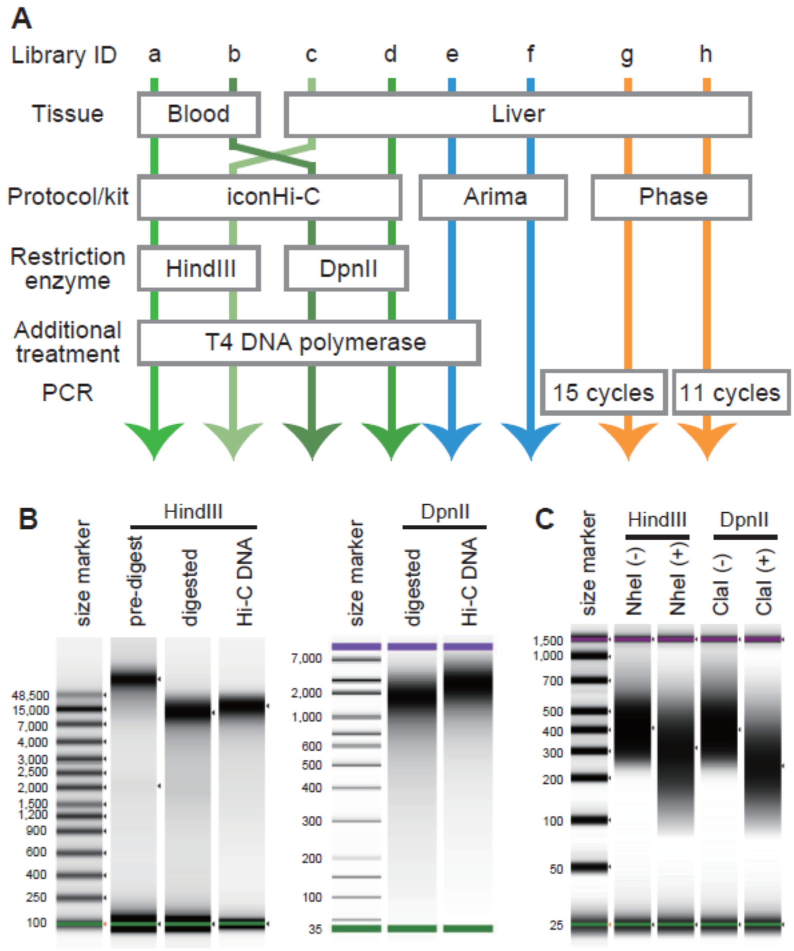


**Figure 7**: Softshell turtle Hi-C libraries prepared for our methodological comparison. (A) Lineup of the prepared libraries. This chart includes only the conditions in preparation methods that varied between these libraries, and the remainder preparation workflows are described in Supplementary Protocol S1 for the non-commercial (‘iconHi-C’) protocol and in the manuals of the commercial kits. (B) Quality control of Hi-C DNA (QC1) for Library c and d. The Hi-C DNA for the Chinese softshell turtle liver sample was prepared with either HindIII or DpnII digestion. (C) Quality control of Hi-C libraries (QC2). The HindIII library prepared from the softshell turtle liver was digested by NheI, and the DpnII library was digested by ClaI (see Fig. 3 for the technical principle). See Supplementary Fig. S2 for the QC1 and QC2 results of the

samples prepared from the blood of this species. See Supplementary Fig. S3 for the QC2 result of the Phase libraries.

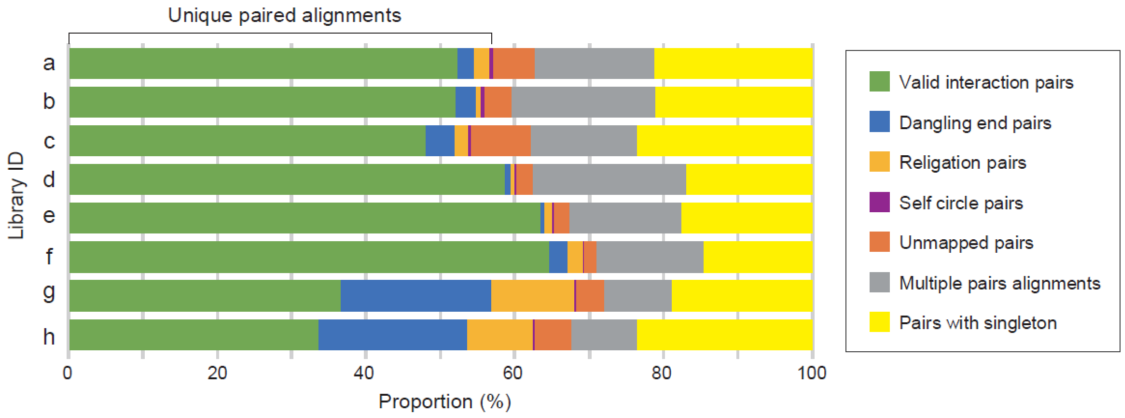


**Figure 8**: Results of the post-sequencing quality control with HiC-Pro. One million read pairs were used for computation with HiC-Pro. See Fig. 7A for the preparation conditions of Library a-h, Fig. 4 for the categorization, and Supplementary Table S5 for the actual proportion of the reads in each category. The post-sequencing quality control using variable read amounts (500 K to 200 M pairs) for one of these softshell turtle libraries (Supplementary Table S9) and human GM12878 libraries (Supplementary Table S2) shows the validity of this quality control with as few as 500 K read pairs.

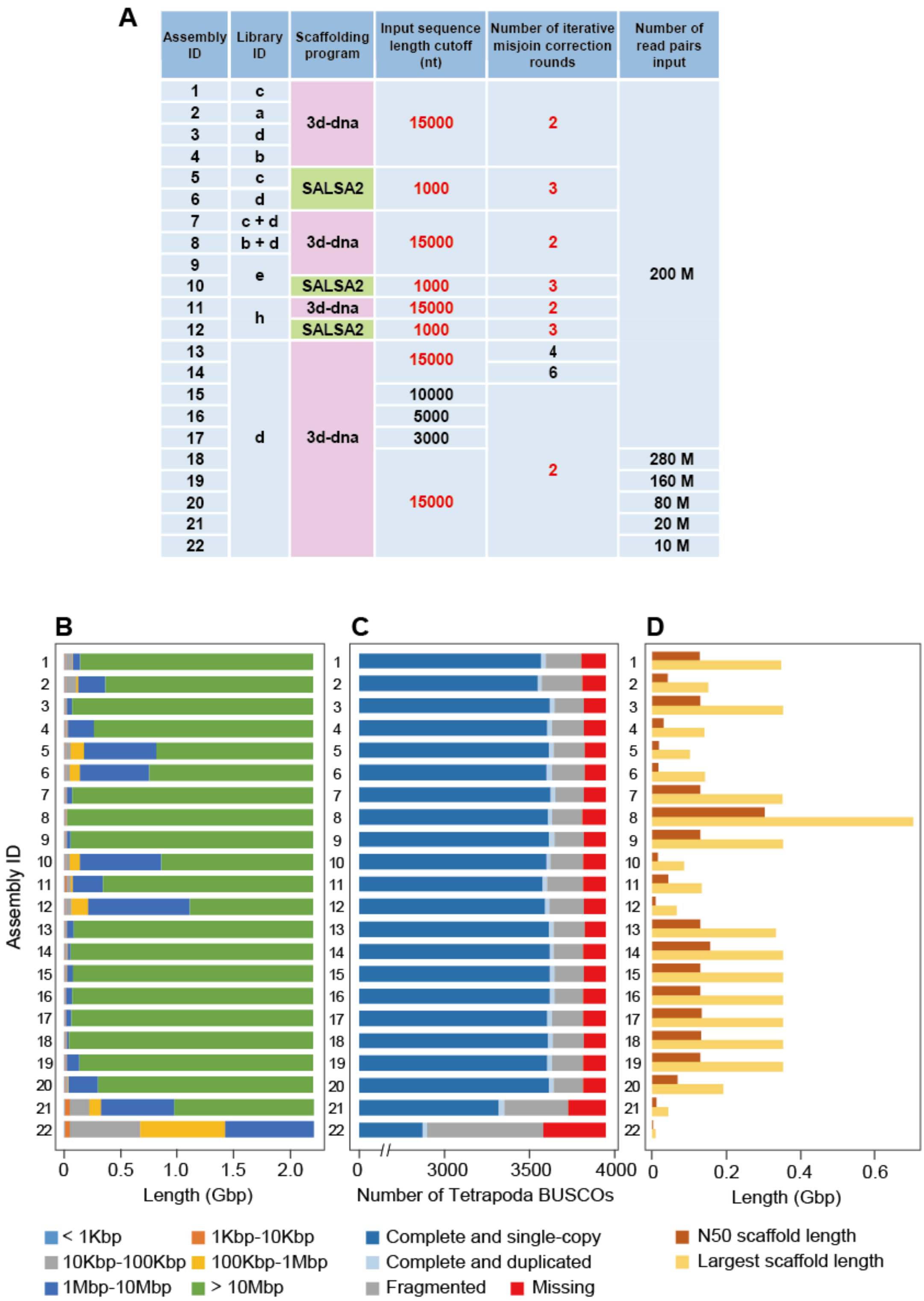

A

| Assembly ID | Library ID | Scaffolding program | Input sequence length cutoff (nt) | Number of iterative misjoin correction rounds | Number of read pairs input |
|---|---|---|---|---|---|
| 1 | c | | | | |
| 2 | a | 3d-dna | 15000 | 2 | |
| 3 | d | | | | |
| 4 | b | | | | |
| 5 | c | SALSA2 | 1000 | 3 | |
| 6 | d | | | | |
| 7 | c + d | | | | |
| 8 | b + d | 3d-dna | 15000 | 2 | |
| 9 | e | | | | 200 M |
| 10 | | SALSA2 | 1000 | 3 | |
| 11 | h | 3d-dna | 15000 | 2 | |
| 12 | | SALSA2 | 1000 | 3 | |
| 13 | | | 15000 | 4 | |
| 14 | | | | 6 | |
| 15 | | | 10000 | | |
| 16 | | | 5000 | | |
| 17 | d | 3d-dna | 3000 | | |
| 18 | | | | 2 | 280 M |
| 19 | | | | | 160 M |
| 20 | | | 15000 | | 80 M |
| 21 | | | | | 20 M |
| 22 | | | | | 10 M |



**Figure 9**: Comparison of Hi-C scaffolding products. (A) Scaffolding conditions used to produce Assembly 1 to 22. The default parameters are shown in red. (B) Scaffold length

distributions. (C) Gene space completeness. (D) Largest and N50 scaffold lengths. See the panel A for Library IDs and Supplementary Table S6 for raw values of the metrics shown in B–D.

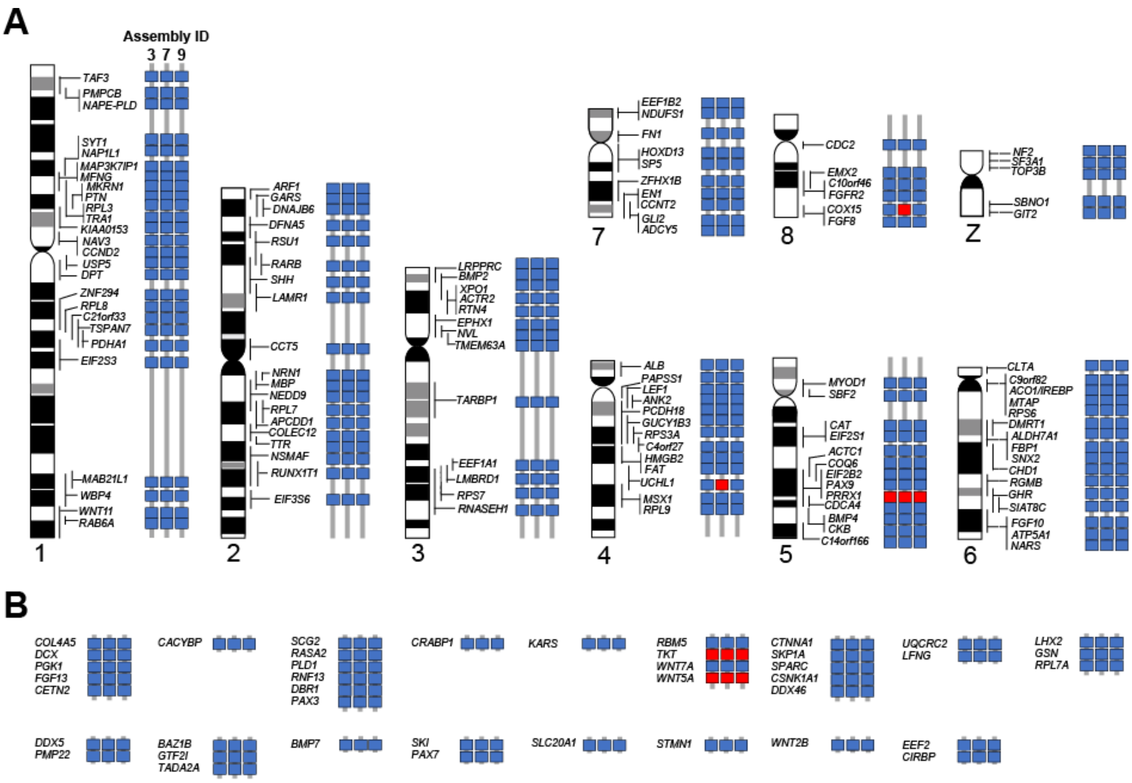


**Figure 10**: Cytogenetic validation of Hi-C scaffolding results. For the scaffolded sequences of Assembly 3, 7, and 9, we evaluated the consistency of the positions of the selected genes that were previously localized on eight macrochromosomes and Z chromosome (A) and microchromosomes (B) by chromosome FISH [18-22] (see Results). Concordant and discordant gene locations on individual assemblies are indicated with blue and red boxes, respectively. The arrays of genes without idiograms in B were identified on chromosomes that are cytogenetically indistinguishable from each other.

distributions. (C) Gene space completeness. (D) Largest and N50 scaffold lengths. See the panel A for Library IDs and Supplementary Table S6 for raw values of the metrics shown in B–D.
