# Supplementary material for: Multifaceted Hi-C benchmarking: what makes a difference in chromosome-scale genome scaffolding?
Source: Gigascience. 2020 Jan 10;9(1):giz158. doi: 10.1093/gigascience/giz158 (PMC6952475; doi:10.1093/gigascience/giz158)
Supplement: giz158_Supplemental_Files [file giz158_supplemental_files.zip › Supplementary_Figs_and_Tables.pdf]

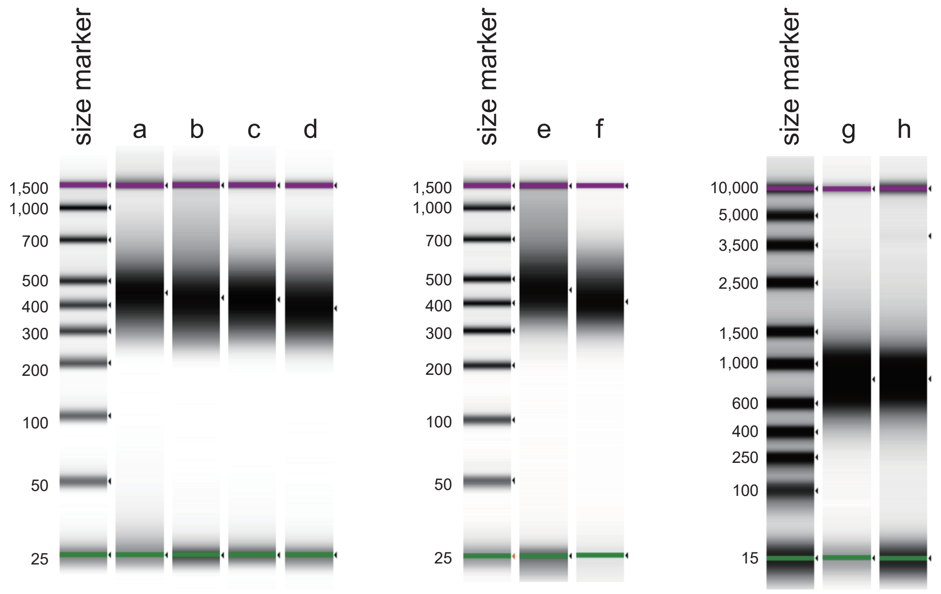

Supplementary Figure S1: DNA size distribution of the softshell turtle Hi-C libraries. The size distribution of the libraries was analysed by an Agilent 4200 TapeStation using the High Sensitivity D1000 kit for Library a-f and the High Sensitivity D5000 kit for Library g and h.

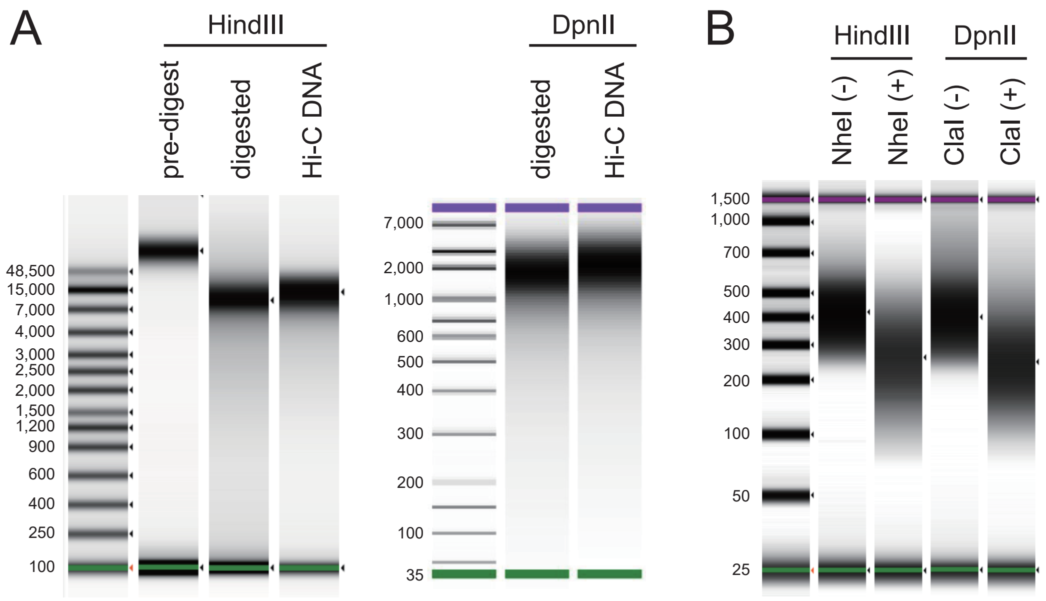

Supplementary Figure S2: Pre-sequencing quality control of softshell turtle blood Hi-C libraries (Library a and b). (A) Quality control of Hi-C DNAs (QC1). Hi-C DNA was prepared from the Chinese softshell turtle blood by HindIII or DpnII digestion (see Fig. 7A for the details). (B) Quality control of Hi-C libraries (QC2). The softshell turtle blood library prepared using HindIII was digested by NheI, and the library prepared using DpnII was digested by ClaI (see Fig. 3 for the technical principle).

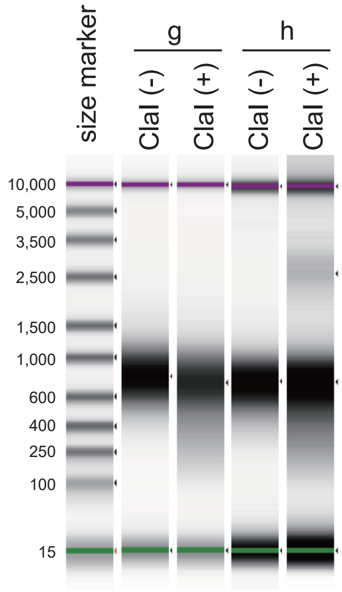

Supplementary Figure S3: Pre-sequencing quality control (QC2) of the Hi-C libraries prepared using the Phase kit (Library g and h). The softshell turtle liver libraries prepared using Sau3A1 were digested by ClaI.

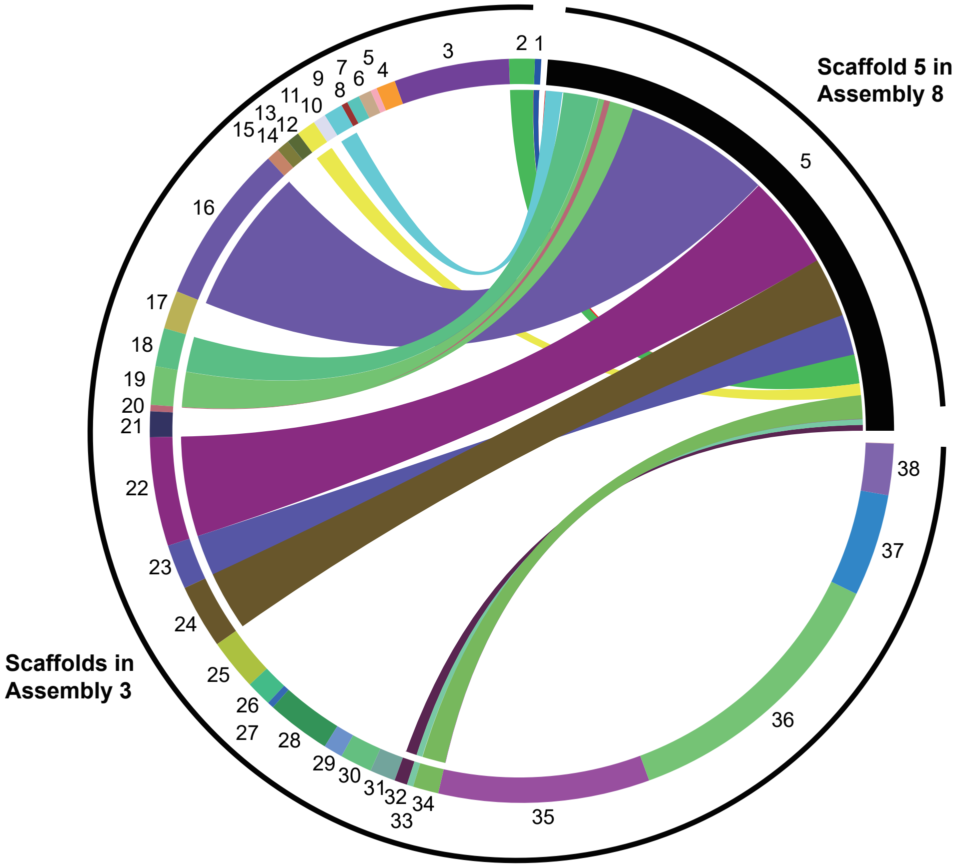

Supplementary Figure S4: Structural analysis of the possibly chimeric scaffold in Assembly 8. This figure shows the nucleotide sequence-level correspondence of the whole sequence of scaffold 5 of Assembly 8 to 14 scaffolds of Assembly 3. Note that the scaffold 5 of Assembly 8 accounts for approximately one-third of the estimated genome size, and that some of the scaffolds of Assembly 3 in the figure have multiple high-similarity regions in scaffold 5 of Assembly 8.

Assembly 3
(iconHi-C)

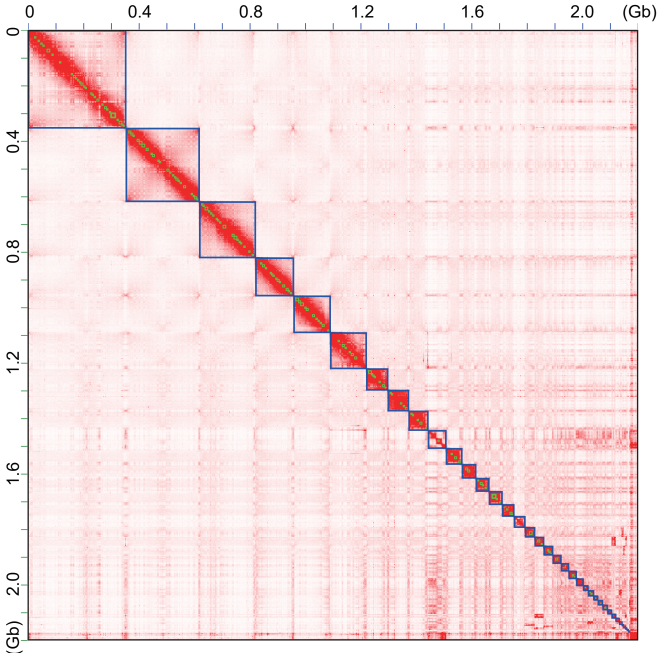

Assembly 9
(Arima)

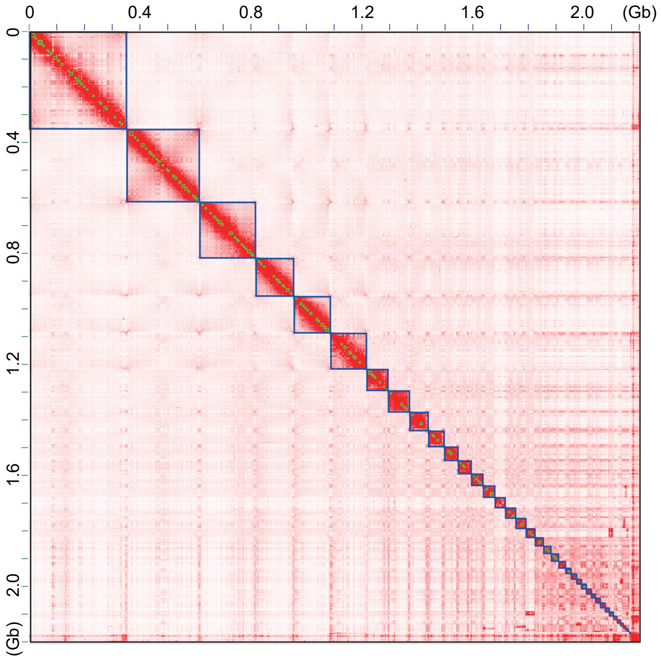

Assembly 11
(Phase)

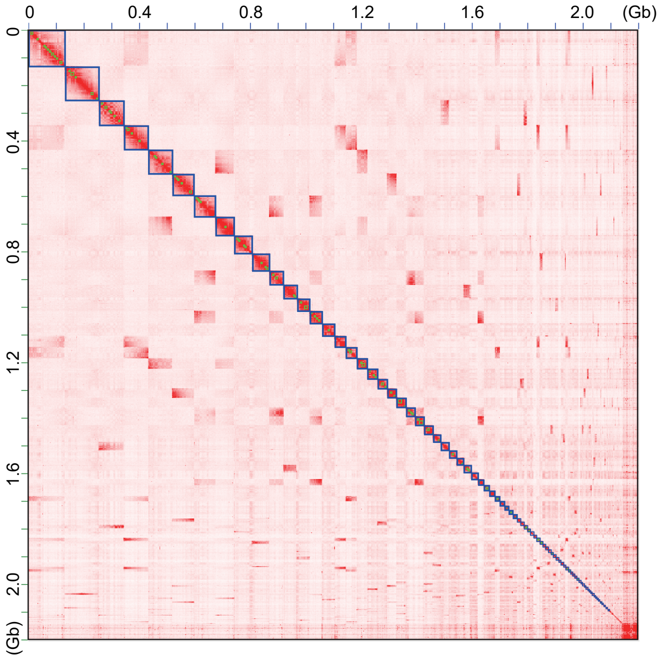

Supplementary Figure S5: Contact maps for selected softshell turtle Hi-C scaffolds. The blue squares are chromosomal units defined by 3d-dna, and the order of the scaffolds is sorted by their length. Assembly 11 exhibits the largest number of intensified blocks diverted from the diagonal line.

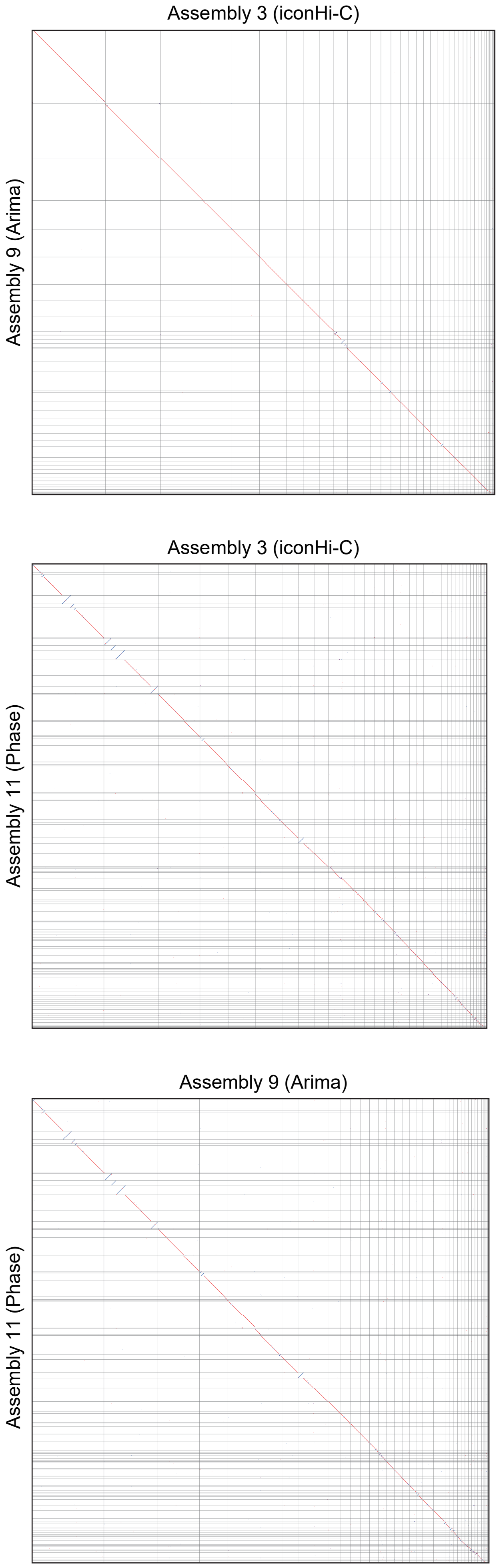

Supplementary Figure S6: Pairwise alignment of Hi-C scaffolds. Genome-wide alignments between the Hi-C scaffolds obtained were performed by LAST, and the dot plots were constructed using the last-dotplot script. Only scaffolds that were 1Mb or longer were included, and the order of the scaffolds along the X-axis was sorted by their length.

Supplementary Table S1: Statistics of the Chinese softshell turtle draft genome assembly before Hi-C.

**Sequence length statistics**	
Number of scaffolds	19,903
Total length of scaffolds	2.20 Gb
Maximum scaffold length	16.02 Mb
Minimum scaffold length	200 b
Number of sequences longer than 1K nt	5,962
Number of sequences longer than 10K nt	3,215
Number of sequences longer than 100K nt	1,491
Number of sequences longer than 1M nt	558
Number of sequences longer than 10M nt	14
Mean scaffold length	110.7 Kb
N50 scaffold length	3.35 Mb
Overall GC-content	44.41%
Overall N-content	4.35%
Sum length proportion of sequences longer than 1M nt	81.00%
Sum length proportion of sequences longer than 10M nt	7.40%

**Completeness of gene space inferred by BUSCO**	
Total number of reference orthologs queried (Tetrapoda)	3,950
Number of reference orthologs detected	
Complete	3,624
Complete + fragmented	3,819
Number of missing orthologs	131
Average number of copies per ortholog	1.01
Detected orthologs that have more than one copy	0.72%

Supplementary Table S2: HiC-Pro results for the human GM12878 HindIII Hi-C library with reduced reads

A. Read alignment category

	Proportion of reads						
Number of input read pairs	500 K	1 M	5 M	10 M	50 M	100 M	200 M
Unique paired alignments	71.0%	71.0%	70.9%	71.0%	71.0%	71.0%	71.0%
Unmapped pairs	3.2%	3.2%	3.2%	3.2%	3.2%	3.2%	3.2%
Low quality pairs	0.0%	0.0%	0.0%	0.0%	0.0%	0.0%	0.0%
Multiple pairs alignments	15.3%	15.3%	15.3%	15.3%	15.3%	15.3%	15.3%
Pairs with singleton	10.5%	10.5%	10.5%	10.5%	10.5%	10.5%	10.5%
Low quality singleton	0.0%	0.0%	0.0%	0.0%	0.0%	0.0%	0.0%
Unique singleton alignments	0.0%	0.0%	0.0%	0.0%	0.0%	0.0%	0.0%
Multiple singleton alignments	0.0%	0.0%	0.0%	0.0%	0.0%	0.0%	0.0%
Reported pairs	71.0%	71.0%	70.9%	71.0%	71.0%	71.0%	71.0%

B. Read pair category

	Proportion of read pairs						
Number of input read pairs	500 K	1 M	5 M	10 M	50 M	100 M	200 M
Valid interaction pairs	65.1%	65.1%	65.1%	65.1%	65.1%	65.1%	65.1%
Valid interaction pairs (forward-forward)	16.2%	16.2%	16.2%	16.2%	16.2%	16.2%	16.2%
Valid interaction pairs (reverse-reverse)	16.1%	16.2%	16.2%	16.2%	16.2%	16.2%	16.2%
Valid interaction pairs (reverse-forward)	15.8%	15.8%	15.7%	15.7%	15.7%	15.7%	15.7%
Valid interaction pairs (forward-reverse)	17.0%	17.0%	16.9%	16.9%	16.9%	16.9%	16.9%
Dangling end pairs	2.8%	2.9%	2.9%	2.9%	2.9%	2.9%	2.9%
Religation pairs	2.6%	2.5%	2.6%	2.6%	2.6%	2.6%	2.6%
Self circle pairs	0.5%	0.4%	0.4%	0.4%	0.4%	0.4%	0.4%
Single-end pairs	0.0%	0.0%	0.0%	0.0%	0.0%	0.0%	0.0%
Filtered pairs	0.0%	0.0%	0.0%	0.0%	0.0%	0.0%	0.0%
Dumped pairs	0.0%	0.0%	0.0%	0.0%	0.0%	0.0%	0.0%

C. Duplicates and contact ranges

	Proportion of read pairs						
Number of input read pairs	500 K	1 M	5 M	10 M	50 M	100 M	200 M
Valid interaction	65.1%	65.1%	65.1%	65.1%	65.1%	65.1%	65.1%
Valid interaction (remove duplicates)	65.1%	65.0%	64.8%	64.5%	62.3%	59.8%	55.2%
Trans interaction	12.0%	12.0%	12.0%	11.9%	11.5%	11.1%	10.2%
Cis interaction	53.1%	53.1%	52.8%	52.6%	50.8%	48.7%	45.0%
Cis short-range interaction (<20Kb)	7.2%	7.2%	7.1%	7.1%	6.8%	6.5%	6.0%
Cis long-range interaction (>20Kb)	45.9%	45.9%	45.7%	45.5%	44.0%	42.2%	38.9%

Note that the *cis* /*trans* judgement based on fragmentary sequences might be unreliable because insufficient continuity increases the trans fraction and instead decreases the cis fraction.

Supplementary Table S3: Quality control of the human GM12878 Hi-C libraries

Library preparation condition

Cell fixation duration (min)	10	30
Amount of tissue for Hi-C reaction (cell number, ug DNA, or mg tissue)	1.5 x 10^6 cells	
Restriction enzyme	HindIII	
Amount of Hi-C DNA used for library preparation (ug)	2	
PCR cycles	6	
Library size (bp)	450	478
Library yield (ng)	140.4	112.2

**Read pair category**	Proportion of read pairs	
Unique paired alignments	72.2%	76.0%
Unmapped pairs	2.6%	2.8%
Low quality pairs	0.0%	0.0%
Multiple pairs alignments	15.6%	14.4%
Pairs with singleton	9.6%	6.8%
Low quality singleton	0.0%	0.0%
Unique singleton alignments	0.0%	0.0%
Multiple singleton alignments	0.0%	0.0%
Reported pairs	72.2%	76.0%

**Hi-C read pair category**	Proportion of read pairs	
Valid interaction pairs	66.4%	59.4%
Dangling end pairs	2.5%	7.9%
Religation pairs	2.7%	7.8%
Self circle pairs	0.5%	0.9%
Single-end pairs	0.0%	0.0%
Filtered pairs	0.0%	0.0%
Dumped pairs	0.0%	0.0%

Supplementary Table S4: Effect of the duration of restriction enzyme digestion and ligation

A. Library preparation condition

Sample	Human GM12878					
Restriction enzyme	DpnII					
Digestion time	16 hrs			1 hr		
Ligation time	6 hrs	1 hr	15 min	6 hrs	1 hr	15 min
PCR cycle	5	6	8	5	5	7
DNA sequencing	Illumina MiSeq, paired-end 300nt					
Number of input read pairs	1,000,000					

B. Read alignment category

Unique paired alignments	73.3%	75.2%	73.4%	74.2%	77.0%	77.5%
Unmapped pairs	4.5%	3.3%	5.2%	4.9%	3.1%	3.1%
Low quality pairs	0.0%	0.0%	0.0%	0.0%	0.0%	0.0%
Multiple pairs alignments	14.4%	14.8%	14.1%	13.9%	14.1%	13.8%
Pairs with singleton	7.8%	6.8%	7.3%	7.1%	5.8%	5.6%
Low quality singleton	0.0%	0.0%	0.0%	0.0%	0.0%	0.0%
Unique singleton alignments	0.0%	0.0%	0.0%	0.0%	0.0%	0.0%
Multiple singleton alignments	0.0%	0.0%	0.0%	0.0%	0.0%	0.0%
Reported pairs	73.3%	75.2%	73.4%	74.2%	77.0%	77.5%

C. Read pair category

Valid interaction pairs	69.0%	71.0%	68.8%	67.4%	70.4%	71.1%
Dangling end pairs	3.6%	3.5%	3.5%	5.5%	5.4%	5.2%
Religation pairs	0.5%	0.5%	0.6%	1.1%	1.1%	1.1%
Self circle pairs	0.1%	0.2%	0.5%	0.1%	0.1%	0.2%
Single-end pairs	0.0%	0.0%	0.0%	0.0%	0.0%	0.0%
Filtered pairs	0.0%	0.0%	0.0%	0.0%	0.0%	0.0%
Dumped pairs	0.0%	0.0%	0.0%	0.0%	0.0%	0.0%

D. Ligation motif and contact ranges

Valid interaction	69.0%	71.0%	68.8%	67.4%	70.4%	71.1%
Valid interaction (remove duplicates)	69.0%	70.9%	68.7%	67.4%	70.4%	71.0%
Ligation motif present	68.2%	70.2%	68.0%	66.7%	69.6%	70.2%
Trans interaction	12.4%	12.6%	12.3%	9.4%	9.6%	9.6%
Cis interaction (total)	56.5%	58.4%	56.5%	58.0%	60.8%	61.4%
(<10Kb)	8.0%	8.0%	7.7%	11.7%	12.1%	11.8%
(10k-100Kb)	13.3%	13.8%	13.5%	14.1%	14.7%	15.1%
(100K-1Mb)	17.0%	17.7%	17.3%	16.0%	17.0%	17.3%
(1Mb-10Mb)	9.1%	9.5%	9.1%	8.3%	8.7%	8.9%
(>10Mb)	9.1%	9.4%	8.8%	7.8%	8.3%	8.3%

Note that the values of ligation motif present and range data of cis interaction were statistically processed from HiC-Pro results.

Supplementary Table S5: Quality control of Hi-C libraries.

Library preparation condition	Library ID							
	a	b	c	d	e	f	g	h
Tissue type	Blood		Liver					
Amount of cells/tissue or DNA used for the Hi-C reaction	2 x 10^6 cells		Tissue estimated to contain 3 µg DNA (based on the iconHi-C protocol: Additional file Protocol Step 3)		10 mg tissue (estimated to contain 4.23 µg DNA based on the input material determination protocol of the Arima-HiC kit)		50 mg tissue (estimated to contain 110.0 ng DNA based on the DNA QC step performed after restriction digestion)	10 mg tissue (estimated to contain 66.3 ng DNA based on the DNA QC step performed after restriction digestion)
Restriction enzyme	HindIII	DpnII	HindIII	DpnII	Arima cocktail		Sau3AI	
Hi-C DNA amount for lib prep (µg)	2	1	2	1	1	1	N/A	
Number of PCR cycles	6	8	6	5	8	9	15	11
Average library size (bp)	488	493	456	444	558	454	892 (1,052 bp before size selection)	882 (1,100 bp before size selection)
Library yield (ng)	112.2	129.6	140.4	172.2	160.0	1,180.0	232.0 (1,008 ng before size selection)	32.8 (88 ng before size selection)
Additional treatment (divergences from the original protocol)	N/A				Treatment of the Hi-C DNA with T4 DNA polymerase	N/A	Size selection of the Hi-C library with AMPure XP	Size selection of the Hi-C library with AMPure XP; Reduced number of PCR cycles
**Read alignment category**	**Proportion of read pairs (%)**							
Unique paired alignments	57.1	55.9	54.2	60.1	65.2	69.3	68.2	62.7
Unmapped pairs	5.7	3.8	8.0	2.5	2.3	1.8	4.0	5.1
Low quality pairs	0.0	0.0	0.0	0.0	0.0	0.0	0.0	0.0
Multiple pairs alignments	16.1	19.4	14.4	20.6	15.1	14.4	9.1	8.8
Pairs with singleton	21.2	20.9	23.4	16.8	17.4	14.5	18.7	23.4
Low quality singleton	0.0	0.0	0.0	0.0	0.0	0.0	0.0	0.0
Unique singleton alignments	0.0	0.0	0.0	0.0	0.0	0.0	0.0	0.0
Multiple singleton alignments	0.0	0.0	0.0	0.0	0.0	0.0	0.0	0.0
Reported pairs	57.1	55.9	54.2	60.1	65.2	69.3	68.2	62.7
**Read pair category**	**Proportion of read pairs (%)**							
Valid interaction pairs	52.4	52.1	48.1	58.8	63.6	64.7	36.5	33.5
Dangling end pairs	2.2	2.8	3.8	0.7	0.5	2.5	20.4	20.1
Religation pairs	2.1	0.7	1.9	0.5	1.1	2.1	11.1	8.9
Self circle pairs	0.4	0.3	0.3	0.1	0.0	0.0	0.1	0.1
Single-end pairs	0.0	0.0	0.0	0.0	0.0	0.0	0.0	0.0
Filtered pairs	0.0	0.0	0.0	0.0	0.0	0.0	0.0	0.0
Dumped pairs	0.1	0.0	0.0	0.0	0.0	0.0	0.0	0.0

See Fig. 4 for the detail of read pair categorization.

Supplementary Table S6: Scaffolding results with variable input data and computational parameters

Assembly ID	Sample preparation condition				Scaffolding condition				Basic sequence compositions													Gene space completeness assessment by BUSCO referring to 3,950 Tetrapoda BUSCOs								
	Library ID	Preparation method	Tissue(s)	Restriction enzyme(s)	Scaffolding program	Smaller input sequence cutoff length*	Numbers of iterative corrections**	Number of input read pairs	Number of scaffolds	Maximum scaffold length	Number of sequences longer than 1K nt	Number of sequences longer than 10K nt	Number of sequences longer than 100K nt	Number of sequences longer than 1M nt	Number of sequences longer than 10M nt	Mean scaffold length	Median scaffold length	N50 scaffold length	Overall N-content (%)	Sum proportion of sequences longer than 1M nt (%)	Sum proportion of sequences longer than 10M nt (%)	Complete + Fragmented BUSCOs (C + F)	Complete and single-copy BUSCOs (S)	Complete and duplicated BUSCOs (D)	Fragmented BUSCOs (F)	Missing BUSCOs (M)	Complete BUSCOs (C)	Average number of copies per ortholog	Detected orthologs that have more than one copy (%)	Scores in percentages
Assembly before Hi-C									19,903	16,024,077	5,962	3,215	1491	558	14	110,660	498	3,350,749	4.35	81.0	7.4	3,819	3,598	26	195	131	3,624	1.01	0.72	C:91.8%[S:91.1%,D:0.7%],F:4.9%,M:3.3%,n:3950
**1**	c	iconHi-C	liver	HindIII	3d-dna	3d-dna defaults		200,000,000	19,824	347,378,596	5,807	2,283	72	46	34	111,169	496	128,648,452	4.41	96.3	93.5	3,807	3,566	30	211	143	3,596	1.01	0.83	C:91.1%[S:90.3%,D:0.8%],F:5.3%,M:3.6%,n:3950
**2**	a		blood						20,899	151,823,619	6,804	3,203	197	91	47	105,456	529	40,769,607	4.41	94.0	83.3	3,811	3,547	27	237	139	3,574	1.01	0.76	C:90.5%[S:89.8%,D:0.7%],F:6.0%,M:3.5%,n:3950
**3**	d		liver	DpnII					18,013	352,879,942	4,005	740	40	38	31	122,341	439	130,239,026	4.41	98.7	96.9	3,821	3,619	27	175	129	3,646	1.01	0.74	C:92.3%[S:91.6%,D:0.7%],F:4.4%,M:3.3%,n:3950
**4**	b		blood						18,215	140,927,669	4,161	821	117	108	62	120,984	446	31,364,868	4.41	98.5	87.8	3,820	3,604	31	185	130	3,635	1.01	0.85	C:92.0%[S:91.2%,D:0.8%],F:4.7%,M:3.3%,n:3950
**5**	c		liver	HindIII	SALSA2	SALSA2 defaults			18,674	102,051,213	4,762	2,051	577	227	53	117,978	462	18,089,892	4.38	92.0	63.0	3,826	3,615	30	181	124	3,645	1.01	0.82	C:92.3%[S:91.5%,D:0.8%],F:4.6%,M:3.1%,n:3950
**6**	d			DpnII					18,469	142,087,433	4,537	1,802	500	215	56	119,293	455	17,382,281	4.38	93.3	65.9	3,823	3,603	31	189	127	3,634	1.01	0.85	C:92.0%[S:91.2%,D:0.8%],F:4.8%,M:3.2%,n:3950
**7**	c+d		liver	HindIII & DpnII	3d-dna	3d-dna defaults			18,087	351,946,035	4,084	766	50	42	34	121,838	441	130,589,277	4.40	98.6	96.8	3,820	3,624	29	167	130	3,653	1.01	0.79	C:92.4%[S:91.7%,D:0.7%],F:4.2%,M:3.4%,n:3950
**8**	b+d		blood & liver	DpnII					17,937	703,313,584	3,922	690	18	15	15	122,863	436	303,431,936	4.41	98.7	98.7	3,809	3,611	25	173	141	3,636	1.01	0.69	C:92.0%[S:91.4%,D:0.6%],F:4.4%,M:3.6%,n:3950
**9**	e	Arima kit	liver	cocktail of A1 and A2	3d-dna	3d-dna defaults			17,756	352,772,394	3,730	670	47	43	35	124,117	429	130,552,621	4.41	98.8	97.4	3,822	3,616	30	176	128	3,646	1.01	0.82	C:92.3%[S:91.5%,D:0.8%],F:4.5%,M:3.2%,n:3950
**10**	e				SALSA2	SALSA2 defaults			18,374	86,047,802	4,450	1,734	505	232	60	119,913	452	14,588,975	4.39	93.5	60.9	3,817	3,599	27	191	133	3,626	1.01	0.74	C:91.8%[S:91.1%,D:0.7%],F:4.8%,M:3.4%,n:3950
**11**	h	Phase kit	liver	Sau3AI	3d-dna	3d-dna defaults			20,629	132,711,915	6,308	1,751	160	99	47	106,827	520	43,805,214	4.41	96.3	84.3	3,817	3,578	28	211	133	3,606	1.01	0.78	C:91.3%[S:90.6%,D:0.7%],F:5.3%,M:3.4%,n:3950
**12**	h				SALSA2	SALSA2 defaults			18,690	65,503,740	4,900	2,241	745	293	55	117,877	467	9,689,575	4.38	90.2	49.6	3,822	3,591	28	203	128	3,619	1.01	0.77	C:91.6%[S:90.9%,D:0.7%],F:5.1%,M:3.3%,n:3950
**13**	d	iconHi-C	liver	DpnII	3d-dna	-i 15000	-r 4	200,000,000	18,154	334,063,790	4,142	826	52	46	34	121,389	443	130,244,796	4.41	98.6	96.1	3,824	3,617	28	179	126	3,645	1.01	0.77	C:92.3%[S:91.6%,D:0.7%],F:4.5%,M:3.2%,n:3950
**14**	d					-i 15000	-r 6		18,199	352,652,404	4,188	845	35	29	22	121,087	445	156,729,449	4.40	98.5	97.3	3,814	3,619	25	170	136	3,644	1.01	0.69	C:92.2%[S:91.6%,D:0.6%],F:4.3%,M:3.5%,n:3950
**15**	d					-i 10000	-r 2		17,775	353,310,449	3,752	450	51	43	33	123,984	430	129,828,393	4.41	98.8	96.4	3,820	3,621	29	170	130	3,650	1.01	0.79	C:92.4%[S:91.7%,D:0.7%],F:4.3%,M:3.3%,n:3950
**16**	d					-i 5000	-r 2		17,424	353,773,630	3,404	391	54	44	34	126,493	419	129,814,843	4.42	98.9	96.6	3,817	3,621	28	168	133	3,649	1.01	0.77	C:92.4%[S:91.7%,D:0.7%],F:4.3%,M:3.3%,n:3950
**17**	d					-i 3000	-r 2		17,206	352,757,195	3,148	357	49	41	34	128,104	412	132,629,580	4.42	99.0	97.1	3,815	3,605	30	180	135	3,635	1.01	0.83	C:92.1%[S:91.3%,D:0.8%],F:4.6%,M:3.3%,n:3950
**18**	d	iconHi-C	liver	DpnII	3d-dna	3d-dna defaults		280,000,000	17,845	352,666,335	3,830	672	44	38	35	123,496	432	132,509,297	4.41	98.8	98.0	3,822	3,612	27	183	128	3,639	1.01	0.74	C:92.1%[S:91.4%,D:0.7%],F:4.6%,M:3.3%,n:3950
**19**	d							160,000,000	18,129	352,585,997	4,128	800	60	54	33	121,556	442	130,809,328	4.40	98.6	93.7	3,817	3,607	29	181	133	3,636	1.01	0.80	C:92.0%[S:91.3%,D:0.7%],F:4.6%,M:3.4%,n:3950
**20**	d							80,000,000	18,729	192,448,115	4,677	1,101	107	90	41	117,654	463	68,103,746	4.40	98.1	86.4	3,817	3,615	27	175	133	3,642	1.01	0.74	C:92.2%[S:91.5%,D:0.7%],F:4.4%,M:3.4%,n:3950
**21**	d							20,000,000	33,115	42,618,151	17,006	7,765	503	208	65	66,681	1,146	11,576,731	4.60	85.2	56.0	3,730	3,322	30	378	220	3,352	1.01	0.89	C:84.9%[S:84.1%,D:0.8%],F:9.6%,M:5.5%,n:3950
**22**	d							10,000,000	47,023	8,708,159	31,216	21,256	2475	367	0	47,049	7,307	616,243	4.78	35.6	0.0	3,583	2,875	28	680	367	2,903	1.01	0.96	C:73.5%[S:72.8%,D:0.7%],F:17.2%,M:9.3%,n:3950
**23**	d	iconHi-C	liver	DpnII	SALSA2	-c 1000	-i 5	200,000,000	18,309	341,886,627	4,379	1,648	383	141	38	120,340	449	49,308,138	4.39	94.0	76.6	3,826	3,606	31	189	124	3,637	1.01	0.85	C:92.1%[S:91.3%,D:0.8%],F:4.8%,M:3.1%,n:3950
**24**	d					-c 1000	-i 7		18,252	427,675,891	4,322	1,593	340	109	32	120,717	448	69,007,845	4.39	94.2	81.4	3,820	3,593	31	196	130	3,624	1.01	0.86	C:91.8%[S:91.0%,D:0.8%],F:5.0%,M:3.2%,n:3950
**25**	d					-c 15000	-i 3		18,485	102,345,483	4,554	1,819	510	220	56	119,189	456	19,070,756	4.38	93.3	65.2	3,827	3,602	29	196	123	3,631	1.01	0.80	C:91.9%[S:91.2%,D:0.7%],F:5.0%,M:3.1%,n:3950
**26**	d					-c 10000	-i 3		18,470	102,549,702	4,538	1,802	500	220	62	119,287	455	17,136,115	4.38	93.5	66.5	3,819	3,601	28	190	131	3,629	1.01	0.77	C:91.9%[S:91.2%,D:0.7%],F:4.8%,M:3.3%,n:3950
**27**	d					-c 5000	-i 3		18,470	102,345,483	4,538	1,802	500	216	57	119,287	455	17,382,281	4.38	93.4	65.8	3,823	3,606	29	188	127	3,635	1.01	0.80	C:92.0%[S:91.3%,D:0.7%],F:4.8%,M:3.2%,n:3950
**28**	d					-c 3000	-i 3		18,464	102,549,702	4,532	1,796	502	217	56	119,326	455	18,421,693	4.38	93.4	64.0	3,827	3,624	30	173	123	3,654	1.01	0.82	C:92.5%[S:91.7%,D:0.8%],F:4.4%,M:3.1%,n:3950

* Default parameters for 3d-dna and SALSA2 are 15000 and 1000, respectively.

** Default parameters for 3d-dna and SALSA2 are 2 and 3, respectively.

Supplementary Table S7: Mapping results of assembled transcript sequences onto Hi-C scaffolds

Assembly ID*	Library ID**	Preparation method	Scaffolding program	Ratio of transcripts with a BLAT entry	Total % coverage of all positions	Number of transcripts mapped to a single contig/scaffold (ratio)	Average number of contigs/scaffolds per mapped transcript
3	d	iconHi-C	3d-dna	0.9877	0.9528	0.8790	1.1953
6	d	iconHi-C	SALSA2	0.9877	0.9528	0.8722	1.2237
7	c + d	iconHi-C	3d-dna	0.9877	0.9528	0.8784	1.1975
9	e	Arima kit	3d-dna	0.9877	0.9528	0.8786	1.1981
11	h	Phase kit	3d-dna	0.9878	0.9496	0.8700	1.2230

*See Fig. 9A for the detail.

**See Fig. 7A for the detail.

Supplementary Table S8: Effect of variable degrees of PCR amplification

Library preparation condition	Library ID	
	**g**	**h**
Tissue type	Liver	
Restriction enzyme	Sau3AI	
Number of PCR cycles	15	11

Hi-C Pro results		
Number of input read pairs	200,000,000	
Category	Proportion of valid interaction (%)	
Valid interaction after removing duplicates	55.1	70.4

See Figure 7A for the detail of the library preparation procedure. Note that 'trans' and 'cis' interactions mean contacts between scaffolds and those within scaffolds,

Supplementary Table S9: HiC-Pro results for the softshell turtle libraries with reduced reads

**Library ID**	**d**			**e**			**h**		

A. Read alignment category

	Proportion of read pairs								
Number of input read pairs	1 M	10 M	100 M	1 M	10 M	100 M	1 M	10 M	100 M
Unique paired alignments	59.4%	59.3%	59.3%	64.5%	64.5%	64.5%	59.4%	59.4%	59.4%
Unmapped pairs	3.0%	2.9%	2.9%	2.7%	2.7%	2.7%	6.7%	6.7%	6.7%
Low quality pairs	0.0%	0.0%	0.0%	0.0%	0.0%	0.0%	0.0%	0.0%	0.0%
Multiple pairs alignments	20.0%	20.1%	20.1%	14.3%	14.4%	14.4%	7.8%	7.8%	7.8%
Pairs with singleton	17.6%	17.7%	17.6%	18.4%	18.5%	18.5%	26.1%	26.2%	26.2%
Low quality singleton	0.0%	0.0%	0.0%	0.0%	0.0%	0.0%	0.0%	0.0%	0.0%
Unique singleton alignments	0.0%	0.0%	0.0%	0.0%	0.0%	0.0%	0.0%	0.0%	0.0%
Multiple singleton alignments	0.0%	0.0%	0.0%	0.0%	0.0%	0.0%	0.0%	0.0%	0.0%
Reported pairs	59.4%	59.3%	59.3%	64.5%	64.5%	64.5%	59.4%	59.4%	59.4%

B. Read pair category

	Proportion of read pairs								
Number of input read pairs	1 M	10 M	100 M	1 M	10 M	100 M	1 M	10 M	100 M
Valid interaction pairs	58.0%	57.9%	58.0%	62.7%	62.7%	62.7%	28.1%	28.1%	28.1%
Valid interaction pairs (forward-forward)	14.5%	14.4%	14.5%	15.4%	15.4%	15.4%	6.1%	6.1%	6.2%
Valid interaction pairs (reverse-reverse)	14.5%	14.5%	14.5%	15.5%	15.5%	15.4%	6.2%	6.2%	6.2%
Valid interaction pairs (reverse-forward)	14.4%	14.4%	14.4%	15.2%	15.2%	15.2%	6.1%	6.1%	6.1%
Valid interaction pairs (forward-reverse)	14.6%	14.6%	14.6%	16.6%	16.6%	16.6%	9.7%	9.7%	9.6%
Dangling end pairs	0.8%	0.8%	0.8%	0.7%	0.7%	0.7%	22.9%	23.0%	23.0%
Religation pairs	0.5%	0.5%	0.5%	1.1%	1.1%	1.1%	8.2%	8.2%	8.2%
Self circle pairs	0.1%	0.1%	0.1%	0.0%	0.0%	0.0%	0.1%	0.1%	0.1%
Single-end pairs	0.0%	0.0%	0.0%	0.0%	0.0%	0.0%	0.0%	0.0%	0.0%
Filtered pairs	0.0%	0.0%	0.0%	0.0%	0.0%	0.0%	0.0%	0.0%	0.0%
Dumped pairs	0.0%	0.0%	0.0%	0.0%	0.0%	0.0%	0.0%	0.0%	0.0%

C. Duplicates and contact ranges

	Proportion of read pairs								
Number of input read pairs	1 M	10 M	100 M	1 M	10 M	100 M	1 M	10 M	100 M
Valid interaction	58.0%	57.9%	58.0%	62.7%	62.7%	62.7%	28.1%	28.1%	28.1%
Valid interaction (remove duplicates)	58.0%	57.7%	55.6%	62.7%	62.3%	58.9%	28.0%	27.5%	23.2%
Trans interaction	35.7%	35.5%	34.2%	34.1%	33.9%	32.1%	19.6%	19.2%	16.2%
Cis interaction	22.3%	22.2%	21.4%	28.6%	28.4%	26.8%	8.4%	8.2%	6.9%
Cis short-range interaction (<20Kb)	7.6%	7.5%	7.3%	13.4%	13.3%	12.6%	5.8%	5.7%	4.8%
Cis long-range interaction (>20Kb)	14.7%	14.6%	14.1%	15.2%	15.1%	14.2%	2.6%	2.6%	2.1%

Note that the *cis* /*trans* judgement based on fragmentary reference sequences might be unreliable because insufficient continuity increases the trans fraction and instead decreases the cis fraction.
