# Supplementary material for: Multifaceted Hi-C benchmarking: what makes a difference in chromosome-scale genome scaffolding?
Source: Gigascience. 2020 Jan 10;9(1):giz158. doi: 10.1093/gigascience/giz158 (PMC6952475; doi:10.1093/gigascience/giz158)
Supplement: giz158_Supplemental_Files [file giz158_supplemental_files.zip › Supplementary_Protocol_S2_to_support_multiple_enzymes.pdf]

Supplementary Protocol S2. Computational protocol to support the use of multiple enzymes

HiC-Pro

# Add restriction sites of Arima-specified enzymes to the `HiC-Pro/bin/utils/digest_genome.py` script

```
RE_cutsite = {
    "mboi": ["^GATC"],
    "dpnii": ["^GATC"],
    "hinfi-1": ["G^AATC"],
    "hinfi-2": ["G^ATTC"],
    "hinfi-3": ["G^AGTC"],
    "hinfi-4": ["G^ACTC"],
    "bglii": ["A^GATCT"],
    "hindiii": ["A^AGCTT"]}
```

# Run the script to make a restriction fragment file with multiple enzymes

```
digest_genome.py -r dpnii hinfi-1 hinfi-2 hinfi-3 hinfi-4 -o HiC-Pro_arima.bed genome.fasta
```

# Edit the HiC-pro `config-hicpro.txt` file for every combination of ligation sites

```
LIGATION_SITE = ATCGATC,GAATGATC,GATTGATC,GAGTGATC,GACTGATC,GAATAATC,GAATATTC,GAATAGTC,
GAATACTC,GATTAATC,GATTATTC,GATTAGTC,GATTACTC,GAGTAATC,GAGTATTC,GAGTAGTC,GAGTACTC,GACTAAT
C,GACTATTC,GACTAGTC,GACTACTC,GATCAATC,GATCATTC,GATCAGTC,GATCACTC
```

**`LIGATION_SITE` should be written in one line*

Juicer

# A python script for conversion of the restriction fragment file

hic-pro2juicer.py

```
from signal import signal, SIGPIPE, SIG_DFL
signal(SIGPIPE,SIG_DFL)

import sys

output = ""
pre_chr = ""

# get HiC-Pro digestion bed file as the 1st parameter
args = sys.argv
path=args[1]

with open(path) as f:
    for s_line in f:

        # split line by tab
        line=s_line.split()

        # chromosome name
        chr = line[0]

        # cutting site
        site = int(line[2])
        site = str(site)

        if chr==pre_chr:
        # same chromosome
            output += " " + site
        else:
            # new chromosome
            if output != "":
                print (output)

            output = chr + " " + site

        # save chromosome name
        pre_chr=chr

# print the last chromosome
print (output)
```

# Convert the restriction fragment file of HiC-Pro to the Juicer format

```
python hic-pro2juicer.py HiC-Pro_arima.bed > Juicer_arima.txt
```
