# Supplementary material for: Multifaceted Hi-C benchmarking: what makes a difference in chromosome-scale genome scaffolding?
Source: Gigascience. 2020 Jan 10;9(1):giz158. doi: 10.1093/gigascience/giz158 (PMC6952475; doi:10.1093/gigascience/giz158)
Supplement: giz158_Response_to_Reviewer_Comments_Original_Submission [file giz158_response_to_reviewer_comments_original_submission.pdf]

Author's Response To Reviewer Comments

Close

We have uploaded the PDF-formatted letter including the point-by-point descriptions of our revision of the manuscript as well as the manuscript with track changes.

Close
