# Supplementary material for: Multifaceted Hi-C benchmarking: what makes a difference in chromosome-scale genome scaffolding?
Source: Gigascience. 2020 Jan 10;9(1):giz158. doi: 10.1093/gigascience/giz158 (PMC6952475; doi:10.1093/gigascience/giz158)
Supplement: giz158_Response_to_Reviewer_Comments_Revision_1 [file giz158_response_to_reviewer_comments_revision_1.pdf]

Author's Response To Reviewer Comments

Close

We have uploaded a letter containing our response to the editor's and reviewer's request and comments.

Close
