# Supplementary material for: Multifaceted Hi-C benchmarking: what makes a difference in chromosome-scale genome scaffolding?
Source: Gigascience. 2020 Jan 10;9(1):giz158. doi: 10.1093/gigascience/giz158 (PMC6952475; doi:10.1093/gigascience/giz158)
Supplement: giz158_Reviewer_1_Report_Original_Submission — Matthew Zachariah DeMaere, Ph.D -- 7/24/2019 Reviewed [file giz158_reviewer_1_report_original_submission.pdf]

Reviewer Report

Title: Multifaceted Hi-C benchmarking: what makes a difference in chromosome-scale genome scaffolding?

Version: Original Submission **Date:** 7/24/2019

Reviewer name: Matthew Zachariah DeMaere, Ph.D

Reviewer Comments to Author:

General assessment
The authors have demonstrated an optimized protocol and accompanying quality control rationale for the reliable generation of good quality Hi-C sequencing libraries. To highlight the benefits of their method, a comparative analysis is employed against current commercial Hi-C library kits from the companies Phase Genomics, Arima Genomics and Dovetail Genomics.
The Hi-C protocol has proven to be a difficult sticking point for many labs, with inconsistent data quality and significant bench time being two factors which hold back a field with so much potential. Commercial kits, which have aimed to ameliorate both, have thus been quickly adopted.
I have separately assessed the Hi-C signal content of the generated libraries and find good agreement with those described by the authors. Notably, their libraries for all protocols contain the highest percentage of Hi-C pairs that I have yet observed. The iconHi-C protocol is an important advancement in library production and I applaud the authors for making their key findings public.
Comments overall
Quality of writing: The writing quality of the manuscript is acceptable, albeit the authors may wish to involve a third-party to assist in revising the text for grammatical errors and unusual word choice.
Though difficult to furnish, a more complete ground truth (genome) would have aided this study in conclusively interpreting the scaffolding results. However, I do not propose this be carried out.
As a parametric sweep, it would be helpful if the authors provided a simple table of the parameter ranges tested, even if supplementary.
The collection primary data-sets generated by the authors will be extremely useful for future work on Hi-C genome scaffolding and consequently so too will their 3d-dna and SALSA2 scaffolding results. In the interests of FAIR, I would strongly encourage the authors to submit all their downstream results to a public archive, such as Zenodo or Figshare.
Supplementary_Protocol_S2: this reviewer greatly appreciated the addition of patching notes for HiC-Pro, so as to support Arima's protocol design. Ideally, however, I encourage the authors to fork the HiC-Pro repository on github, make these changes and then submit a pull request back to the maintainers.
Comments by section
Background
In setting the stage, it would be helpful to readers if the authors made clear the motivation for why protocol optimisation should be pursued. What, if anything, is wrong with the status quo?
Results
Line 134: In the sentence containing "overt differences", the description of how the authors arrived at

their chosen set of parameters is extremely brief. Considering the success of their study, expanding on their observations here would be interesting.
Considering the wide range in quality of published Hi-C data-sets, the quality of Hi-C libraries in this study (regardless of protocol), which made it through to the stage of rapid-run and HiC-Pro, is extremely high. It would have been interesting to see HiC-Pro results for libraries which failed QC1 and QC2, so as to better calibrate expectations for the reader.
Did the authors take the restriction digest and ligation reactions to further timepoints? It would seem from figure 6 that neither are slowign down at their final timepoints. How have the authors convinced themselves that these edges of their parameter sweep represent optimal values?
Although the authors have explored length cut-offs for 3d-dna down to the default of SALSA2 (1000bp), it does not seem that they've attempted the converse; namely the performance of both tools at 15000bp. There exists a large difference in statistical confidence when counting Hi-C associations (between 1k and 15k), as well as the tendancy for smaller contigs to possess confounding features such as repeats. In this way, the potential for error when scaffolding grows as the contig size decreases. Parallel to this, the criteria governing the choice of default limits are not universal between developers. Holding in mind an understanding of the error processes in their tool, one developer might select a conservative value to minimise error while others might simply chose a limit based on their experience with computational scaling.
Line 25: Is it true that there is a lack of published articles on library protocol development? There are definitely articles which aim to extend or modify the Hi-C protocol, but perhaps a shortage of articles which only aim to optimise the existing protocol. Work that has been done, kept behind closed doors as intellectual property.
Comment line 208: I do not fault the authors for restricting their focus, but the potential depth of discussion on enzyme choice is much greater than what the authors have limited themselves; DpnII, HindIII and multi-enzyme digest. For instance, there are 18 commercially supplied 4-cutters with 4nt overhangs, whose 6 distinct sites effectively cover the spectrum of GC richness. The enzymes in this larger pool will possess differences which could positively or negatively affect the Hi-C protocol. Differences such as methylation sensitivity and fidelity in non-optimal conditions. In any study such as this, some words on limitations would be informative to readers.
Minor comments
Line 54: The sentence might read better as "... both within and between chromosomes, ..."
Line 57: Rather than dangling ", more recently" at the end, a more active voice would perhaps be "... which has recently prompted this method to be employed ..."
Line 64: "In early 2018" could begin a new paragraph.
Line 71: "has been limited."
Line 78: Perhaps the authors meant "desirable" rather than "anticipated".
Lines 84-86: The sentence beginning with "Despite its moderate global GC-content ..." seems to be missing a final prepositional phrase. What about GC heterogeneity and chromosomal sizes was suggested by the study?
Line 87: species'
Line 122: Does "unusable" mean "not valid" in the eyes of HiC-Pro? I recommend that the authors avoid introducing a new term and simply replace unusable with invalid in the body of the text.

Paragraph at 121: it may improve manuscript consistency to label the pilot-sequencing based QC step as QC3. This type of pilot-run based QC analysis is likely to become standard procedure and see further software support. The manuscript would benefit from introducing a convenient term of reference for all three stages of QC.
Line 142: insert "also" and change tense: "Increased duration of cell fixation also reduced the proportion..."
Line 170: More conventional QC language would be "passing controls" rather than being qualified by them. e.g. "All samples prepared using the iconHi-C protocol passed both controls." Stating that iconHi-C is compatible with these tests could mentioned separately.
Line 172: Here, you could employ the name QC3 if you named the post-sequencing test as suggested above.
Line 201: "Of those" seems unnecessary. Instead, "Assembly 8, which employed input Hi-c reads derived from both ..."
Line 240-241: It may be clearer to say "... or perhaps indicates an erroneous ..."
Lines 246, 251, 255: Unnecessary pluralisation "starting material"
Line 255-256: It may be better to replace "seems" with "is" and remove the comma before "to". "In preparing the starting materials, it is important to optimize the degree of cell fixation depending on your sample choice to obtain an optimal result in Hi-C scaffolding."
Line 261: It may be better to replace enhanced with increased.
Line 280: It may be better to replace "species-by-species" with "interspecies"
Line 296: insert comma "... libraries, including the one employing..."
Line 303-304: It may be clearer to say: "This procedure allowed us to minimize the PCR cycles, down to as few as five."
Line 317-319: I am not sure what is meant by "... operability of library insert lengths".
Line 320: It may improve continuity to begin with "This is especially so if Hi-C ..."
Line 331: Support for the observation that assembly analysis outcome improves with increasing number of Hi-C pairs can be found in the article describing the metagenomic Hi-C binner bin3C.
Line 348: remove comma after consider "... points to consider in order to ..."
Line 351: remove comma after maps "... to contact maps using an interactive ..."
Line 365-367: The sentence about cut-off length beginning with "One needs..." is unclear. This may simply be word choice.
Line 500-503: Recommend splitting this sentence in two and revising. "The restriction fragment..."
Comment: Employing downstream tools such as Juicebox and taking these assemblies as different starting points, it would be interesting to see how many hand-optimisation steps were required before achieving diminishing returns and how close to optimal was each final solution. This may require a more complete ground truth than to what the authors have access.
Figure 9: Condensing panels B, C and D into a single frame or adding grid lines would make it much easier to make comparative observations between the various assemblies. As well, carrying over the groupings from panel A onto the other panels. I accept that these layout operations may be difficult to achieve.

Methods

Are the methods appropriate to the aims of the study, are they well described, and are necessary controls included? Choose an item.

Conclusions

Are the conclusions adequately supported by the data shown? Choose an item.

Reporting Standards

Does the manuscript adhere to the journal's guidelines on minimum standards of reporting? Choose an item.

Choose an item.

Statistics

Are you able to assess all statistics in the manuscript, including the appropriateness of statistical tests used? Choose an item.

Quality of Written English

Please indicate the quality of language in the manuscript: Choose an item.

Declaration of Competing Interests

Please complete a declaration of competing interests, considering the following questions:

- Have you in the past five years received reimbursements, fees, funding, or salary from an organisation that may in any way gain or lose financially from the publication of this manuscript, either now or in the future?
- Do you hold any stocks or shares in an organisation that may in any way gain or lose financially from the publication of this manuscript, either now or in the future?
- Do you hold or are you currently applying for any patents relating to the content of the manuscript?
- Have you received reimbursements, fees, funding, or salary from an organization that holds or has applied for patents relating to the content of the manuscript?
- Do you have any other financial competing interests?
- Do you have any non-financial competing interests in relation to this paper?

If you can answer no to all of the above, write 'I declare that I have no competing interests' below. If your reply is yes to any, please give details below.

I declare that I have no competing interests.

I agree to the open peer review policy of the journal. I understand that my name will be included on my report to the authors and, if the manuscript is accepted for publication, my named report including any attachments I upload will be posted on the website along with the authors' responses. I agree for my report to be made available under an Open Access Creative Commons CC-BY license (http://creativecommons.org/licenses/by/4.0/). I understand that any comments which I do not wish to be included in my named report can be included as confidential comments to the editors, which will not be published.

Choose an item.

To further support our reviewers, we have joined with Publons, where you can gain additional credit to further highlight your hard work (see: https://publons.com/journal/530/gigascience). On publication of this paper, your review will be automatically added to Publons, you can then choose whether or not to claim your Publons credit. I understand this statement.

Yes Choose an item.
