# Supplementary material for: Multifaceted Hi-C benchmarking: what makes a difference in chromosome-scale genome scaffolding?
Source: Gigascience. 2020 Jan 10;9(1):giz158. doi: 10.1093/gigascience/giz158 (PMC6952475; doi:10.1093/gigascience/giz158)
Supplement: giz158_Reviewer_2_Report_Original_Submission — Derek Bickhart -- 7/29/2019 Reviewed [file giz158_reviewer_2_report_original_submission.pdf]

Reviewer Report

Title: Multifaceted Hi-C benchmarking: what makes a difference in chromosome-scale genome scaffolding?

Version: Original Submission **Date:** 7/29/2019

Reviewer name: Derek Bickhart

Reviewer Comments to Author:

Summary: In this manuscript, Kadota et al. present the results of a comparison of several kit-based methods for Hi-C library prep against a composite method they have developed called, iconHi-C. They test parameters related to library construction, RE digestion and even scaffolding software with the goal of identifying the best parameters for Hi-C scaffolding. Unfortunately, I do not think that their tests are always appropriate, and I worry that their use of extended duration ligation and restriction digestion adds more bias into Hi-C library preparation. My comments follow in the order in which I encountered an issue in the manuscript:

There appear to be many grammar and terminology errors in the submitted manuscript. As currently written, it would require professional English language editing to improve the text. As an example of the problem, I have identified the following grammar/terminology errors in the abstract alone:

Line 20: This sentence contains a redundant predicate: "a derivative of chromosome conformation capture" was, "originally developed as a means for characterizing chromosome conformation." I think that the authors should instead reformat the predicate of the sentence to refer to the fact that Hi-C is a "whole-genome" method -- in contrast to 3C -- and abbreviate the sentence from there.

Line 23: Hi-C data is used for "scaffolding." It does not "elongate" nucleotide sequences.

Line 25: Replace "the prevailing and irreplaceable use" with "Despite its prevalent use"

Line 38: Replace "and release the resultant" with "and demonstrate this technique on a"

And there are many more scattered throughout the rest of the manuscript.

Line 38: The authors did not "assemble" the Chinese softshell turtle but used existing contigs from the previously released assembly in scaffolding. The difference is slight but important: I expected to see new de novo contigs for this species in this manuscript because of this statement.

Fig 1: There are some misleading statistics in the figure. Firstly, Phase Genomics has several different kits for Hi-C preparation, and some of these kits (specifically the "Microbe kit") contain additional RE enzymes such as MluCI. I understand that the authors list the "animal versions" where applicable, but isn't this cherry-picking? Furthermore, RE enzyme digestion is likely dependent on RE motif prevalence in the target organism. Finally, what do the authors define as the "Hi-C reaction" row specification? Is this the required, post-fixation DNA concentration?

Line 67: While Bickhart et al. 2017 was one of the first demonstrated uses of LACHESIS, this was not the publication that described the method. Burton et al. 2013 should be cited here.

Line 111: I do not understand the sentence as written. What does, "exhibit a slight length recovery of restricted DNA fragments," mean? Did the authors mean that post-fixation, post-digested DNA should have a higher observed molecular weight on a gel?

Line 114: The difference in shift is quite small -- did the authors calculate an average or variance in shift that can be used to assess the quality of the preparation in a quantitative manner? The authors mention that they used an Agilent Tapestation, so these metrics should be available to them.
Line 117: Again, what is the size of the "shift" of gel electrophoresis products here? Can this be identified and used as a quantitative indicator of library quality rather than a qualitative indicator?
Line 139: Here is where a quantitative metric would help. The fragment distributions in the 10- and 30-minute fixation samples appear to be different. The 30-minute fraction appears to be universally higher. Isn't this significant enough to even be a qualitative indicator of differences in the prep?
Line 148: I am concerned with this interpretation of the data here. First, prolonged RE digests can exhibit star activity. Second, prolonged ligation can increase the proportion of chimeric fragments. Both enzymatic activities have measured rates of activity (typically stated in "units") that can be customized based on measured inputs to the reaction. Did the authors estimate the molarity of DNA for the ligation reactions or estimate the amount of time for DNA digestion based on the units of RE enzyme added? Finally, the authors claim that the last timepoint is the best in all cases -- was data collected for a 24-hour timepoint or an 8-hour timepoint for the digest and ligation, respectively?
Line 152: So the optimization was based on gel shift data? What was the goal of this optimization? I think that the authors may have simply optimized the shift of sample on the gel here. A sufficient test of optimization would involve the use of several different timepoints for each enzymatic prep in separate Hi-C libraries, and then using the data derived from these libraries in scaffolding.
Figure 7: Why was the blood sample not used with other kits? Why include it in the comparisons?
Line 171: Aren't you only showing the QC1 and QC2 results for iconHi-C in this figure? Also, the authors do not label their alignment-based quality control (via HiC-Pro) as a separate form of QC (e.g. QC3). This becomes confusing later in the paragraph, where the blank "QC" term is used indiscriminantly.
Line 218: What about short-read WGS alignment comparisons using FRC_align or comparisons with a third technology such as an optical map? I find that the use of the positions of 162 marker genes may be too small to identify fine-scale errors in scaffolding smaller contigs which is a known problem in Hi-C scaffolding (Bickhart et al. 2017). Additionally, assembly-to-assembly alignments and comparisons of WGS read-mapping profiles across these regions could be used to assess quality.
Line 260: Not "overassembly" but "chimeric scaffolding." This is a major issue with Hi-C that was not adequately measured by the authors in their quality control assessments. In fact, it is difficult to tell the overall "correctness" of scaffolding in each assembly apart from the BUSCO scores and scaffold N50 lengths provided by the authors -- each of which were not very informative by their own admission. More substantial scaffold quality assessment is needed.
Line 296: The authors refer to the Arima Hi-C assembly by number, but do not refer to the "library d" assembly by number. This is confusing to the reader.
Line 297: This could be a concern, but it is not addressed in the results by the authors. What noticeable effects on scaffold quality were determined by PCR over-amplification?
Line 315: I disagree with this interpretation. Figure 8 shows that the Arima kit had ~10% higher unique paired alignments than any of the iconHi-C preps. Was this discrepancy due to over-digestion and over-ligation in the iconHi-C protocol?
Line 333: While downsampling reads is a useful and novel comparison, did the authors consider that the same results could apply to the libraries obtained from the other kits?

Line 397: While agree with this conclusion, this study did not adequately measure erroneous scaffolds.
Line 399: I would recommend removing this entire paragraph as it does not add value to the manuscript. So long as gap regions are set to a fixed size (in the case of unknown gaps) the size of the gap sequence is irrelevant to downstream applications.

Methods

Are the methods appropriate to the aims of the study, are they well described, and are necessary controls included? Choose an item.

Conclusions

Are the conclusions adequately supported by the data shown? Choose an item.

Reporting Standards

Does the manuscript adhere to the journal's guidelines on minimum standards of reporting? Choose an item.

Choose an item.

Statistics

Are you able to assess all statistics in the manuscript, including the appropriateness of statistical tests used? Choose an item.

Quality of Written English

Please indicate the quality of language in the manuscript: Choose an item.

Declaration of Competing Interests

Please complete a declaration of competing interests, considering the following questions:

- Have you in the past five years received reimbursements, fees, funding, or salary from an organisation that may in any way gain or lose financially from the publication of this manuscript, either now or in the future?
- Do you hold any stocks or shares in an organisation that may in any way gain or lose financially from the publication of this manuscript, either now or in the future?
- Do you hold or are you currently applying for any patents relating to the content of the manuscript?
- Have you received reimbursements, fees, funding, or salary from an organization that holds or has applied for patents relating to the content of the manuscript?
- Do you have any other financial competing interests?

- Do you have any non-financial competing interests in relation to this paper?

If you can answer no to all of the above, write 'I declare that I have no competing interests' below. If your reply is yes to any, please give details below.

I declare that I have no competing interests.

I agree to the open peer review policy of the journal. I understand that my name will be included on my report to the authors and, if the manuscript is accepted for publication, my named report including any attachments I upload will be posted on the website along with the authors' responses. I agree for my report to be made available under an Open Access Creative Commons CC-BY license (http://creativecommons.org/licenses/by/4.0/). I understand that any comments which I do not wish to be included in my named report can be included as confidential comments to the editors, which will not be published.

Choose an item.

To further support our reviewers, we have joined with Publons, where you can gain additional credit to further highlight your hard work (see: https://publons.com/journal/530/gigascience). On publication of this paper, your review will be automatically added to Publons, you can then choose whether or not to claim your Publons credit. I understand this statement.

Yes Choose an item.
