# Supplementary material for: Multifaceted Hi-C benchmarking: what makes a difference in chromosome-scale genome scaffolding?
Source: Gigascience. 2020 Jan 10;9(1):giz158. doi: 10.1093/gigascience/giz158 (PMC6952475; doi:10.1093/gigascience/giz158)
Supplement: giz158_Reviewer_3_Report_Original_Submission — Jay Ghurye -- 7/29/2019 Reviewed [file giz158_reviewer_3_report_original_submission.pdf]

Reviewer Report

Title: Multifaceted Hi-C benchmarking: what makes a difference in chromosome-scale genome scaffolding?

Version: Original Submission **Date:** 7/29/2019

Reviewer name: Jay Ghurye

Reviewer Comments to Author:

I thoroughly enjoyed reading the manuscript benchmarking HiC data for assembly through different aspects. To my knowledge, this is the first study that comprehensively studies this topic. This is a novel study and I think the topic of the manuscript will receive tremendous interest. However, I have some queries/concerns that I would like authors to address.

- I see in Supplementary Table S2 the percentage of long and short range read pairs. However less than 20 kbp and greater than 20 kbp is not very informative. Can you stratify more? Like percentage of read pairs between 10k -100k, 100k -1Mbp, 1Mbp-10Mbp, and 10 Mbp and above. This would highlight in what range the utility of iconHi-C protocol.
- I understand from Figure 9 the bulk assembly contiguity statistics. However, it doesn't tell much about how correct is the assembly. I would like to see a contact matrix for a couple of assemblies that authors think are the best. Also, a heatmap for iconHi-C assembly constructed using other Hi-C datasets is also interesting to see. Such a comparison would highlight the valuable contact information that's probably missed in iconHi-C or other Hi-C datasets.
- I saw the library QC results for GM12878, however I was not able to see any scaffolding results for it with different Hi-C datasets. Since we have a known reference genome, we can get a solid evidence that which parameter setting and what type of Hi-C library provides the best assembly in terms of both contiguity and accuracy.
- This may be out of the scope of this manuscript. Did authors find out minimum amount Hi-C read pairs required for good scaffolding? Such a discussion or recommendation would guide the amount of sequencing needed for the scaffolding project and would reduce the cost.
- The scope of the manuscript is mainly understanding the effect of different parameters on scaffolding. But, do authors have any intuition about usage of iconHi-C in other 3D genomic application such as detecting TADs, chromatin loops, etc? Some discussion would be helpful.
- Figure 8 and Figure 9 is kind of hard to understand. I would appreciate if the data is displayed in a tabular format.

Methods

Are the methods appropriate to the aims of the study, are they well described, and are necessary controls included? Choose an item.

Conclusions

Are the conclusions adequately supported by the data shown? Choose an item.

Reporting Standards

Does the manuscript adhere to the journal's guidelines on minimum standards of reporting? Choose an item.

Choose an item.

Statistics

Are you able to assess all statistics in the manuscript, including the appropriateness of statistical tests used? Choose an item.

Quality of Written English

Please indicate the quality of language in the manuscript: Choose an item.

Declaration of Competing Interests

Please complete a declaration of competing interests, considering the following questions:

- Have you in the past five years received reimbursements, fees, funding, or salary from an organisation that may in any way gain or lose financially from the publication of this manuscript, either now or in the future?
- Do you hold any stocks or shares in an organisation that may in any way gain or lose financially from the publication of this manuscript, either now or in the future?
- Do you hold or are you currently applying for any patents relating to the content of the manuscript?
- Have you received reimbursements, fees, funding, or salary from an organization that holds or has applied for patents relating to the content of the manuscript?
- Do you have any other financial competing interests?
- Do you have any non-financial competing interests in relation to this paper?

If you can answer no to all of the above, write 'I declare that I have no competing interests' below. If your reply is yes to any, please give details below.

I am an employee of Dovetail Genomics, a commercial HiC sequencing technology company.

I agree to the open peer review policy of the journal. I understand that my name will be included on my report to the authors and, if the manuscript is accepted for publication, my named report including any attachments I upload will be posted on the website along with the authors' responses. I agree for my report to be made available under an Open Access Creative Commons CC-BY license (http://creativecommons.org/licenses/by/4.0/). I understand that any comments which I do not wish to

be included in my named report can be included as confidential comments to the editors, which will not be published.

Choose an item.

To further support our reviewers, we have joined with Publons, where you can gain additional credit to further highlight your hard work (see: https://publons.com/journal/530/gigascience). On publication of this paper, your review will be automatically added to Publons, you can then choose whether or not to claim your Publons credit. I understand this statement.

Yes Choose an item.
